# Supplementary material for: Cu-mediated enantioselective C–H alkynylation of ferrocenes with chiral BINOL ligands
Source: Nat Commun. 2023 Nov 24;14:7698. doi: 10.1038/s41467-023-43278-z (PMC10673954; doi:10.1038/s41467-023-43278-z)
Supplement: Supplementary file 1 — Supplementary Information [file 41467_2023_43278_MOESM1_ESM.pdf]

Supplementary Information

for

Cu-Mediated Enantioselective C–H Alkynylation of Ferrocenes with Chiral BINOL Ligands

Xin Kuang[†,‡], Jian-Jun Li[‡], Tao Liu[‡], Chang-Hua Ding,[†] Kevin Wu,[¶]

Peng Wang,[*,‡,§] and Jin-Quan Yu[*,¶]

[‡]*State Key Laboratory of Organometallic Chemistry, Center for Excellence in Molecular Synthesis, Shanghai Institute of Organic Chemistry, CAS 345 Lingling Road, Shanghai 200032, P. R. China*

[†]*School of Science, Shanghai University, 99 Shang-Da Road, Shanghai 200444, P. R. China*

[¶]*The Scripps Research Institute (TSRI), 10550 North Torrey Pines Road, La Jolla, CA 92037, USA*

[§]*School of Chemistry and Materials Science, Hangzhou Institute for Advanced Study, University of Chinese Academy of Sciences, 1 Sub-lane Xiangshan, Hangzhou 310024, P. R. China*

Supplementary Methods

1. General Information

$Cu(OAc)_2$, CuOAc was purchased from Energy Chemical. BINOL ligands were synthesized according to known references.[1-6] Other reagents were purchased from the highest purity grades TCI, Sigma-Aldrich, Acros, Adamas-beta, Accela, d&b, J&K, 9-Ding, Bidepharm and Energy Chemical and were used without further purification unless otherwise stated. Tetrahydrofuran (THF), acetonitrile (CH_3CN), dichloromethane (CH_2Cl_2) and *N*,*N*-dimethylformamide (DMF) were dried using a solvent purification system (Vigor). DMSO was purchased from J&K or Energy Chemical. The extent of the reaction was monitored by thin layer chromatography (TLC) on 0.25 mm silica gel HSGF254. TLC plates were visualized after treatment with UV light (254 nm).

NMR spectra were recorded on Varian 400, Bruker 400, and Agilent 400 (400 MHz for ^{1}H; 375 MHz for ^{19}F; 100 MHz for ^{13}C) spectrometers. Chemical shifts (δ) are quoted in parts per million (ppm) with reference to TMS (^{1}H NMR 0.0 ppm) and $CDCl_3$ (^{13}C NMR 77.0 ppm). The following abbreviations are used to explain multiplicity: s = singlet, d = doublet, t = triplet, q = quartet, m = multiplet and br = broad. The coupling constant *J* is reported in Hertz units (Hz). High-resolution mass spectra (HRMS) were recorded on an Agilent mass spectrometer and Thermo Fisher Scientific LTQ FTICR-MS using ESI–TOF. HPLC analyses were performed on a Thermo Fisher UltiMate 3000 liquid chromatograph.

2. Experimental Section

2.1 Preparation of Substrates

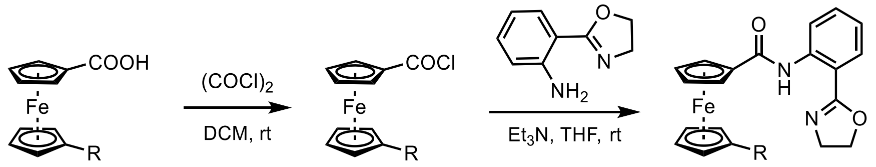

General Procedure A for the Preparation of Substrates: To a tetrahydrofuran solution of ferrocenecarboxylic acid chloride, prepared from the corresponding ferrocenecarboxylic acid (1.0 equiv.), was added the mixture of 2-(4,5-dihydrooxazol-2-yl)aniline (1.0 equiv.), Et_3N (1.5 equiv.) and dry THF (0.5 M). This reaction mixture was stirred at room temperature overnight. Upon completion, the reaction mixture was concentrated under reduced pressure and the residue was purified by silica gel column chromatography with a gradient eluent of petroleum ether/ethyl acetate to provide the desired product.

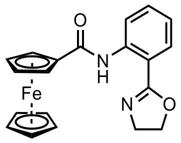

1a

***N*-(2-(4,5-Dihydrooxazol-2-yl)phenyl)ferrocenyl-1-carboxamide**

1a was prepared from ferrocenecarboxylic acid (5.0 mmol) following the general procedure A. Purification by flash chromatography on silica gel (PE/EA = 20/1) gave the product as an orange solid (1.65 g, 88% yield), R_f = 0.6 (PE/EA = 10/1). ^{1}H NMR (400 MHz, $CDCl_3$) δ 12.45 (s, 1H), 8.88 (d, *J* = 8.4 Hz, 1H), 7.88 (d, *J* = 8.0 Hz, 1H), 7.48 (t, *J* = 7.6 Hz, 1H), 7.06 (t, *J* = 8.0, 1H), 4.94 (s, 2H), 4.46–4.37 (m, 4H), 4.29–4.18 (m, 7H); ^{13}C NMR (100 MHz, $CDCl_3$) δ 169.9, 164.9, 140.4, 132.6, 129.2, 121.6, 119.4, 112.6, 77.0, 70.8, 69.8, 68.7, 66.1, 54.8; HRMS (ESI-TOF) *m/z* calculated for $C_{20}H_{18}N_2O_2NaFe$ $[M+Na]^+$: 397.0610, found: 397.0608.

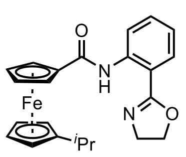

1b

***N*-(2-(4,5-Dihydrooxazol-2-yl)phenyl)-1'-isopropyl ferrocenyl-1-carboxamide**

1b was prepared from the corresponding ferrocenecarboxylic acid[7] (2.0 mmol) following the general procedure A. Purification by flash chromatography on silica gel (PE/EA = 20/1) gave the product as an orange solid (743 mg, 89% yield), R_f = 0.6 (PE/EA = 10/1). ^{1}H NMR (400 MHz, $CDCl_3$) δ 12.42 (s, 1H), 8.90 (d, *J* = 8.4 Hz, 1H), 7.87 (d, *J* = 8.0 Hz, 1H), 7.47 (t, *J* = 7.8 Hz, 1H), 7.05 (t, *J* = 7.6 Hz, 1H), 4.90 (s, 2H), 4.44–4.32 (m, 4H), 4.22 (t, *J* = 9.6 Hz,

2H), 4.11 (s, 2H), 4.08 (s, 2H), 2.67–2.51 (m, 1H), 1.14 (d, J = 6.4 Hz, 6H); ^{13}C NMR (100 MHz, $CDCl_3$) δ 169.8, 164.8, 140.3, 132.5, 129.2, 121.5, 119.3, 112.5, 98.4, 76.7, 71.3, 69.0, 68.9, 67.7, 66.0, 54.7, 27.0, 23.4; HRMS (ESI-TOF) *m/z* calculated for $C_{23}H_{24}N_2O_2NaFe$ $[M+Na]^+$: 439.1079, found: 439.1074.

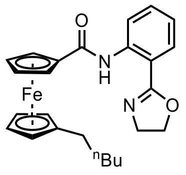

1c

***N*-(2-(4,5-Dihydrooxazol-2-yl)phenyl)-1'-pentyl ferrocenyl-1-carboxamide**

1c was prepared from the corresponding ferrocenecarboxylic acid[7] (2.0 mmol) following the general procedure A. Purification by flash chromatography on silica gel (PE/EA = 20/1) gave the product as a reddish brown solid (432 mg, 49% yield), R_f = 0.6 (PE/EA = 10/1). ^{1}H NMR (400 MHz, $CDCl_3$) δ 12.41 (s, 1H), 8.91 (d, J = 8.4 Hz, 1H), 7.87 (d, J = 8.0 Hz, 1H), 7.47 (t, J = 8.0 Hz, 1H), 7.04 (t, J = 7.8 Hz, 1H), 4.87 (s, 2H), 4.44–4.31 (m, 4H), 4.22 (t, J = 9.4 Hz, 2H), 4.08 (s, 4H), 2.24 (t, J = 7.8 Hz, 2H), 1.47–1.33 (m, 2H), 1.27–1.11 (m, 4H), 0.82 (t, J = 6.8 Hz, 3H); ^{13}C NMR (100 MHz, $CDCl_3$) δ 169.6, 164.8, 140.4, 132.5, 129.2 , 121.5, 119.3, 112.5, 91.0, 77.2, 71.4, 69.6, 69.0, 68.9, 66.0, 54.7, 31.6, 30.7, 28.4, 22.3, 13.9; HRMS (ESI-TOF) *m/z* calculated for $C_{25}H_{28}N_2O_2NaFe$ $[M+Na]^+$: 467.1392, found: 467.1398.

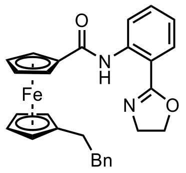

1d

***N*-(2-(4,5-Dihydrooxazol-2-yl)phenyl)-1'-phenethyl ferrocenyl-1-carboxamide**

1d was prepared from the corresponding ferrocenecarboxylic acid[7] (2.0 mmol) following the general procedure A. Purification by flash chromatography on silica gel (PE/EA = 20/1) gave the product as an orange solid(689 mg, 72% yield), R_f = 0.6 (PE/EA = 10/1). ^{1}H NMR (400 MHz, $CDCl_3$) δ 12.43 (s, 1H), 8.90 (d, J = 8.4 Hz, 1H), 7.88 (d, J = 8.4 Hz, 1H), 7.47 (t, J = 8.0 Hz, 1H), 7.26–7.11 (m, 3H), 7.06 (t, J = 7.8 Hz, 3H), 4.88 (s, 2H), 4.43–4.26 (m, 4H), 4.20–4.00 (m, 6H), 2.78–2.68 (m, 2H), 2.63–2.53 (m, 2H); ^{13}C NMR (100 MHz, $CDCl_3$) δ 169.6, 164.8, 141.7, 140.3, 132.6, 129.2, 128.2, 128.1, 125.7, 121.6, 119.4, 112.5, 89.9, 77.3, 71.3, 69.8, 69.2, 69.0, 66.0, 54.6, 37.4, 30.5; HRMS (ESI-TOF) *m/z* calculated for $C_{28}H_{26}N_2O_2NaFe$ $[M+Na]^+$: 501.1236, found: 501.1231.

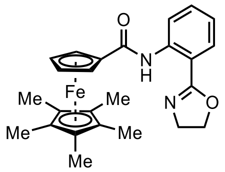

1e

N-(2-(4,5-Dihydrooxazol-2-yl)phenyl)-1',2',3',4',5'-pentamethyl ferrocenyl-1-carboxamide

1e was prepared from the corresponding ferrocenecarboxylic acid[8,9] (1.0 mmol) following the general procedure A. Purification by flash chromatography on silica gel (PE/EA = 20/1) gave the product as an orange solid (92 mg, 21% yield), R_f = 0.6 (PE/EA = 5/1). ^{1}H NMR (400 MHz, $CDCl_3$) δ 12.22 (s, 1H), 8.99 (d, J = 8.4 Hz, 1H), 7.87 (d, J = 7.8 Hz, 1H), 7.47 (t, J = 8.0 Hz, 1H), 7.04 (t, J = 7.6 Hz, 1H), 4.51–4.34 (m, 4H), 4.22 (t, J = 9.6 Hz, 2H), 3.97 (s, 2H), 1.80 (s, 15H); ^{13}C NMR (100 MHz, $CDCl_3$) δ 168.9, 164.8, 140.6, 132.6, 129.2, 121.3, 119.2, 112.3, 81.3, 77.8, 75.1, 71.0, 66.0, 54.7, 10.4; HRMS (ESI-TOF) *m/z* calculated for $C_{25}H_{29}N_2O_2Fe$ [M+H]$^+$: 445.1573, found: 445.1559.

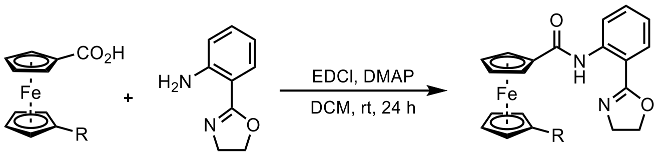

General Procedure B for the Preparation of Substrates: A corresponding ferrocenecarboxylic acid[7] (1.0 equiv.), 2-(4,5-dihydrooxazol-2-yl)aniline (1.0 equiv.) and DMAP (1.0 eq.) were charge to a 50 mL flask and dissolved in dry dichloromethane (0.2 M) under nitrogen. The dichloromethane solution of EDCI (2.0 equiv.) was added to above solution at 0 °C. The mixture was stirred at room temperature for 24 hours. Upon completion, the reaction mixture was concentrated under reduced pressure and the residue was purified by silica gel column chromatography with a gradient eluent of petroleum ether/ethyl acetate to provide the desired products.

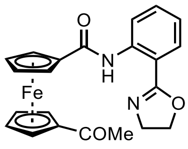

1f

1'-Acetyl-N-(2-(4,5-dihydrooxazol-2-yl)phenyl)ferrocenyl-1-carboxamide

1f was prepared from the corresponding ferrocenecarboxylic acid[7] (2.0 mmol) following the general procedure B. Purification by flash chromatography on silica gel (PE/EA = 4/1) gave the product as an orange-red solid (531 mg, 64% yield), R_f = 0.3 (PE/EA = 2/1). ^{1}H NMR (400 MHz, $CDCl_3$) δ 12.53 (s, 1H), 8.86 (d, J = 8.4 Hz, 1H), 7.89 (d, J = 7.8 Hz, 1H), 7.49 (t, J = 7.8 Hz, 1H), 7.09 (t, J = 7.8 Hz, 1H), 4.96 (s, 2H), 4.78 (s, 2H), 4.52 (s, 2H), 4.48–4.39 (m, 4H), 4.27 (t, J = 9.4 Hz, 2H), 2.33 (s, 3H); ^{13}C NMR (100 MHz, $CDCl_3$) δ 201.5, 168.1, 164.9, 140.0, 132.6, 129.3, 122.0, 119.4, 112.8, 80.4, 78.6, 73.8, 72.5, 71.0, 70.1, 66.2, 54.6, 27.5; HRMS (ESI-TOF) *m/z* calculated for $C_{22}H_{20}N_2O_3NaFe$ [M+Na]$^+$: 439.0716, found: 439.0713.

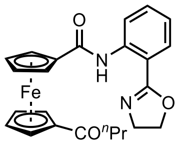

1g

1'-Butyryl-*N*-(2-(4,5-dihydrooxazol-2-yl)phenyl)ferrocenyl-1-carboxamide

1g was prepared from the corresponding ferrocenecarboxylic acid[7] (3.0 mmol) following the general procedure B. Purification by flash chromatography on silica gel (PE/EA = 4/1) gave the product as a reddish brown solid (638 mg, 48% yield), R_f = 0.4 (PE/EA = 2/1). ^{1}H NMR (400 MHz, $CDCl_3$) δ 12.53 (s, 1H), 8.87 (d, *J* = 8.4 Hz, 1H), 7.89 (d, *J* = 8.0 Hz, 1H), 7.49 (t, *J* = 8.0 Hz, 1H), 7.09 (t, *J* = 7.6 Hz, 1H), 4.96 (s, 2H), 4.80 (s, 2H), 4.51 (s, 2H), 4.48–4.39 (m, 4H), 4.27 (t, *J* = 9.4 Hz, 2H), 2.62 (t, *J* = 7.4 Hz, 2H), 1.70–1.57 (m, 2H), 0.89 (t, *J* = 7.4 Hz, 3H); ^{13}C NMR (100 MHz, $CDCl_3$) δ 203.8, 168.3, 164.9, 140.0, 132.6, 129.3, 122.0, 119.4, 112.7, 80.3, 78.5, 73.7, 72.4, 70.6, 70.0, 66.2, 54.6, 41.7, 17.6, 13.8; HRMS (ESI-TOF) *m/z* calculated for $C_{24}H_{24}N_2O_3NaFe$ [M+Na]$^+$: 467.1029, found: 467.1026.

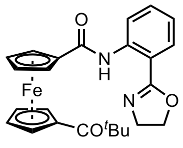

1h

1'-Pivaloyl-*N*-(2-(4,5-dihydrooxazol-2-yl)phenyl)ferrocenyl-1-carboxamide

1h was prepared from the corresponding ferrocenecarboxylic acid[7] (4.4 mmol) following the general procedure B. Purification by flash chromatography on silica gel (PE/EA = 4/1) gave the product as a reddish brown solid (956 mg, 47% yield), R_f = 0.5 (PE/EA = 2/1). ^{1}H NMR (400 MHz, $CDCl_3$) δ 12.50 (s, 1H), 8.86 (d, *J* = 8.4 Hz, 1H), 7.90 (d, *J* = 7.6 Hz, 1H), 7.48 (t, *J* = 7.2 Hz, 1H), 7.08 (t, *J* = 7.6 Hz, 1H), 4.97 (s, 2H), 4.91 (s, 2H), 4.52–4.37 (m, 6H), 4.29 (t, *J* = 9.4 Hz, 2H), 1.30 (s, 9H); ^{13}C NMR (100 MHz, $CDCl_3$) δ 209.6, 168.6, 165.0, 140.1, 132.6, 129.3, 121.9, 119.4, 112.7, 78.2, 77.8, 73.6, 73.1, 72.1, 69.8, 66.2, 54.7, 44.3, 27.9; HRMS (ESI-TOF) *m/z* calculated for $C_{25}H_{26}N_2O_3NaFe$ [M+Na]$^+$: 481.1185, found: 481.1179.

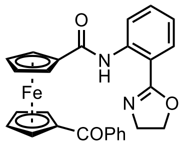

1i

1'-Benzoyl-*N*-(2-(4,5-dihydrooxazol-2-yl)phenyl)ferrocenyl-1-carboxamide

1i was prepared from the corresponding ferrocenecarboxylic acid[7] (2.8 mmol) following the general procedure B. Purification by flash chromatography on silica gel (PE/EA = 4/1) gave the product as a reddish brown solid (630 mg, 47% yield), R_f = 0.7 (PE/EA = 2/1). ^{1}H NMR (400 MHz, $CDCl_3$) δ 12.49 (s, 1H), 8.79 (d, *J* = 8.4 Hz, 1H), 7.89–7.80 (m, 3H), 7.46 (t, *J* = 7.8 Hz, 2H), 7.36 (t, *J* = 7.6 Hz, 2H), 7.07 (t, *J* = 7.6 Hz, 1H), 4.97 (s, 4H), 4.59 (s, 2H), 4.47–4.32 (m, 4H), 4.15 (t, *J* = 9.6 Hz, 2H); ^{13}C NMR (100 MHz, $CDCl_3$) δ 198.1, 168.1, 164.8,140.0, 139.1, 132.6, 131.6, 129.2, 128.1, 121.9, 119.4, 112.8, 79.2, 78.7, 74.5, 73.2, 72.7,70.2, 66.1, 54.5; HRMS (ESI-TOF) *m/z* calculated for $C_{27}H_{22}N_2O_3NaFe$ [M+Na]$^+$: 501.0872, found: 501.0868.

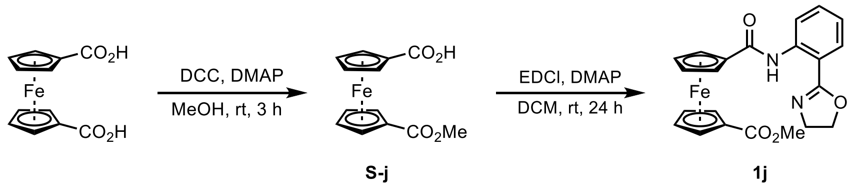

1,1'-Ferrocnedocarboxylic acid (5.0 mmol, 1.0 equiv.) was charge to a 50 mL Schlenk flask and dissolved in MeOH (20 mL) under nitrogen. DCC (5.0 mmol, 1.0 equiv.) and DMAP (5.0 mmol, 1.0 equiv.) were added to above solution at room temperature. The mixture was stirred at room temperature for 3.0 hours. Upon completion, the reaction mixture was concentrated under reduced pressure and the residue was purified by silica gel column chromatography with a gradient eluent of methanol/dichloromethane to afford **S-j** as an orange solid (235 mg, 16% yield).

1j was prepared from **S-j** (1.7 mmol) following the general procedure B. Purification by flash chromatography on silica gel (PE/EA = 4/1) gave the product as an orange solid (178 mg, 25% yield), $R_f = 0.3$ (PE/EA = 2/1).

Methyl 1'-((2-(4,5-dihydrooxazol-2-yl)phenyl)carbamoyl)ferrocenyl-1-carboxylate

^{1}H NMR (400 MHz, $CDCl_3$) δ 12.51 (s, 1H), 8.87 (d, J = 8.4 Hz, 1H), 7.87 (d, J = 8.0 Hz, 1H), 7.48 (t, J = 8.0 Hz, 1H), 7.06 (t, J = 7.8 Hz, 1H), 4.97 (s, 2H), 4.84 (s, 2H), 4.46–4.35 (m, 6H), 4.25 (t, J = 9.6 Hz, 2H), 3.65 (s, 3H); ^{13}C NMR (100 MHz, $CDCl_3$) δ 170.8, 168.1, 164.8, 140.1, 132.5, 129.1, 121.8, 119.3, 112.6, 78.4, 72.9, 72.6, 72.4, 71.4, 69.9, 66.1, 54.6, 51.5; HRMS (ESI-TOF) *m/z* calculated for $C_{22}H_{20}N_2O_4NaFe$ $[M+Na]^+$: 455.0665, found: 455.0655.

2.2 Experimental Optimization for Cu-Mediated C–H Alkynylation

2.2.1 Loading of $Cu(OAc)_2$[a]

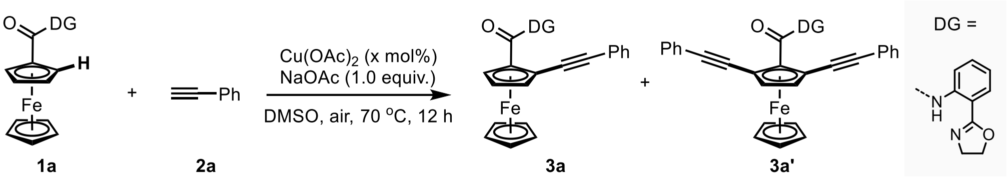

Entry	x (mol%)	3a+3a' (%)	3a/3a'	1a (%)
1	20	5	> 25/1.0	77
2	30	8	> 25/1.0	71
3	40	11	> 25/1.0	70
4	50	13	> 25/1.0	66
5	60	13	> 25/1.0	64
6	80	13	> 25/1.0	65
7	100	14	> 25/1.0	58

[a]Reaction conditions: **1a** (0.1 mmol), **2a** (0.3 mmol), $Cu(OAc)_2$ (x mol%), NaOAc (1.0 equiv.), DMSO (5.0 mL), air, 70 °C, 12 h. The yield was determined by ^1H NMR using TTCE as internal standard.

Supplementary Table 1. Copper(II) acetate loading screen in the absence of exogenous ligand.

2.2.2 Oxidants[a]

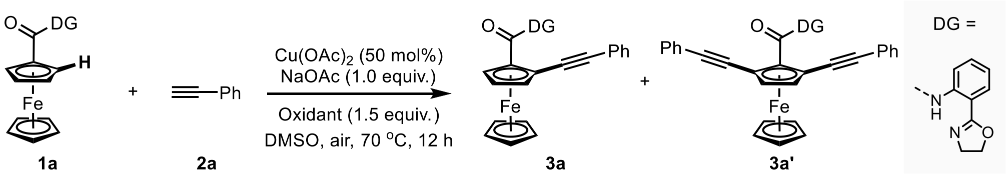

Entry	Oxidants	3a+3a' (%)	3a/3a'	1a (%)	Entry	Oxidants	3a+3a' (%)	3a/3a'	1a (%)
1	Ag_2O	21	9.0/1.0	53	10	BQ	-	-	94
2	Ag_2CO_3	29	4.8/1.0	47	11	Selectfluor	-	-	71
3	AgOAc	16	10/1.0	58	12	NMO	13	> 25/1.0	65
4	$AgNO_3$	7	> 25/1.0	76	13	Oxone	-	-	58
5	AgOTs	trace	> 25/1.0	74	14	MnO_2	19	> 25/1.0	62
6	Ag_2SO_4	15	> 25/1.0	55	15	$Mn(OAc)_3 \cdot 2H_2O$	10	9.0/1.0	53
7	Ag_3PO_4	-	-	82	16	$Na_2CO_3 \cdot 1.5H_2O_2$	18	> 25/1.0	55
8	AgTFA	5	> 25/1.0	78	17	$NaIO_4$	13	> 25/1.0	59
9	PIDA	17	10/1.0	58	18	$K_2S_2O_8$	-	-	54

[a]Reaction conditions: **1a** (0.1 mmol), **2a** (0.3 mmol), $Cu(OAc)_2$ (50 mol%), Oxidant (1.5 equiv.), NaOAc (1.0 equiv.), DMSO (5.0 mL), air, 70 °C, 12 h. The yield was determined by ^1H NMR using TTCE as internal standard.

Supplementary Table 2. Oxidant screening in the absence of exogenous ligand.

2.2.3 Loading of Ag_2CO_3[a]

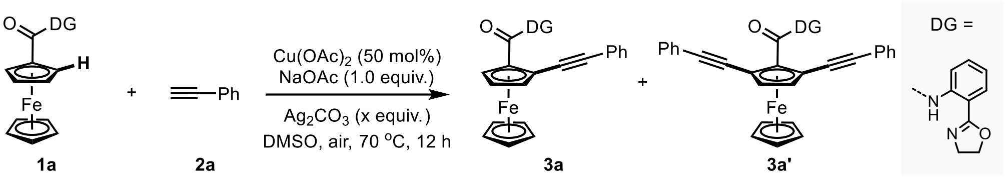

Entry	x (equiv.)	3a+3a' (%)	3a/3a'	1a (%)
1	0.5	23	10/1.0	63
2	1.0	29	6.2/1.0	49
3	1.5	29	4.8/1.0	47
4	2.0	28	3.0/1.0	33

[a]Reaction conditions: **1a** (0.1 mmol), **2a** (0.3 mmol), $Cu(OAc)_2$ (50 mol%), Ag_2CO_3 (x equiv.), NaOAc (1.0 equiv.), DMSO (5.0 mL), air, 70 °C, 12 h. The yield was determined by ^1H NMR using TTCE as internal standard.

Supplementary Table 3. Silver carbonate oxidant loading screen in the absence of exogenous ligand.

2.2.4 Ligand Effect[a]

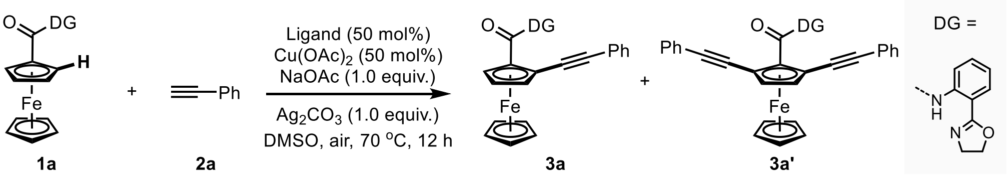

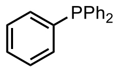	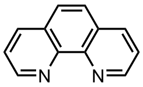	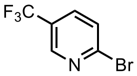	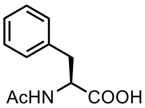
**L1, 3a+3a'**: 14%<br>**3a/3a'** = 6.0/1.0<br>**1a**: 23%	**L3, 3a+3a'**: 6%<br>**3a/3a'** > 25/1.0<br>**1a**: 78%	**L23, 3a+3a'**: 22%<br>**3a/3a'** = 10/1.0<br>**1a**: 53%	**L5, 3a+3a'**: 22%<br>**3a/3a'** = 8.0/1.0<br>**1a**: 55%
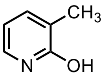	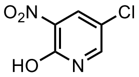	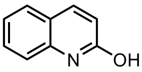	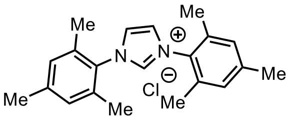
**L24, 3a+3a'**: 23%<br>**3a/3a'** = 7.0/1.0<br>**1a**: 47%	**L25, 3a+3a'**: 20%<br>**3a/3a'** = 20/1.0<br>**1a**: 62%	**L26, 3a+3a'**:27%<br>**3a/3a'** = 6.0/1.0<br>**1a**: 42%	**L27, 3a+3a'**:20%<br>**3a/3a'** = 12/1.0<br>**1a**: 58%
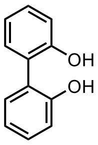	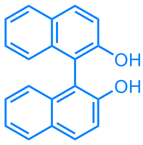	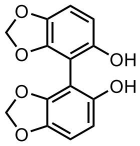	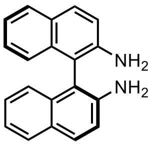
**L9, 3a+3a'**: 27%<br>**3a/3a'** = 8.0/1.0<br>**1a**: 54%	(±)-**L10, 3a+3a'**: 54%<br>**3a/3a'** = 1.0/1.0<br>**1a**: 14%	(±)-**L28, 3a+3a'**: 31%<br>**3a/3a'** = 5.0/1.0<br>**1a**: 49%	(*S*)-**L29, 3a+3a'**: 25%<br>**3a/3a'** = 6.0/1.1<br>**1a**: 55%

[a]Reaction conditions: **1a** (0.1 mmol), **2a** (0.3 mmol), $Cu(OAc)_2$ (50 mol%), Ag_2CO_3 (1.0 equiv.), Ligand (50 mol%), NaOAc (1.0 equiv.), DMSO (5.0 mL), air, 70 °C, 12 h. The yield was determined by ^1H NMR using TTCE as internal standard.

Supplementary Table 4. Ligand screening for the racemic C–H alkynylation.

2.2.5 Cu(II) Salts[a]

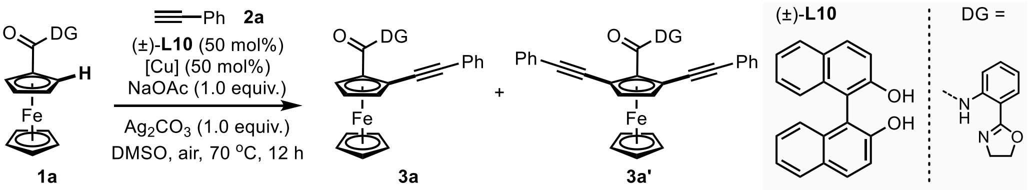

Entry	[Cu]	3a+3a' (%)	3a/3a'	1a (%)
1	$Cu(OAc)_2$	54	1.0/1.0	14
2	$Cu(PhCO_2)_2$	53	1.5/1.0	19
3	$Cu(HCO_2)_2$	42	2.2/1.0	31
4	$Cu(1\text{-}AdCO_2)_2$	49	1.0/1.3	9
5	$Cu(TFA)_2\cdot xH_2O$	53	4.3/1.0	23
6	$Cu(OTf)_2$	40	3.0/1.0	34
7	$Cu(ClO_4)_2\cdot 6H_2O$	32	10/1.0	47
8	CuF_2	43	1.5/1.0	29

[a]Reaction conditions: **1a** (0.1 mmol), **2a** (0.3 mmol), [Cu] (50 mol%), (±)-**L10** (50 mol%), Ag_2CO_3 (1.0 equiv.), NaOAc (1.0 equiv.), DMSO (5.0 mL), air, 70 °C, 12 h. The yield was determined by ^1H NMR using TTCE as internal standard.

Supplementary Table 5. Cu(II) source screen when using racemic L10 as ligand.

2.2.6 Loading of $Cu(OAc)_2$[a]

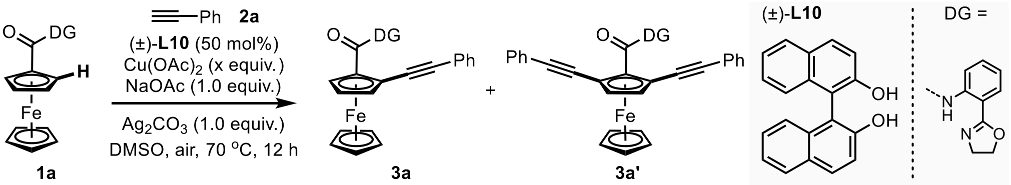

Entry	x (equiv.)	3a+3a' (%)	3a/3a'	1a (%)
1	0.5	54	1.0/1.0	14
2	0.7	56	1.7/1.0	9
3	1.0	57	2.8/1.0	2
4	1.2	50	24/1.0	12

[a]Reaction conditions: **1a** (0.1 mmol), **2a** (0.3 mmol), $Cu(OAc)_2$ (x equiv.), (±)-**L10** (50 mol%), Ag_2CO_3 (1.0 equiv.), NaOAc (1.0 equiv.), DMSO (5.0 mL), air, 70 °C, 12 h. The yield was determined by ^1H NMR using TTCE an internal standard.

Supplementary Table 6. Cu(II) acetate loading screen when using racemic L10 as ligand.

2.2.7 Loading of Ag_2CO_3[a]

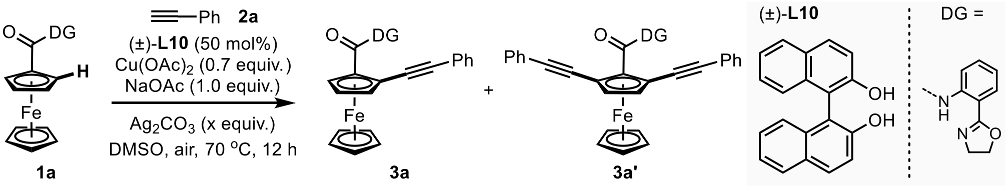

Entry	x (equiv.)	3a+3a' (%)	3a/3a'	1a (%)
1	1.5	58	1.9/1.0	13
2	1.0	56	1.7/1.0	9
3	0.5	57	2.6/1.0	15
4	0.4	53	3.4/1.0	14

[a]Reaction conditions: **1a** (0.1 mmol), **2a** (0.3 mmol), $Cu(OAc)_2$ (0.7 equiv.), (±)-**L10** (50 mol%), Ag_2CO_3 (x equiv.), NaOAc (1.0 equiv.), DMSO (5.0 mL), air, 70 °C, 12 h. The yield was determined by ^1H NMR using TTCE as internal standard.

Supplementary Table 7. Silver carbonate loading screen when using racemic L10 as ligand.

2.2.8 Cu(I) Salts[a]

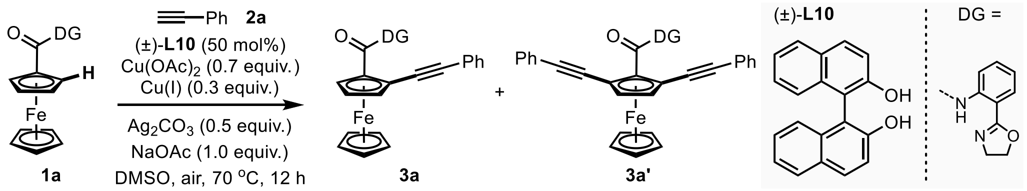

Entry	Cu(I)	3a+3a' (%)	3a/3a'	1a (%)
1	CuBr	42	> 25/1.0	26
2	CuCl	38	> 25/1.0	28
3	CuI	47	23/1.0	22
4	CuOAc	64	1.2/1.0	9
5	CuTC	51	> 25/1.0	22
6	CuCN	54	3.2/1.0	11
7	CuSCN	32	> 25/1.0	22

[a]Reaction conditions: **1a** (0.1 mmol), **2a** (0.3 mmol), $Cu(OAc)_2$ (0.7 equiv.), Cu(I) (0.3 equiv.), (±)-**L10** (50 mol%), Ag_2CO_3 (0.5 equiv.), NaOAc (1.0 equiv.), DMSO (5.0 mL), air, 70 °C, 12 h. The yield was determined by ^1H NMR using TTCE as internal standard.

Supplementary Table 8. Cu(I) salt screening when using racemic L10 as ligand.

2.2.9 Loading of Copper Salts[a]

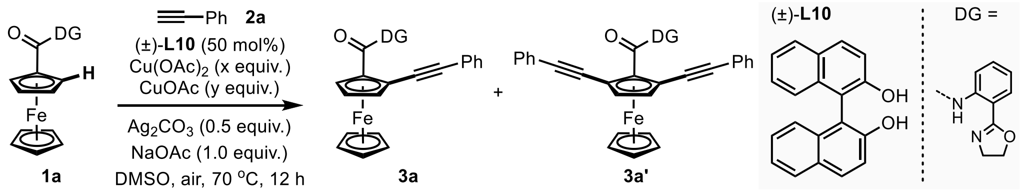

Entry	$Cu(OAc)_2$ (equiv.)	CuOAc (equiv.)	3a+3a' (%)	3a/3a'	1a (%)
1	0.7	0.3	64	1.2/1.0	9
2	0.7	0.4	66	3.4/1.0	14
3	-	1.5	66	16/1.0	1
4	-	2.0	63	> 25/1.0	1

[a]Reaction conditions: **1a** (0.1 mmol), **2a** (0.3 mmol), $Cu(OAc)_2$ (x equiv.), CuOAc (y equiv.), (±)-**L10** (50 mol%), Ag_2CO_3 (0.5 equiv.), NaOAc (1.0 equiv.), DMSO (5.0 mL), air, 70 °C, 12 h. The yield was determined by ^1H NMR using TTCE as internal standard.

Supplementary Table 9. Copper salt loading screen when using racemic L10 as ligand.

2.2.10 Loading of Ligand[a]

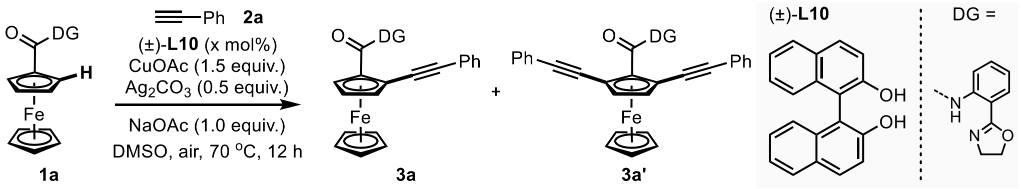

Entry	x (mol%)	3a+3a' (%)	3a/3a'	1a (%)
1	30	64	> 25/1.0	6
2	40	67	> 25/1.0	4
3	50	66	16/1.0	1

[a]Reaction conditions: **1a** (0.1 mmol), **2a** (0.3 mmol), CuOAc (1.5 equiv.), (±)-**L10** (x mol%), Ag_2CO_3 (0.5 equiv.), NaOAc (1.0 equiv.), DMSO (5.0 mL), air, 70 °C, 12 h. The yield was determined by ^1H NMR using TTCE as internal standard.

Supplementary Table 10. L10 ligand loading screen.

2.2.11 Loading of Phenylacetylene (2a)[a]

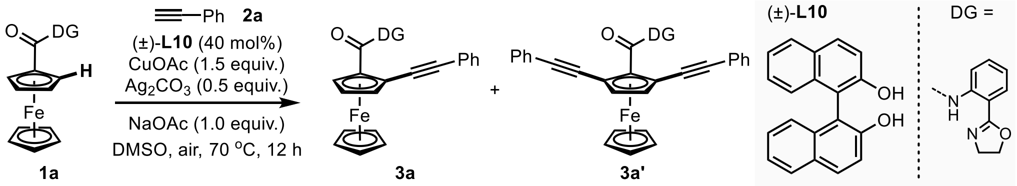

Entry	x (equiv.)	3a+3a' (%)	3a/3a'	1a (%)
1	2.0	63	> 25/1.0	-
2	2.5	68	> 25/1.0	3
3	3.0	67	> 25/1.0	4

[a]Reaction conditions: **1a** (0.1 mmol), **2a** (x equiv.), CuOAc (1.5 equiv.), (±)-**L10** (40 mol%), Ag_2CO_3 (0.5 equiv.), NaOAc (1.0 equiv.), DMSO (5.0 mL), air, 70 °C, 12 h. The yield was determined by ^1H NMR using TTCE as internal standard.

Supplementary Table 11. Screening the loading of the alkyne coupling partner.

2.2.12 Ligand Effect[a]

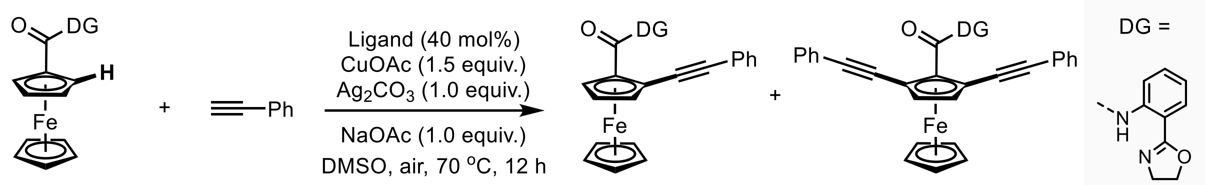

1a + **2a** → **3a** + **3a'**

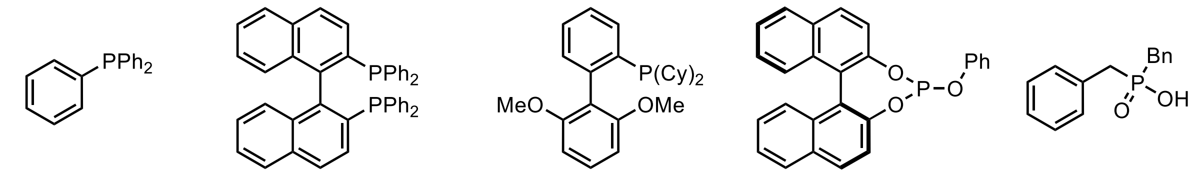

L1, **3a+3a'**: 49%; **3a/3a'** > 25/1.0; **1a**: 26%

(±)-**L2**, **3a+3a'**: 58%; **3a/3a'** > 25/1.0; **1a**: 15%

L30, **3a+3a'**: 61%; **3a/3a'** > 25/1.0; **1a**: 4%

(S)-L31, **3a+3a'**: 43%; **3a/3a'** > 25/1.0; **1a**: 26%

L32, **3a+3a'**: 38%; **3a/3a'** > 25/1.0; **1a**: 25%

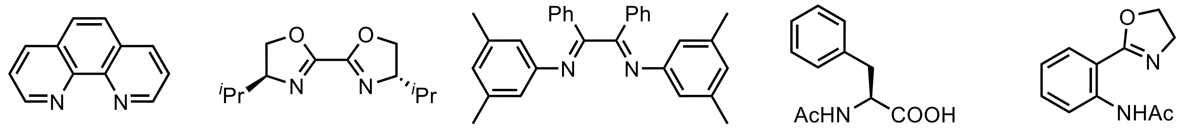

L3, **3a+3a'**: 57%; **3a/3a'** > 25/1.0; **1a**: 25%

(*S, S*)-**L4**, **3a+3a'**: 47%; **3a/3a'** > 25/1.0; **1a**: 31%

L33, **3a+3a'**: 42%; **3a/3a'** > 25/1.0; **1a**: 8%

L5, **3a+3a'**: 42%; **3a/3a'** > 25/1.0; **1a**: 25%

L34, **3a+3a'**: 63%; **3a/3a'** = 6.9/1.0; **1a**: 8%

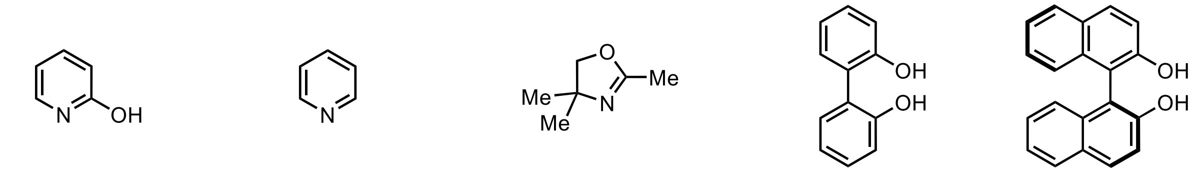

L6, **3a+3a'**: 52%; **3a/3a'** > 25/1.0; **1a**: 12%

L7, **3a+3a'**: 53%; **3a/3a'** = 17/1.0; **1a**: 8%

L8, **3a+3a'**: 52%; **3a/3a'** > 25/1.0; **1a**: 2%

L9, **3a+3a'**: 40%; **3a/3a'** > 25/1.0; **1a**: 35%

(*S*)-**L10**, **3a+3a'**: 69%; **3a/3a'** = 22/1.0; **1a**: 3%

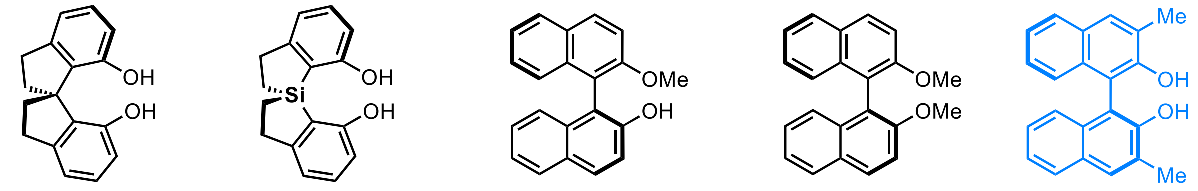

(*S*)-**L11**, **3a+3a'**: 65%; **3a/3a'** = 15/1.0; **1a**: 4%

(*S*)-**L12**, **3a+3a'**: 42%; **3a/3a'** > 25/1.0; **1a**: 34%

(*S*)-**L13**, **3a+3a'**: 62%; **3a/3a'** > 25/1.0; **1a**: 7%

(*S*)-**L14**, **3a+3a'**: 48%; **3a/3a'** > 25/1.0; **1a**: 31%

(*S*)-**L15**, **3a+3a'**: 76%; **3a/3a'** = 4.8/1; **1a**: -

[a]Reaction conditions: **1a** (0.1 mmol), **2a** (0.25 mmol), CuOAc (1.5 equiv.), Ligand (40 mol%), Ag_2CO_3 (0.5 equiv.), NaOAc (1.0 equiv.), DMSO (5.0 mL), air, 70 °C, 12 h. The yield was determined by ^1H NMR using TTCE as internal standard.

Supplementary Table 12. Additional ligand screening, including expanded evaluation of bisphenol ligands.

2.2.13 Control Experiments[a]

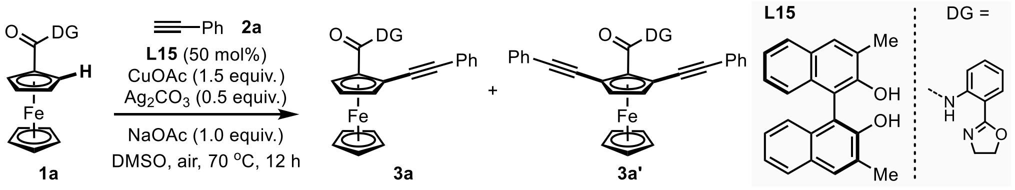

Entry	Conditions	3a+3a' (%)	3a/3a'	1a (%)
1	without CuOAc	-	-	97
2	without **L15**	56	> 25:1	23
3	without Ag_2CO_3	68	> 25:1	14
4	without NaOAc	9	> 25:1	81
5	none	76	4.8/1.0	-

[a]Reaction conditions: **1a** (0.1 mmol), **2a** (2.5 equiv.), CuOAc (1.5 equiv.), **L15** (40 mol%), Ag_2CO_3 (0.5 equiv.), NaOAc (1.0 equiv.), DMSO (5.0 mL), air, 70 °C, 12 h. The yield was determined by ^1H NMR using TTCE as internal standard.

Supplementary Table 13. Control experiments.

2.3 Experimental Optimizationfor Cu-Mediated Asymmetric C–H Alkynylation

2.3.1 Ligand Effect[a]

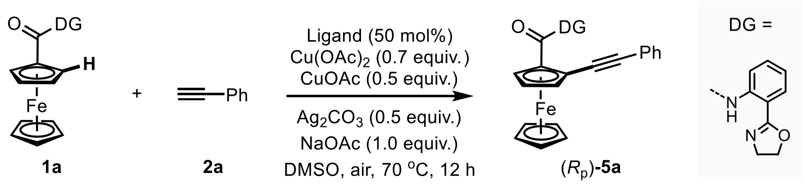

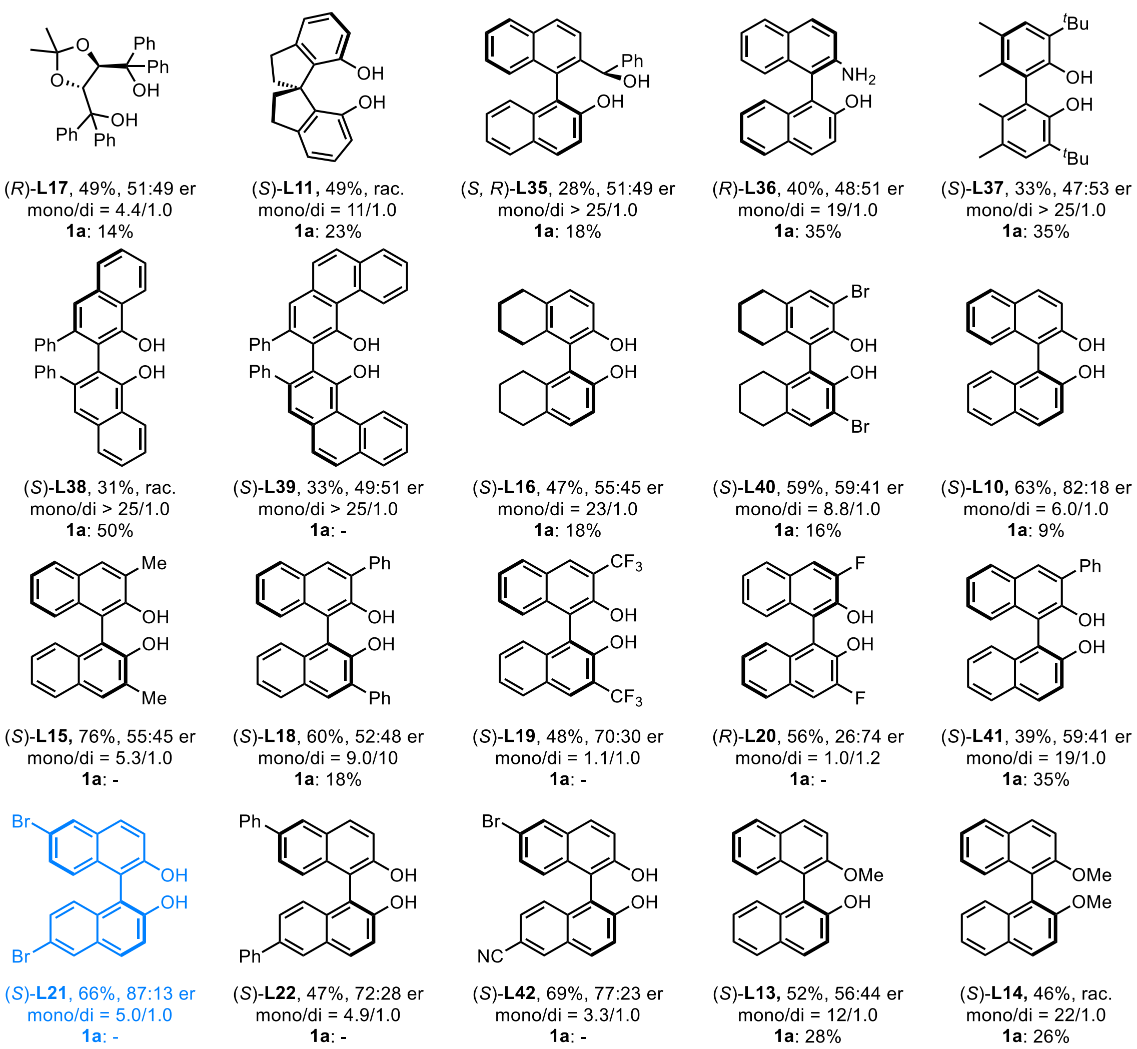

[a]Reaction conditions: **1a** (0.1 mmol), **2a** (0.3 mmol), $Cu(OAc)_2$ (0.7 equiv.), CuOAc (0.5 equiv.), Ag_2CO_3 (0.5 equiv.), Ligand (0.5 equiv.), NaOAc (1.0 equiv.), DMSO (5.0 mL), air, 70 °C, 12 h. The yield was determined by ^{1}H NMR using TTCE as internal standard. The er value for mono alkynylated product was determined by chiral HPLC.

Supplementary Table 14. Ligand screening for the asymmetric C–H alkynylation.

2.3.2 Loading of Phenylacetylene (2a)[a]

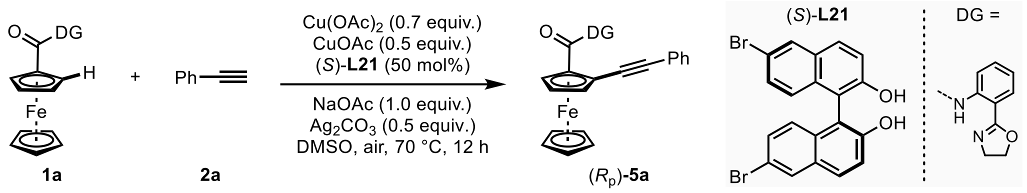

Entry	x (equiv.)	Yield (%)	mono/di	er	1a (%)
1	2.0	63	4.7/1.0	86:14	-
2	2.5	69	3.6/1.0	87:13	-
3	3.0	66	5.0/1.0	87:13	-
4	4.0	62	7.8/1.0	85:15	9

[a]Reaction conditions: **1a** (0.1 mmol), **2a** (x equiv.), $Cu(OAc)_2$ (0.7 equiv.), CuOAc (0.5 equiv.), (*S*)-**L21** (50 mol%), Ag_2CO_3 (0.5 equiv.), NaOAc (1.0 equiv.), DMSO (5.0 mL), air, 70 °C, 12 h. The yield was determined by ^{1}H NMR using TTCE as internal standard. The er value for mono alkynylated product was determined by chiral HPLC.

Supplementary Table 15. Loading screen of the alkyne coupling partner for the asymmetric transformation with (*S*)-**L21**.

2.3.3 Temperature

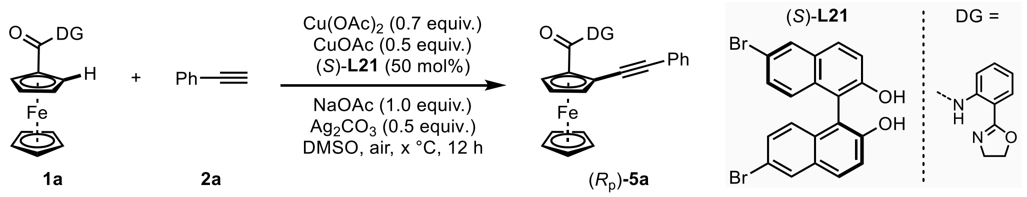

Entry	x (°C)	Yield (%)	mono/di	e.r.	1a (%)
1	70	69	3.6/1.0	87:13	-
2	60	60	2.8/1.0	89:11	-
3	50	54	6.7/1.0	90:10	8

[a]Reaction conditions: **1a** (0.1 mmol), **2a** (2.5 equiv.), $Cu(OAc)_2$ (0.7 equiv.), CuOAc (0.5 equiv.), (*S*)-**L21** (50 mol%), Ag_2CO_3 (0.5 equiv.), NaOAc (1.0 equiv.), DMSO (5.0 mL), air, 70 °C, 12 h. The yield was determined by ^{1}H NMR using TTCE as internal standard. The er value for mono alkynylated product was determined by chiral HPLC.

Supplementary Table 16. Temperature screen for the asymmetric transformation with (*S*)-**L21**.

2.3.4 Loading of $Cu(OAc)_2$ and Ligand[a]

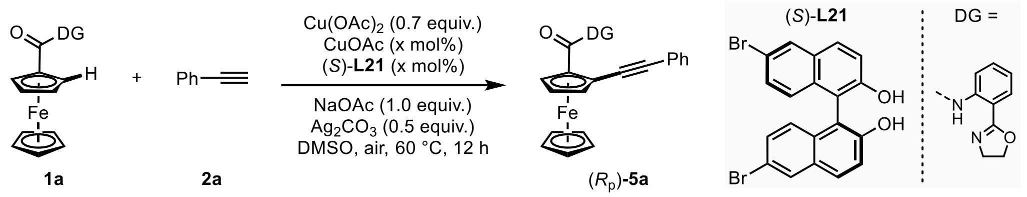

Entry	x (mol%)	Yield (%)	mono/di	e.r.	1a (%)
1	50	60	2.8/1.0	89:11	-
2	40	65	2.1/1.0	90:10	-
3	30	64	1.6/1.0	92.5:7.5	2
4	20	62	2.6/1.0	90:10	9

[a]Reaction conditions: **1a** (0.1 mmol), **2a** (2.5 equiv.), $Cu(OAc)_2$ (0.7 equiv.), CuOAc (x mol%), (*S*)-**L21** (x mol%), Ag_2CO_3 (0.5 equiv.), NaOAc (1.0 equiv.), DMSO (5.0 mL), air, 70 °C, 12 h. The yield was determined by ^{1}H NMR using TTCE as internal standard. The er value for mono alkynylated product was determined by chiral HPLC.

Supplementary Table 17. Cu(II) acetate loading and ligand loading screen for the asymmetric transformation with (*S*)-**L21**.

2.4 Evaluation of Cu-Mediated C–H Alkynylation of Ferrocenes

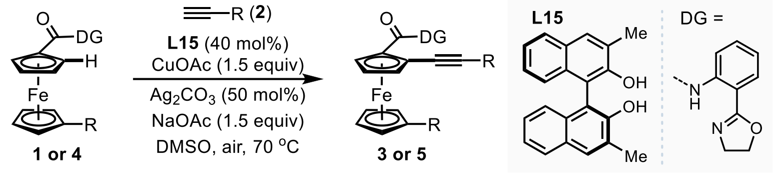

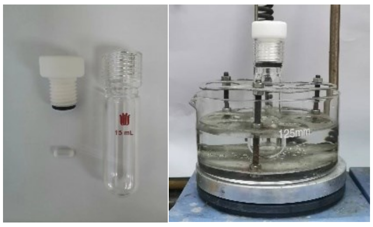

General Procedure for Cu-Mediated C–H Alkynylation of Ferrocenes: A 15 mL scale tube was charged with substrate **1a** (37.4 mg, 0.1 mmol, 1.0 equiv.), CuOAc (18.4 mg, 0.15 mmol), **L15** (12.6 mg, 40 mol%), Ag_2CO_3 (13.8 mg, 0.05 mmol), NaOAc (8.2 mg, 0.1 mmol), **2** (0.25 mmol, 2.5 equiv.), DMSO (5.0 mL) under air atmosphere. The tube was capped tightly, and the mixture was stirred at room temperature for 30 s then stirred at 70 °C for another 12 hours. Upon completion, the reaction was cooled to room temperature, then EtOAc was added to dilute the reaction mixture. The organic layer was washed with $NH_3{\cdot}H_2O$, saturated brine, and dried over Na_2SO_4. Volatiles were removed under vacuum. The crude product was purified by column chromatography to afford the desired product **3** or **5**. The ratio of mono/di was determined by the analysis of crude ^{1}H NMR (**3a-r**), and the ratio of mono/di was determined by the analysis of isolated yield (**5a-i**).

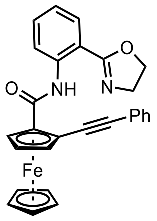

3a (mono)

***N*-(2-(4,5-Dihydrooxazol-2-yl)phenyl)-2-(phenylethynyl)ferrocenyl-1-carboxamide**

3a (mono) and **3a’** (di) were synthesized following the general procedure (**3a/3a’** = 4.8/1.0). After purification by column chromatography on silica gel (PE/EA/DCM = 30/1/1), **3a** (mono) was obtained in 69% yield (32.5 mg). **3a’** (di) was obtained in 13% yield (7.7 mg).

Orange solid, R_f = 0.5 (PE/EA/DCM = 30/2/1), ^{1}H NMR (400 MHz, $CDCl_3$) δ 12.48 (s, 1H), 8.88 (d, *J* = 8.4 Hz, 1H), 7.89 (d, *J* = 7.6 Hz, 1H), 7.50 (t, *J* = 7.8 Hz, 1H), 7.44–7.37 (m, 2H), 7.31–7.23 (m, 3H), 7.10 (t, *J* = 7.6, 1H), 4.98 (s, 1H), 4.73 (s, 1H), 4.48 (t, *J* = 2.6 Hz, 1H), 4.36 (s, 5H), 4.25–4.17 (m, 1H), 4.11–3.99 (m, 2H), 3.99–3.88 (m, 1H); ^{13}C NMR (100 MHz, $CDCl_3$) δ 168.9, 164.2, 140.1, 132.4, 131.2, 129.2, 128.2, 127.7, 123.8, 122.0, 120.3, 113.3, 89.4, 87.1, 78.9, 74.4, 71.8, 70.2, 70.1, 66.1, 66.0, 54.8; HRMS (ESI-TOF) *m/z* calculated for $C_{28}H_{23}N_2O_2Fe$ $[M+H]^+$: 475.1103, found: 475.1109.

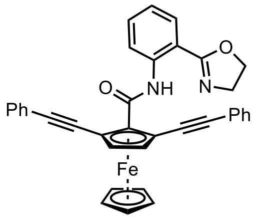

3a' (di)

***N*-(2-(4,5-Dihydrooxazol-2-yl)phenyl)-2,5-bis(phenylethynyl)ferrocenyl-1-carboxamide**

Orange solid, R_f = 0.7 (PE/EA/DCM = 30/2/1), ^{1}H NMR (400 MHz, $CDCl_3$) δ 12.68 (s, 1H), 9.03 (d, *J* = 8.4 Hz, 1H), 7.90 (d, *J* = 7.6 Hz, 1H), 7.52 (t, *J* = 8.0 Hz, 1H), 7.44–7.34 (m, 4H), 7.34–7.19 (m, 6H), 7.13 (t, *J* = 8.0 Hz, 1H), 4.78 (s, 2H), 4.48 (s, 5H), 3.98–3.81 (m, 4H); ^{13}C NMR (100 MHz, $CDCl_3$) δ 167.1, 163.7, 140.0, 132.3, 131.2, 129.2, 128.1, 127.8, 123.5, 122.1, 120.4, 113.4, 89.5, 86.3, 81.8, 73.6, 73.3, 67.0, 65.8, 54.7; HRMS (ESI-TOF) *m/z* calculated for $C_{36}H_{27}N_2O_2Fe$ $[M+H]^+$: 575.1416, found: 575.1420.

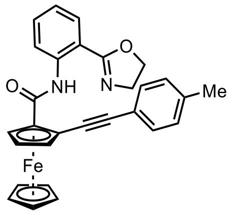

3b (mono)

***N*-(2-(4,5-dihydrooxazol-2-yl)phenyl)-2-(p-tolylethynyl)ferrocenyl-1-carboxamide**

3b (mono) and **3b'** (di) were synthesized following the general procedure (**3b/3b'** = 2.7/1.0). After purification by column chromatography on silica gel (PE/EA/DCM = 30/1/1), **3b** (mono) was obtained in 41% yield (20.2 mg). **3b'** (di) was obtained in 15% yield (9.0 mg).

Orange solid, R_f = 0.5 (PE/EA/DCM = 30/2/1), ^{1}H NMR (400 MHz, $CDCl_3$) δ 12.47 (s, 1H), 8.87 (d, *J* = 8.6 Hz, 1H), 7.90 (d, *J* = 8.0 Hz, 1H), 7.51 (t, *J* = 8.0 Hz, 1H), 7.30 (d, *J* = 7.8 Hz, 2H), 7.14–7.04 (m, 3H), 4.98 (s, 1H), 4.72 (s, 1H), 4.47 (s, 1H), 4.36 (s, 5H), 4.28–4.18 (m, 1H), 4.12–3.87 (m, 3H), 2.33 (s, 3H); ^{13}C NMR (100 MHz, $CDCl_3$) δ 169.0, 164.1, 140.1, 137.8, 132.4, 131.1, 129.2, 129.0, 122.0, 120.8, 120.4, 113.4, 89.6, 86.3, 78.8, 74.4, 71.8, 70.2, 70.0, 66.4, 66.0, 54.8, 21.5; HRMS (ESI-TOF) *m/z* calculated for $C_{29}H_{25}N_2O_2Fe$ $[M+H]^+$: 489.1260, found: 489.1258.

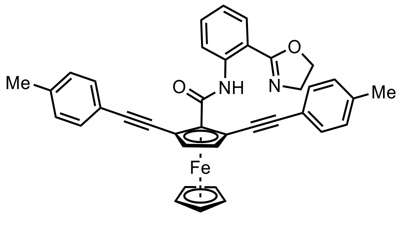

3b' (di)

***N*-(2-(4,5-dihydrooxazol-2-yl)phenyl)-2,5-bis(p-tolylethynyl)ferrocenyl-1-carboxamide**

Orange solid, R_f = 0.7 (PE/EA/DCM = 30/2/1), ^{1}H NMR (400 MHz, $CDCl_3$) δ 12.66 (s, 1H), 9.02 (d, J = 8.4 Hz, 1H), 7.90 (d, J = 7.8 Hz, 1H), 7.52 (t, J = 8.0 Hz, 1H), 7.30 (d, J = 7.6 Hz, 4H), 7.13 (d, J = 7.6 Hz, 1H), 7.08 (d, J = 8.0 Hz, 4H), 4.76 (s, 2H), 4.48 (s, 5H), 4.02–3.84 (m, 4H), 2.33 (s, 6H); ^{13}C NMR (100 MHz, $CDCl_3$) δ 167.3, 163.8, 140.1, 137.9, 132.4, 131.2, 129.2, 128.9, 122.1, 120.6, 113.5, 89.7, 85.6, 81.6, 73.6, 73.3, 73.3, 67.3, 65.9, 54.8, 21.5; HRMS (ESI-TOF) *m/z* calculated for $C_{38}H_{31}N_2O_2Fe$ $[M+H]^+$: 603.1729, found: 603.1729.

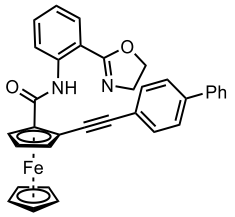

3c (mono)

2-([1,1'-Biphenyl]-4-ylethynyl)-*N*-(2-(4,5-dihydrooxazol-2-yl)phenyl)ferrocenyl-1-carboxamide

3c (mono) and **3c'** (di) were synthesized following the general procedure (**3c/3c'** = 7.7/1.0). After purification by column chromatography on silica gel (PE/EA/DCM = 30/1/1), **3c** (mono) was obtained in 46% yield (25.5 mg). **3c'** (di) was obtained in 6% yield (4.7 mg).

Orange solid, R_f = 0.5 (PE/EA/DCM = 30/2/1), ^{1}H NMR (400 MHz, $CDCl_3$) δ 12.53 (s, 1H), 8.90 (d, J = 8.4 Hz, 1H), 7.92 (d, J = 7.6 Hz, 1H), 7.70–7.30 (m, 10H), 7.11 (t, J = 7.2 Hz, 1H), 5.01 (s, 1H), 4.76 (s, 1H), 4.50 (s, 1H), 4.38 (s, 5H), 4.31–4.15 (m, 1H), 4.15–3.88 (m, 3H); ^{13}C NMR (100 MHz, $CDCl_3$) δ 168.9, 164.2, 140.3, 140.3, 140.1, 132.5, 131.6, 129.3, 128.8, 127.6, 126.9, 126.8, 122.8, 122.0, 120.4, 113.3, 89.3, 88.0, 79.0, 74.5, 71.8, 70.3, 70.2, 66.1, 66.0, 54.8; HRMS (ESI-TOF) *m/z* calculated for $C_{34}H_{27}N_2O_2Fe$ $[M+H]^+$: 551.1416, found: 551.1410.

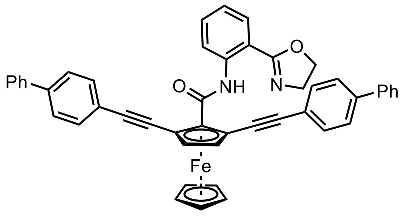

3c' (di)

2,5-Bis([1,1'-biphenyl]-4-ylethynyl)-*N*-(2-(4,5-dihydrooxazol-2-yl)phenyl)ferrocenyl-1-carboxamide

Orange solid, R_f = 0.7 (PE/EA/DCM = 30/2/1), ^{1}H NMR (400 MHz, $CDCl_3$) δ 12.73 (s, 1H), 9.07 (d, J = 8.4 Hz, 1H), 7.92 (d, J = 8.0 Hz, 1H), 7.60–7.39 (m, 17H), 7.33 (t, J = 7.4 Hz, 2H), 7.13 (t, J = 7.6 Hz, 1H), 4.80 (s, 2H), 4.50 (s, 5H), 4.00–3.83 (m, 4H); ^{13}C NMR (100 MHz, $CDCl_3$) δ 167.2, 163.8, 140.4, 140.2, 140.1, 132.5, 131.7, 129.3, 128.8, 127.5, 126.9, 126.8, 122.5, 122.2, 120.5, 113.4, 89.5, 87.2, 81.8, 73.7, 73.4, 67.1, 65.9, 54.8; HRMS (ESI-TOF) *m/z* calculated for $C_{48}H_{35}N_2O_2Fe$ $[M+H]^+$: 727.2042, found: 727.2046.

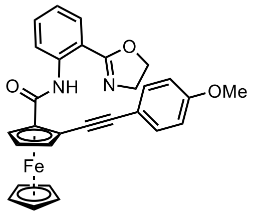

3d (mono)

N-(2-(4,5-Dihydrooxazol-2-yl)phenyl)-2-((4-methoxyphenyl)ethynyl)ferrocenyl-1-carboxamide

3d (mono) and **3d'** (di) were synthesized following the general procedure (**3d/3d'** = 4.6/1.0). After purification by column chromatography on silica gel (PE/EA/DCM = 30/1/1), **3d** (mono) was obtained in 51% yield (25.7 mg). **3d'** (di) was obtained in 13% yield (8.4 mg).

Orange solid, R_f = 0.4 (PE/EA/DCM = 25/2/1), ^{1}H NMR (400 MHz, $CDCl_3$) δ 12.48 (s, 1H), 8.88 (d, J = 8.4 Hz, 1H), 7.91 (d, J = 8.0, 1H), 7.51 (t, J = 8.2 Hz, 1H), 7.35 (d, J = 6.8 Hz, 2H), 7.12 (d, J = 7.2 Hz, 1H), 6.81 (d, J = 6.8 Hz, 2H), 4.97 (s, 1H), 4.72 (s, 1H), 4.47 (s, 1H), 4.36 (s, 5H), 4.31–4.19 (m, 1H), 4.16–3.92 (m, 3H), 3.81 (s, 3H); ^{13}C NMR (100 MHz, $CDCl_3$) δ 168.9, 164.2, 140.1, 132.4, 129.7, 129.3, 127.8, 125.1, 122.9, 122.0, 120.3, 113.3, 86.4, 84.5, 78.8, 74.4, 71.8, 70.2, 70.0, 66.2, 66.1, 54.8; HRMS (ESI-TOF) *m/z* calculated for $C_{29}H_{25}N_2O_3Fe$ $[M+H]^+$: 505.1209, found: 505.1206.

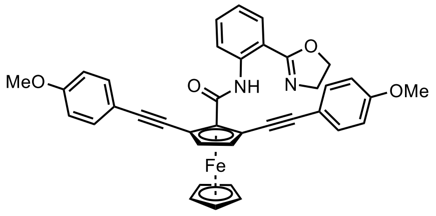

3d' (di)

N-(2-(4,5-Dihydrooxazol-2-yl)phenyl)-2,5-bis((4-methoxyphenyl)ethynyl)ferrocenyl-1-carboxamide

Orange solid, R_f = 0.7 (PE/EA/DCM = 25/2/1), ^{1}H NMR (400 MHz, $CDCl_3$) δ 12.67 (s, 1H), 9.01 (d, J = 8.4 Hz, 1H), 7.91 (d, J = 8.0 Hz, 1H), 7.53 (t, J = 8.0 Hz, 1H), 7.35 (d, J = 7.0 Hz, 4H), 7.13 (t, J = 7.6 Hz, 1H), 6.81 (d, J = 6.8 Hz, 4H), 4.75 (s, 2H), 4.48 (s, 5H), 4.08–3.89 (m, 4H), 3.81 (s, 6H); ^{13}C NMR (125 MHz, $CDCl_3$) δ 167.4, 163.8, 159.3, 140.1, 132.8, 132.4, 129.2, 122.1, 120.6, 115.8, 113.8, 113.4, 89.4, 84.8, 81.3, 73.6, 73.1, 67.4, 65.9, 55.3, 54.9; HRMS (ESI-TOF) *m/z* calculated for $C_{38}H_{31}N_2O_4Fe$ $[M+H]^+$: 635.1628, found: 635.1631.

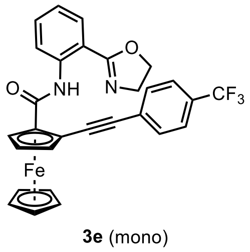

3e (mono)

N-(2-(4,5-Dihydrooxazol-2-yl)phenyl)-2-((4-(trifluoromethyl)phenyl)ethynyl)ferrocenyl-1-carboxamide

3e (mono) and **3e'** (di) were synthesized following the general procedure (**3e/3e'** = 6.4/1.0). After purification by column chromatography on silica gel (PE/EA/DCM = 30/1/1), **3e** (mono) was obtained in 63% yield (34.2 mg). **3e'** (di) was obtained in 12% yield (8.6 mg).

Orange solid, R_f = 0.5 (PE/EA/DCM = 30/2/1), ^{1}H NMR (400 MHz, $CDCl_3$) δ 12.48 (s, 1H), 8.88 (d, J = 8.8 Hz, 1H), 7.91 (d, J = 8.0 Hz, 1H), 7.58–7.46 (m, 5H), 7.11 (t, J = 7.6 Hz, 1H), 5.01 (s, 1H), 4.75 (s, 1H), 4.51 (s, 1H), 4.36 (s, 5H), 4.33–3.92 (m, 4H); ^{13}C NMR (100 MHz, $CDCl_3$) δ 168.7, 164.5, 140.1, 132.6, 131.4, 129.3, 129.2 (q, J = 32.5 Hz), 127.7, 125.1 (q, J = 3.8 Hz), 124.0 (q, J = 270.4 Hz), 122.1, 120.2, 113.1, 90.2, 87.9, 79.1, 74.7, 71.9, 70.6, 70.3, 66.0, 65.3, 54.8; ^{19}F NMR (375 MHz, $CDCl_3$) δ -62.67 (s, 3F); HRMS (ESI-TOF) *m/z* calculated for $C_{29}H_{22}N_2O_2F_3Fe$ $[M+H]^+$: 543.0977, found: 543.0972.

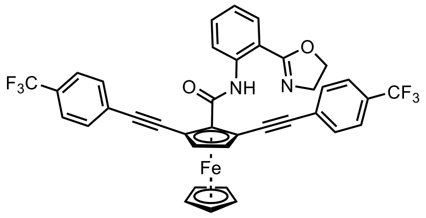

3e' (di)

N-(2-(4,5-Dihydrooxazol-2-yl)phenyl)-2,5-bis((4-(trifluoromethyl)phenyl)ethynyl)ferrocenyl-1-carboxamide

Orange solid, R_f = 0.7 (PE/EA/DCM = 30/2/1), ^{1}H NMR (400 MHz, $CDCl_3$) δ 12.69 (s, 1H), 9.00 (d, J = 8.4 Hz, 1H), 7.93 (d, J = 8.1 Hz, 1H), 7.58–7.47 (m, 9H), 7.20–7.11 (m, 1H), 4.84 (s, 2H), 4.51 (s, 5H), 4.05–3.96 (m, 2H), 3.92–3.82 (m, 2H); ^{13}C NMR (100 MHz, $CDCl_3$) δ 166.8, 164.1, 140.0, 132.6, 131.5, 129.5 (q, J = 32.0 Hz), 129.4, 127.3, 125.1 (q, J = 3.8 Hz), 123.9 (q, J = 271.0 Hz), 122.5, 120.5, 113.3, 89.1, 88.4, 82.4, 73.9, 73.8, 66.5, 65.9, 54.7; ^{19}F NMR (375 MHz, $CDCl_3$) δ -62.72 (s, 3F); HRMS (ESI-TOF) *m/z* calculated for $C_{38}H_{25}N_2O_2F_6Fe$ $[M+H]^+$: 711.1164, found: 711.1163.

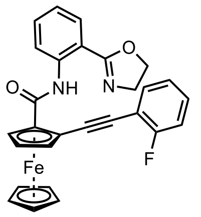

3f (mono)

***N*-(2-(4,5-Dihydrooxazol-2-yl)phenyl)-2-((2-fluorophenyl)ethynyl)ferrocenyl-1-carboxamide**

3f (mono) and **3f'** (di) were synthesized following the general procedure (16 h, **3f/3f'** = 3.6/1.0). After purification by column chromatography on silica gel (PE/EA/DCM = 30/1/1), **3f** (mono) was obtained in 62% yield (30.5 mg). **3f'** (di) was obtained in 16% yield (9.8 mg).

Orange solid, R_f = 0.5 (PE/EA/DCM = 25/2/1), ^{1}H NMR (400 MHz, $CDCl_3$) δ 12.47 (s, 1H), 8.88 (d, J = 8.6 Hz, 1H), 7.89 (d, J = 7.8 Hz, 1H), 7.50 (t, J = 8.0 Hz, 1H), 7.43 (t, J = 7.6 Hz, 1H), 7.31–7.23 (m, 1H), 7.14–7.00 (m, 3H), 5.01 (s, 1H), 4.78 (s, 1H), 4.51 (t, J = 2.6 Hz, 1H), 4.37 (s, 5H), 4.32–4.20 (m, 1H), 4.19–3.95 (m, 3H); ^{13}C NMR

(100 MHz, $CDCl_3$) δ 168.7, 164.4, 162.4 (d, *J* = 251.3 Hz), 140.1, 133.3 (d, *J* = 1.4 Hz), 132.4, 129.4 (d, *J* = 7.8 Hz), 129.2, 123.9 (d, *J* = 3.7 Hz), 122.0, 120.3, 115.4 (d, *J* = 20.9 Hz), 113.3, 112.6 (d, *J* = 15.6 Hz), 92.3 (d, *J* = 3.2 Hz), 82.4, 79.0, 74.8, 71.9, 70.4, 70.2, 66.0, 65.7, 54.8; ^{19}F NMR (375 MHz, $CDCl_3$) δ -109.88–-110.02 (m, 1F); HRMS (ESI-TOF) *m/z* calculated for $C_{28}H_{22}N_2O_2FFe$ $[M+H]^+$: 493.1009, found: 493.1009.

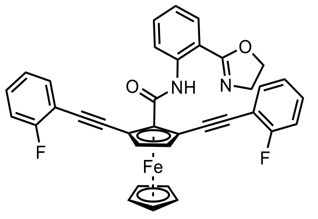

3f' (di)

***N*-(2-(4,5-Dihydrooxazol-2-yl)phenyl)-2,5-bis((2-fluorophenyl)ethynyl)ferrocenyl-1-carboxamide**

Orange solid, R_f = 0.7 (PE/EA/DCM = 25/2/1), ^{1}H NMR (400 MHz, $CDCl_3$) δ 12.61 (s, 1H), 8.97 (d, *J* = 8.4 Hz, 1H), 7.88 (d, *J* = 7.8 Hz, 1H), 7.50 (t, *J* = 8.0 Hz, 1H), 7.38 (t, *J* = 7.4 Hz, 2H), 7.31–7.21 (m, 2H), 7.11 (t, *J* = 7.6 Hz, 1H), 7.04 (t, *J* = 8.4 Hz, 4H), 4.83 (s, 2H), 4.52 (s, 5H), 4.07–3.86 (m, 4H); ^{13}C NMR (100 MHz, $CDCl_3$) δ 166.9, 163.9, 162.4 (d, *J* = 251.6 Hz), 140.0, 133.4 (d, *J* = 1.3 Hz), 132.4, 129.6 (d, *J* = 7.8 Hz), 129.2, 123.9 (d, *J* = 3.7 Hz), 122.2, 120.6, 115.4 (d, *J* = 20.9 Hz), 113.6, 112.3 (d, *J* = 15.7 Hz), 91.4 (d, *J* = 3.1 Hz), 82.8, 82.2, 73.9, 73.8, 66.7, 65.9, 54.8; ^{19}F NMR (375 MHz, $CDCl_3$) δ -109.80–-109.91 (m, 2F); HRMS (ESI-TOF) *m/z* calculated for $C_{36}H_{25}N_2O_2F_2Fe$ $[M+H]^+$: 611.1228, found: 611.1220.

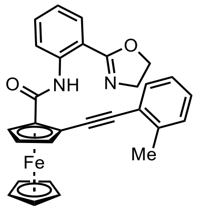

3g (mono)

***N*-(2-(4,5-dihydrooxazol-2-yl)phenyl)-2-(o-tolylethynyl)ferrocenyl-1-carboxamide**

3g (mono) and **3g'** (di) were synthesized following the general procedure (**3g/3g'** = 2.6/1.0). After purification by column chromatography on silica gel (PE/EA/DCM = 30/1/1), **3g** (mono) was obtained in 53% yield (25.8 mg). **3g'** (di) was obtained in 19% yield (11.5 mg).

Orange solid, R_f = 0.5 (PE/EA/DCM = 25/2/1), ^{1}H NMR (400 MHz, $CDCl_3$) δ 12.46 (s, 1H), 8.88 (d, *J* = 8.6 Hz, 1H), 7.88 (d, *J* = 8.0, 1H), 7.50 (t, *J* = 7.8 Hz, 1H), 7.40 (d, *J* = 7.6 Hz, 1H), 7.24–7.15 (m, 2H), 7.15–7.04 (m, 2H), 4.99 (s, 1H), 4.74 (s, 1H), 4.49 (d, *J* = 2.4 Hz, 1H), 4.36 (s, 5H), 4.28–4.16 (m, 1H), 4.13–3.86 (m, 3H), 2.44 (s, 3H); ^{13}C NMR (100 MHz, $CDCl_3$) δ 168.9, 164.2, 140.1, 137.9, 132.4, 131.8, 129.2, 128.6, 128.1, 128.1, 123.6, 122.0, 120.3, 113.3, 89.6, 86.7, 79.0, 74.3, 71.8, 70.2, 70.1, 66.3, 66.0, 54.8, 21.1; HRMS (ESI-TOF) *m/z* calculated for $C_{29}H_{25}N_2O_2Fe$ $[M+H]^+$: 489.1260, found: 489.1258.

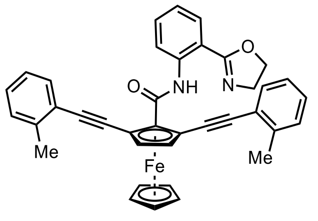

3g' (di)

N-(2-(4,5-Dihydrooxazol-2-yl)phenyl)-2,5-bis(o-tolylethynyl)ferrocenyl-1-carboxamide

Orange solid, R_f = 0.7 (PE/EA/DCM = 25/2/1), ^{1}H NMR (400 MHz, $CDCl_3$) δ 12.59 (s, 1H), 9.00 (d, J = 8.6 Hz, 1H), 7.85 (d, J = 8.0 Hz, 1H), 7.50 (t, J = 8.0 Hz, 1H), 7.37 (d, J = 7.6 Hz, 2H), 7.21–7.13 (m, 4H), 7.12–7.05 (m, 3H), 4.78 (s, 2H), 4.48 (s, 5H), 3.94–3.80 (m, 4H), 2.40 (s, 6H); ^{13}C NMR (100 MHz, $CDCl_3$) δ 167.0, 163.7, 140.1, 140.0, 132.4, 131.6, 129.3, 129.1, 127.9, 125.4, 123.4, 122.1, 120.5, 113.6, 90.0, 88.5, 82.2, 73.7, 73.3, 67.3, 65.9, 54.8, 20.6 ; HRMS (ESI-TOF) m/z calculated for $C_{38}H_{31}N_2O_2Fe$ $[M+H]^+$: 603.1729, found: 603.1734.

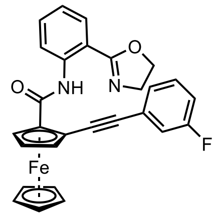

3h (mono)

N-(2-(4,5-Dihydrooxazol-2-yl)phenyl)-2-((3-fluorophenyl)ethynyl)ferrocenyl-1-carboxamide

3h (mono) and **3h'** (di) were synthesized following the general procedure (**3h/3h'** = 5.8/1.0). After purification by column chromatography on silica gel (PE/EA/DCM = 30/1/1), **3h** (mono) was obtained in 66% yield (32.5 mg). **3h'** (di) was obtained in 5% yield (2.9 mg).

Orange solid, R_f = 0.5 (PE/EA/DCM = 25/2/1), ^{1}H NMR (400 MHz, $CDCl_3$) δ 12.49 (s, 1H), 8.88 (d, J = 8.4 Hz, 1H), 7.91 (d, J = 7.8 Hz, 1H), 7.51 (t, J = 7.0 Hz, 1H), 7.32–7.18 (m, 2H), 7.18–7.05 (m, 2H), 6.99 (t, J = 8.0 Hz, 1H), 5.00 (s, 1H), 4.74 (s, 1H), 4.50 (s, 1H), 4.44–4.20 (m, 6H), 4.22–3.92 (m, 3H); ^{13}C NMR (100 MHz, $CDCl_3$) δ 168.7, 164.4, 162.3 (d, J = 246.2 Hz), 140.1, 132.5, 129.7 (d, J = 8.8 Hz), 129.3, 127.1 (d, J = 3.0 Hz), 125.8 (d, J = 9.6 Hz), 122.1, 120.3, 117.9 (d, J = 22.7 Hz), 115.0 (d, J = 21.2 Hz), 113.2, 88.4, 88.0 (d, J = 3.5 Hz), 79.0, 74.6, 71.8, 70.4, 70.2, 66.0, 65.6, 54.8; ^{19}F NMR (375 MHz, $CDCl_3$) δ -113.06–-113.22 (m, 1F); HRMS (ESI-TOF) m/z calculated for $C_{28}H_{22}N_2O_2FFe$ $[M+H]^+$: 493.1009, found: 493.1014.

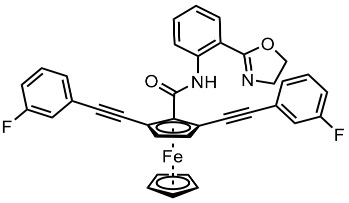

3h' (di)

N-(2-(4,5-dihydrooxazol-2-yl)phenyl)-2,5-bis((3-fluorophenyl)ethynyl)ferrocenyl-1-carboxamide

Orange solid, R_f = 0.7 (PE/EA/DCM = 25/2/1), ^{1}H NMR (400 MHz, $CDCl_3$) δ 12.61 (s, 1H), 8.97 (d, J = 8.4 Hz, 1H), 7.88 (d, J = 7.8 Hz, 1H), 7.50 (t, J = 8.0 Hz, 1H), 7.38 (t, J = 7.6 Hz, 2H), 7.31–7.21 (m, 2H), 7.11 (t, J = 7.6 Hz, 1H), 7.04 (t, J = 8.4 Hz, 4H), 4.83 (s, 2H), 4.52 (s, 5H), 4.06–3.83 (m, 4H); ^{13}C NMR (100 MHz, $CDCl_3$) δ 166.9, 164.0, 162.3 (d, J = 246.3 Hz), 140.0, 132.6, 129.8 (d, J = 8.7 Hz), 129.4, 127.2 (d, J = 3.0 Hz), 125.4 (d, J = 9.5 Hz), 122.4, 120.6, 118.0 (d, J = 22.8 Hz), 115.2 (d, J = 21.2 Hz), 113.4, 88.4 (d, J = 3.5 Hz), 87.4, 82.2, 73.8, 73.6, 66.7, 65.9, 54.8; ^{19}F NMR (375 MHz, $CDCl_3$) δ -113.03–-113.20 (m, 2F); HRMS (ESI-TOF) *m/z* calculated for $C_{36}H_{25}N_2O_2F_2Fe$ $[M+H]^+$: 611.1228, found: 611.1237.

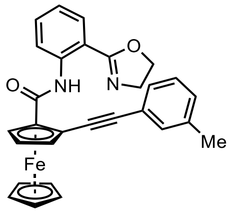

3i (mono)

***N*-(2-(4,5-Dihydrooxazol-2-yl)phenyl)-2-(m-tolylethynyl)ferrocenyl-1-carboxamide**

3i (mono) and **3i'** (di) were synthesized following the general procedure (**3i/3i'** = 5.0/1.0). After purification by column chromatography on silica gel (PE/EA/DCM = 30/1/1), **3i** (mono) was obtained in 69% yield (33.7 mg). **3i'** (di) was obtained in 14% yield (8.7 mg).

Orange solid, R_f = 0.5 (PE/EA/DCM = 30/2/1), ^{1}H NMR (400 MHz, $CDCl_3$) δ 12.51 (s, 1H), 8.90 (d, J = 8.4 Hz, 1H), 7.91 (d, J = 8.0 Hz, 1H), 7.51 (t, J = 7.8 Hz, 1H), 7.25–7.06 (m, 5H), 4.98 (s, 1H), 4.72 (s, 1H), 4.49 (d, J = 2.4 Hz, 1H), 4.37 (s, 5H), 4.27–4.17 (m, 1H), 4.11–3.87 (m, 3H), 2.28 (s, 3H); ^{13}C NMR (100 MHz, $CDCl_3$) δ 169.0, 164.2, 140.1, 137.9, 132.4, 131.9, 129.2, 128.6, 128.1, 128.1, 123.6, 122.0, 120.3, 113.4, 89.6, 86.7, 79.0, 74.4, 71.8, 70.2, 70.1, 66.3, 66.0, 54.8, 21.1; HRMS (ESI-TOF) *m/z* calculated for $C_{29}H_{25}N_2O_2Fe$ $[M+H]^+$: 489.1260, found: 489.1266.

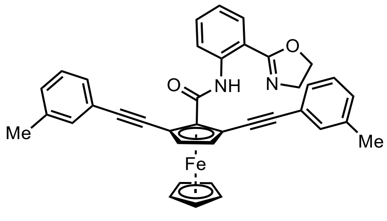

3i' (di)

***N*-(2-(4,5-Dihydrooxazol-2-yl)phenyl)-2,5-bis(m-tolylethynyl)ferrocenyl-1-carboxamide**

Orange solid, R_f = 0.7 (PE/EA/DCM = 30/2/1), ^{1}H NMR (400 MHz, $CDCl_3$) δ 12.68 (s, 1H), 9.05 (d, J = 8.6 Hz, 1H), 7.92 (d, J = 8.0 Hz, 1H), 7.54 (t, J = 8.4 Hz, 1H), 7.24–7.06 (m, 9H), 4.77 (s, 2H), 4.49 (s, 5H), 3.99–3.80 (m, 4H), 2.29 (s, 6H); ^{13}C NMR (100 MHz, $CDCl_3$) δ 167.2, 163.9, 140.2, 137.9, 132.5, 132.0, 129.2, 128.7, 128.3, 128.1, 123.4, 122.1, 120.5, 113.5, 89.8, 86.0, 81.9, 73.7, 73.3, 67.2, 65.9, 54.9, 21.1; HRMS (ESI-TOF) *m/z* calculated for $C_{38}H_{31}N_2O_2Fe$ $[M+H]^+$: 603.1729, found: 603.1734.

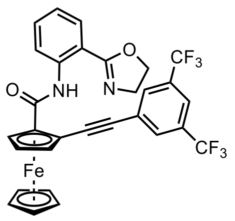

3j

2-((3,5-Bis(trifluoromethyl)phenyl)ethynyl)-*N*-(2-(4,5-dihydrooxazol-2-yl)phenyl)ferrocenyl-1-carboxamide

3j was synthesized following the general procedure. After purification by column chromatography on silica gel (PE/EA/DCM = 30/1/1), **3j** was obtained in 50% yield (30.8 mg).

Orange solid, $R_f = 0.5$ (PE/EA/DCM = 25/2/1), ^{1}H NMR (400 MHz, $CDCl_3$) δ 12.54 (s, 1H), 8.87 (d, $J = 8.4$ Hz, 1H), 7.99–7.83 (m, 3H), 7.77 (s, 1H),7.52 (t, $J = 6.8$ Hz, 1H), 7.12 (t, $J = 7.6$ Hz, 1H), 5.05 (s, 1H), 4.78 (s, 1H), 4.55 (s, 1H), 4.43–4.24 (m, 6H), 4.25–4.01 (m, 3H); ^{13}C NMR (100 MHz, $CDCl_3$) δ 168.5, 164.9, 140.0, 132.7, 131.7 (q, J = 33.4 Hz), 131.1, 129.4, 126.3, 123.0 (q, $J = 271.2$ Hz), 122.2, 120.8, 120.2, 113.0, 91.6, 86.1, 79.1, 74.9, 72.0, 70.8, 70.3, 66.0, 64.7, 54.8; ^{19}F NMR (375 MHz, $CDCl_3$) δ -63.09 (s, 6F); HRMS (ESI-TOF) *m/z* calculated for $C_{30}H_{21}N_2O_2F_6Fe$ $[M+H]^+$: 611.0851, found: 611.0854.

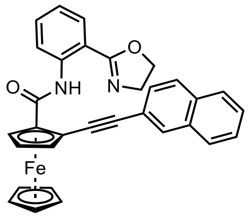

3k (mono)

***N*-(2-(4,5-Dihydrooxazol-2-yl)phenyl)-2-(naphthalen-2-ylethynyl)ferrocenyl-1-carboxamide**

3k (mono) and **3k'** (di) were synthesized following the general procedure (**3k/3k'** = 4.4/1.0). After purification by column chromatography on silica gel (PE/EA/DCM = 30/1/1), **3k** (mono) was obtained in 36% yield (19.0 mg). **3k'** (di) was obtained in 9% yield (6.3 mg).

Orange solid, $R_f = 0.5$ (PE/EA/DCM = 30/2/1), ^{1}H NMR (400 MHz, $CDCl_3$) δ 12.53 (s, 1H), 8.91 (d, $J = 8.4$ Hz, 1H), 8.01–7.85 (m, 2H), 7.84–7.68 (m, 3H), 7.59–7.40 (m, 4H), 7.13 (t, $J = 7.6$ Hz, 1H), 5.01 (s, 1H), 4.77 (s, 1H), 4.51 (t, $J = 2.8$ Hz, 1H), 4.39 (s, 5H), 4.21–3.84 (m, 4H); ^{13}C NMR (100 MHz, $CDCl_3$) δ 168.9, 164.3, 140.2, 133.0, 132.5, 132.5, 130.8, 129.3, 128.1, 127.8, 127.7, 127.6, 126.5, 126.4, 122.1, 121.2, 120.4, 113.3, 89.8, 87.7, 79.1, 74.5, 71.8, 70.3, 70.2, 66.1, 66.0, 54.8; HRMS (ESI-TOF) *m/z* calculated for $C_{32}H_{25}N_2O_2Fe$ $[M+H]^+$: 525.1260, found: 525.1261.

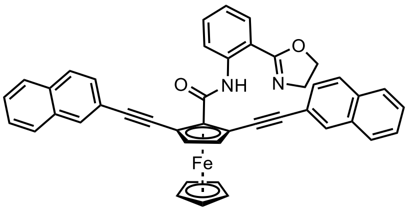

3k' (di)

_N_-(2-(4,5-Dihydrooxazol-2-yl)phenyl)-2,5-bis(naphthalen-2-ylethynyl)ferrocenyl-1-carboxamide

Orange solid, R_f = 0.7 (PE/EA/DCM = 30/2/1), ^{1}H NMR (400 MHz, $CDCl_3$) δ 12.78 (s, 1H), 9.09 (d, J = 8.6 Hz, 1H), 7.97 (d, J = 7.6 Hz, 1H), 7.92 (s, 2H), 7.83–7.68 (m, 6H), 7.58 (t, J = 8.0 Hz, 1H), 7.47 (m, 6H), 7.18 (m, 1H), 4.85 (s, 2H), 4.54 (s, 5H), 3.85 (s, 4H); ^{13}C NMR (100 MHz, $CDCl_3$) δ 167.3, 163.9, 140.2, 133.0, 132.6, 132.5, 131.0, 129.3, 128.2, 127.8, 127.7, 127.6, 126.5, 126.5, 122.3, 120.9, 120.6, 113.5, 90.0, 86.9, 81.9, 73.8, 73.5, 67.2, 65.8, 54.8; HRMS (ESI-TOF) *m/z* calculated for $C_{44}H_{31}N_2O_2Fe$ $[M+H]^+$: 675.1730, found: 675.1730.

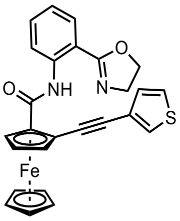

3l (mono)

_N_-(2-(4,5-Dihydrooxazol-2-yl)phenyl)-2-(thiophen-3-ylethynyl)ferrocenyl-1-carboxamide

3l (mono) and **3l'** (di) were synthesized following the general procedure (**3l/3l'** = 12/1.0). After purification by column chromatography on silica gel (PE/EA/DCM = 30/1/1), **3l** (mono) was obtained in 59% yield (28.2 mg). **3l'** (di) was obtained in 5% yield (3.1 mg) as an orange solid (R_f = 0.7 (PE/EA/DCM = 30/2/1)).

Orange solid, R_f = 0.5 (PE/EA/DCM = 30/2/1), ^{1}H NMR (400 MHz, $CDCl_3$) δ 12.44 (s, 1H), 8.86 (d, J = 8.8 Hz, 1H), 7.90 (d, J = 8.0 Hz, 1H), 7.50 (t, J = 7.6 Hz, 1H), 7.40–7.35 (m, 1H), 7.30–7.20 (m, 1H), 7.14–7.05 (m, 2H), 4.98 (s, 1H), 4.71 (s, 1H), 4.47 (s, 1H), 4.36 (s, 5H), 4.33–4.19 (m, 1H), 4.21–3.94 (m, 3H); ^{13}C NMR (100 MHz, $CDCl_3$) δ 168.9, 164.2, 140.1, 132.4, 129.7, 129.3, 127.8, 125.1, 122.9, 122.0, 120.3, 113.3, 86.4, 84.5, 78.8, 74.4, 71.8, 70.2, 70.0, 66.2, 66.1, 54.8; HRMS (ESI-TOF) *m/z* calculated for $C_{26}H_{21}N_2O_2SFe$ $[M+H]^+$: 481.0668, found: 481.0675.

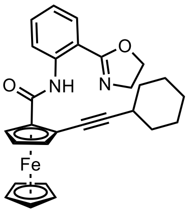

3m

2-(Cyclohexylethynyl)-_N_-(2-(4,5-dihydrooxazol-2-yl)phenyl)ferrocenyl-1-carboxamide

3m was synthesized following the general procedure. After purification by column chromatography on silica gel

(PE/EA/DCM = 30/1/1), **3m** was obtained in 75% yield (35.8 mg).

Orange solid, R_f = 0.5 (PE/EA/DCM = 25/2/1), ^{1}H NMR (400 MHz, $CDCl_3$) δ 12.23 (s, 1H), 8.80 (d, *J* = 8.8 Hz, 1H), 7.89 (d, *J* = 8.0 Hz, 1H), 7.48 (t, *J* = 8.0 Hz, 1H), 7.09 (t, *J* = 6.6 Hz, 1H), 4.86 (s, 1H), 4.60 (s, 1H), 4.46–4.34 (m, 3H), 4.33–4.18 (m, 7H), 2.58–2.45 (m, 1H), 1.83 (s, 2H), 1.72 (s, 2H), 1.60–1.40 (m, 2H), 1.36–1.19 (m, 4H); ^{13}C NMR (100 MHz, $CDCl_3$) δ 169.0, 164.4, 140.1, 132.3, 129.2, 121.9, 120.5, 113.3, 94.2, 78.1, 76.5, 74.6, 71.6, 69.5, 69.1, 67.9, 66.0, 54.9, 32.7, 32.6, 25.9, 24.9; HRMS (ESI-TOF) *m/z* calculated for $C_{28}H_{29}N_2O_2Fe$ $[M+H]^+$: 481.1573, found: 481.1576.

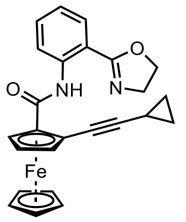

3n

2-(Cyclopropylethynyl)-*N*-(2-(4,5-dihydrooxazol-2-yl)phenyl)ferrocenyl-1-carboxamide

3n was synthesized following the general procedure. After purification by column chromatography on silica gel (PE/EA/DCM = 30/1/1), **3n** was obtained in 73% yield (31.8 mg).

Orange solid, R_f = 0.5 (PE/EA/DCM = 25/2/1), ^{1}H NMR (400 MHz, $CDCl_3$) δ 12.30 (s, 1H), 8.84 (d, *J* = 8.6 Hz, 1H), 7.90 (d, *J* = 8.0 Hz, 1H), 7.49 (t, *J* = 8.0 Hz, 1H), 7.09 (t, *J* = 8.0 Hz, 1H), 4.87 (s, 1H), 4.58 (s, 1H), 4.49–4.34 (m, 3H), 4.34–4.17 (m, 7H), 1.44–1.32 (m, 1H), 0.85–0.67 (m, 4H); ^{13}C NMR (100 MHz, $CDCl_3$) δ 169.0, 164.4, 140.1, 132.4, 129.2, 121.9, 120.4, 113.2, 93.1, 78.1, 74.6, 71.9, 71.6, 69.6, 69.1, 67.7, 66.1, 54.9, 8.5, 0.7; HRMS (ESI-TOF) *m/z* calculated for $C_{25}H_{23}N_2O_2Fe$ $[M+H]^+$: 439.1103, found: 439.1102.

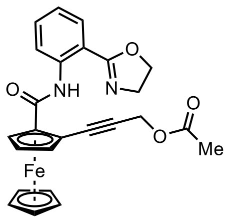

3o

2-(2-((2-(4,5-Dihydrooxazol-2-yl)phenyl)carbamoyl)ferrocenyl-1-yl)prop-1-yn-1-yl acetate

3o was synthesized following the general procedure. After purification by column chromatography on silica gel (PE/EA/DCM = 30/1/1), **3o** was obtained in 74% yield (34.6 mg).

Orange solid, R_f = 0.4 (PE/EA/DCM = 30/2/1), ^{1}H NMR (400 MHz, $CDCl_3$) δ 12.41 (s, 1H), 8.85 (d, *J* = 8.8 Hz, 1H), 7.90 (d, *J* = 8.0 Hz, 1H), 7.48 (t, *J* = 8.0 Hz, 1H), 7.09 (t, *J* = 7.6 Hz, 1H), 4.94 (s, 1H), 4.86 (s, 2H), 4.69 (s, 1H), 4.47–4.39 (m, 3H), 4.31 (s, 5H), 4.24 (t, *J* = 9.6 Hz, 2H), 2.08 (s, 3H); ^{13}C NMR (100 MHz, $CDCl_3$) δ 170.4, 168.6, 164.7, 140.1, 132.5, 129.2, 122.0, 120.2, 113.1, 84.0, 82.4, 78.4, 75.2, 71.8, 70.3, 69.6, 66.1, 65.3, 54.8, 53.4, 20.8; HRMS (ESI-TOF) *m/z* calculated for $C_{25}H_{23}N_2O_4Fe$ $[M+H]^+$: 471.1002, found: 471.1002.

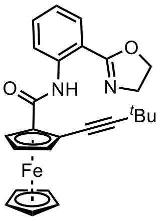

3p (mono)

***N*-(2-(4,5-Dihydrooxazol-2-yl)phenyl)-2-(3,3-dimethylbut-1-yn-1-yl)ferrocenyl-1-carboxamide**

3p (mono) and **3p'** (di) were synthesized following the general procedure (**3p/3p'** = 11/1.0). After purification by column chromatography on silica gel (PE/EA/DCM = 30/1/1), **3p** (mono) was obtained in 74% yield (33.7 mg), as an orange solid. **3p'** (di) was obtained in 5% yield (2.9 mg) as an orange solid (R_f = 0.7 (PE/EA/DCM = 30/2/1)).
Orange solid, R_f = 0.5 (PE/EA/DCM = 30/2/1), ^{1}H NMR (400 MHz, $CDCl_3$) δ 12.11 (s, 1H), 8.70 (d, J = 9.2 Hz, 1H), 7.90 (d, J = 8.0 Hz, 1H), 7.48 (t, J = 7.8 Hz, 1H), 7.08 (t, J = 7.6 Hz, 1H), 4.84 (s, 1H), 4.57 (s, 1H), 4.46–4.34 (m, 3H), 4.34–4.18 (m, 7H), 1.24 (s, 9H); ^{13}C NMR (100 MHz, $CDCl_3$) δ 169.0, 164.3, 140.0, 132.3, 129.2, 121.9, 120.8, 113.5, 98.4, 78.6, 75.0, 74.2, 71.6, 69.4, 69.1, 67.8, 66.0, 55.0, 30.9, 28.1; HRMS (ESI-TOF) *m/z* calculated for $C_{26}H_{27}N_2O_2Fe$ $[M+H]^+$: 455.1416, found: 455.1420.

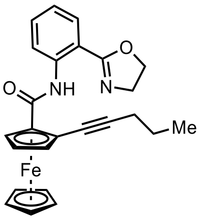

3q (mono)

***N*-(2-(4,5-Dihydrooxazol-2-yl)phenyl)-2-(pent-1-yn-1-yl)ferrocenyl-1-carboxamide**

3q (mono) and **3q'** (di) were synthesized following the general procedure (**3q/3q'** = 7.7/1.0). After purification by column chromatography on silica gel (PE/EA/DCM = 30/1/1), **3q** (mono) was obtained in 69% yield (30.2 mg). **3q'** (di) was obtained in 9% yield (4.7 mg).
Orange solid, R_f = 0.5 (PE/EA/DCM = 30/2/1), ^{1}H NMR (400 MHz, $CDCl_3$) δ 12.29 (s, 1H), 8.84 (d, J = 8.0 Hz, 1H), 7.89 (d, J = 8.0 Hz, 1H), 7.47 (t, J = 7.0 Hz, 1H), 7.07 (t, J = 7.6 Hz, 1H), 4.88 (s, 1H), 4.60 (s, 1H), 4.48–4.34 (m, 3H), 4.29 (s, 5H), 4.23 (t, J = 9.0 Hz, 2H), 2.32 (t, J = 7.4 Hz, 2H), 1.65–1.52 (m, 2H), 1.00 (t, J = 7.2 Hz, 3H); ^{13}C NMR (100 MHz, $CDCl_3$) δ 169.0, 164.5, 140.1, 132.4, 129.2, 121.8, 120.4, 113.2, 90.0, 77.8, 76.8, 74.7, 71.6, 69.5, 69.0, 67.9, 66.0, 54.9, 22.2, 21.8, 13.6; HRMS (ESI-TOF) *m/z* calculated for $C_{25}H_{25}N_2O_2Fe$ $[M+H]^+$: 441.1260, found: 441.1264.

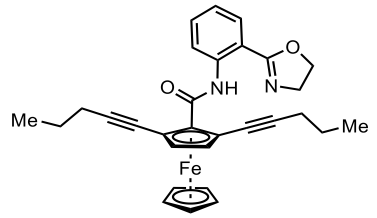

3q' (di)

N-(2-(4,5-Dihydrooxazol-2-yl)phenyl)-2,5-di(pent-1-yn-1-yl)ferrocenyl-1-carboxamide

Orange solid, R_f = 0.7 (PE/EA/DCM = 30/2/1), ^{1}H NMR (400 MHz, $CDCl_3$) δ 12.23 (s, 1H), 8.83 (d, J = 8.4 Hz, 1H), 7.88 (d, J = 8.0 Hz, 1H), 7.47 (t, J = 7.8 Hz, 1H), 7.08 (t, J = 7.6 Hz, 1H), 4.54 (s, 2H), 4.44–4.33 (m, 7H), 4.20 (t, J = 9.4 Hz, 2H), 2.23 (t, J = 7.0 Hz, 4H), 1.55–1.45 (m, 4H), 0.93 (t, J = 7.2 Hz, 6H); ^{13}C NMR (100 MHz, $CDCl_3$) δ 167.6, 163.9, 140.0, 132.3, 129.2, 122.0, 121.0, 113.7, 90.2, 81.3, 76.3, 73.3, 72.7, 67.7, 66.0, 55.1, 22.1, 21.7, 13.5; HRMS (ESI-TOF) *m/z* calculated for $C_{30}H_{31}N_2O_2Fe$ $[M+H]^+$: 507.1729, found: 507.1729.

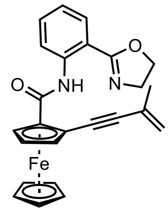

3r

N-(2-(4,5-Dihydrooxazol-2-yl)phenyl)-2-(3-methylbut-3-en-1-yn-1-yl)ferrocenyl-1-carboxamide

3r was synthesized following the general procedure. After purification by column chromatography on silica gel (PE/EA/DCM = 30/1/1), **3r** was obtained in 62% yield (27.0 mg).

Orange solid, R_f = 0.5 (PE/EA/DCM = 30/2/1),^{1}H NMR (400 MHz, $CDCl_3$) δ 12.35 (s, 1H), 8.82 (d, J = 8.4 Hz, 1H), 7.89 (d, J = 8.0 Hz, 1H), 7.48 (d, J = 8.8 Hz, 1H), 7.08 (t, J = 7.6 Hz 1H), 5.28 (s, 1H), 5.22 (s, 1H), 4.94 (s, 1H), 4.65 (s, 1H), 4.44 (t, J = 2.6 Hz, 1H), 4.42–4.36 (m, 1H), 4.34 (s, 5H), 4.25–4.18 (m, 3H), 1.92 (s, 3H); ^{13}C NMR (100 MHz, $CDCl_3$) δ 168.8, 164.3, 140.1, 132.4, 129.2, 127.3, 122.0, 120.9, 120.4, 113.3, 90.5, 85.9, 78.7, 74.6, 71.7, 70.1, 69.9, 66.4, 66.1, 54.9, 23.4; HRMS (ESI-TOF) *m/z* calculated for $C_{25}H_{23}N_2O_2Fe$ $[M+H]^+$: 439.1103, found: 439.1105.

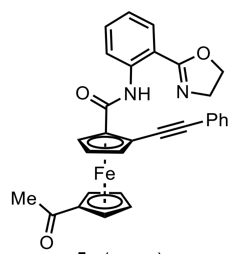

5a (mono)

1'-Acetyl-N-(2-(4,5-dihydrooxazol-2-yl)phenyl)-2-(phenylethynyl)ferrocenyl-1-carboxamide

5a (mono) and **5a’** (di) were synthesized following the general procedure (**5a/5a’** = 5.8/1.0). After purification by column chromatography on silica gel (PE/EA/DCM = 4/1/1), **5a** (mono) was obtained in 64% yield (33.1 mg). **5a’** (di) was obtained in 11% yield (6.9 mg).

Orange solid, R_f = 0.4 (PE/EA/DCM = 2/1/1), ^{1}H NMR (400 MHz, $CDCl_3$) δ 12.51 (s, 1H), 8.88 (d, J = 8.4 Hz, 1H), 7.91 (d, J = 8.0 Hz, 1H), 7.57–7.41 (m, 3H), 7.31 (d, J = 5.2 Hz, 3H), 7.13 (t, J = 7.6 Hz, 1H), 5.00 (s, 1H), 4.90 (d, J = 7.2 Hz, 2H), 4.69 (d, J = 8.8 Hz, 2H), 4.63 (s, 1H), 4.52 (s, 1H), 4.30–4.20 (m, 1H), 4.20–4.09 (m, 1H), 4.09–3.96 (m, 2H), 2.43 (s, 3H); ^{13}C NMR (100 MHz, $CDCl_3$) δ 201.1, 167.3, 164.4, 139.8, 132.6, 131.4, 129.3, 128.3, 128.1, 123.4, 122.3, 120.3, 113.3, 90.7, 85.3, 81.4, 79.8, 76.3, 76.2, 75.7, 73.4, 71.8, 71.7, 71.3, 67.9, 66.1, 54.7, 27.8; HRMS (ESI-TOF) *m/z* calculated for $C_{30}H_{25}N_2O_3Fe$ [M+H]$^+$: 517.1209, found: 517.1203.

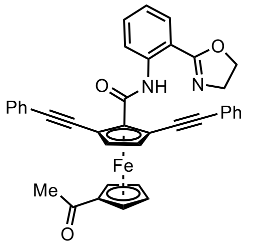

5a' (di)

1'-Acetyl-*N*-(2-(4,5-dihydrooxazol-2-yl)phenyl)-2,5-bis(phenylethynyl)ferrocenyl-1-carboxamide

Brown solid, R_f = 0.6 (PE/EA/DCM = 2/1/1), ^{1}H NMR (400 MHz, $CDCl_3$) δ 12.67 (s, 1H), 9.01 (d, J = 8.4 Hz, 1H), 7.91 (d, J = 8.0 Hz, 1H), 7.55 (t, J = 8.0 Hz, 1H), 7.45–7.39 (m, 4H), 7.33–7.27 (m, 6H), 7.15 (t, J = 7.7 Hz, 1H), 5.03 (s, 2H), 4.80–4.71 (m, 4H), 4.00–3.80 (m, 4H), 2.46 (s, 3H); ^{13}C NMR (100 MHz, $CDCl_3$) δ 200.8, 165.8, 163.9, 139.8, 132.6, 131.5, 129.3, 128.3, 123.1, 122.5, 120.5, 113.5, 90.9, 84.6, 82.7, 82.2, 78.4, 74.8, 73.9, 68.6, 65.9, 54.8, 28.0; HRMS (ESI-TOF) *m/z* calculated for $C_{38}H_{29}N_2O_3Fe$ [M+H]$^+$: 617.1522, found: 617.1520.

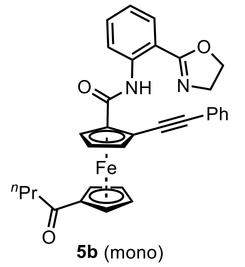

5b (mono)

1'-Butyryl-*N*-(2-(4,5-dihydrooxazol-2-yl)phenyl)-2-(phenylethynyl)ferrocenyl-1-carboxamide

5b (mono) and **5b’** (di) were synthesized following the general procedure (**5b/5b’** = 4.8/1.0). After purification by column chromatography on silica gel (PE/EA/DCM = 4/1/1), **5b** (mono) was obtained in 62% yield (30.5 mg). **5b’** (di) was obtained in 13% yield (8.7 mg).

Brown solid, R_f = 0.5 (PE/EA/DCM = 2/1/1), ^{1}H NMR (400 MHz, $CDCl_3$) δ 12.51 (s, 1H), 8.89 (d, J = 8.4 Hz, 1H), 7.91 (d, J = 8.0 Hz, 1H), 7.59–7.37 (m, 3H), 7.31 (s, 3H), 7.13 (t, J = 7.8 Hz, 1H), 5.06–4.84 (m, 3H), 4.75–4.56 (m,

3H), 4.51 (s, 1H), 4.34–4.18 (m, 1H), 4.18–3.92 (m, 3H), 2.88–2.58 (m, 2H), 1.76–1.59 (m, 2H), 0.91 (t, J = 7.6 Hz, 3H); ^{13}C NMR (100 MHz, $CDCl_3$) δ 203.4, 167.3, 164.3, 139.8, 132.5, 131.3, 129.3, 128.2, 128.1, 123.4, 122.3, 120.2, 113.3, 90.6, 85.5, 81.2, 79.8, 76.5, 76.1, 75.6, 73.0, 71.8, 71.4, 71.1, 67.7, 66.0, 54.7, 41.9, 17.5, 13.9; HRMS (ESI-TOF) *m/z* calculated for $C_{32}H_{29}N_2O_3Fe$ $[M+H]^+$: 545.1522, found: 545.1529.

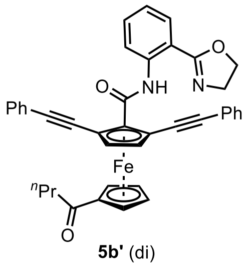

5b' (di)

1'-Butyryl-*N*-(2-(4,5-dihydrooxazol-2-yl)phenyl)-2,5-bis(phenylethynyl)ferrocenyl-1-carboxamide

Brown solid, R_f = 0.7 (PE/EA/DCM = 2/1/1), ^{1}H NMR (400 MHz, $CDCl_3$) δ 12.68 (s, 1H), 9.02 (d, J = 8.4 Hz, 1H), 7.91 (d, J = 7.8 Hz, 1H), 7.54 (t, J = 8.0 Hz, 1H), 7.46–7.38 (m, 4H), 7.34–7.27 (m, 6H), 7.15 (t, J = 7.6 Hz, 1H), 5.04 (s, 2H), 4.77 (s, 2H), 4.73 (s, 2H), 4.00–3.82 (m, 4H), 2.79 (t, J = 7.4 Hz, 2H), 1.73–1.62 (m, 2H), 0.89 (t, J = 7.2 Hz, 3H); ^{13}C NMR (100 MHz, $CDCl_3$) δ 203.0, 165.8, 163.9, 139.8, 132.5, 131.4, 129.3, 128.3, 128.2, 123.1, 122.4, 120.5, 113.5, 90.9, 84.8, 82.6, 81.9, 78.5, 74.9, 73.6, 68.4, 65.9, 54.8, 42.0, 17.4, 13.9; HRMS (ESI-TOF) *m/z* calculated for $C_{40}H_{33}N_2O_3Fe$ $[M+H]^+$: 645.1835, found: 645.1832.

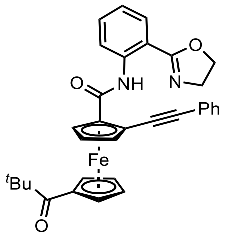

5c (mono)

***N*-(2-(4,5-Dihydrooxazol-2-yl)phenyl)-2-(phenylethynyl)-1'-pivaloyl ferrocenyl-1-carboxamide**

5c (mono) and **5c'** (di) were synthesized following the general procedure (**5c**/**5c'** = 6.3/1.0). After purification by column chromatography on silica gel (PE/EA/DCM = 4/1/1), **5c** (mono) was obtained in 63% yield (35.1 mg). **5c'** (di) was obtained in 10% yield (6.5 mg).

Brown solid, R_f = 0.6 (PE/EA/DCM = 2/1/1), ^{1}H NMR (400 MHz, $CDCl_3$) δ 12.49 (s, 1H), 8.88 (d, J = 8.4 Hz, 1H), 7.91 (d, J = 8.0 Hz, 1H), 7.51 (t, J = 7.8 Hz, 1H), 7.44–7.39 (m, 2H), 7.31–7.26 (m, 3H), 7.12 (t, J = 7.6 Hz, 1H), 5.08–4.95 (m, 3H), 4.71 (s, 1H), 4.64 (s, 2H), 4.47 (t, J = 2.8 Hz, 1H), 4.28–4.18 (m, 1H), 4.13–3.93 (m, 3H), 1.32 (s, 9H); ^{13}C NMR (100 MHz, $CDCl_3$) δ 209.2, 167.7, 164.3, 139.9, 132.5, 131.2, 129.3, 128.2, 128.0, 123.5, 122.2, 120.2, 113.3, 90.5, 85.9, 79.7, 78.5, 76.9, 76.3, 75.1, 74.2, 72.9, 72.3, 71.3, 67.6, 66.0, 54.8, 44.5, 27.9; HRMS (ESI-TOF) *m/z* calculated for $C_{33}H_{31}N_2O_3Fe$ $[M+H]^+$: 559.1679, found: 559.1681.

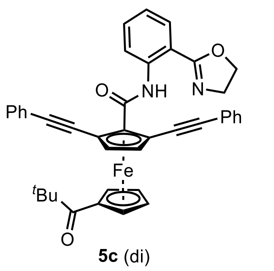

5c (di)

***N*-(2-(4,5-Dihydrooxazol-2-yl)phenyl)-2,5-bis(phenylethynyl)-1'-pivaloyl ferrocenyl-1-carboxamide**

Brown solid, R_f = 0.8 (PE/EA/DCM = 2/1/1), ^{1}H NMR (400 MHz, $CDCl_3$) δ 12.68 (s, 1H), 9.01 (d, *J* = 8.4 Hz, 1H), 7.91 (d, *J* = 7.8 Hz, 1H), 7.53 (t, *J* = 8.2 Hz, 1H), 7.55–7.42 (m, 4H), 7.32–7.27 (m, 6H), 7.14 (t, *J* = 7.6 Hz, 1H), 5.10 (s, 2H), 4.78–4.71 (m, 4H), 3.99–3.83 (m, 4H), 1.32 (s, 9H); ^{13}C NMR (100 MHz, $CDCl_3$) δ 208.5, 166.0, 163.9, 139.9, 132.5, 131.4, 129.3, 128.2, 128.1, 123.2, 122.4, 120.4, 113.4, 90.8, 85.2, 82.1, 79.6, 78.2, 75.1, 75.0, 74.9, 68.7, 65.9, 54.8, 44.5, 27.8; HRMS (ESI-TOF) *m/z* calculated for $C_{41}H_{35}N_2O_3Fe$ [M+H]$^+$: 659.1992, found: 659.1984.

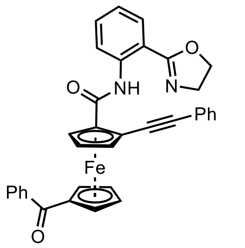

5d (mono)

1'-Benzoyl-*N*-(2-(4,5-dihydrooxazol-2-yl)phenyl)-2-(phenylethynyl)ferrocenyl-1-carboxamide

5d (mono) and **5d'** (di) were synthesized following the general procedure (**5d/5d'** = 4.8/1.0). After purification by column chromatography on silica gel (PE/EA/DCM = 4/1/1), **5d** (mono) was obtained in 72% yield (41.8 mg). **5d'** (di) was obtained in 15% yield (9.9 mg).

Brown solid, R_f = 0.8 (PE/EA/DCM = 2/1/1), ^{1}H NMR (400 MHz, $CDCl_3$) δ 12.50 (s, 1H), 8.83 (d, *J* = 8.5 Hz, 1H), 7.90 (t, *J* = 8.2 Hz, 3H), 7.53–7.42 (m, 2H), 7.42–7.32 (m, 4H), 7.32–7.23 (m, 3H), 7.12 (t, *J* = 7.6 Hz, 1H), 5.12 (s, 1H), 5.06 (s, 1H), 5.02 (s, 1H), 4.82–4.67 (m, 3H), 4.52 (s, 1H), 4.28–4.16 (m, 1H), 4.14–3.82 (m, 3H); ^{13}C NMR (100 MHz, $CDCl_3$) δ 197.8, 167.3, 164.3, 139.8, 139.2, 132.5, 131.8, 131.3, 129.2, 128.3, 128.2, 128.1, 123.4, 122.3, 120.2, 113.3, 90.7, 85.5, 80.0, 79.9, 77.6, 76.3, 75.0, 73.2, 72.7, 71.6, 68.0, 66.0, 54.7; HRMS (ESI-TOF) *m/z* calculated for $C_{35}H_{27}N_2O_3Fe$ [M+H]$^+$: 579.1365, found: 579.1357.

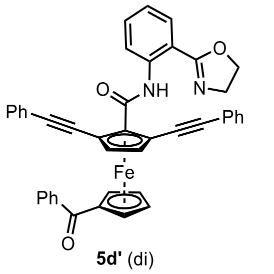

5d' (di)

1'-Benzoyl-*N*-(2-(4,5-dihydrooxazol-2-yl)phenyl)-2,5-bis(phenylethynyl)ferrocenyl-1-carboxamide

Brown solid, R_f = 0.9 (PE/EA/DCM = 2/1/1), ^{1}H NMR (400 MHz, $CDCl_3$) δ 12.69 (s, 1H), 9.00 (d, J = 8.4 Hz, 1H), 7.96 (d, J = 7.6 Hz, 2H), 7.89 (d, J = 7.6 Hz, 1H), 7.53 (t, J = 7.8 Hz, 1H), 7.42 (t, J = 7.4 Hz, 1H), 7.36–7.24 (m, 12H), 7.14 (t, J = 7.4 Hz, 1H), 5.19 (s, 2H), 4.88 (s, 2H), 4.78 (s, 2H), 3.96–3.79 (m, 4H); ^{13}C NMR (100 MHz, $CDCl_3$) δ 197.4, 165.8, 163.9, 139.8, 139.2, 132.5, 131.8, 131.4, 129.3, 128.5, 128.2, 123.1, 122.4, 120.4, 113.4, 91.0, 84.7, 82.5, 80.9, 79.1, 75.5, 75.5, 68.7, 65.9, 54.7; HRMS (ESI-TOF) *m/z* calculated for $C_{43}H_{31}N_2O_3Fe$ $[M+H]^+$: 679.1679, found: 679.1680.

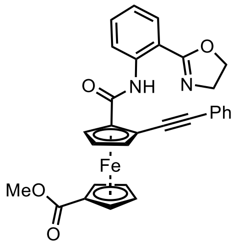

5e (mono)

Methyl 1'-((2-(4,5-dihydrooxazol-2-yl)phenyl)carbamoyl)-2'-(phenylethynyl)ferrocenyl-1-carboxylate

5e (mono) and **5e'** (di) were synthesized following the general procedure (**5e/5e'** = 7.4/1.0). After purification by column chromatography on silica gel (PE/EA/DCM = 4/1/1), **5e** (mono) was obtained in 52% yield (27.8 mg). **5e'** (di) was obtained in 7% yield (4.6 mg).

Orange solid, R_f = 0.4 (PE/EA/DCM = 2/1/1), ^{1}H NMR (400 MHz, $CDCl_3$) δ 12.51 (s, 1H), 8.89 (d, J = 8.4 Hz, 1H), 7.91 (d, J = 8.0 Hz, 1H), 7.52 (t, J = 7.8 Hz, 1H), 7.49–7.40 (m, 2H), 7.35–7.28 (m, 3H), 7.12 (t, J = 7.6 Hz, 1H), 5.05–4.97 (m, 2H), 4.95 (s, 1H), 4.73 (s, 1H), 4.61 (s, 1H), 4.57 (s, 1H), 4.49 (s, 1H), 4.30–4.18 (m, 1H), 4.18–3.94 (m, 3H), 3.72 (s, 3H); ^{13}C NMR (100 MHz, $CDCl_3$) δ 170.4, 167.4, 164.3, 140.0, 132.5, 131.4, 129.3, 128.2, 128.0, 123.6, 122.2, 120.3, 113.3, 90.4, 85.5, 79.9, 75.9, 75.8, 75.1, 73.9, 73.5, 72.6, 72.0, 71.4, 67.8, 66.1, 54.7, 51.8; HRMS (ESI-TOF) *m/z* calculated for $C_{30}H_{25}N_2O_4Fe$ $[M+H]^+$: 533.1158, found: 533.1153.

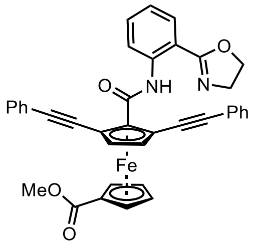

5e' (di)

Methyl 1'-((2-(4,5-dihydrooxazol-2-yl)phenyl)carbamoyl)-2',5'-bis(phenylethynyl)ferrocenyl-1-carboxylate

Orange solid, R_f = 0.6 (PE/EA/DCM = 2/1/1), ^{1}H NMR (400 MHz, $CDCl_3$) δ 12.68 (s, 1H), 9.02 (d, J = 8.5 Hz, 1H), 7.91 (d, J = 8.0 Hz, 1H), 7.54 (t, J = 7.9 Hz, 1H), 7.46–7.42 (m, 4H), 7.32–7.28 (m, 6H), 7.14 (t, J = 7.5 Hz, 1H), 5.09 (s, 2H), 4.77 (s, 2H), 4.72 (s, 2H), 4.01–3.85 (m, 4H), 3.71 (s, 3H); ^{13}C NMR (100 MHz, $CDCl_3$) δ 171.2, 169.6,

165.9, 163.9, 140.0, 132.5, 131.5, 129.3, 128.3, 128.1, 123.3, 122.4, 120.5, 113.5, 90.7, 84.8, 82.5, 75.4, 74.9, 74.7, 68.7, 65.9, 60.4, 54.8, 53.4, 52.0; HRMS (ESI-TOF) *m/z* calculated for $C_{38}H_{29}N_2O_4Fe$ $[M+H]^+$: 633.1471, found: 633.1467.

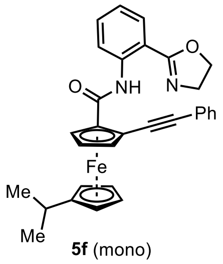

5f (mono)

N-(2-(4,5-Dihydrooxazol-2-yl)phenyl)-1'-isopropyl-2-(phenylethynyl)ferrocenyl-1-carboxamide

5f (mono) and **5f'** (di) were synthesized following the general procedure (**5f/5f'** = 6.9/1.0). After purification by column chromatography on silica gel (PE/EA/DCM = 30/1/1), **5f** (mono) was obtained in 62% yield (31.9 mg). **5f'** (di) was obtained in 10% yield (5.9 mg).

Orange solid, R_f = 0.4 (PE/EA/DCM = 20/2/1), ^{1}H NMR (400 MHz, $CDCl_3$) δ 12.38 (s, 1H), 8.88 (d, *J* = 8.5 Hz, 1H), 7.89 (d, *J* = 8.0 Hz, 1H), 7.49 (t, *J* = 8.0 Hz, 1H), 7.45–7.37 (m, 2H), 7.31–7.23 (m, 3H), 7.09 (t, *J* = 7.6 Hz, 1H), 4.96 (s, 1H), 4.67 (s, 1H), 4.45 (s, 1H), 4.34–4.14 (m, 5H), 4.14–3.88 (m, 3H), 2.80–2.62 (m, 1H), 1.18 (d, *J* = 6.8 Hz, 6H); ^{13}C NMR (100 MHz, $CDCl_3$) δ 168.9, 164.2, 140.1, 132.4, 131.1, 129.2, 128.2, 127.6, 124.0, 121.9, 120.3, 113.3, 99.2, 89.4, 87.2, 78.6, 75.3, 71.7, 71.1, 70.8, 70.5, 70.1, 68.8, 66.1, 66.0, 54.8, 26.7, 23.4, 23.4; HRMS (ESI-TOF) *m/z* calculated for $C_{31}H_{29}N_2O_2Fe$ $[M+H]^+$: 517.1573, found: 517.1579.

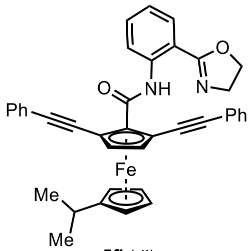

5f' (di)

N-(2-(4,5-Dihydrooxazol-2-yl)phenyl)-1'-isopropyl-2,5-bis(phenylethynyl)ferrocenyl-1-carboxamide

Orange solid, R_f = 0.5 (PE/EA/DCM = 20/2/1), ^{1}H NMR (400 MHz, $CDCl_3$) δ 12.58 (s, 1H), 9.00 (d, *J* = 8.4 Hz, 1H), 7.89 (d, *J* = 8.0 Hz, 1H), 7.52 (t, *J* = 7.2 Hz, 1H), 7.42–7.37 (m, 4H), 7.30–7.24 (m, 6H), 7.12 (t, *J* = 7.0 Hz, 1H), 4.73 (s, 2H), 4.43 (s, 2H), 4.36 (s, 2H), 3.98–3.81 (m, 4H), 2.85–2.72 (m, 1H), 1.21 (d, *J* = 6.8 Hz, 6H); ^{13}C NMR (100 MHz, $CDCl_3$) δ 167.2, 163.7, 140.1, 132.4, 131.2, 129.2, 128.2, 127.8, 123.7, 122.1, 120.5, 113.5, 99.7, 89.7, 86.4, 81.4, 74.2, 73.9, 71.0, 67.2, 65.8, 54.8, 26.2, 23.3; HRMS (ESI-TOF) *m/z* calculated for $C_{39}H_{33}N_2O_2Fe$ $[M+H]^+$: 617.1886, found: 617.1887.

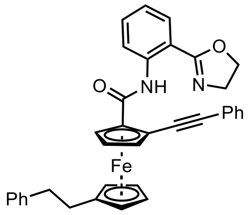

5g (mono)

***N*-(2-(4,5-Dihydrooxazol-2-yl)phenyl)-1'-phenethyl-2-(phenylethynyl)ferrocenyl-1-carboxamide**

5g (mono) and **5g'** (di) were synthesized following the general procedure (**5g/5g'** = 5.4/1.0). After purification by column chromatography on silica gel (PE/EA/DCM = 30/1/1), **5g** (mono) was obtained in 65% yield (37.6 mg). **5g'** (di) was obtained in 12% yield (8.1 mg)

Orange solid, R_f = 0.5 (PE/EA/DCM = 20/2/1), ^{1}H NMR (400 MHz, $CDCl_3$) δ 12.37 (s, 1H), 8.88 (d, *J* = 8.4 Hz, 1H), 7.89 (d, *J* = 7.8 Hz, 1H), 7.49 (t, *J* = 7.8 Hz, 1H), 7.43–7.34 (m, 2H), 7.28–7.21 (m, 3H), 7.21–7.12 (m, 3H), 7.12–7.00 (m, 3H), 4.95 (s, 1H), 4.65 (s, 1H), 4.43 (t, *J* = 2.8 Hz, 1H), 4.29–4.13 (m, 5H), 4.13–3.83 (m, 3H), 2.85–2.65 (m, 4H). ^{13}C NMR (100 MHz, $CDCl_3$) δ 168.7, 164.2, 141.7, 140.1, 132.4, 131.2, 129.2, 128.3, 128.2, 128.1, 127.6, 125.7, 123.9, 121.9, 120.3, 113.2, 91.0, 89.6, 87.0, 78.6, 75.4, 71.9, 71.3, 71.2, 71.0, 70.9, 70.6, 66.4, 66.0, 54.7, 37.1, 29.8; HRMS (ESI-TOF) *m/z* calculated for $C_{36}H_{31}N_2O_2Fe$ $[M+H]^+$: 579.1730, found: 579.1732.

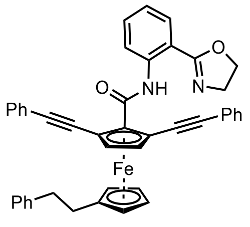

5g' (di)

***N*-(2-(4,5-Dihydrooxazol-2-yl)phenyl)-1'-phenethyl-2,5-bis(phenylethynyl)ferrocenyl-1-carboxamide**

Orange solid, R_f = 0.7 (PE/EA/DCM = 20/2/1), ^{1}H NMR (400 MHz, $CDCl_3$) δ 12.57 (s, 1H), 9.00 (d, *J* = 8.2 Hz, 1H), 7.89 (d, *J* = 7.6 Hz, 1H), 7.51 (t, *J* = 7.8 Hz, 1H), 7.35 (d, *J* = 7.2 Hz, 4H), 7.29–7.18 (m, 6H), 7.19–7.02 (m, 6H), 4.71 (s, 2H), 4.40 (s, 2H), 4.33 (s, 2H), 3.96–3.86 (m, 2H), 3.85–3.73 (t, *J* = 8.8 Hz, 2H), 2.87–2.78 (m, 2H), 2.77–2.68 (m, 2H).; ^{13}C NMR (100 MHz, $CDCl_3$) δ 167.1, 163.7, 141.7, 140.1, 132.4, 131.3, 131.3, 129.2, 128.4, 128.2, 127.8, 125.7, 123.6, 122.1, 120.6, 113.5, 91.5, 89.9, 86.2, 81.6, 74.3, 73.5, 73.1, 67.4, 65.8, 54.7, 36.9, 29.1; HRMS (ESI-TOF) *m/z* calculated for $C_{44}H_{35}N_2O_2Fe$ $[M+H]^+$: 679.2042, found: 679.2050.

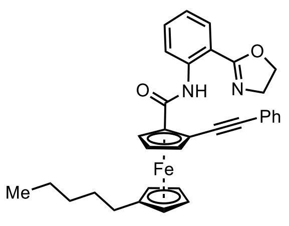

5h (mono)

N-(2-(4,5-Dihydrooxazol-2-yl)phenyl)-1'-pentyl-2-(phenylethynyl)ferrocenyl-1-carboxamide

5h (mono) and **5h'** (di) were synthesized following the general procedure (**5h/5h'** = 7.0/1.0). After purification by column chromatography on silica gel (PE/EA/DCM = 30/1/1), **5h** (mono) was obtained in 70% yield (38.1 mg). **5h'** (di) was obtained in 10% yield (6.7 mg).

Orange solid, R_f = 0.5 (PE/EA/DCM = 20/2/1), ^{1}H NMR (400 MHz, $CDCl_3$) δ 12.36 (s, 1H), 8.88 (d, J = 8.4 Hz, 1H), 7.89 (d, J = 7.6 Hz, 1H), 7.56–7.37 (m, 3H), 7.27 (d, J = 7.2 Hz, 3H), 7.08 (t, J = 7.6 Hz, 1H), 4.94 (s, 1H), 4.64 (s, 1H), 4.42 (s, 1H), 4.33–4.16 (m, 5H), 4.15–3.89 (m, 3H), 2.34 (t, J = 7.8 Hz, 2H), 1.53–1.40 (m, 2H), 1.33–1.16 (m, 4H), 0.81 (t, J = 6.6 Hz, 3H); ^{13}C NMR (100 MHz, $CDCl_3$) δ 168.8, 164.2, 140.2, 132.4, 131.2, 129.2, 128.1, 127.6, 124.0, 121.9, 120.3, 113.2, 92.2, 89.4, 87.7, 78.6, 75.4, 71.9, 71.2, 71.1, 70.9, 70.9, 70.6, 66.2, 66.0, 54.8, 31.7, 30.5, 27.9, 22.4, 14.0; HRMS (ESI-TOF) *m/z* calculated for $C_{33}H_{33}N_2O_2Fe$ $[M+H]^+$: 545.1886, found: 545.1891.

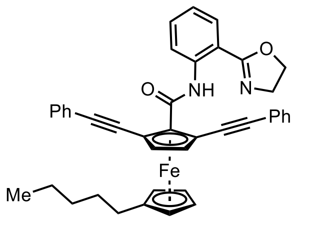

5h' (di)

N-(2-(4,5-Dihydrooxazol-2-yl)phenyl)-1'-pentyl-2,5-bis(phenylethynyl)ferrocenyl-1-carboxamide

Orange solid, R_f = 0.7 (PE/EA/DCM = 20/2/1), ^{1}H NMR (400 MHz, $CDCl_3$) δ 12.57 (s, 1H), 9.00 (d, J = 8.4 Hz, 1H), 7.89 (d, J = 7.6 Hz, 1H), 7.52 (d, J = 7.0 Hz, 1H), 7.44–7.38 (m, 4H), 7.30–7.24 (m, 6H), 7.12 (t, J = 7.6 Hz, 1H), 4.70 (s, 2H), 4.40 (s, 2H), 4.34 (s, 2H), 3.98–3.79 (m, 4H), 2.37 (t, J = 7.8 Hz, 2H), 1.55–1.45 (m, 2H), 1.26–1.16 (m, 4H), 0.79 (t, J = 6.6 Hz, 3H); ^{13}C NMR (100 MHz, $CDCl_3$) δ 167.2, 163.7, 140.1, 132.4, 131.3, 129.2, 128.2, 127.8, 123.7, 122.1, 120.6, 113.5, 92.7, 89.7, 86.4, 81.5, 74.3, 73.5, 73.0, 67.3, 65.8, 54.8, 31.8, 30.4, 27.3, 22.5, 14.0; HRMS (ESI-TOF) *m/z* calculated for $C_{41}H_{37}N_2O_2Fe$ $[M+H]^+$: 645.2199, found: 645.2207.

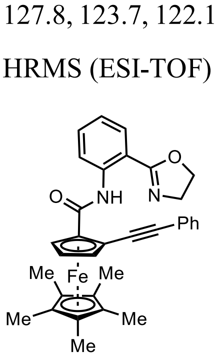

5l

N-(2-(4,5-Dihydrooxazol-2-yl)phenyl)-1',2',3',4',5'-pentamethyl-2-(phenylethynyl)ferrocenyl-1-carboxamide

5l was synthesized following the general procedure. After purification by column chromatography on silica gel (PE/EA/DCM = 30/1/1), **5l** was obtained in 38% yield (20.7 mg).

Orange solid, R_f = 0.5 (PE/EA/DCM = 30/1/1), ^{1}H NMR (400 MHz, $CDCl_3$) δ 12.07 (s, 1H), 8.93 (d, J = 8.4 Hz, 1H),

7.88 (d, J = 8.0 Hz, 1H), 7.59–7.51 (m, 2H), 7.53–7.43 (m, 1H), 7.37–7.27 (m, 3H), 7.06 (t, J = 7.6 Hz, 1H), 4.65 (s, 1H), 4.37–4.28 (m, 1H), 4.28–4.16 (m, 2H), 4.15–3.96 (m, 3H), 1.82 (s, 15H); ^{13}C NMR (100 MHz, $CDCl_3$) δ 167.8, 164.4, 140.3, 132.4, 131.3, 129.2, 128.2, 127.4, 124.5, 121.6, 120.2, 113.0, 89.4, 87.4, 82.4, 79.1, 77.9, 74.9, 72.8, 66.8, 66.0, 54.8, 9.9; HRMS (ESI-TOF) *m/z* calculated for $C_{33}H_{33}N_2O_2Fe$ $[M+H]^+$: 545.1886, found: 545.1882.

2.5 Evaluation of Cu-Mediated Asymmetric C–H Alkynylation of Ferrocenes

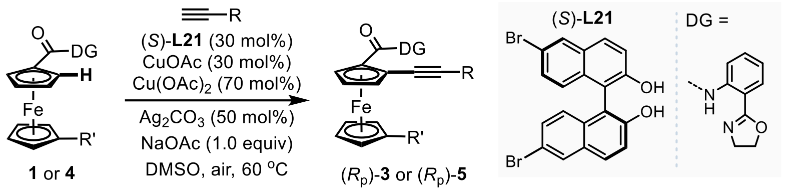

General Procedure for Cu-Mediated Asymmetric C–H Alkynylation of Ferrocenes: A 15 mL scale tube was charged with substrate **1** or **4** (37.4 mg, 0.1 mmol, 1.0 equiv.), $Cu(OAc)_2$ (12.7 mg, 0.07 mmol), CuOAc (3.7 mg, 0.03 mmol), (*S*)-**L21** (13.3 mg, 30 mol%), Ag_2CO_3 (13.8 mg, 0.05 mmol), NaOAc (8.2 mg, 0.1 mmol), **2** (0.25 mmol, 2.5 equiv.), DMSO (5.0 mL) under air atmosphere. The tube was capped tightly, and the reaction mixture was stirred at room temperature for 30 s and then stirred at 60 °C for another 12 h. Upon completion, the reaction was cooled to room temperature, then EtOAc was added to dilute the reaction mixture. The organic layer was washed with $NH_3{\cdot}H_2O$, saturated brine, and dried over Na_2SO_4. Volatiles were removed under vacuum. The crude product was purified by column chromatography to afford the desired product (R_p)-**3** or (R_p)-**5**. The ratio of mono/di was determined by the analysis of crude ^{1}H NMR.

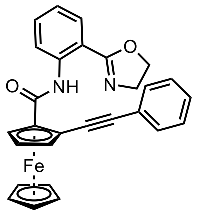

(R_p)-**3a**

(R_p)-*N*-(2-(4,5-Dihydrooxazol-2-yl)phenyl)-2-(phenylethynyl)ferrocenyl-1-carboxamide

68% total yield [(R_p)-**3a**, 92.5:7.5 er, 39% (18.3 mg); **3a'** (di), 29% (16.7 mg)], mono/di = 1.6/1.0. The er value of the mono-alkynylated product (R_p)-**3a** was determined by chiral HPLC analysis: Chiralcel IA column (25 cm); hexane/^{i}PrOH = 92/8; flow rate, 1.0 mL/min; 254 nm, t_R (major) = 13.18 min; t_R (minor) = 16.60 min; $[a]_D^{26}$ = +51.9 (c = 0.5, CH_2Cl_2).

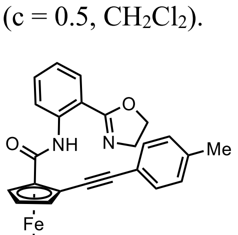

(R_p)-**3b**

(R_p)-*N*-(2-(4,5-Dihydrooxazol-2-yl)phenyl)-2-(p-tolylethynyl)ferrocenyl-1-carboxamide

66% total yield [(R_p)-**3b**, 93:7 er, 36% (17.7 mg); **3b'** (di), 30% (17.8 mg)], mono/di = 1.7/1.0. The er value of the mono-alkynylated product (R_p)-**3b** was determined by chiral HPLC analysis: Chiralcel IA column (25 cm);

hexane/^{i}PrOH = 92/8; flow rate, 1.0 mL/min; 254 nm, t_R (major) = 13.01 min; t_R (minor) = 16.36 min; $[a]_D^{26}$ = +43.7 (c = 0.5, CH_2Cl_2).

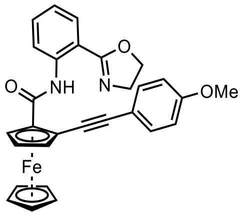

(R_p)-**3d**

(R_p)-*N*-(2-(4,5-Dihydrooxazol-2-yl)phenyl)-2-((4-methoxyphenyl)ethynyl)ferrocenyl-1-carboxamide

60% total yield [(R_p)-**3d**, 90.5:9.5 er, 44% (22.0 mg); **3d'** (di), 16% (9.9 mg)], mono/di = 2.9/1.0. The er value of the mono-alkynylated product (R_p)-**3d** was determined by chiral HPLC analysis: Chiralcel IA column (25 cm); hexane/^{i}PrOH = 92/8; flow rate, 1.0 mL/min; 254 nm, t_R (major) = 20.25 min; t_R (minor) = 27.13 min; $[a]_D^{24}$ = +33.0 (c = 0.5, CH_2Cl_2).

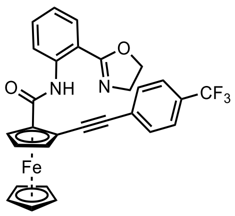

(R_p)-**3e**

(R_p)-*N*-(2-(4,5-Dihydrooxazol-2-yl)phenyl)-2-((4-(trifluoromethyl)phenyl)ethynyl)ferrocenyl-1-carboxamide

58% total yield [(R_p)-**3e**, 92:8 er, 35% (18.8 mg; **3e'** (di), 23% (16.6 mg)], mono/di = 3.4/1.0. The er value of the mono-alkynylated product (R_p)-**3e** was determined by chiral HPLC analysis: Chiralcel IC column (25 cm); hexane/^{i}PrOH = 92/8; flow rate, 1.0 mL/min; 254 nm, t_R (minor) = 22.04 min; t_R (major) = 25.26 min; $[a]_D^{26}$ = +81.0 (c = 0.5, CH_2Cl_2).

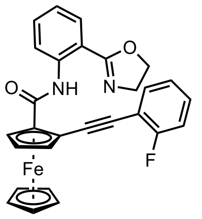

(R_p)-**3f**

(R_p)-*N*-(2-(4,5-Dihydrooxazol-2-yl)phenyl)-2-((2-fluorophenyl)ethynyl)ferrocenyl-1-carboxamide

63% total yield [(R_p)-**3f**, 92:8 er, 40% (19.6 mg); **3f'** (di), 23% (14.2 mg)], mono/di = 2.2/1.0. The er value of the mono-alkynylated product (R_p)-**3f** was determined by chiral HPLC analysis: Chiralcel IA column (25 cm); hexane/^{i}PrOH = 92/8; flow rate, 1.0 mL/min; 254 nm, t_R (major) = 14.34 min; t_R (minor) = 16.66 min; $[a]_D^{27}$ = +98.4 (c = 0.5, CH_2Cl_2).

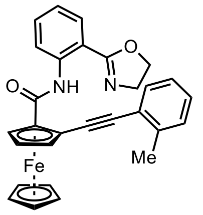

(R_p)-**3g**

(R_p)-*N*-(2-(4,5-Dihydrooxazol-2-yl)phenyl)-2-(o-tolylethynyl)ferrocenyl-1-carboxamide

73% total yield [(R_p)-**3g**, 93:7 er, 38% (18.4 mg); **3g'** (di), 35% (21.1 mg)], mono/di = 1.2/1.0. The er value of the mono-alkynylated product (R_p)-**3g** was 35% (21.1 mg). The er value was determined by chiral HPLC analysis: Chiralcel IA column (25 cm); hexane/^{i}PrOH = 92/8; flow rate, 1.0 mL/min; 254 nm, t_R (major) = 11.56 min; t_R (minor) = 13.14 min; $[a]_D^{26}$ = +53.0 (c = 0.5, CH_2Cl_2).

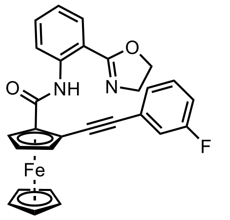

(R_p)-**3h**

(R_p)-*N*-(2-(4,5-Dihydrooxazol-2-yl)phenyl)-2-((3-fluorophenyl)ethynyl)ferrocenyl-1-carboxamide

61% total yield [(R_p)-**3h**, 92:8 er, 46% (22.7 mg); **3h'** (di), 15% (8.9 mg)], mono/di = 3.4/1.0. The er value of the mono-alkynylated product (R_p)-**3h** was determined by chiral HPLC analysis: Chiralcel IA column (25 cm); hexane/^{i}PrOH = 92/8; flow rate, 1.0 mL/min; 254 nm, t_R (major) = 13.21 min; t_R (minor) = 15.64 min; $[a]_D^{27}$ = +92.5 (c = 0.5, CH_2Cl_2).

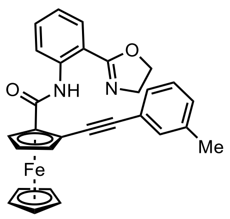

(R_p)-**3i**

(R_p)-*N*-(2-(4,5-Dihydrooxazol-2-yl)phenyl)-2-(m-tolylethynyl)ferrocenyl-1-carboxamide

62% total yield [(R_p)-**3i**, 90:10 er, 50% (24.2 mg); **3i'** (di), 12% (7.0 mg)], mono/di = 4.5/1.0. The er value of the mono-alkynylated product (R_p)-**3i** was determined by chiral HPLC analysis: Chiralcel IA column (25 cm); hexane/^{i}PrOH = 92/8; flow rate, 1.0 mL/min; 254 nm, t_R (major) = 12.09 min; t_R (minor) = 15.40 min; $[a]_D^{25}$ = +49.8 (c = 0.5, CH_2Cl_2).

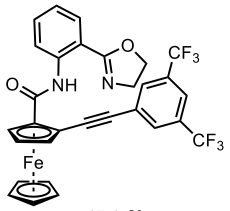

(R_p)-**3j**

(R_p)-2-((3,5-Bis(trifluoromethyl)phenyl)ethynyl)-*N*-(2-(4,5-dihydrooxazol-2-yl)phenyl)ferrocenyl-1-carboxamide

43% total yield [(R_p)-**3j**, 88:12 er, 38% (23.0 mg); **3j'** (di), 5% (4.2 mg)], mono/di = 10/1.0. The er value of the mono-alkynylated product (R_p)-**3j** was determined by chiral HPLC analysis: Chiralcel IA column (25 cm); hexane/^{i}PrOH = 92/8; flow rate, 1.0 mL/min; 254 nm, t_R (minor) = 7.34 min; t_R (major) = 8.09 min; $[a]_D^{25}$ = +73.3 (c = 0.5, CH_2Cl_2).

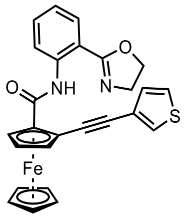

(R_p)-**3l**

(R_p)-*N*-(2-(4,5-Dihydrooxazol-2-yl)phenyl)-2-(thiophen-3-ylethynyl)ferrocenyl-1-carboxamide

82% total yield [(R_p)-**3l**, 89.5:10.5 er, 68% (32.6 mg); **3l'** (di), 14% (8.5 mg)], mono/di = 3.5/1.0. The er value of the mono-alkynylated product (R_p)-**3l** was determined by chiral HPLC analysis: Chiralcel IA column (25 cm); hexane/^{i}PrOH = 92/8; flow rate, 1.0 mL/min; 254 nm, t_R (major) = 16.13 min; t_R (minor) = 21.45 min; $[a]_D^{24}$ = +62.6 (c = 0.5, CH_2Cl_2).

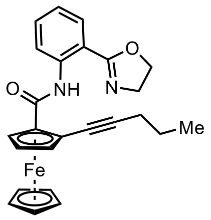

(R_p)-**3q**

(R_p)-*N*-(2-(4,5-Dihydrooxazol-2-yl)phenyl)-2-(pent-1-yn-1-yl)ferrocenyl-1-carboxamide

67% total yield [(R_p)-**3q**, 91:9 er, 51% (22.4 mg); **3q'** (di), 16% (8.2 mg)], mono/di = 1.5/1.0. The er value of the mono-alkynylated product (R_p)-**3q** was determined by chiral HPLC analysis: Chiralcel IA column (25 cm); hexane/^{i}PrOH = 92/8; flow rate, 1.0 mL/min; 254 nm, t_R (major) = 11.29 min; t_R (minor) = 12.45 min; $[a]_D^{30}$ = +202.6 (c = 0.25, CH_2Cl_2).

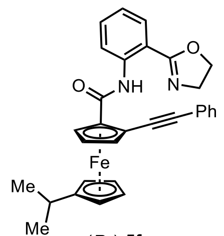

(R_p)-**5f**

(R_p)-*N*-(2-(4,5-Dihydrooxazol-2-yl)phenyl)-1'-isopropyl-2-(phenylethynyl)ferrocenyl-1-carboxamide

73% total yield [(R_p)-**5f**, 91:9 er, 51% (26.1 mg); **5f'** (di), 22% (13.5 mg)], mono/di = 2.5/1.0. The er value of the mono-alkynylated product (R_p)-**5f** was determined by chiral HPLC analysis: Chiralcel IA column (25 cm); hexane/^{i}PrOH = 92/8; flow rate, 1.0 mL/min; 254 nm, t_R (major) = 8.85 min; t_R (minor) = 10.66 min; $[a]_D^{26}$ = +114.4 (c = 0.5, CH_2Cl_2).

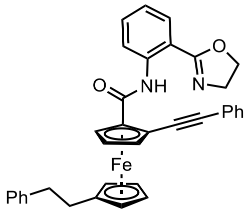

(R_p)-**5g**

(R_p)-*N*-(2-(4,5-dihydrooxazol-2-yl)phenyl)-1'-phenethyl-2-(phenylethynyl)ferrocenyl-1-carboxamide

73% total yield [(R_p)-**5g**, 92.5:7.5 er, 49% (28.5 mg); **5g'** (di), 24% (16.4 mg)], mono/di = 2.0/1.0. The er value of the mono-alkynylated product (R_p)-**5g** was determined by chiral HPLC analysis: Chiralcel IA column (25 cm); hexane/^{i}PrOH=92/8; flow rate, 1.0 mL/min; 254 nm, t_R (major) = 13.55 min; t_R (minor) = 15.66 min; $[a]_D^{25}$ = +122.7 (c = 0.5, CH_2Cl_2).

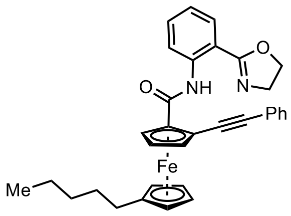

(R_p)-**5h**

(R_p)-*N*-(2-(4,5-dihydrooxazol-2-yl)phenyl)-1'-pentyl-2-(phenylethynyl)ferrocenyl-1-carboxamide

62% total yield [(R_p)-**5h**, 91:9 er, 42% (22.6 mg); **5h'** (di), 20% (13.1 mg)], mono/di = 2.1/1.0. The er value of the mono-alkynylated product (R_p)-**5h** was determined by chiral HPLC analysis: Chiralcel IA column (25 cm); hexane/^{i}PrOH = 92/8; flow rate, 1.0 mL/min; 254 nm, t_R (major) = 10.51 min; t_R (minor) = 12.44 min; $[a]_D^{24}$ = +129.1 (c = 0.5, CH_2Cl_2).

2.6 Gram-Scale Reaction

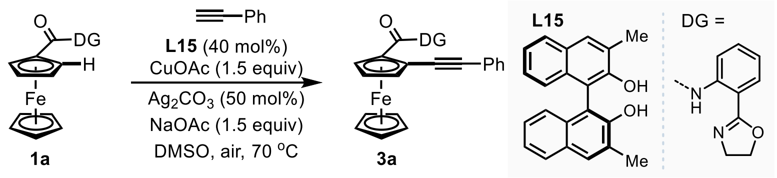

A 500 mL round-bottom flask was charged with substrate **1a** (1.0 g, 2.70 mmol, 1.0 equiv.), CuOAc (496 mg, 4.05 mmol), **L15** (339 mg, 1.08 mmol), Ag_2CO_3 (372 mg, 1.35 mmol), NaOAc (221 mg, 2.70 mmol), following by the addition of DMSO (135 mL), phenylacetylene (689 mg, 0.74 mL, 6.75 mmol) at room temperature under air. The reaction mixture was stirred for 30 s at room temperature and then stirred at 70 °C for another12 h. Upon completion, the reaction was cooled to room temperature, and EtOAc was added to dilute the reaction mixture. The organic layer was washed with $NH_3{\cdot}H_2O$, saturated brine, and dried over Na_2SO_4. Volatiles were removed under vacuum. The crude product was purified by column chromatography to afford the desired product **3a** (818 mg, 64% yield) and **3a'** (180 mg, 12% yield).

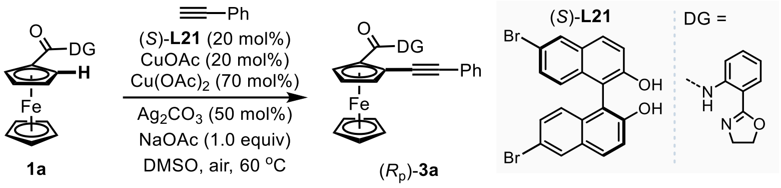

A 500 mL round-bottom flask was charged with substrate **1a** (1.0 g, 2.70 mmol, 1.0 equiv.), CuOAc (62.2 mg, 0.54 mmol, 20 mol%), (*S*)-**L21** (239.8 mg, 0.54 mmol 20 mol%), $Cu(OAc)_2$ (343 mg, 1.89 mmol), Ag_2CO_3 (372 mg, 1.35 mmol), NaOAc (221 mg, 2.70 mmol), following by the addition of DMSO (135 mL), phenylacetylene (689 mg, 0.74 mL, 6.75 mmol) at room temperature under air. The reaction mixture was stirred for 30 s at room temperature and then stirred at 70 °C for another12 h. Upon completion, the reaction was cooled to room temperature, and EtOAc was added to dilute the reaction mixture. The organic layer was washed with $NH_3{\cdot}H_2O$, saturated brine, and dried over Na_2SO_4. Volatiles were removed under vacuum. The crude product was purified by column chromatography to afford the desired product (R_p)-**3a** (661.6 mg, 52% yield, 90:10 er) and **3a'** (115.8 mg, 7% yield). Then (R_p)-**3a** (483.6 mg, 38%, 95.5:0.5 er) was obtained by recrystallization (Hexane/DCM).

2.7 Synthetic Applications of (R_p)-3a

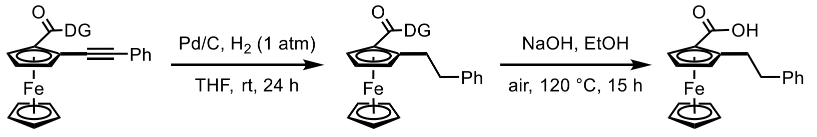

(R_p)-**3a**, 0.1 mmol 99.5:0.5 er | (R_p)-**6a** | (R_p)-**7a**, 65% yield 98:2 er

To a 25 mL round-bottom flask were added (*R*p)-**3a** (47.4 mg, 0.1 mmol), Pd/C (10 wt. % loading on carbon, 10.6 mg), THF (2.0 mL). The mixture was sonicated for 3 minutes. Then the reaction flask was back-filled with H_2 (5 times), and stirred at room temperature for 24 h. Upon completion (monitored by TLC), the mixture was filtered through a short pad of celite, washed with EtOAc, and concentrated under vacuum to provide the crude product (*R*p)-**6a**.

(*R*p)-**6a**, NaOH (240 mg, 6.0 mmol), EtOH (2.0 mL) were added to a 15 mL scaled tube under air atmosphere. The tube was capped tightly, and the mixture was stirred at 120 °C for 15 hours. Upon completion (monitored by TLC), the reaction mixture was evaporated to remove solvent under reduced pressure. Then the mixture was acidified with HCl (30 mL, 1.0 M) to adjust the pH ~ 2, and extracted with DCM (3 x 30 mL). The combined organic phases were dried over Na_2SO_4, and concentrated under vacuum. The product was purified by preparative thin-layer chromatography (PE/EA = 5/1) to give the desired product (R_p)-**7a** as orange solid (21.6 mg, 65%). ^{1}H NMR (400 MHz, $CDCl_3$) δ 7.29 (t, *J* = 7.4 Hz, 2H), 7.25–7.17 (m, 3H), 4.85 (t, *J* = 2.2 Hz, 1H), 4.33 (d, *J* = 2.0 Hz, 2H), 4.19 (s, 5H), 3.34–3.23 (m, 1H), 3.00–2.89 (m, 1H), 2.87–2.67 (m, 2H); ^{13}C NMR (100 MHz, $CDCl_3$) δ 179.2, 142.1, 128.5, 128.3, 125.8, 91.5, 73.3, 71.3, 70.6, 69.8, 67.5, 37.7, 31.4; HRMS (ESI-TOF) *m/z* calculated for $C_{19}H_{17}O_2Fe$ $[M–H]^-$: 333.0583, found: 333.0586; HPLC, Chiralcel OJ-H column (hexane/isopropanol = 90/10, 0.1 mL/min, 254 nm), t_R = 71.71 min (minor), 85.35 min (major): 98:2 er; $[a]_D^{25}$ = +32.5 (c = 0.25, $CHCl_3$).

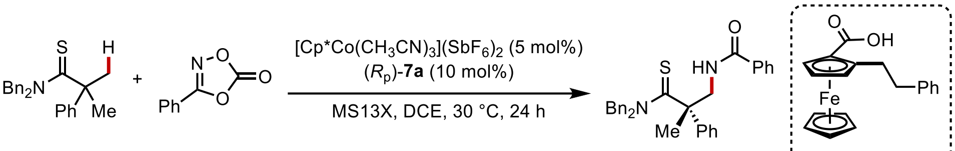

8 | **9** | **10**, 95% yield 70.5 : 29.5 er | (R_p)-**7a** 98:2 er

To a 10 mL scale tube were added **8** (35.9 mg, 0.1 mmol), **9** (19.6 mg, 0.12 mmol), chiral ferrocene carboxylic acid (R_p)-**7a** (3.3 mg, 10 mol%), and $[Cp^*Co(MeCN)_3][SbF_6]_2$ (3.9 mg, 5.0 mol%), activated MS 13X (20 mg) was added in nitrogen glovebox. The vial was capped and brought outside of the glovebox. The reaction flask was back-filled with argon (5 times), and DCE (1.0 mL) was injected. The reaction mixture was stirred for 24 h at 30 °C. Upon completion, the mixture was filtrated through a short pad of silica gel (PE/EA = 1/1). After evaporation, the crude mixture was purified by preparative thin-layer chromatography (PE/EA = 10/1) to afford **10** as white solid. ^{1}H NMR (400 MHz, $CDCl_3$) δ 8.11 (t, *J* = 6.8 Hz, 1H), 7.81 (d, *J* = 7.6 Hz, 2H), 7.54–7.42 (m, 3H), 7.38–7.16 (m, 13H), 6.85 (d, *J* = 7.6 Hz, 2H), 5.93 (d, *J* = 14.6 Hz, 1H), 4.78–4.65 (m, 2H), 4.11 (d, *J* = 16.4 Hz, 1H), 4.02 (d, *J* = 6.6 Hz, 2H), 1.81 (s, 3H). This data is consistent with literature reports[10]. HPLC, Chiralcel IF column (hexane/isopropanol = 90/10, 1.0 mL/min, 224 nm), t_R = 28.76 min (minor), 37.03 min (major): 70.5:29.5 er; $[\alpha]_D^{26}$: + 3.4 (c = 0.84, $CHCl_3$).

2.8 Mechanistic Experiments

2.8.1 Kinetic Isotope Effect Experiment

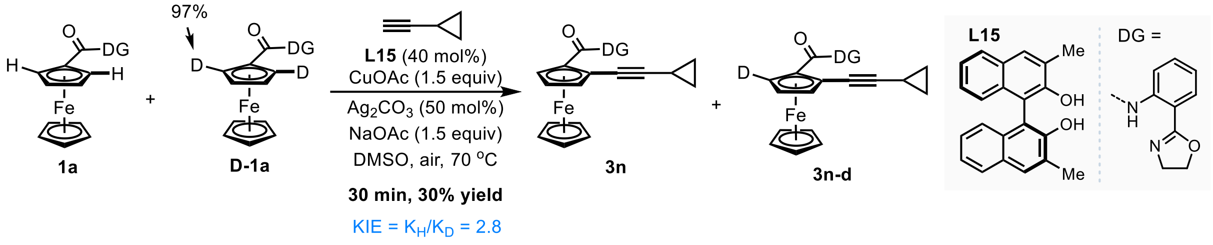

Substrate **1a** (18.7 mg, 0.05 mmol), **D-1a** (18.7 mg, 0.05 mmol), CuOAc (18.4 mg, 0.15 mmol), **L15** (12.6 mg, 40 mol%), Ag_2CO_3 (13.8 mg, 0.05 mmol), NaOAc (8.2 mg, 0.1 mmol) were charged in 15 mL scale tube, followed by the addition of DMSO (5.0 mL), Cyclopropane (21.2 μL, 0.25 mmol) under air. The tube was capped tightly. Then the reaction mixture stirred for 30 s at room temperature and placed into a pre-heated oil bath for 30 min at 70 °C. After cooled to room temperature, EtOAc was added to dilute the reaction, and organic layer was washed with $NH_3{\cdot}H_2O$, saturated brine, and dried over Na_2SO_4. Volatiles were removed under vacuum. The crude product was purified by preparative thin-layer chromatography (PE/EA/DCM = 40/2/3) to afford the desired product **3n** and **3n-d**. The distribution of **3n** and **3n-d** was calculated from the integrals of their respective ^{1}H NMR signals at 4.87 ppm (**3n** and **3n-d**) (see Supplementary Fig. 1).

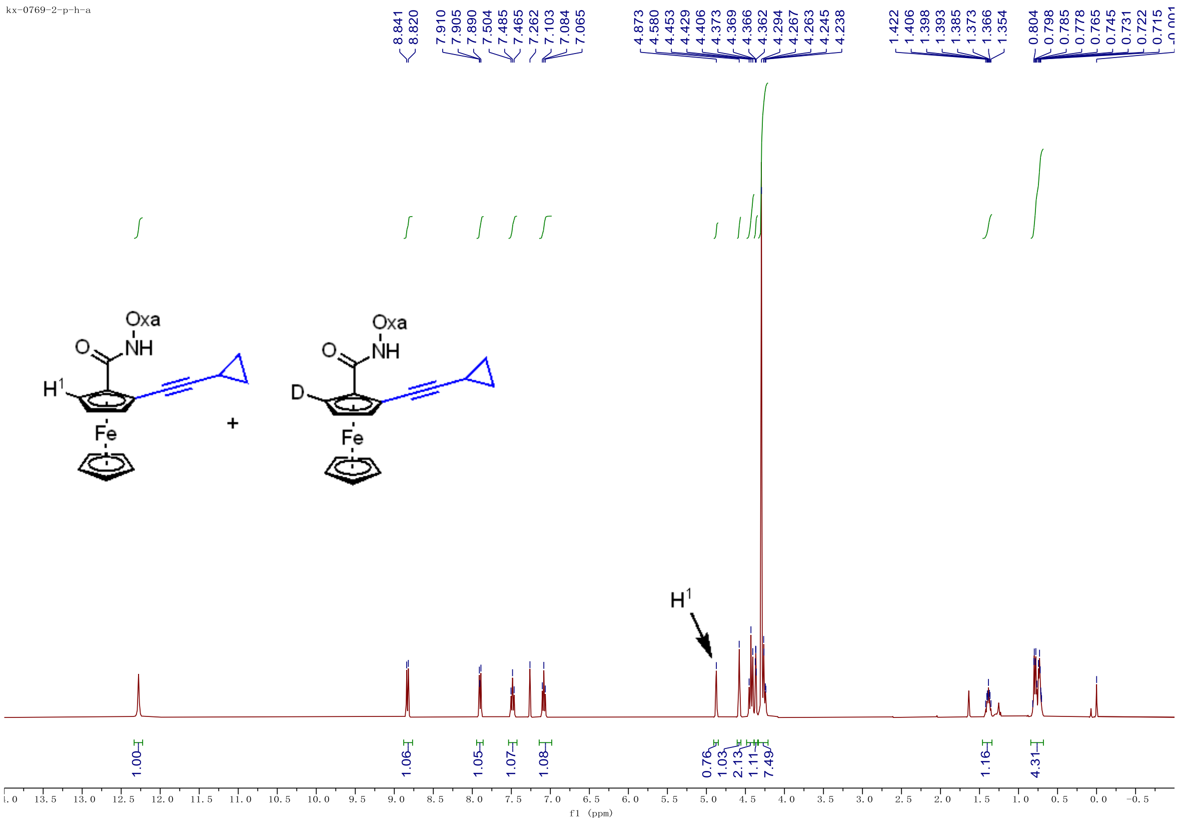

Supplementary Fig. 1. The ^{1}H NMR spectrum of the product mixture obtained in the intermolecular KIE experiment with **L15**.

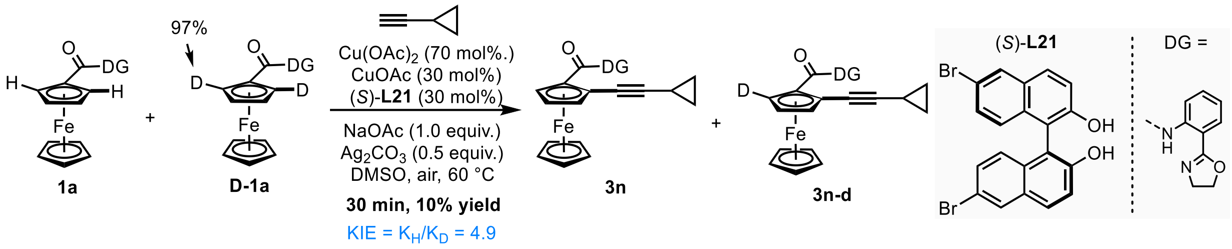

Substrate **1a** (18.7 mg, 0.05 mmol), **D-1a** (18.8 mg, 0.05 mmol), CuOAc (3.7 mg, 0.03 mmol), $Cu(OAc)_2$ (12.7 mg, 0.07 mmol), (*S*)-**L21** (13.3 mg, 30 mol%), Ag_2CO_3 (13.8 mg, 0.05 mmol), NaOAc (8.2 mg, 0.1 mmol) were charged in 15 mL scale tube, followed by the addition of DMSO (5.0 mL), Cyclopropane (21.2 μL, 0.25 mmol) under air. The tube was capped tightly. Then the reaction mixture stirred for 30 s at room temperature and placed into a pre-heated oil bath for 30 min at 60 °C. After cooled to room temperature, EtOAc was added to dilute the reaction, and organic layer was washed with $NH_3·H_2O$, saturated brine, and dried over Na_2SO_4. Volatiles were removed under vacuum. The crude product was purified by preparative thin-layer chromatography (PE/EA/DCM = 40/2/3) to afford the desired product **3n** and **3n-d**. The distribution of **3n** and **3n-d** was calculated from the integrals of their respective ^{1}H NMR signals at 4.87 ppm (**3n** and **3n-d**) (see Supplementary Fig. 2).

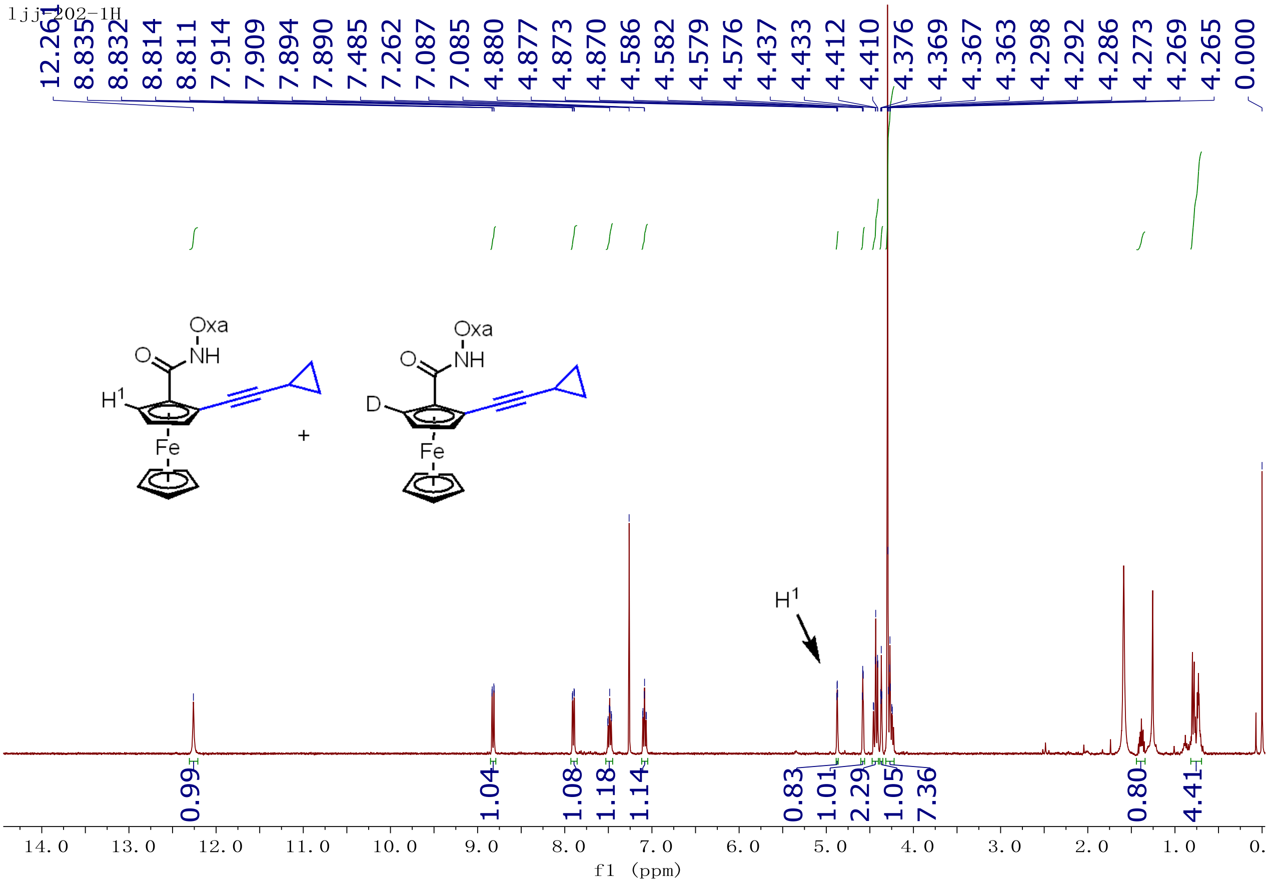

Supplementary Fig. 2. The ^{1}H NMR spectrum of the product mixture obtained in the intermolecular KIE experiment with (*S*)-**L21**.

2.8.2 Kinetics data of phenylacetylene substrate with or without ligand

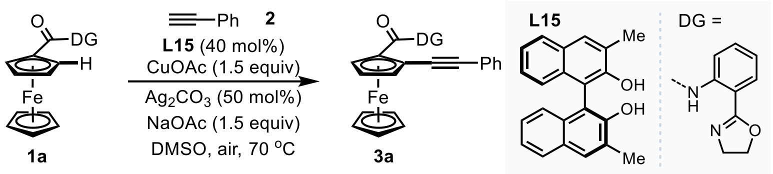

A 15 mL scale tube was charged with substrate **1a** (37.4 mg, 0.1 mmol, 1.0 equiv.), CuOAc (18.4 mg, 0.15 mmol), **L15** (12.6 mg, 40 mol%) or without **L15**, Ag_2CO_3 (13.8 mg, 0.05 mmol), NaOAc (8.2 mg, 0.1 mmol), **2** (27.5 µL, 0.25 mmol, 2.5 equiv.), DMSO (5.0 mL) under air atmosphere. The tube was capped tightly, and the mixture was stirred at room temperature for 30 s then stirred at 70 °C. Then immediately quenched with EA. The organic layer was washed with $NH_3{\cdot}H_2O$, saturated brine, and dried over Na_2SO_4. Volatiles were removed under vacuum. The yield was determined by ^{1}H NMR using 1,1,2,2-tetrachloroethane as the internal standard. The obtained average yields for three trials were plotted as yield [3a] vs. time t (Supplementary Fig. 3).

t (min)	Yield of **3a** with **L15** (%)				Standard	Yield of **3a** without **L15** (%)				Standard
	Trial 1	Trial 2	Trial 3	Average	Deviation	Trial 1	Trial 2	Trial 3	Average	Deviation
3	5.0	4.0	5.0	4.7	0.58	0.0	1.0	1.0	0.7	0.58
5	9.0	7.0	9.5	8.5	1.32	1.0	1.0	1.0	1.0	0.00
10	16.0	13.5	15.5	15.0	1.32	1.0	2.0	2.0	1.7	0.58
15	20.0	18.5	20.0	19.5	0.87	3.0	3.0	4.0	3.3	0.58

Supplementary Table 18. Initial rate kinetic data for the racemic transformation with and without ligand **L15**.

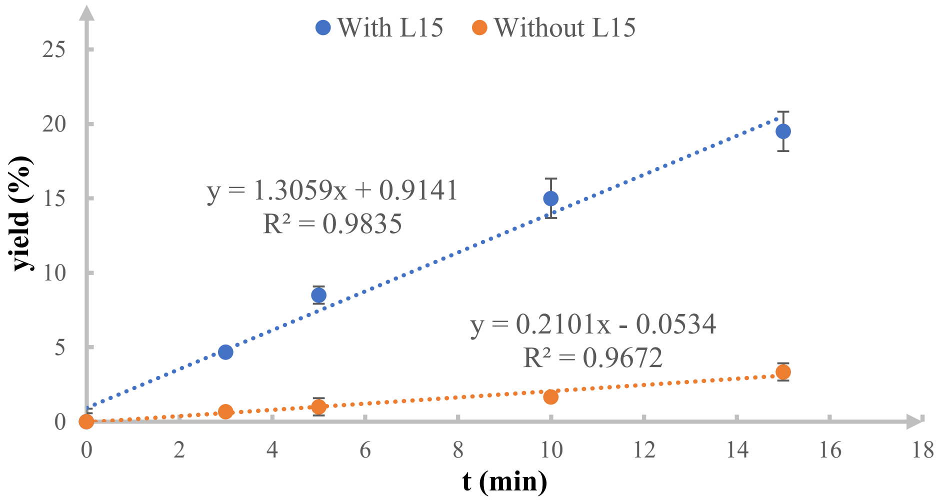

Supplementary Fig. 3. Initial reaction rate plot for the racemic reaction with or without ligand **L15**.

The date shows the ligand plays a crucial role in this reaction. The initial ratio refers to relative rates of the reaction with ligand vs without ([k with **L15**]/[k without **L15**] = 6.2, Supplementary Fig. 3).

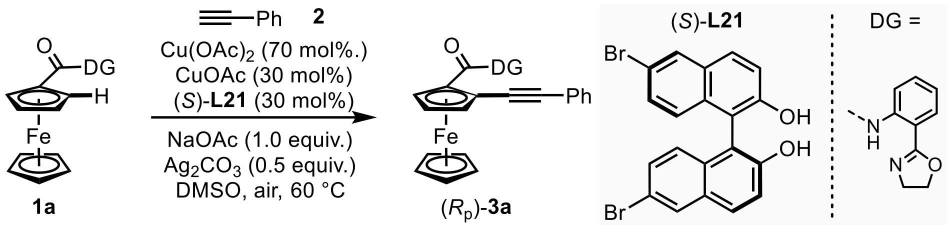

A 15 mL scale tube was charged with substrate **1a** (37.4 mg, 0.1 mmol, 1.0 equiv.), CuOAc (3.7 mg, 0.03 mmol), $Cu(OAc)_2$ (12.7 mg, 0.07 mmol), (*S*)-**L21** (13.3 mg, 30 mol%) or without (*S*)-**L21**, Ag_2CO_3 (13.8 mg, 0.05 mmol), NaOAc (8.2 mg, 0.1 mmol), **2** (27.5 μL, 0.25 mmol, 2.5 equiv.), DMSO (5.0 mL) under air atmosphere. The tube was capped tightly, and the mixture was stirred at room temperature for 30 s then stirred at 60 °C. Then immediately quenched with EA. The organic layer was washed with $NH_3·H_2O$, saturated brine, and dried over Na_2SO_4. Volatiles were removed under vacuum. The yield was determined by ^{1}H NMR using 1,1,2,2-tetrachloroethane as the internal standard. The obtained average yields for three trials were plotted as yield [(R_p)-3a] vs. time t (Supplementary Fig. 4).

t (min)	Yield of **3a** with (*S*)-**L21** (%)				Standard Deviation	Yield of **3a** without 9**L21** (%)				Standard Deviation
	Trial 1	Trial 2	Trial 3	Average		Trial 1	Trial 2	Trial 3	Average	
15	5.0	7.0	6.0	6.0	1.0	1.0	0.0	1.0	0.67	0.58
30	8.0	9.0	10.0	9.0	1.0	1.0	1.0	1.0	1.0	0.00
60	16.0	15.0	16.0	15.67	0.58	1.0	2.0	2.0	1.67	0.58
90	27.0	27.0	28.0	27.33	0.58	2.0	2.0	3.0	2.33	0.58

Supplementary Table 19. Initial rate kinetic data for the asymmetric transformation with or without ligand (*S*)-**L21**.

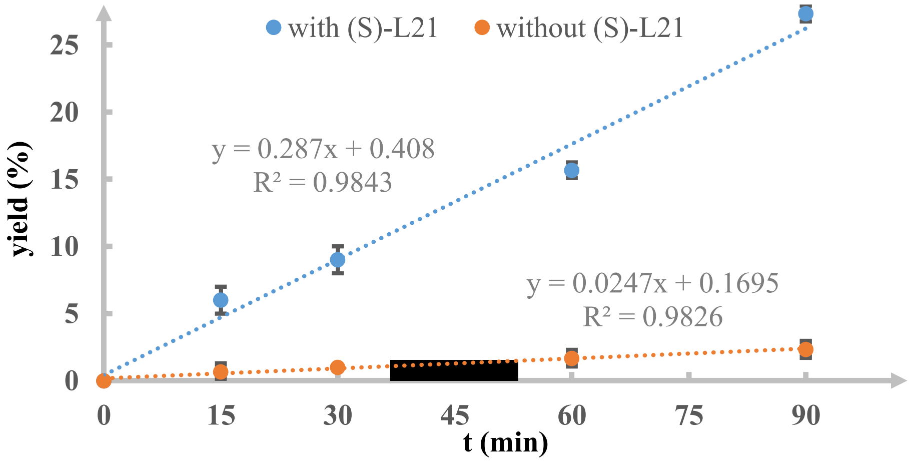

Supplementary Fig. 4. Initial reaction rate plot for the reaction with or without ligand (*S*)-**L21**.

The date shows the ligand plays a crucial role in this reaction. The initial ratio refers to relative rates of the reaction with ligand vs without ([k with (*S*)-**L21**]/[k without (*S*)-**L21**] = 11.6 Supplementary Fig. 4).

3. X-Ray Structure of (R_p)-3a

Single crystals of (R_p)-**3a** were obtained by the recrystallization from PE/ether. The molecular structure and X-ray diffraction data/refinement of (R_p)-**3a** were shown below.

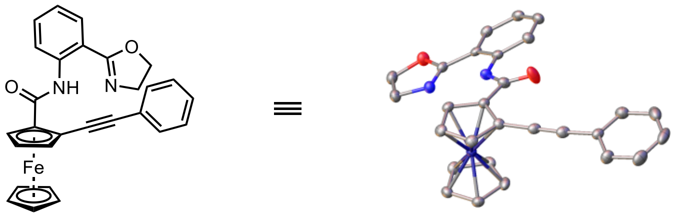

(R_p)-**3a**

Bond precision:	C-C = 0.0081 A		Wavelength=1.34139
Cell:	a=12.1155(5)	b=12.7907(5)	c=15.5853(6)
	alpha=90	beta=112.720(2)	gamma=90

Temperature:213 K

	Calculated	Reported
Volume	2227.78(16)	2227.78(16)
Space group	P 21	P 1 21 1
Hall group	P 2yb	P 2yb
Moiety formula	C28 H22 Fe N2 O2	C28 H22 Fe N2 O2
Sum formula	C28 H22 Fe N2 O2	C28 H21 Fe N2 O2
Mr	474.33	474.33
Dx,g cm-3	1.414	1.411
Z	4	4
Mu (mm-1)	3.891	3.891
F000	984.0	984.0
F000'	984.85	
h,k,lmax	14,15,19	14,15,19
Nref	8551[4479]	7990
Tmin,Tmax	0.800,0.823	0.455,0.751
Tmin'	0.725	

Correction method= # Reported T Limits: Tmin=0.455 Tmax=0.751

Data completeness= 1.78/0.93 Theta(max)= 55.075

R(reflections)= 0.0418(6528) wR2(reflections)= 0.0982(7990)

S = 1.000 Npar= 596

4. NMR Spectra

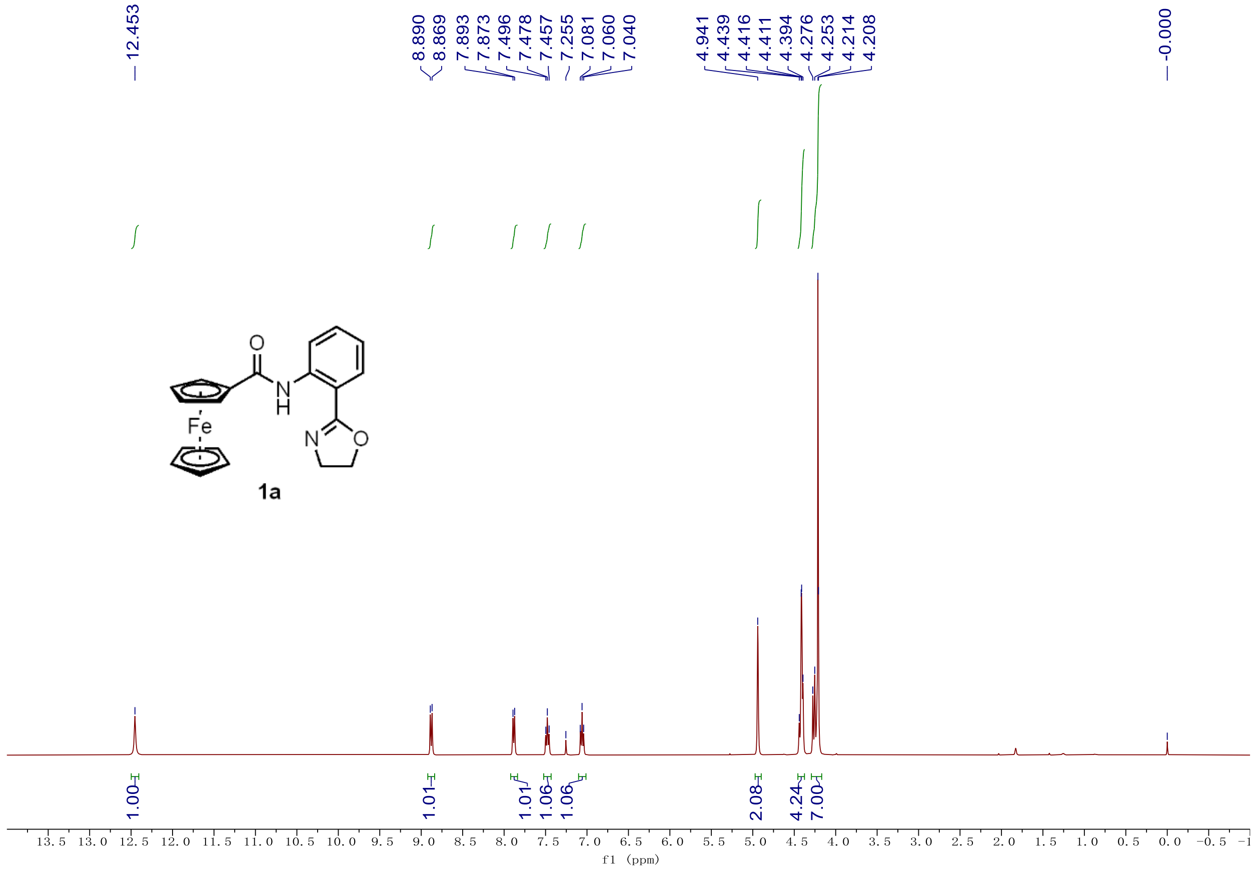

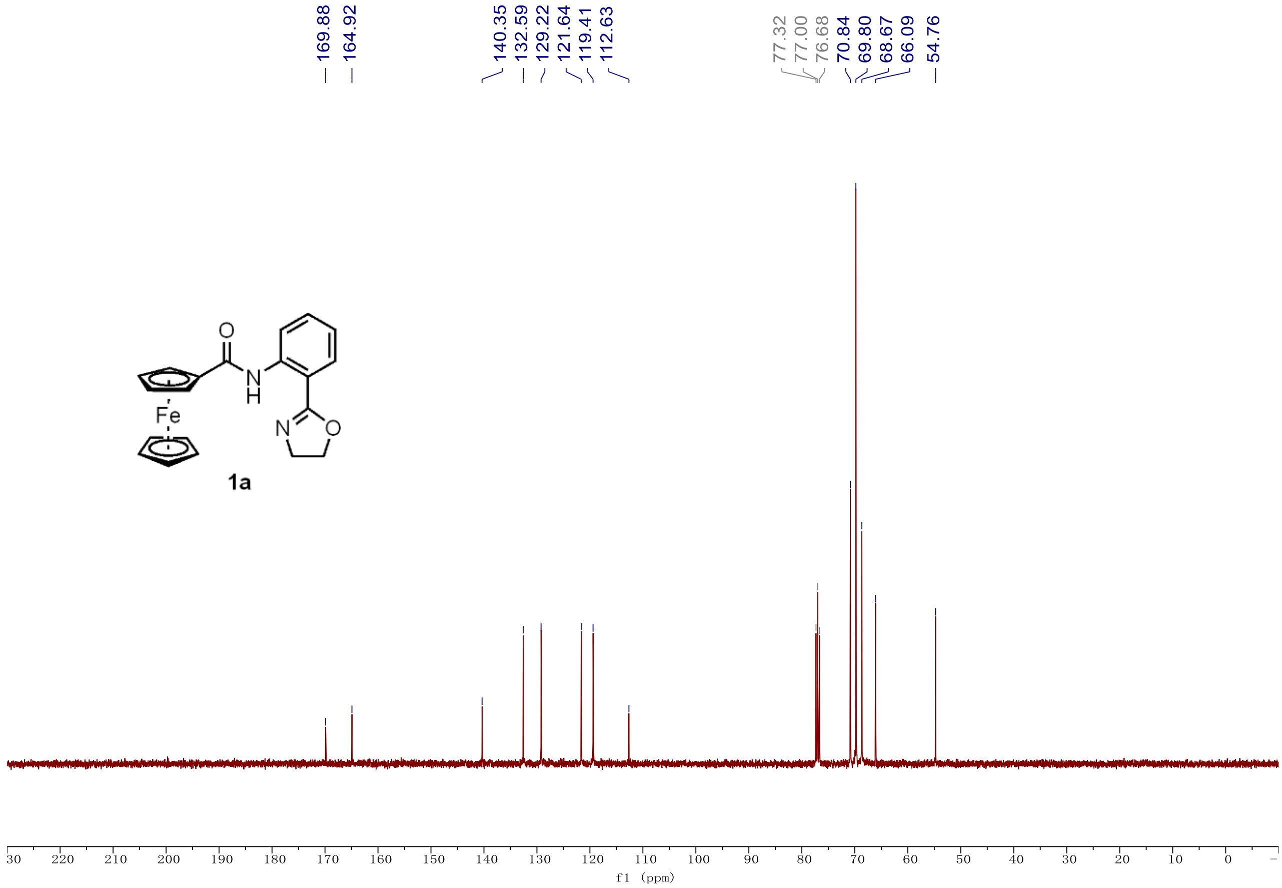

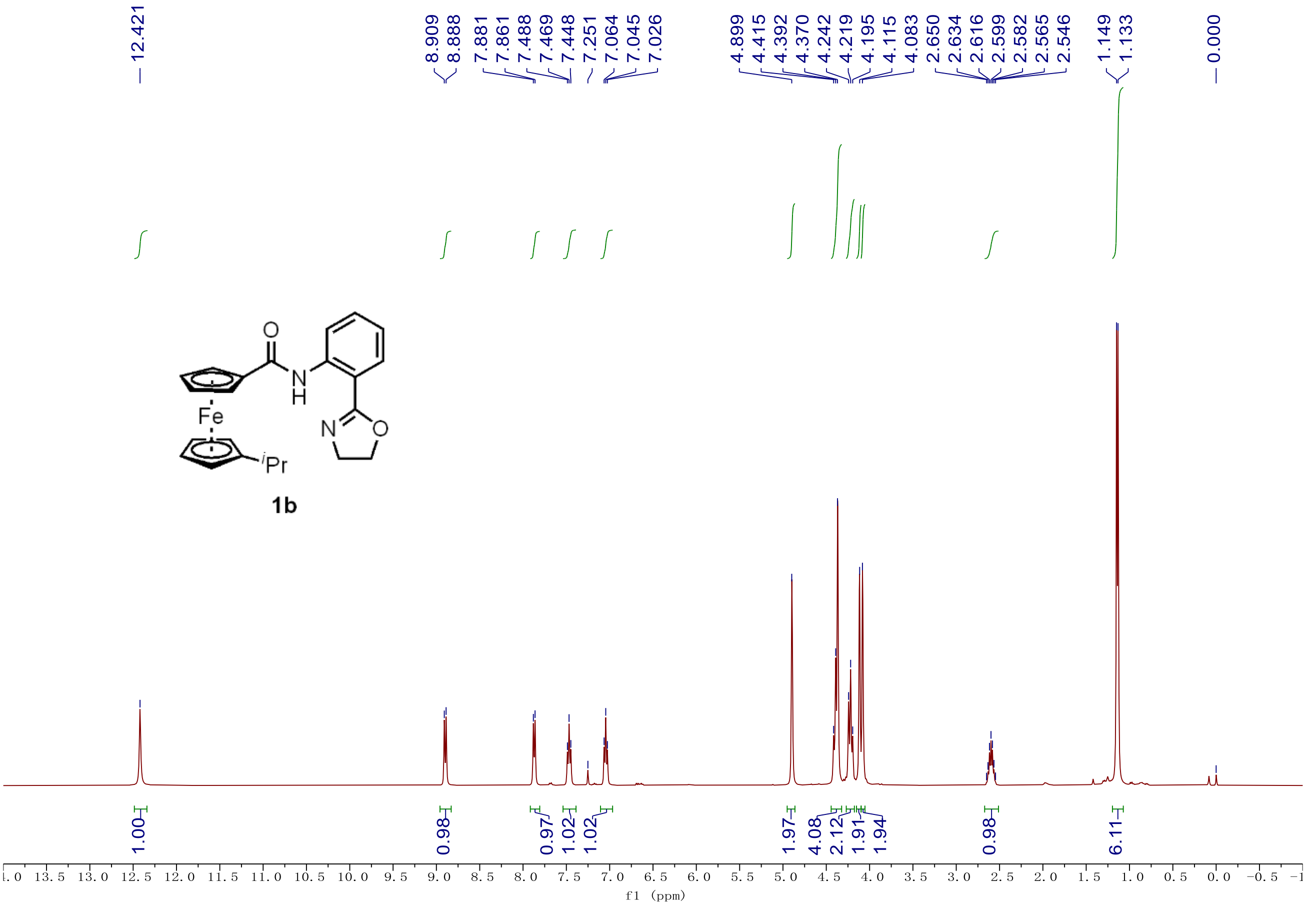
12.421
8.909
8.888
7.881
7.861
7.488
7.469
7.448
7.251
7.064
7.045
7.026
4.899
4.415
4.392
4.370
4.242
4.219
4.195
4.115
4.083
2.650
2.634
2.616
2.599
2.582
2.565
2.546
1.149
1.133
0.000
O
N
H
Fe
N
O
iPr
1b
1.00
0.98
0.97
1.02
1.02
1.97
4.08
2.12
1.91
1.94
0.98
6.11
f1 (ppm)

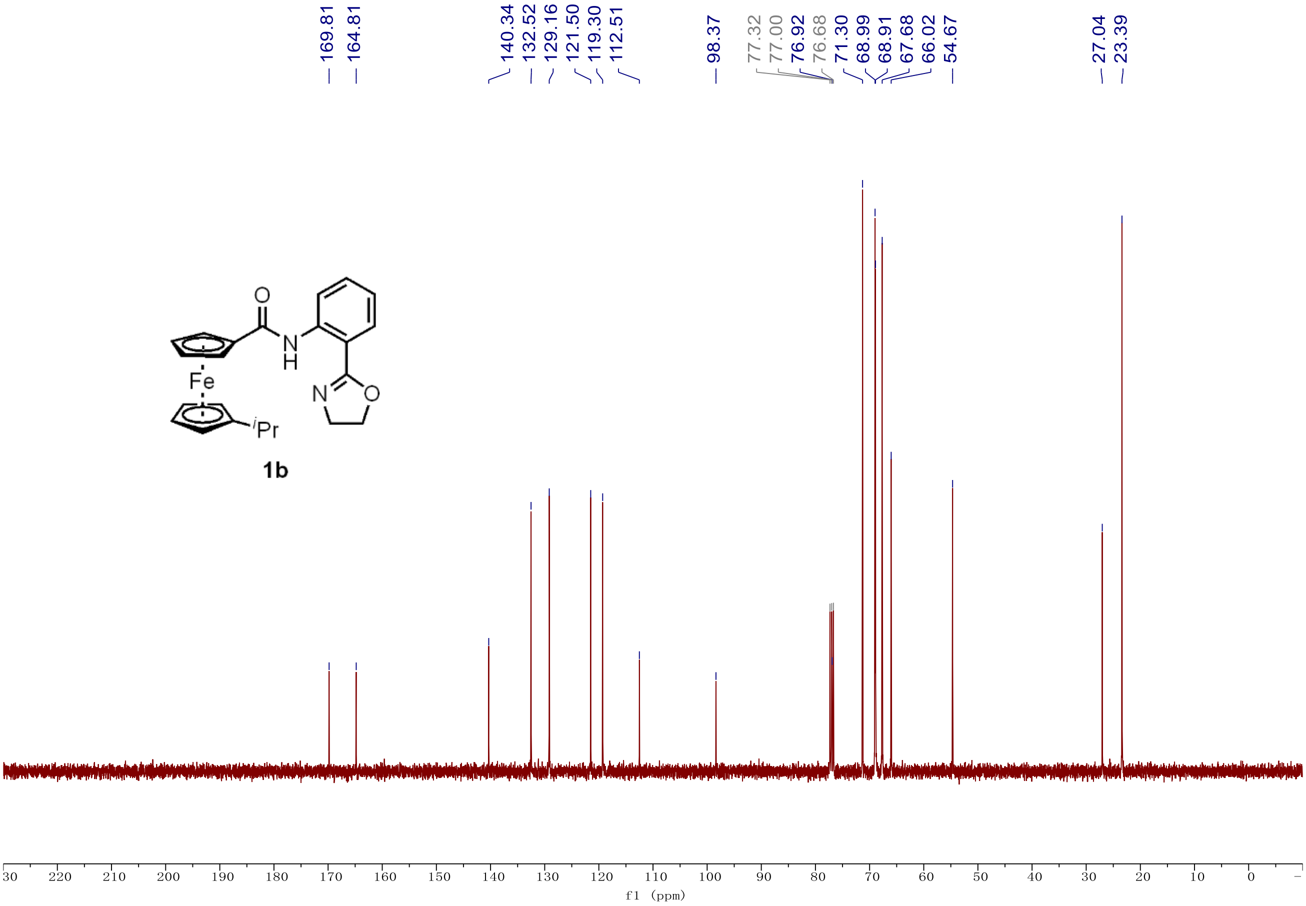
169.81
164.81
140.34
132.52
129.16
121.50
119.30
112.51
98.37
77.32
77.00
76.92
76.68
71.30
68.99
68.91
67.68
66.02
54.67
27.04
23.39
O
N
H
Fe
N
O
iPr
1b
f1 (ppm)

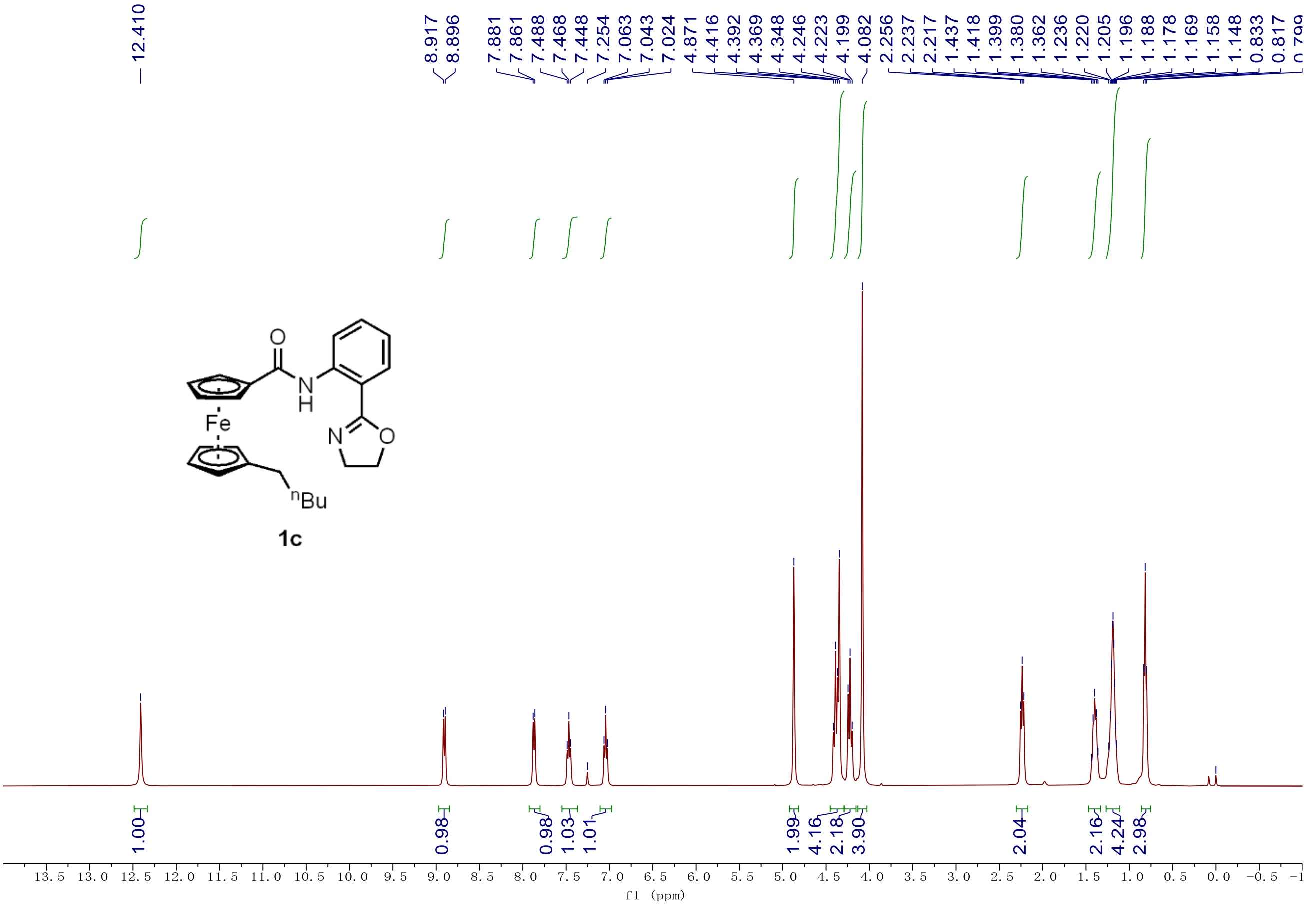

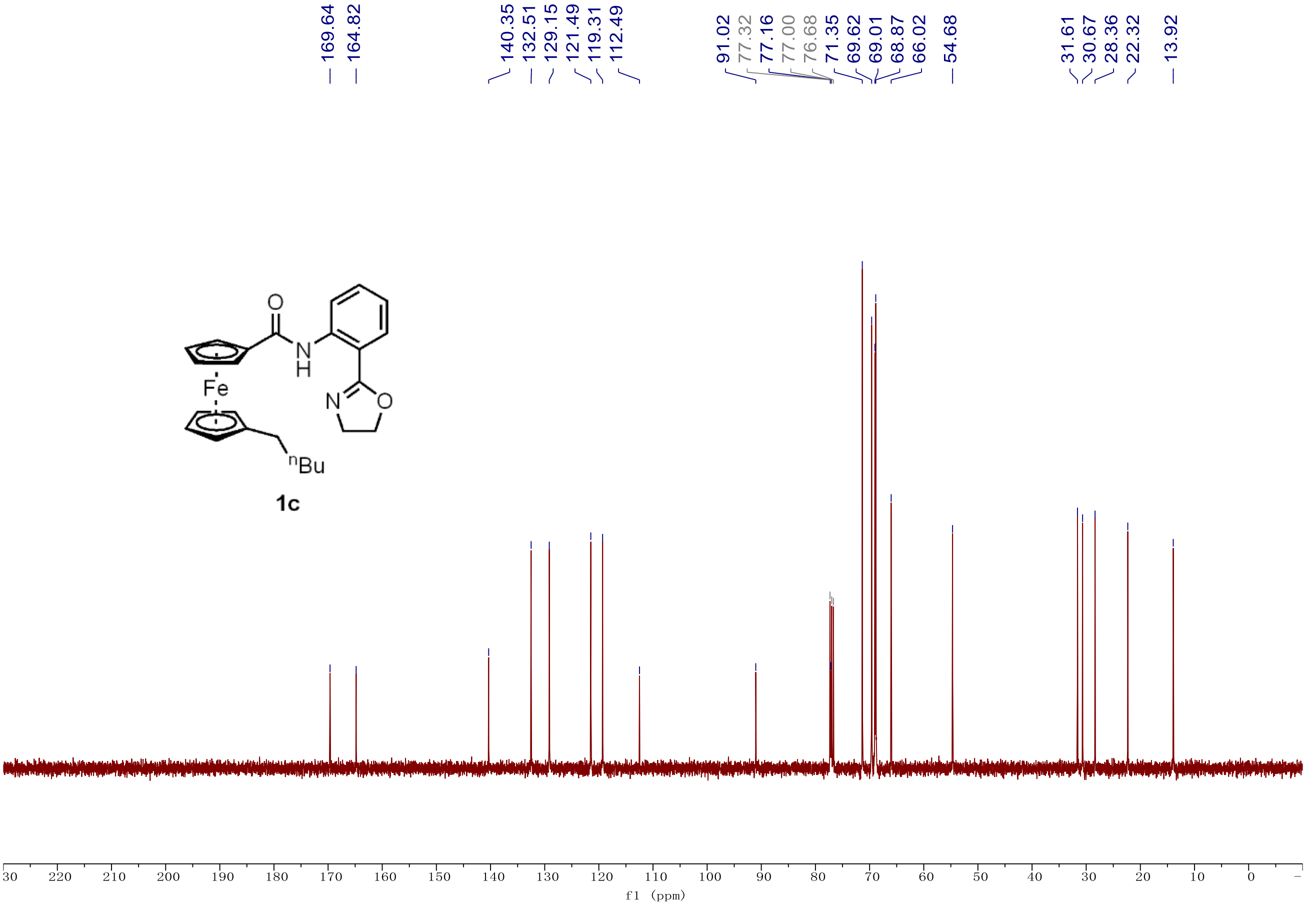

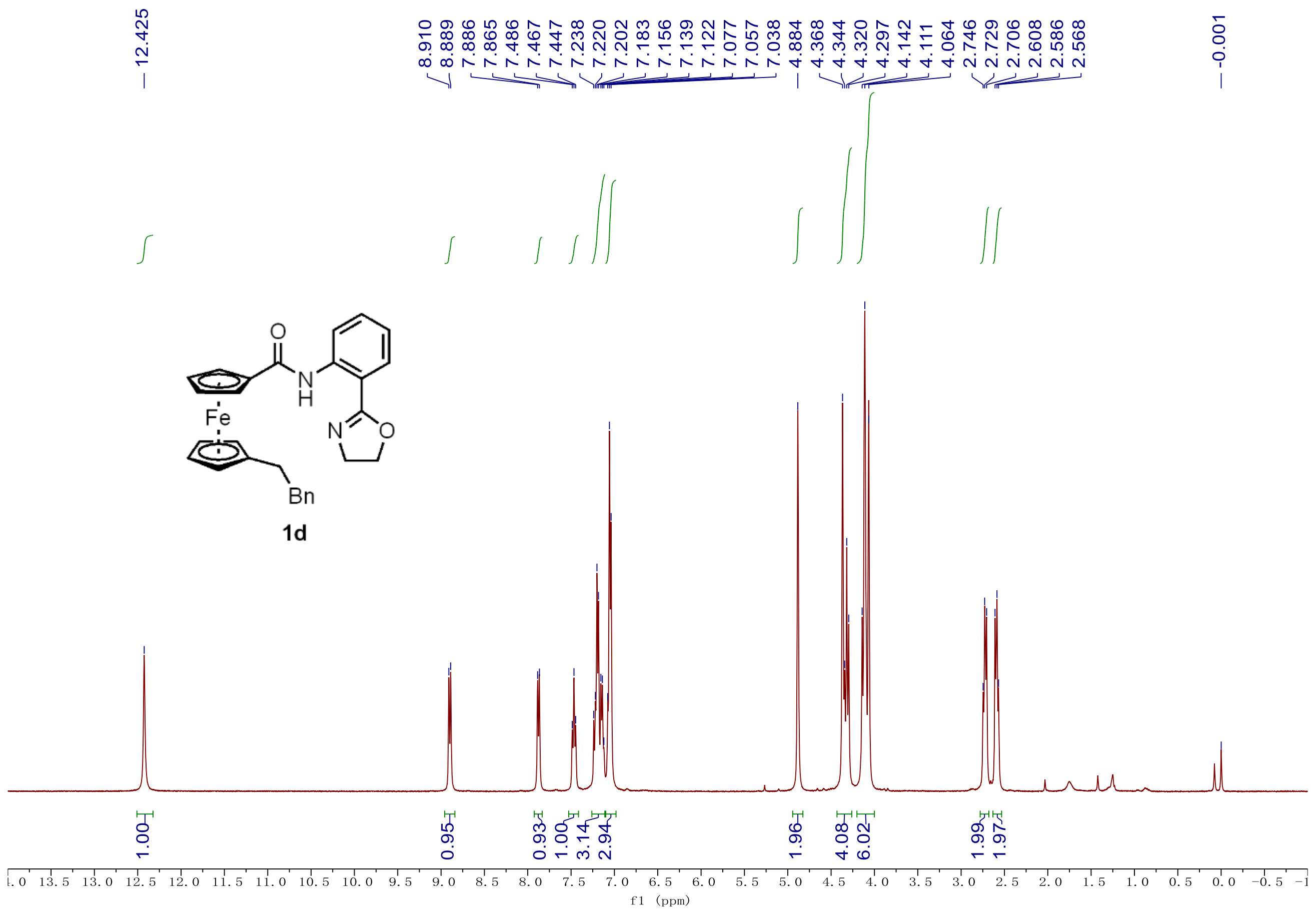
12.425
8.910
8.889
7.886
7.865
7.486
7.467
7.447
7.238
7.220
7.202
7.183
7.156
7.139
7.122
7.077
7.057
7.038
4.884
4.368
4.344
4.320
4.297
4.142
4.111
4.064
2.746
2.729
2.706
2.608
2.586
2.568
-0.001
O
N
H
Fe
N
O
Bn
1d
1.00
0.95
0.93
1.00
3.14
2.94
1.96
4.08
6.02
1.99
1.97
f1 (ppm)

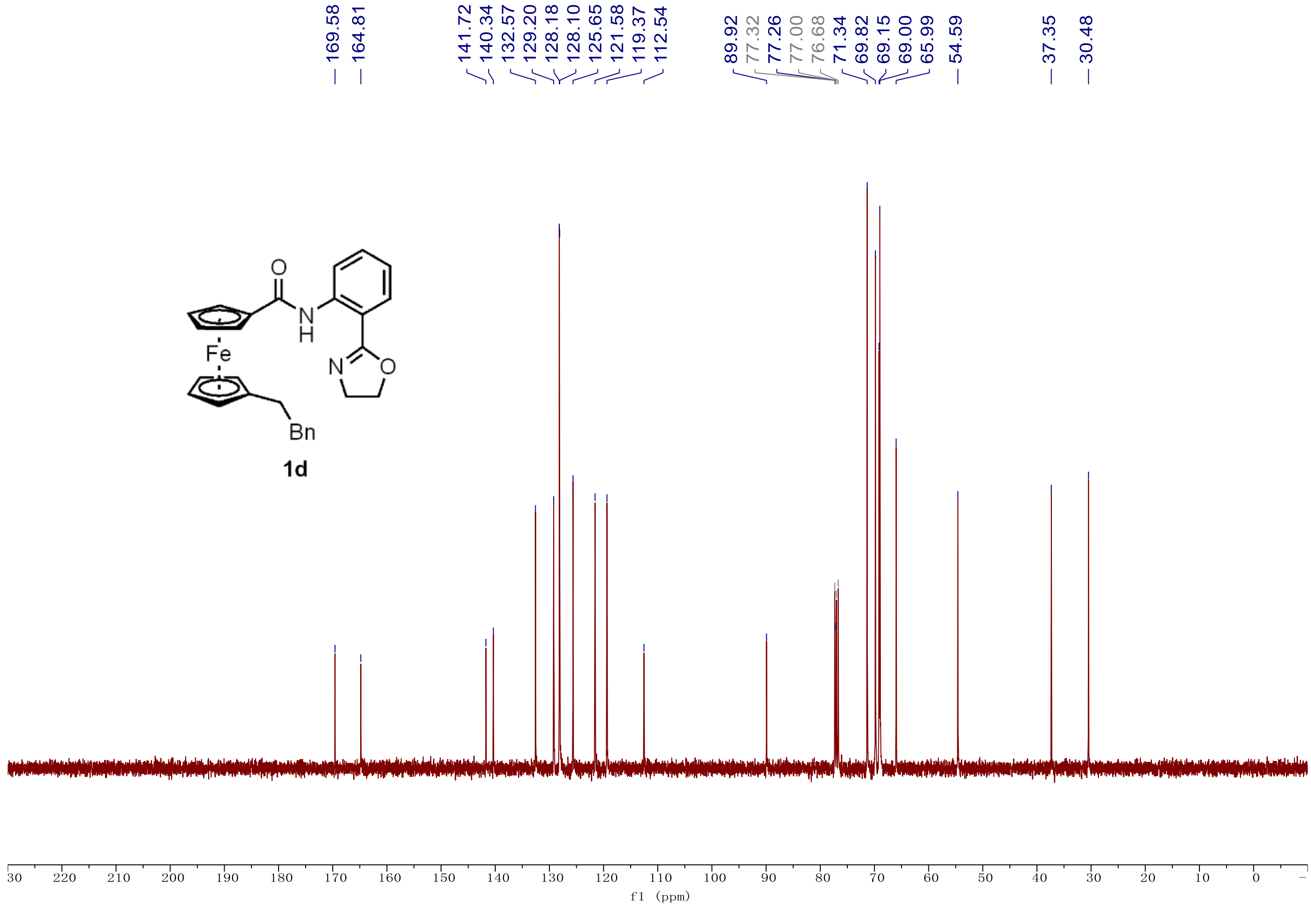
169.58
164.81
141.72
140.34
132.57
129.20
128.18
128.10
125.65
121.58
119.37
112.54
89.92
77.32
77.26
77.00
76.68
71.34
69.82
69.15
69.00
65.99
54.59
37.35
30.48
O
N
H
Fe
N
O
Bn
1d
f1 (ppm)

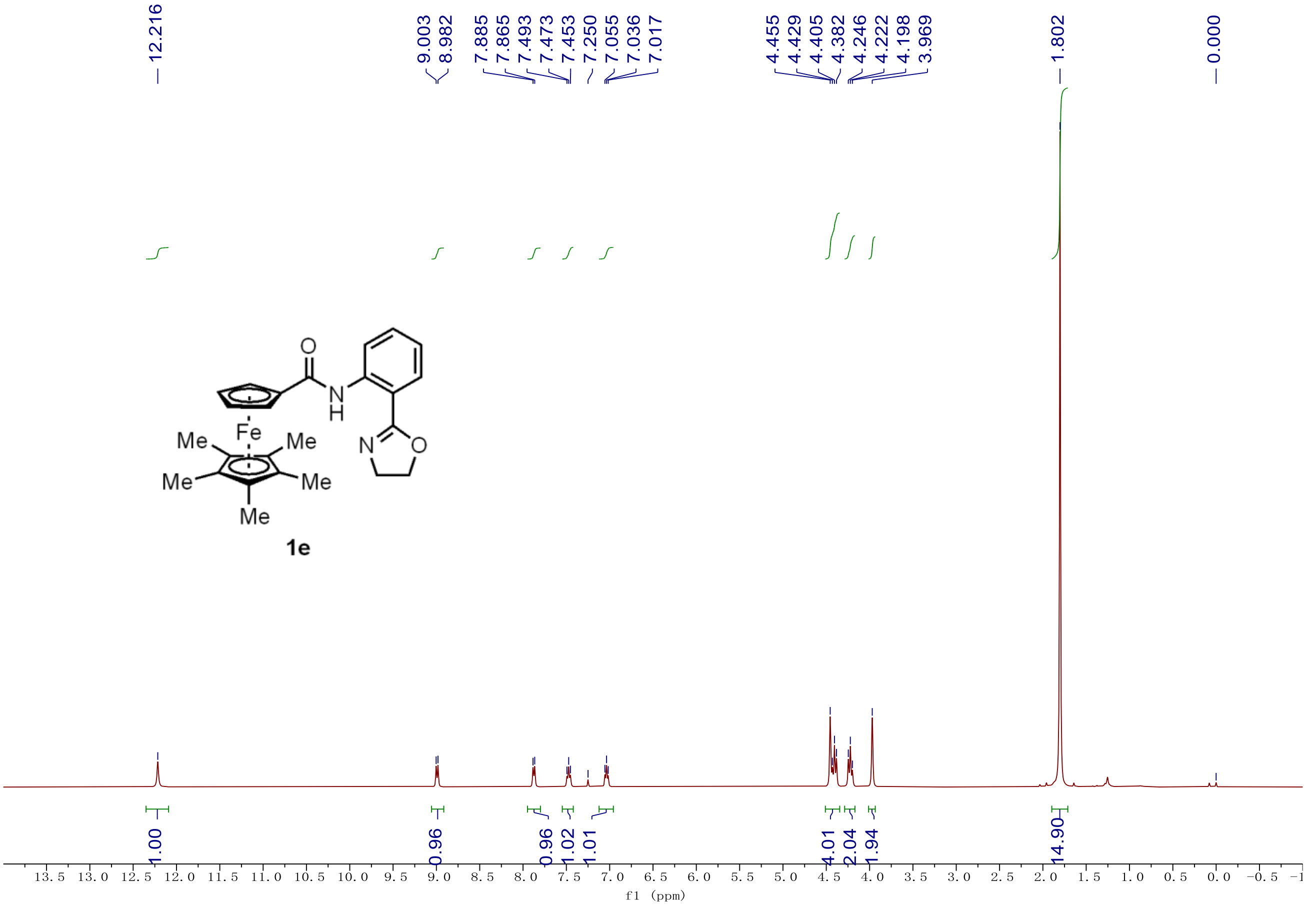
12.216
9.003
8.982
7.885
7.865
7.493
7.473
7.453
7.250
7.055
7.036
7.017
4.455
4.429
4.405
4.382
4.246
4.222
4.198
3.969
1.802
0.000
Fe
Me
Me
Me
Me
Me
O
N
H
N
O
1e
1.00
0.96
0.96
1.02
1.01
4.01
2.04
1.94
14.90
f1 (ppm)

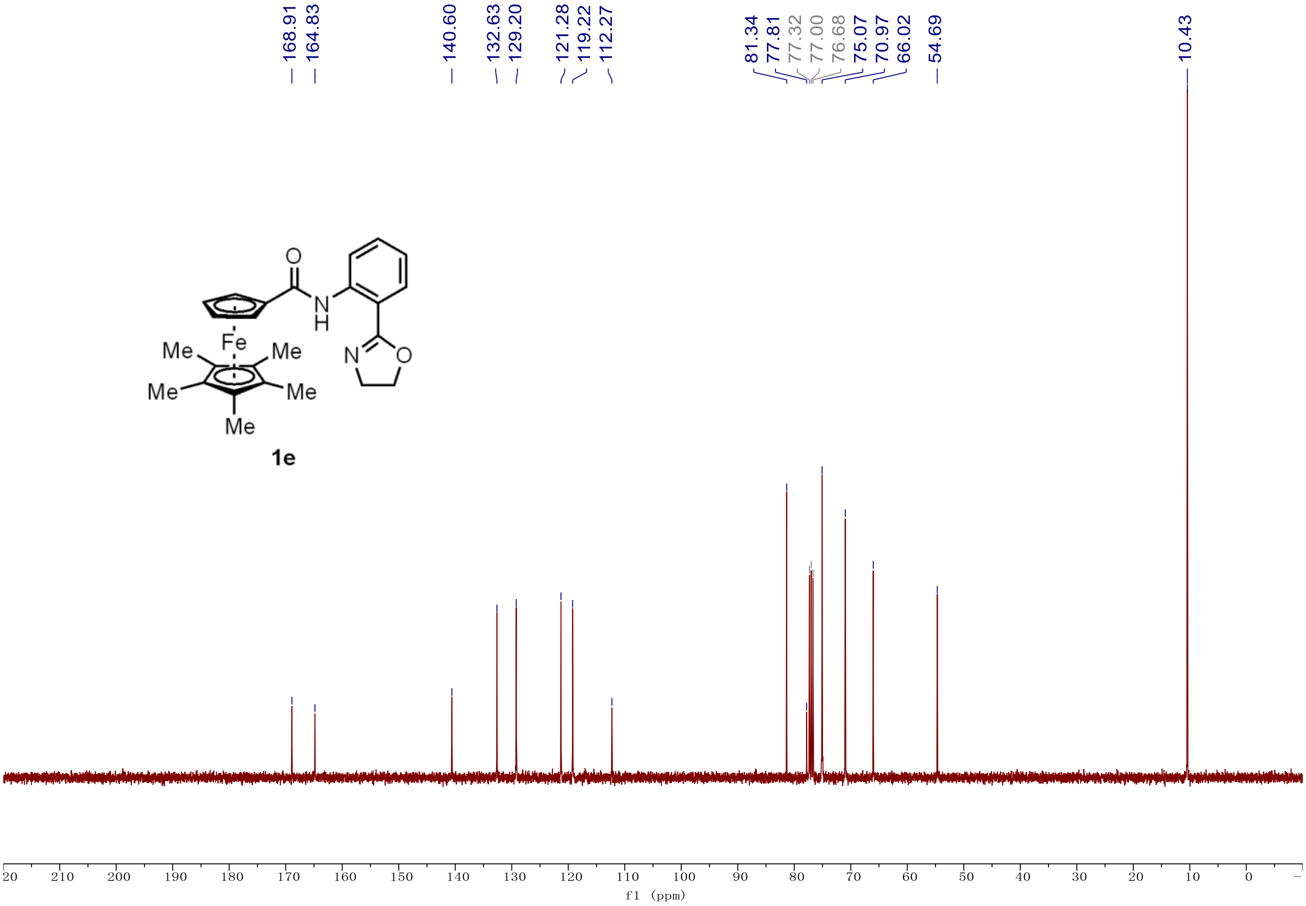
168.91
164.83
140.60
132.63
129.20
121.28
119.22
112.27
81.34
77.81
77.32
77.00
76.68
75.07
70.97
66.02
54.69
10.43
Fe
Me
Me
Me
Me
Me
O
N
H
N
O
1e
f1 (ppm)

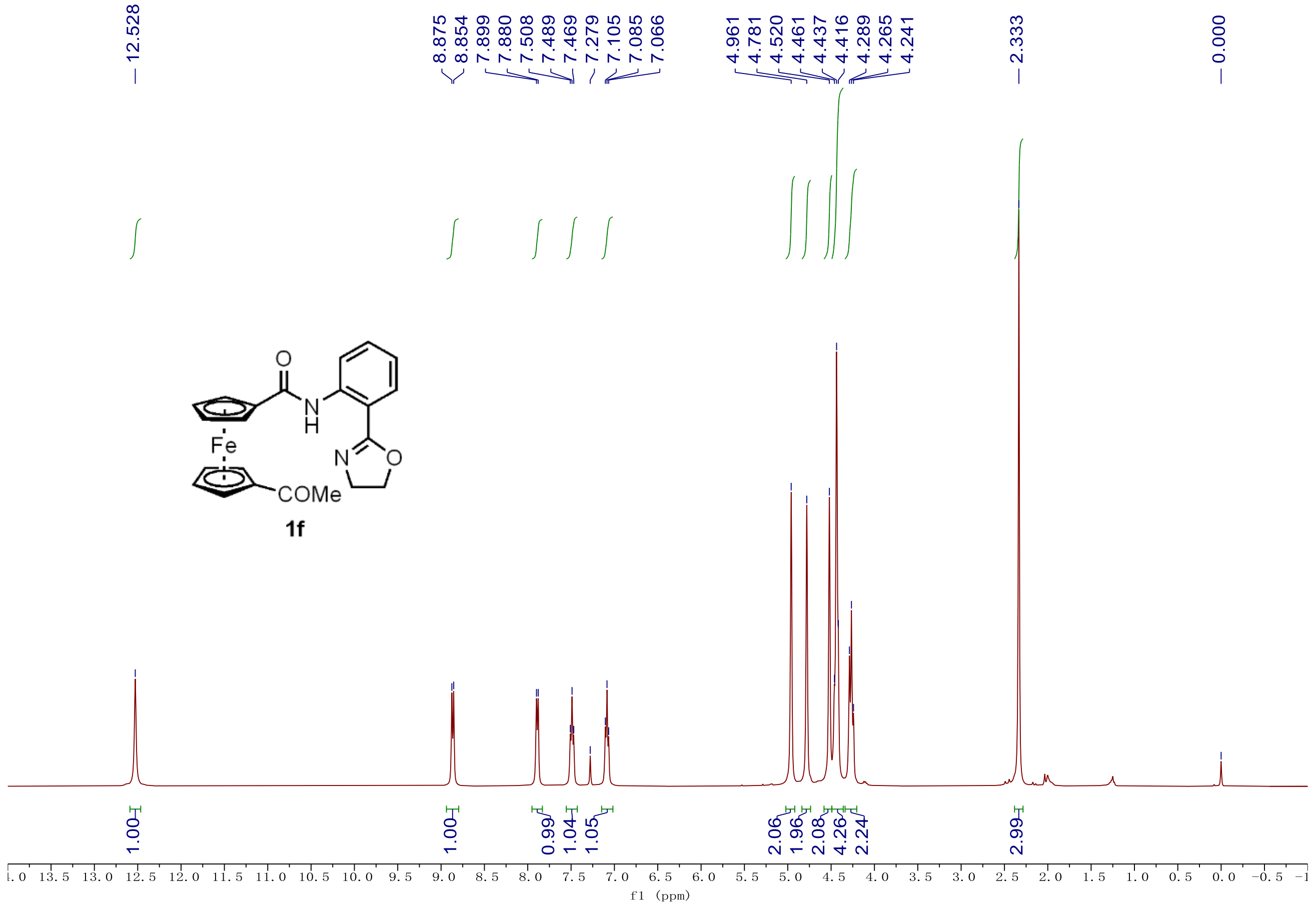
12.528
8.875
8.854
7.899
7.880
7.508
7.489
7.469
7.279
7.105
7.085
7.066
4.961
4.781
4.520
4.461
4.437
4.416
4.289
4.265
4.241
2.333
0.000
O
N
H
Fe
N
O
COMe
1f
1.00
1.00
0.99
1.04
1.05
2.06
1.96
2.08
4.26
2.24
2.99
f1 (ppm)

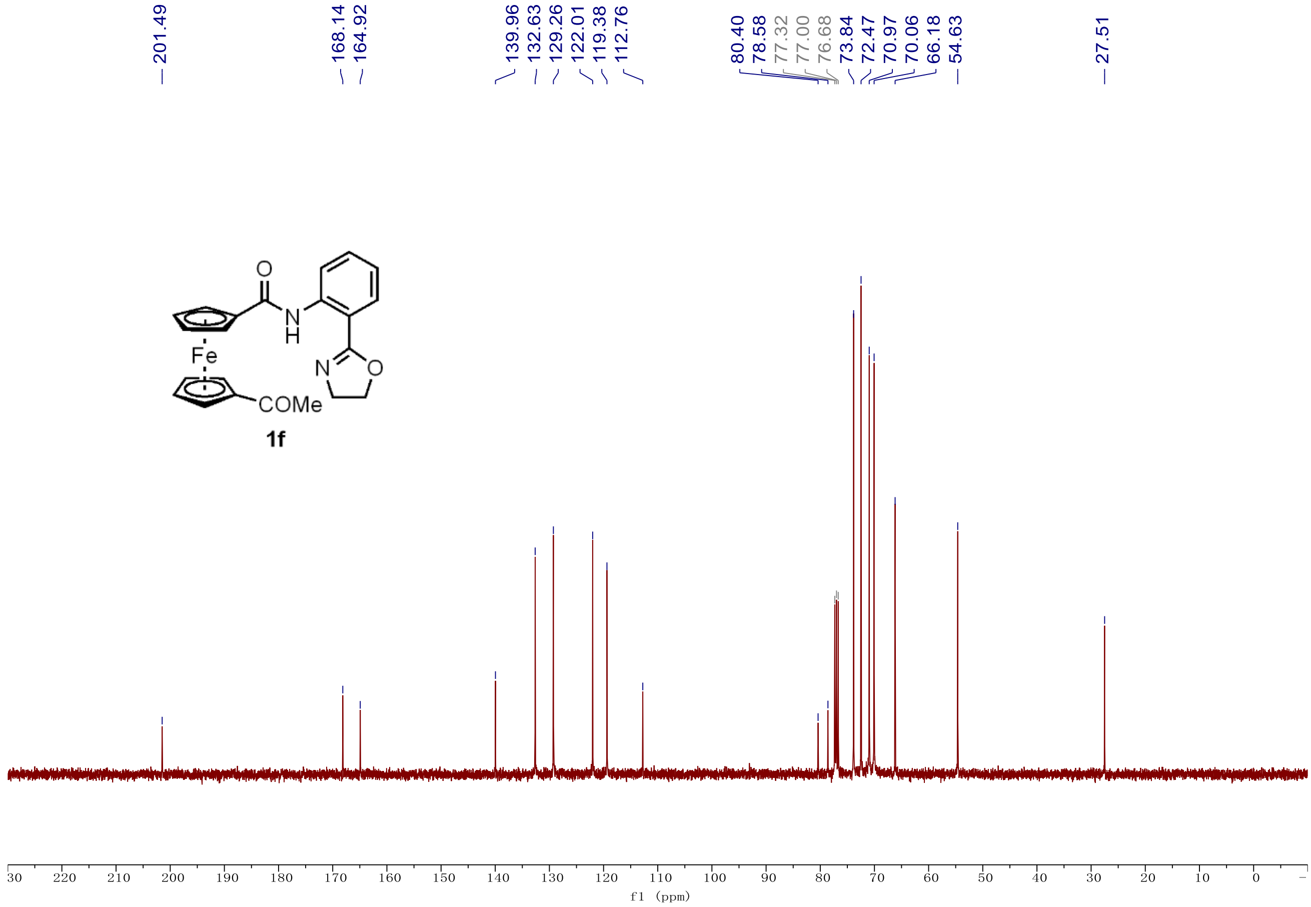
201.49
168.14
164.92
139.96
132.63
129.26
122.01
119.38
112.76
80.40
78.58
77.32
77.00
76.68
73.84
72.47
70.97
70.06
66.18
54.63
27.51
O
N
H
Fe
N
O
COMe
1f
f1 (ppm)

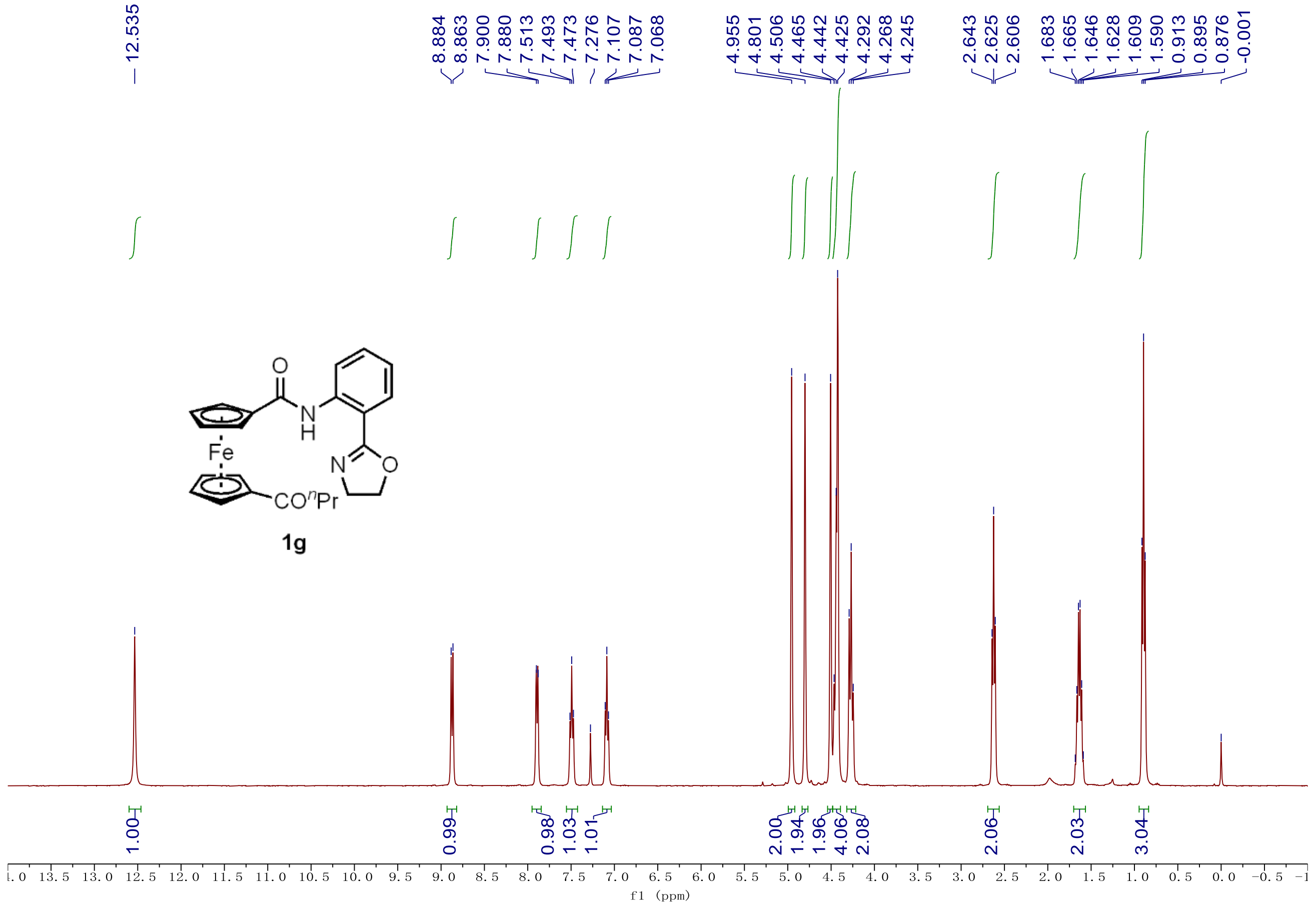
12.535
8.884
8.863
7.900
7.880
7.513
7.493
7.473
7.276
7.107
7.087
7.068
4.955
4.801
4.506
4.465
4.442
4.425
4.292
4.268
4.245
2.643
2.625
2.606
1.683
1.665
1.646
1.628
1.609
1.590
0.913
0.895
0.876
-0.001
Fe
CO^{n}Pr
1g
1.00
0.99
0.98
1.03
1.01
2.00
1.94
1.96
4.06
2.08
2.06
2.03
3.04
f1 (ppm)

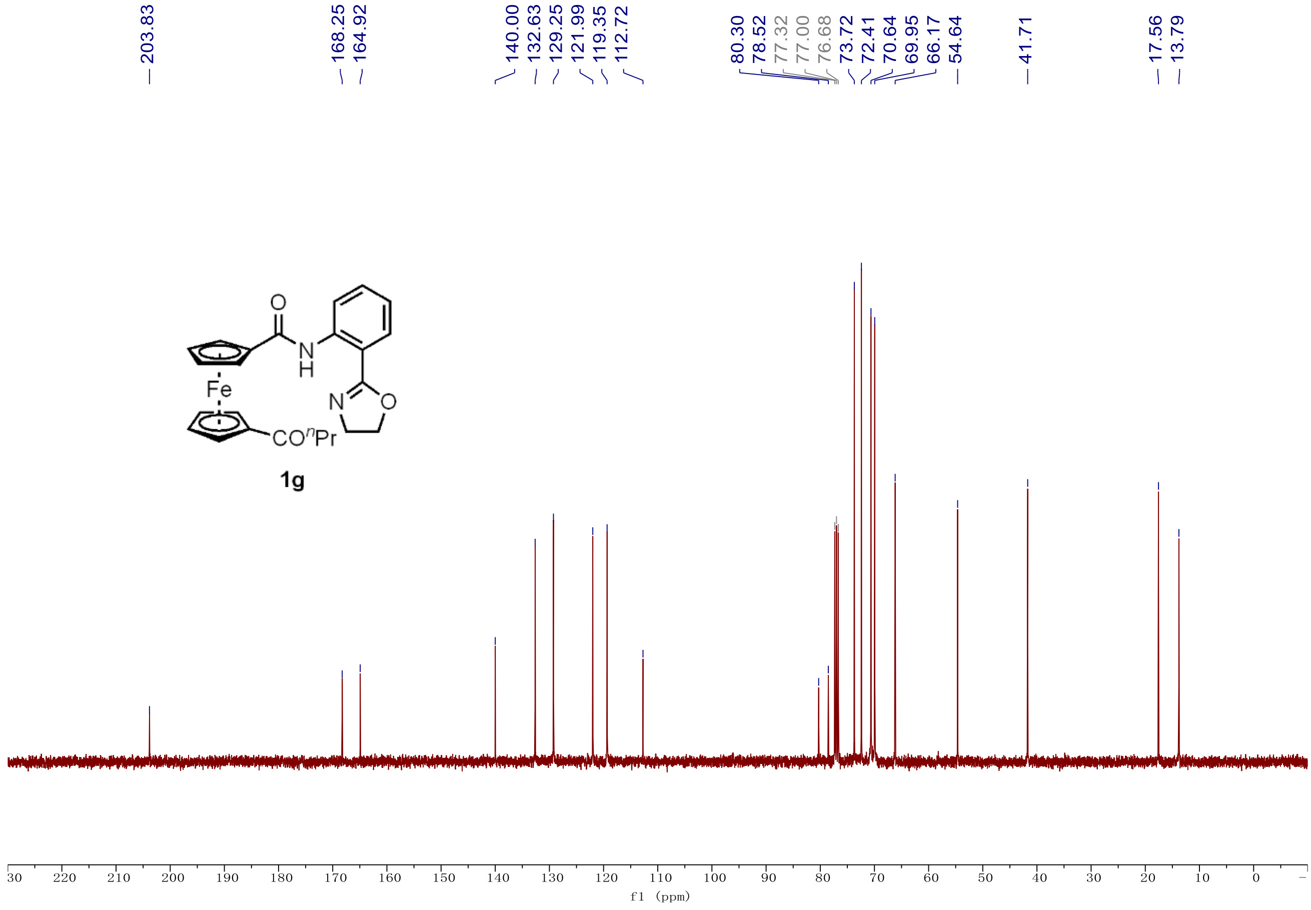
203.83
168.25
164.92
140.00
132.63
129.25
121.99
119.35
112.72
80.30
78.52
77.32
77.00
76.68
73.72
72.41
70.64
69.95
66.17
54.64
41.71
17.56
13.79
Fe
CO^{n}Pr
1g
f1 (ppm)

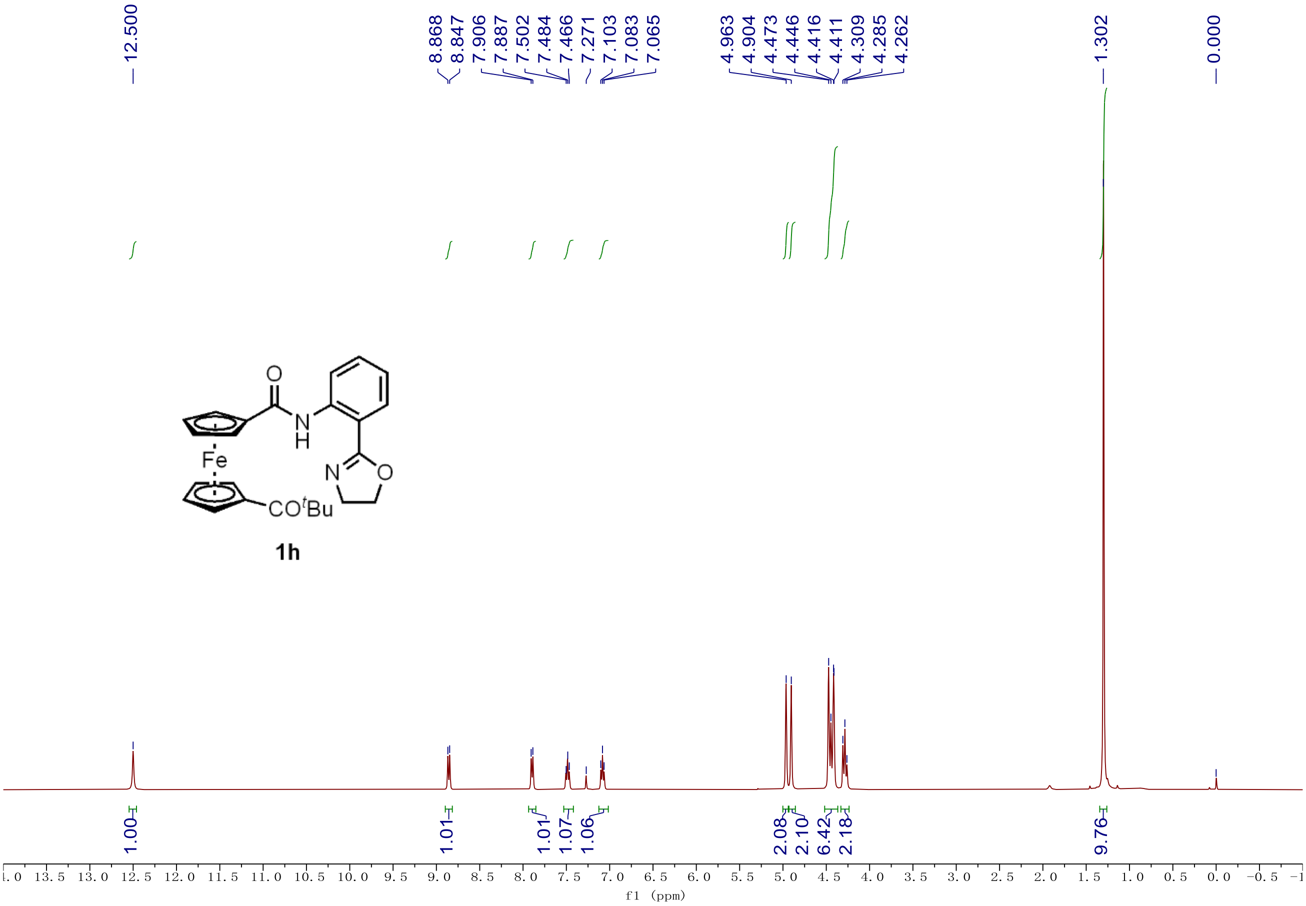
12.500
8.868
8.847
7.906
7.887
7.502
7.484
7.466
7.271
7.103
7.083
7.065
4.963
4.904
4.473
4.446
4.416
4.411
4.309
4.285
4.262
1.302
0.000
Fe
O
N
H
N
O
CO^{t}Bu
1h
1.00
1.01
1.01
1.07
1.06
2.08
2.10
6.42
2.18
9.76
f1 (ppm)

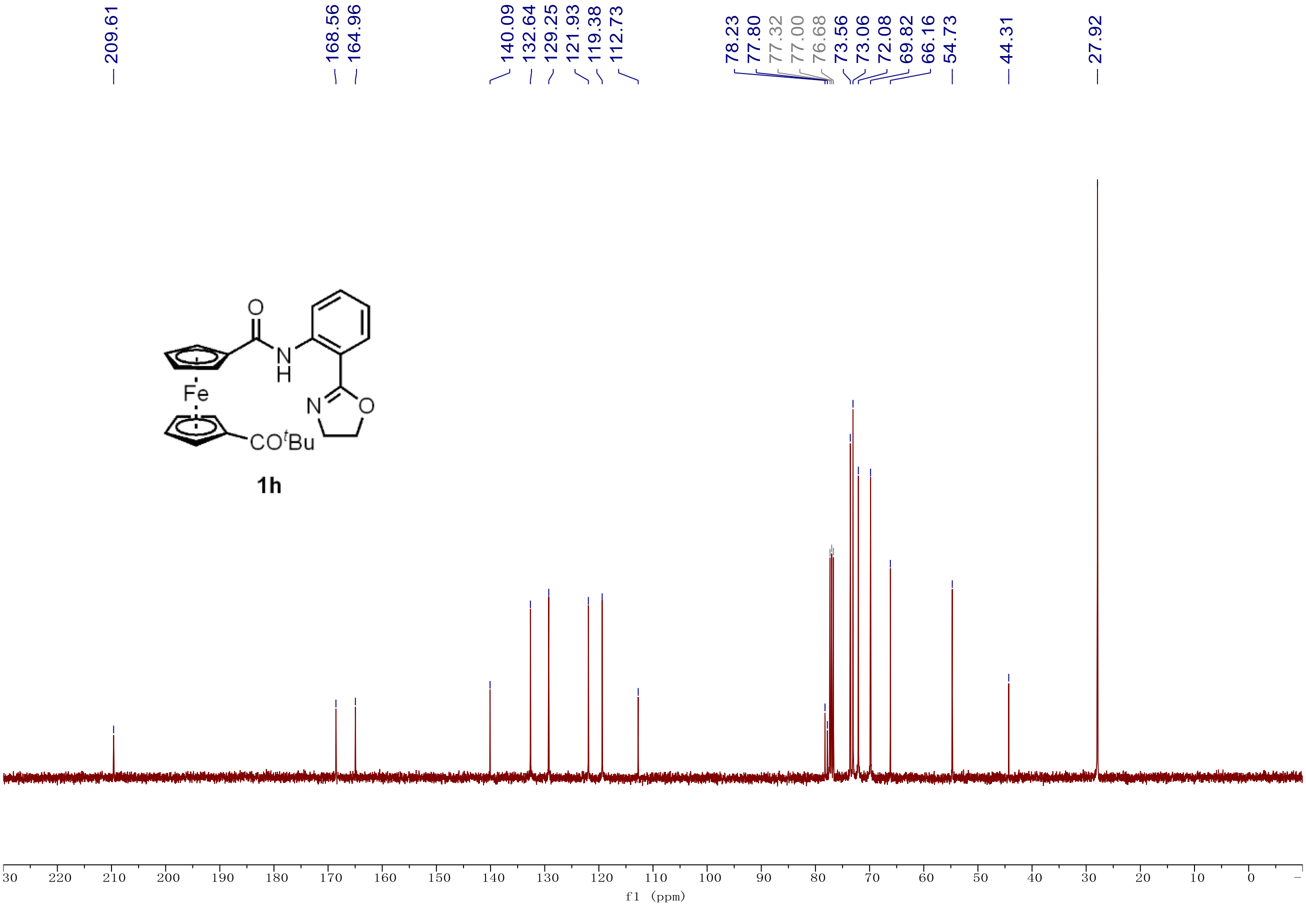
209.61
168.56
164.96
140.09
132.64
129.25
121.93
119.38
112.73
78.23
77.80
77.32
77.00
76.68
73.56
73.06
72.08
69.82
66.16
54.73
44.31
27.92
Fe
O
N
H
N
O
CO^{t}Bu
1h
f1 (ppm)

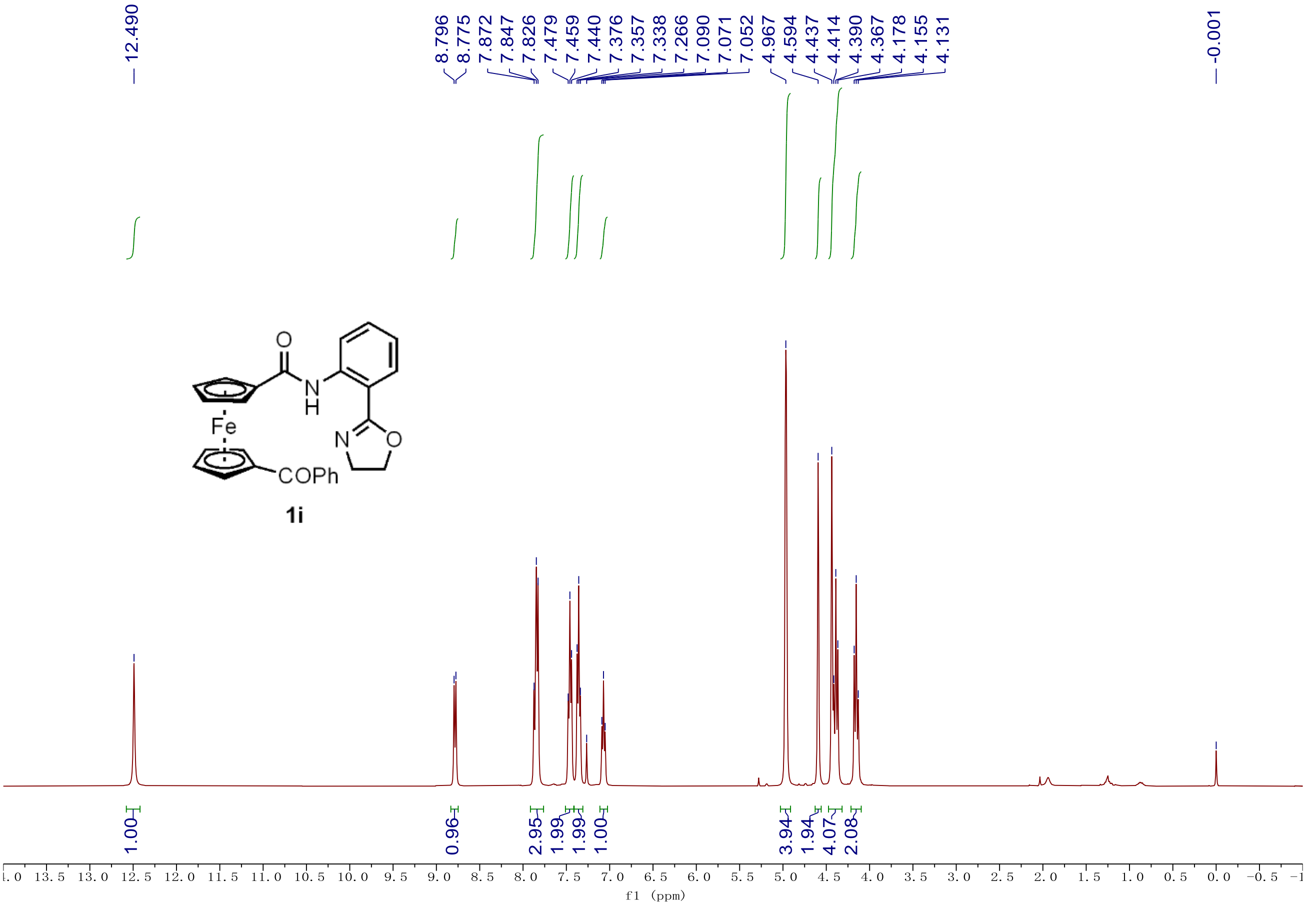
12.490
8.796 8.775 7.872 7.847 7.826 7.479 7.459 7.440 7.376 7.357 7.338 7.266 7.090 7.071 7.052 4.967 4.594 4.437 4.414 4.390 4.367 4.178 4.155 4.131
-0.001
O
N
H
Fe
N
O
COPh
1i
1.00 0.96 2.95 1.99 1.99 1.00 3.94 1.94 4.07 2.08
f1 (ppm)

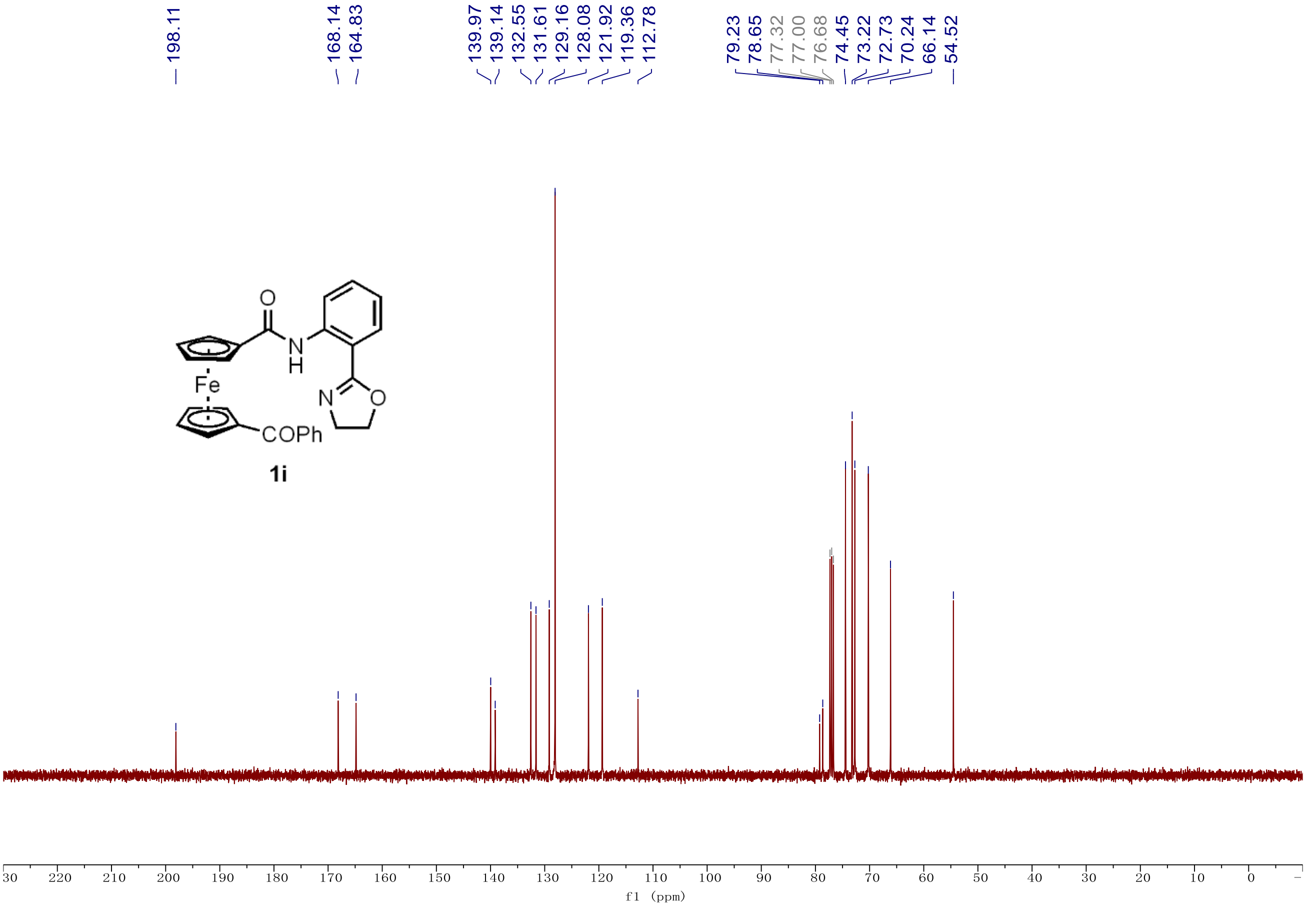
198.11
168.14 164.83
139.97 139.14 132.55 131.61 129.16 128.08 121.92 119.36 112.78
79.23 78.65 77.32 77.00 76.68 74.45 73.22 72.73 70.24 66.14 54.52
O
N
H
Fe
N
O
COPh
1i
f1 (ppm)

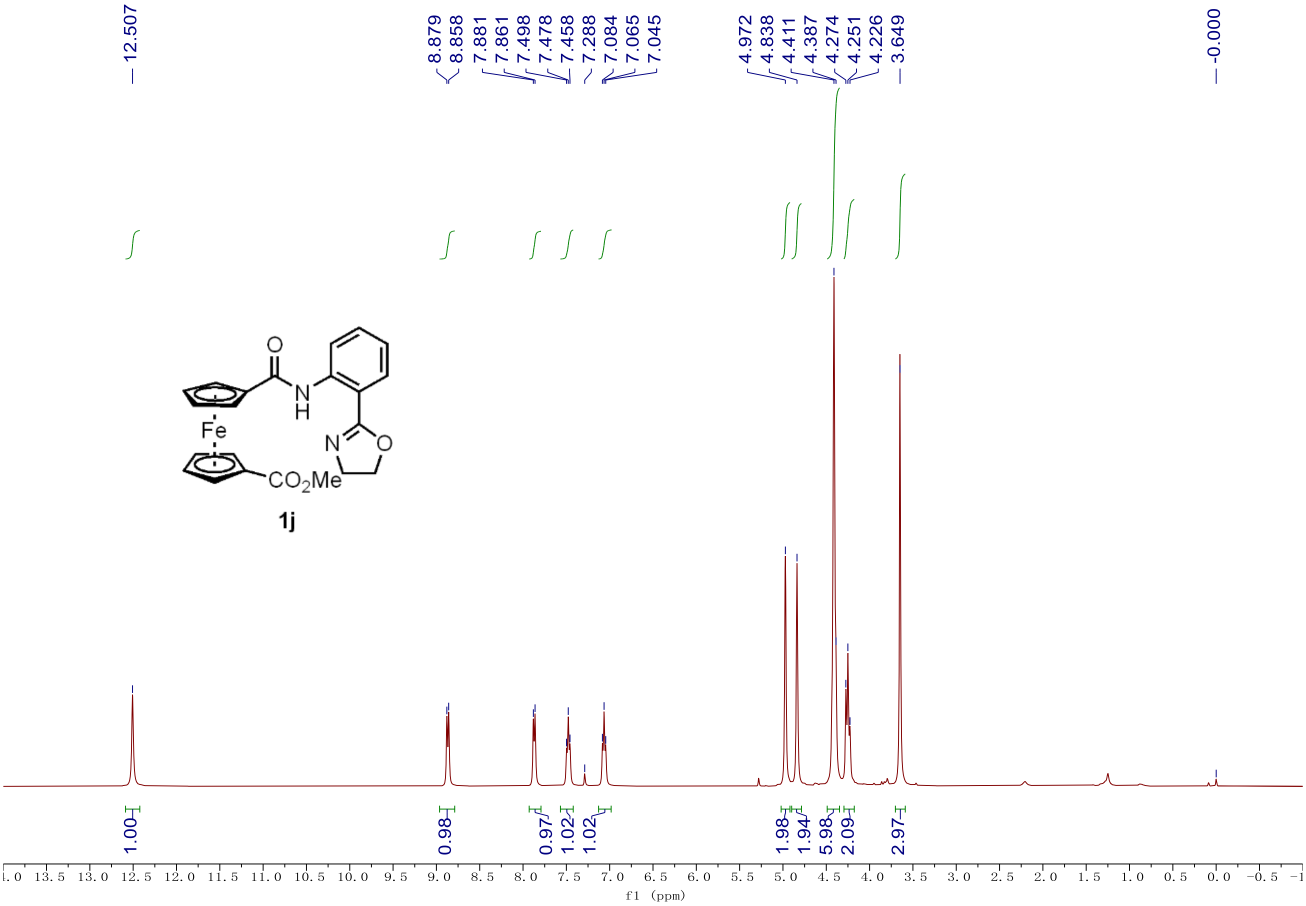
12.507
8.879
8.858
7.881
7.861
7.498
7.478
7.458
7.288
7.084
7.065
7.045
4.972
4.838
4.411
4.387
4.274
4.251
4.226
3.649
-0.000
1.00
0.98
0.97
1.02
1.02
1.98
1.94
5.98
2.09
2.97
Fe
CO2Me
N
H
O
N
O
1j
f1 (ppm)

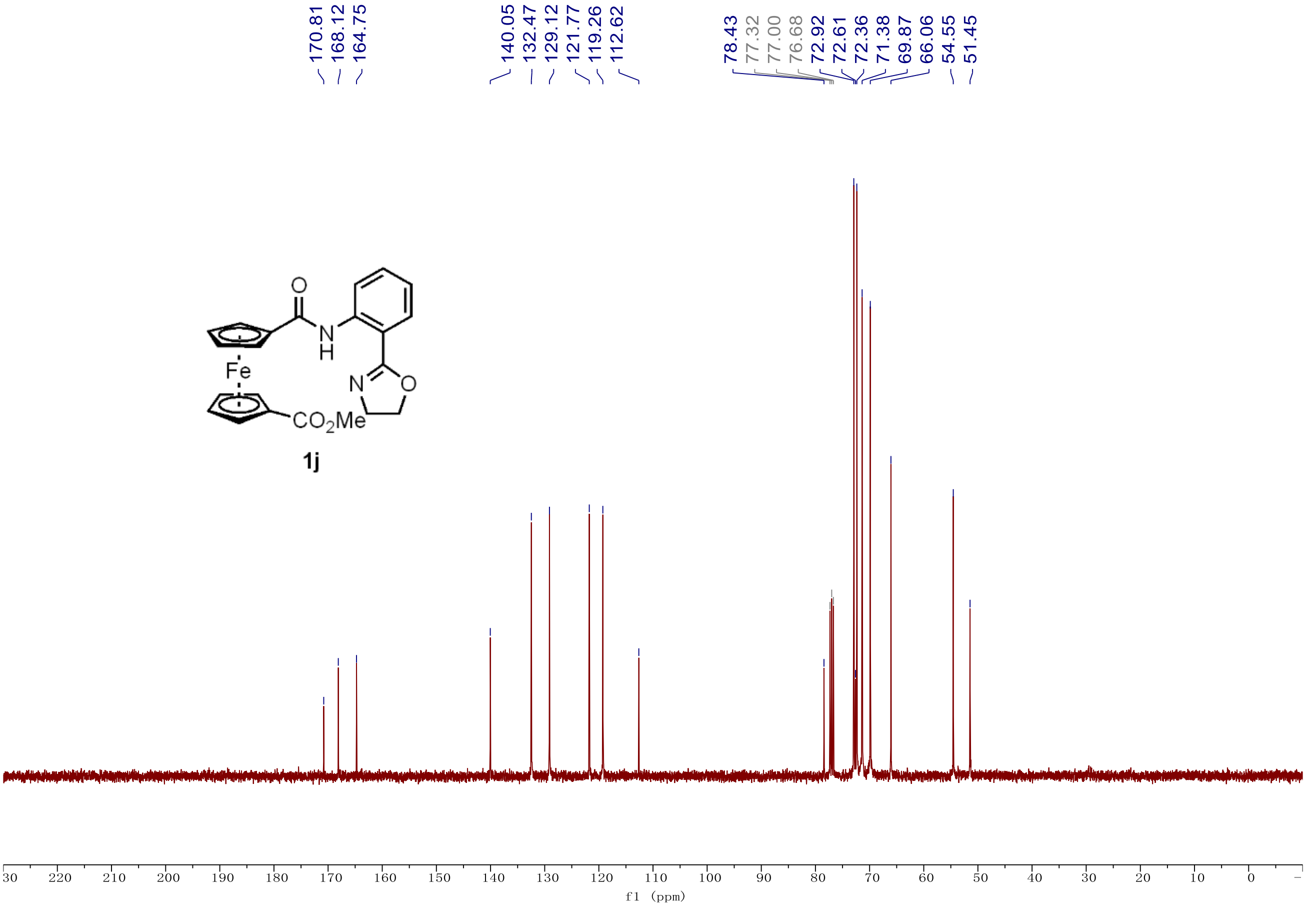
170.81
168.12
164.75
140.05
132.47
129.12
121.77
119.26
112.62
78.43
77.32
77.00
76.68
72.92
72.61
72.36
71.38
69.87
66.06
54.55
51.45
Fe
CO2Me
N
H
O
N
O
1j
f1 (ppm)

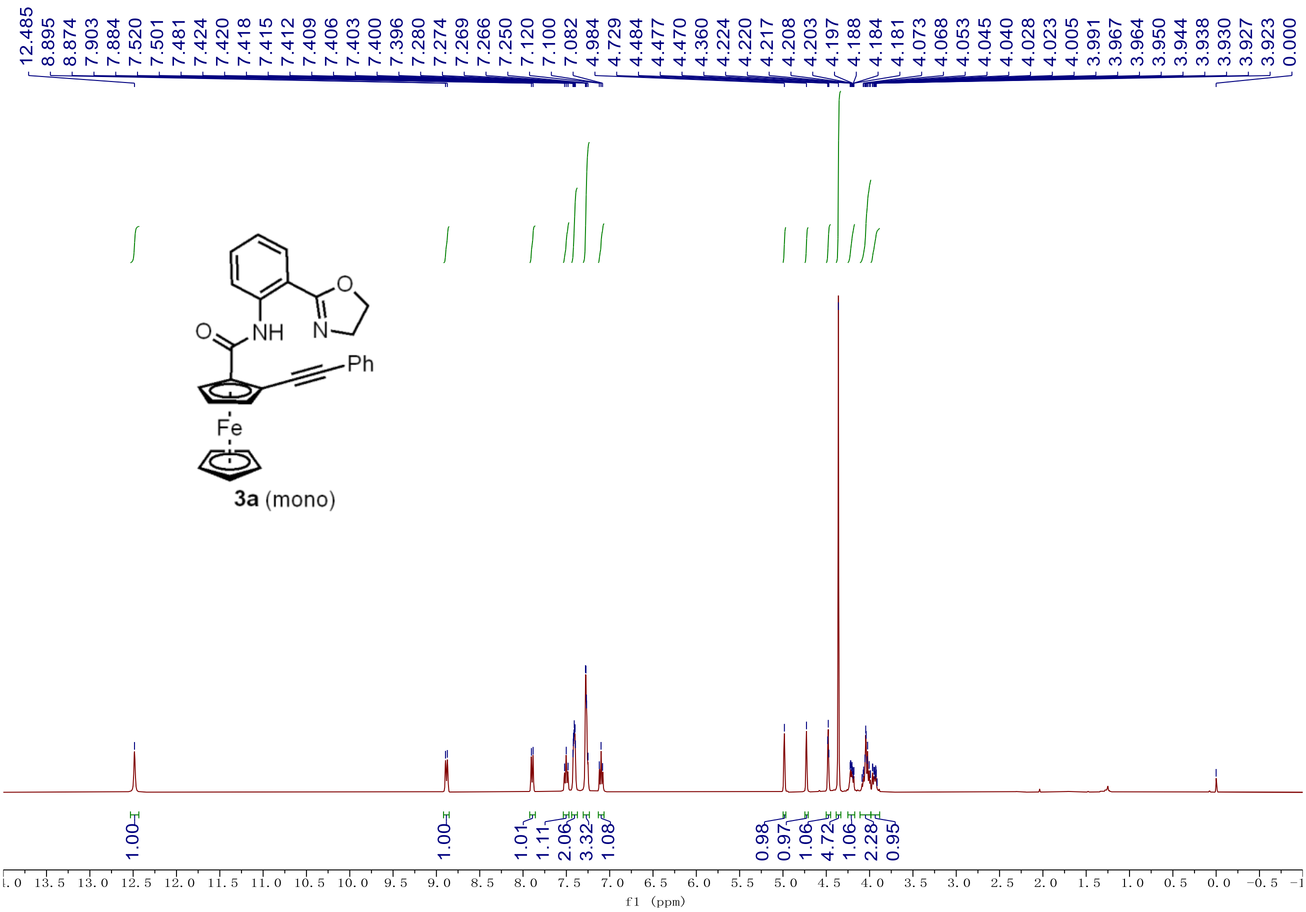
12.485
8.895
8.874
7.903
7.884
7.520
7.501
7.481
7.424
7.420
7.418
7.415
7.412
7.409
7.406
7.403
7.400
7.396
7.280
7.274
7.269
7.266
7.250
7.120
7.100
7.082
4.984
4.729
4.484
4.477
4.470
4.360
4.224
4.220
4.217
4.208
4.203
4.197
4.188
4.184
4.181
4.073
4.068
4.053
4.045
4.040
4.028
4.023
4.005
3.991
3.967
3.964
3.950
3.944
3.938
3.930
3.927
3.923
0.000
O
NH
O
N
Ph
Fe
3a (mono)
1.00
1.00
1.01
1.11
2.06
3.32
1.08
0.98
0.97
1.06
4.72
1.06
2.28
0.95
f1 (ppm)

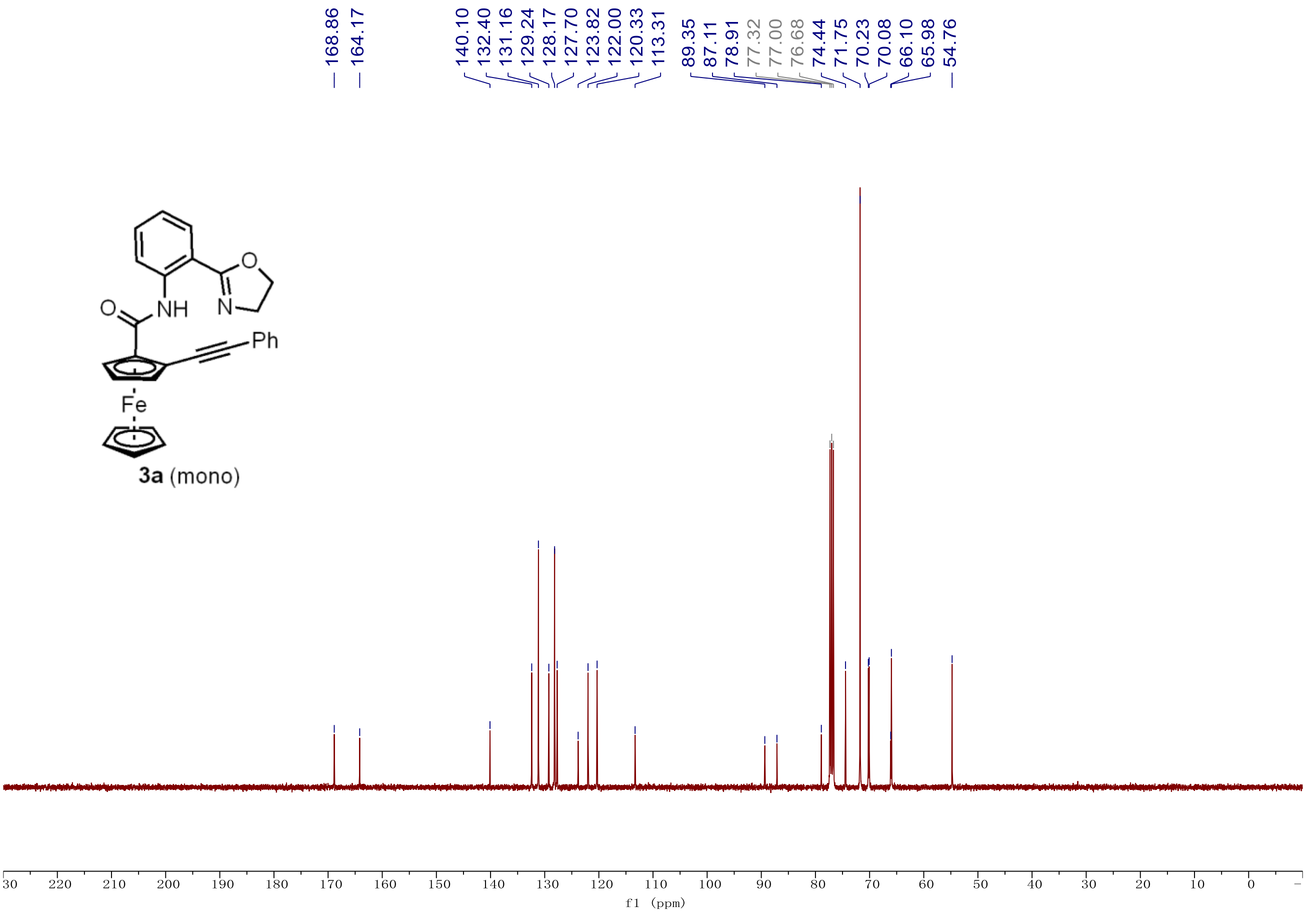
168.86
164.17
140.10
132.40
131.16
129.24
128.17
127.70
123.82
122.00
120.33
113.31
89.35
87.11
78.91
77.32
77.00
76.68
74.44
71.75
70.23
70.08
66.10
65.98
54.76
O
NH
O
N
Ph
Fe
3a (mono)
f1 (ppm)

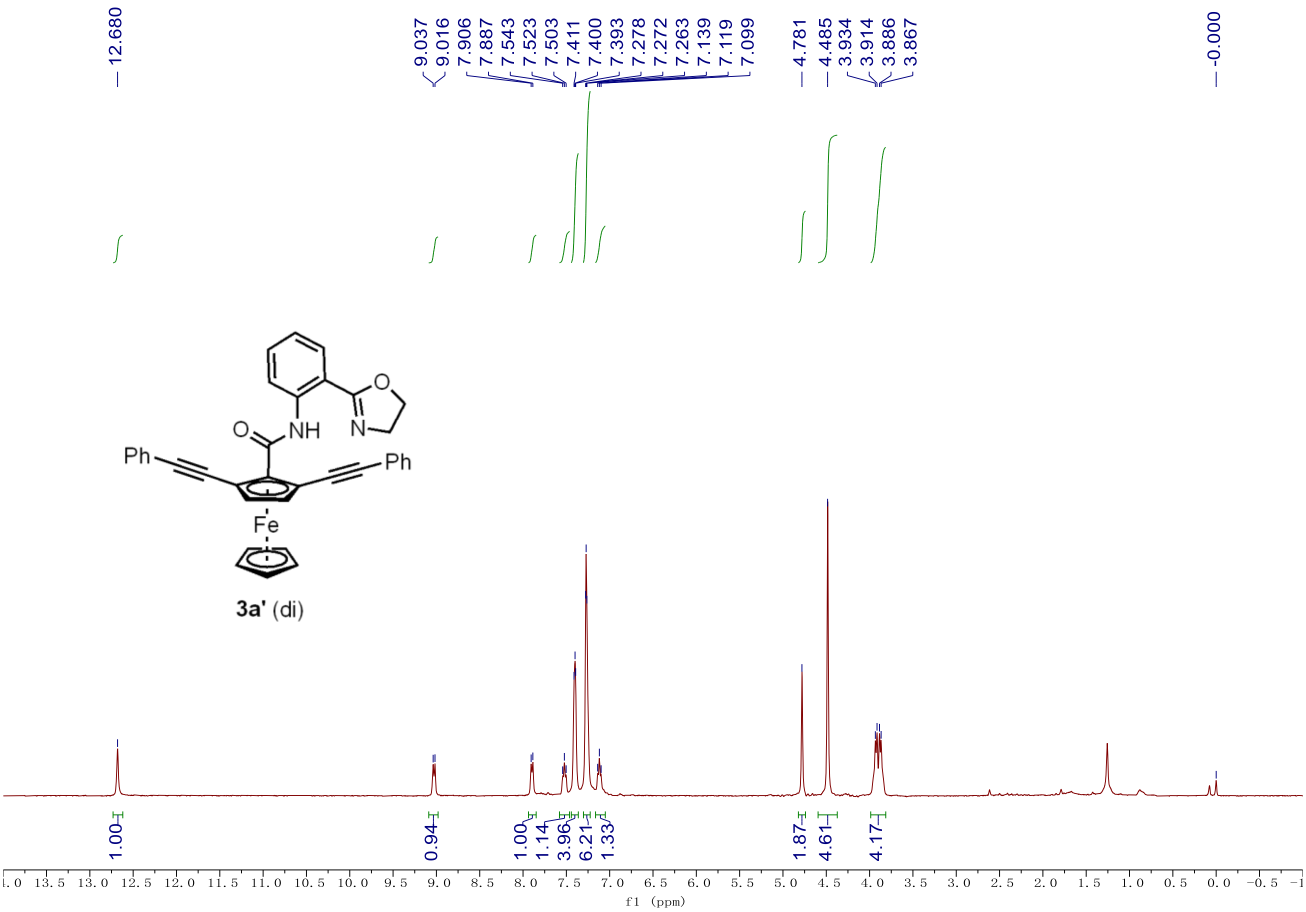
12.680
9.037 9.016 7.906 7.887 7.543 7.523 7.503 7.411 7.400 7.393 7.278 7.272 7.263 7.139 7.119 7.099
4.781 4.485 3.934 3.914 3.886 3.867
-0.000
1.00 0.94 1.00 1.14 3.96 6.21 1.33 1.87 4.61 4.17
Ph
O
NH
N
O
Ph
Fe
3a' (di)
f1 (ppm)

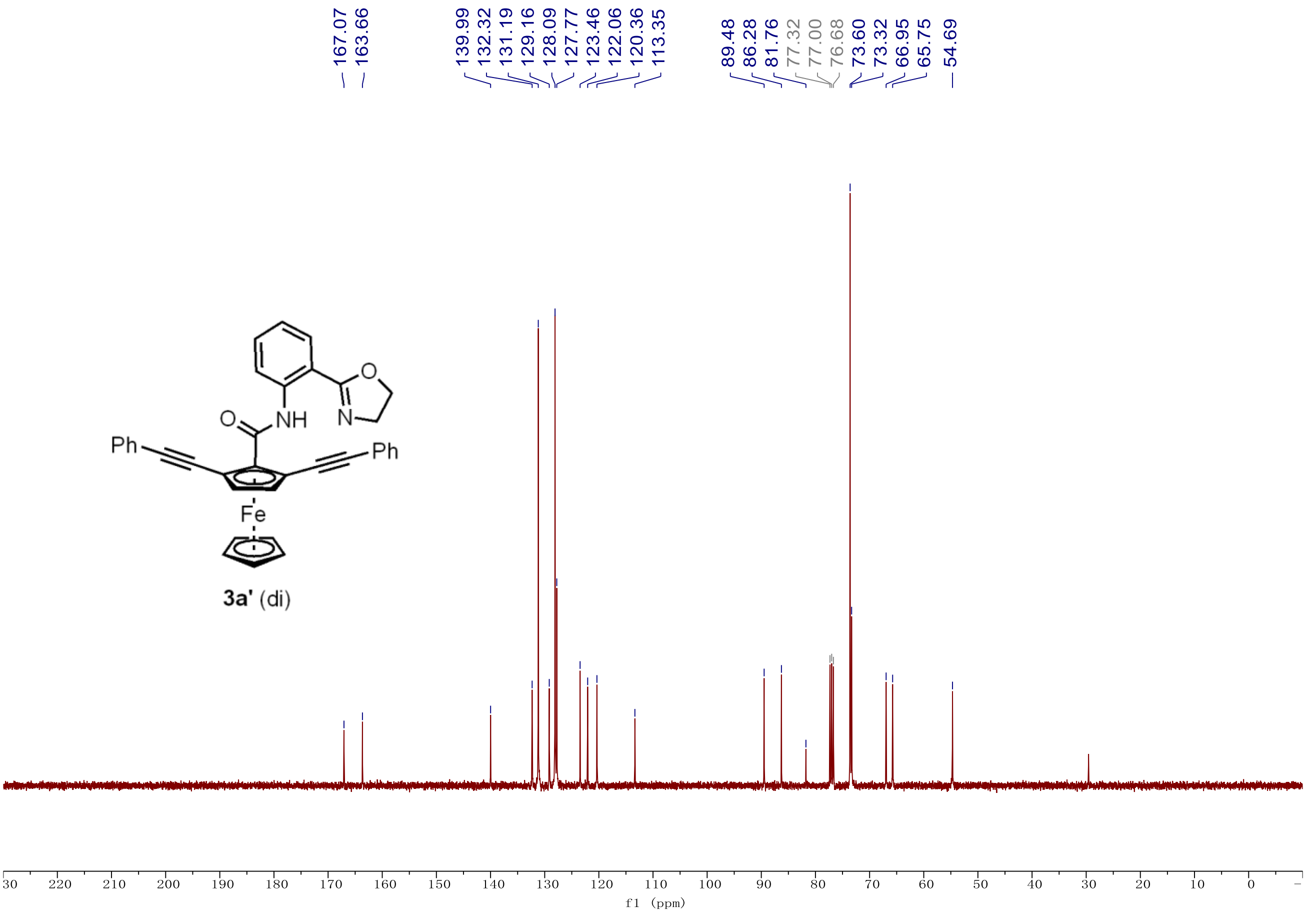
167.07 163.66
139.99 132.32 131.19 129.16 128.09 127.77 123.46 122.06 120.36 113.35
89.48 86.28 81.76 77.32 77.00 76.68 73.60 73.32 66.95 65.75 54.69
Ph
O
NH
N
O
Ph
Fe
3a' (di)
f1 (ppm)

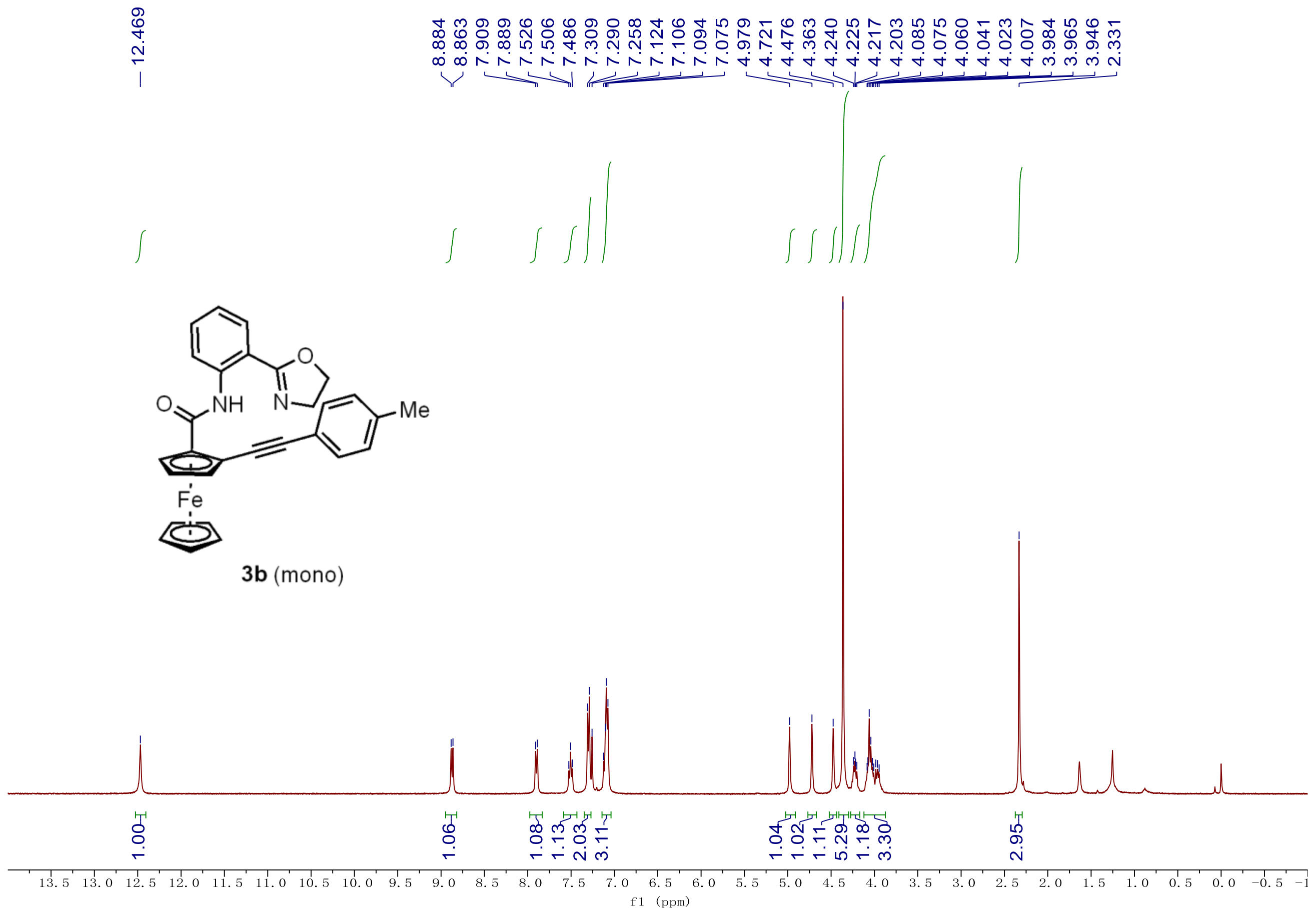
12.469
8.884
8.863
7.909
7.889
7.526
7.506
7.486
7.309
7.290
7.258
7.124
7.106
7.094
7.075
4.979
4.721
4.476
4.363
4.240
4.225
4.217
4.203
4.085
4.075
4.060
4.041
4.023
4.007
3.984
3.965
3.946
2.331
O
NH
N
O
Me
Fe
3b (mono)
1.00
1.06
1.08
1.13
2.03
3.11
1.04
1.02
1.11
5.29
1.18
3.30
2.95
f1 (ppm)

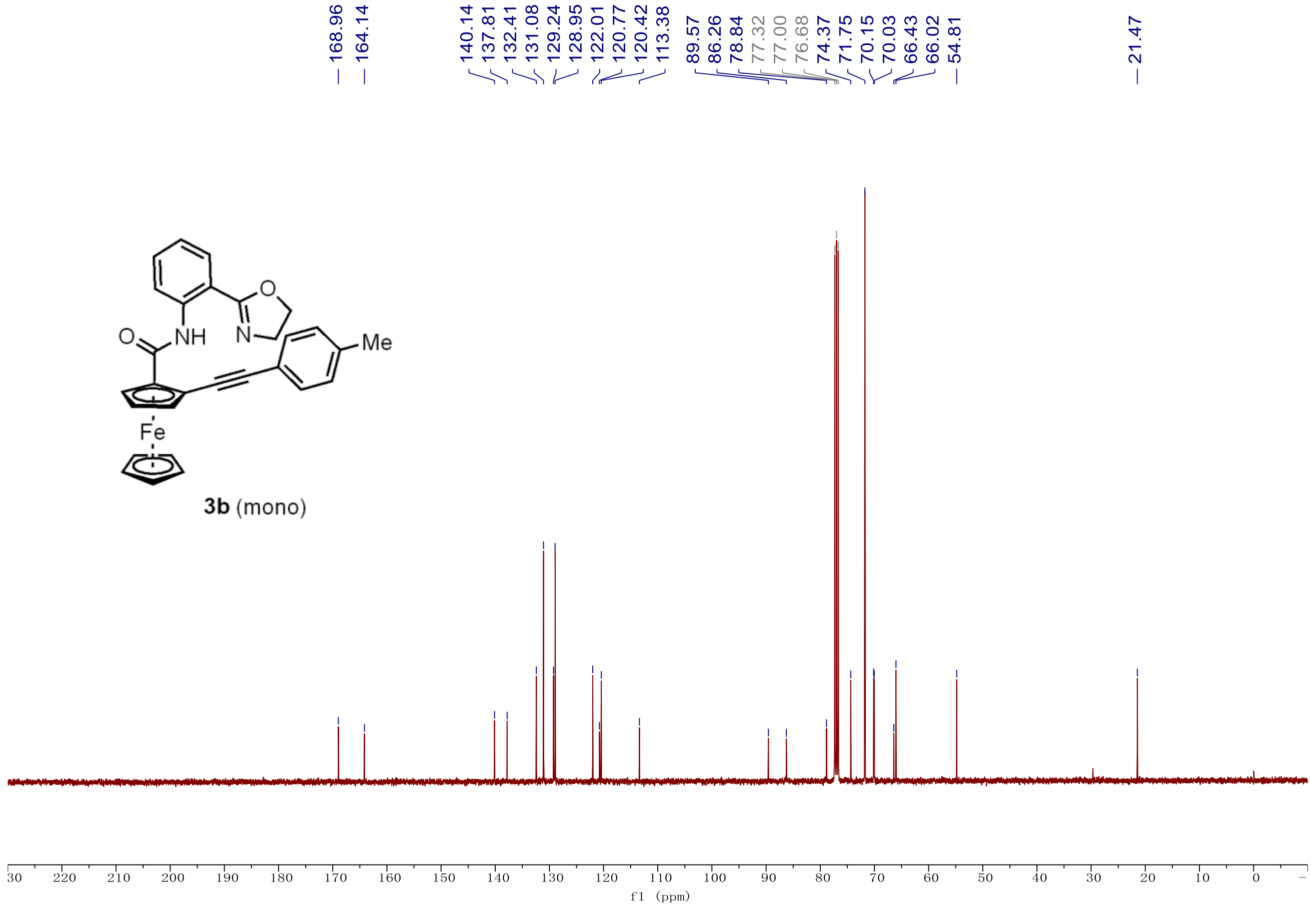
168.96
164.14
140.14
137.81
132.41
131.08
129.24
128.95
122.01
120.77
120.42
113.38
89.57
86.26
78.84
77.32
77.00
76.68
74.37
71.75
70.15
70.03
66.43
66.02
54.81
21.47
O
NH
N
O
Me
Fe
3b (mono)
f1 (ppm)

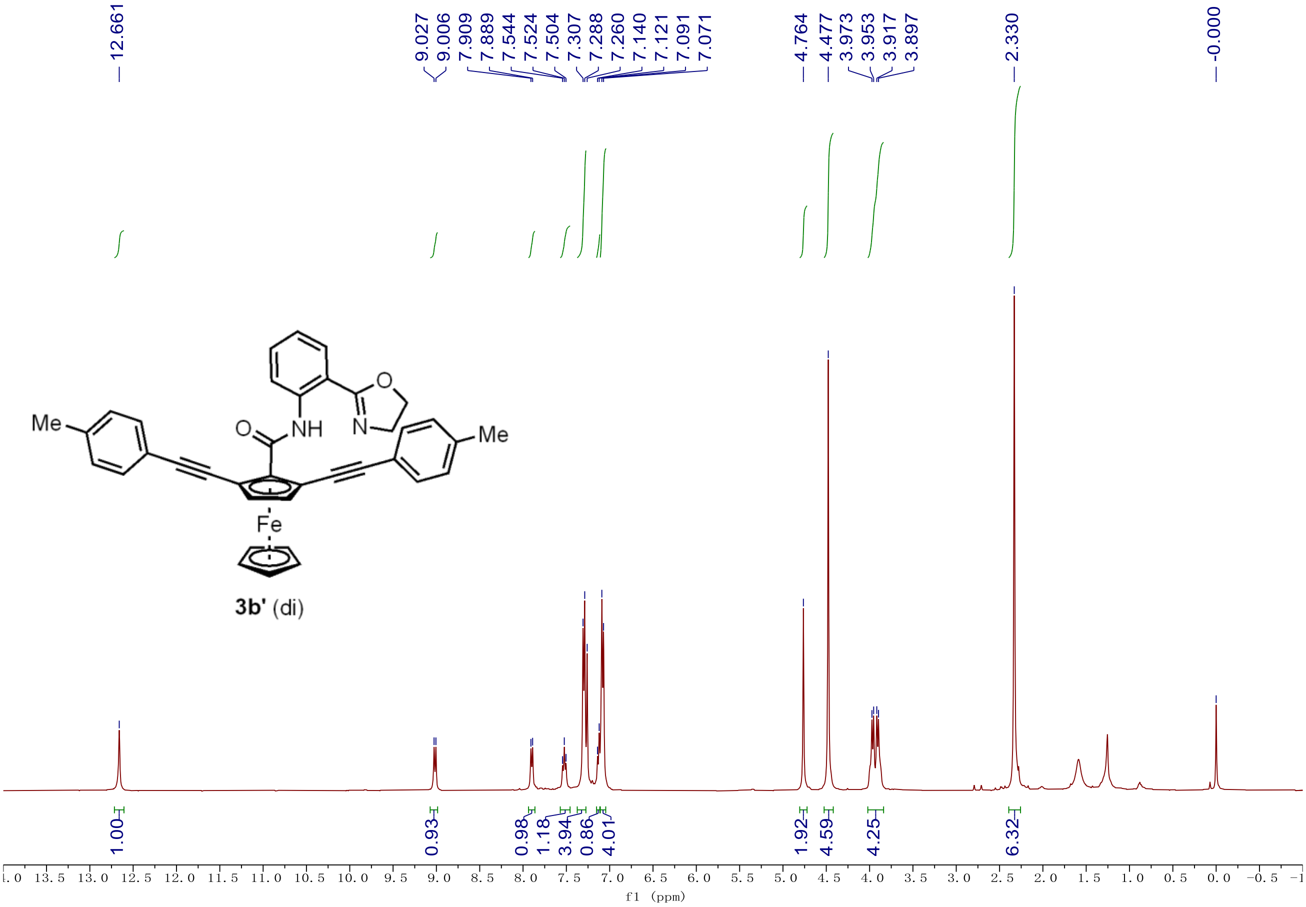
12.661
9.027
9.006
7.909
7.889
7.544
7.524
7.504
7.307
7.288
7.260
7.140
7.121
7.091
7.071
4.764
4.477
3.973
3.953
3.917
3.897
2.330
-0.000
Me
O
NH
N
O
Me
Fe
3b' (di)
1.00
0.93
0.98
1.18
3.94
0.86
4.01
1.92
4.59
4.25
6.32
f1 (ppm)

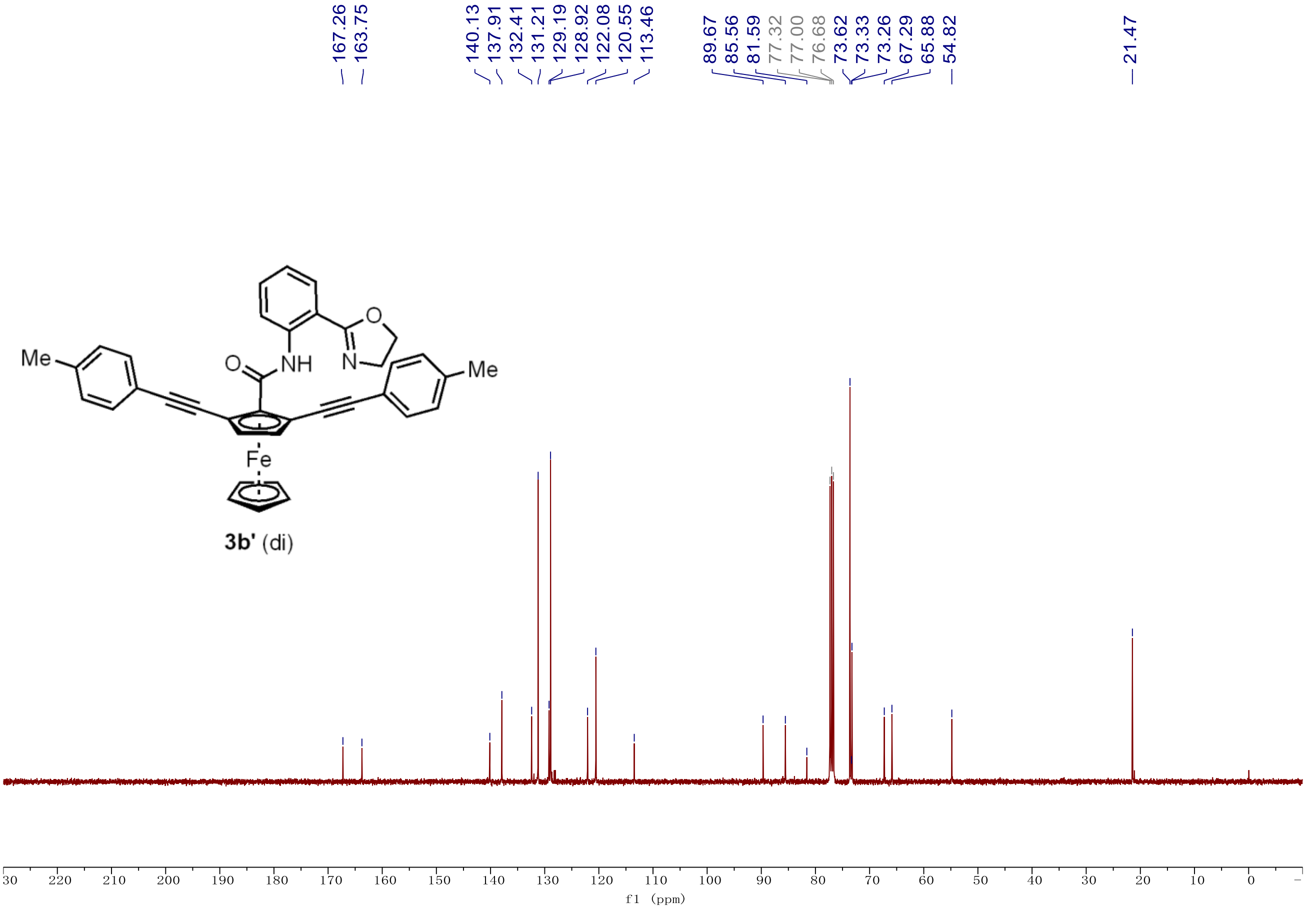
167.26
163.75
140.13
137.91
132.41
131.21
129.19
128.92
122.08
120.55
113.46
89.67
85.56
81.59
77.32
77.00
76.68
73.62
73.33
73.26
67.29
65.88
54.82
21.47
Me
O
NH
N
O
Me
Fe
3b' (di)
f1 (ppm)

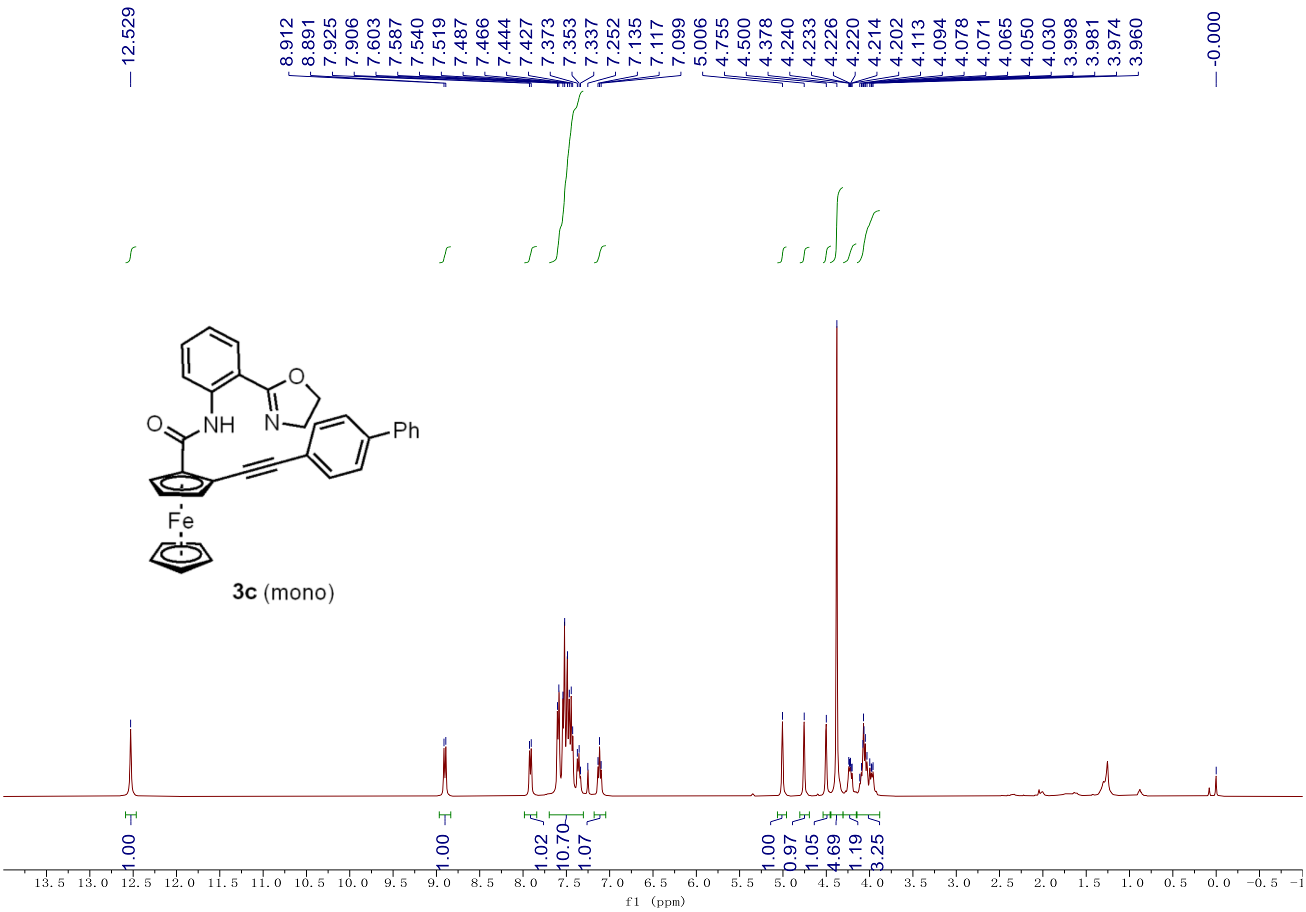
12.529
8.912 8.891 7.925 7.906 7.603 7.587 7.540 7.519 7.487 7.466 7.444 7.427 7.373 7.353 7.337 7.252 7.135 7.117 7.099 5.006 4.755 4.500 4.378 4.240 4.233 4.226 4.220 4.214 4.202 4.113 4.094 4.078 4.071 4.065 4.050 4.030 3.998 3.981 3.974 3.960
-0.000
O
NH
N
O
Ph
Fe
3c (mono)
1.00 1.00 1.02 10.70 1.07 1.00 0.97 1.05 4.69 1.19 3.25
f1 (ppm)

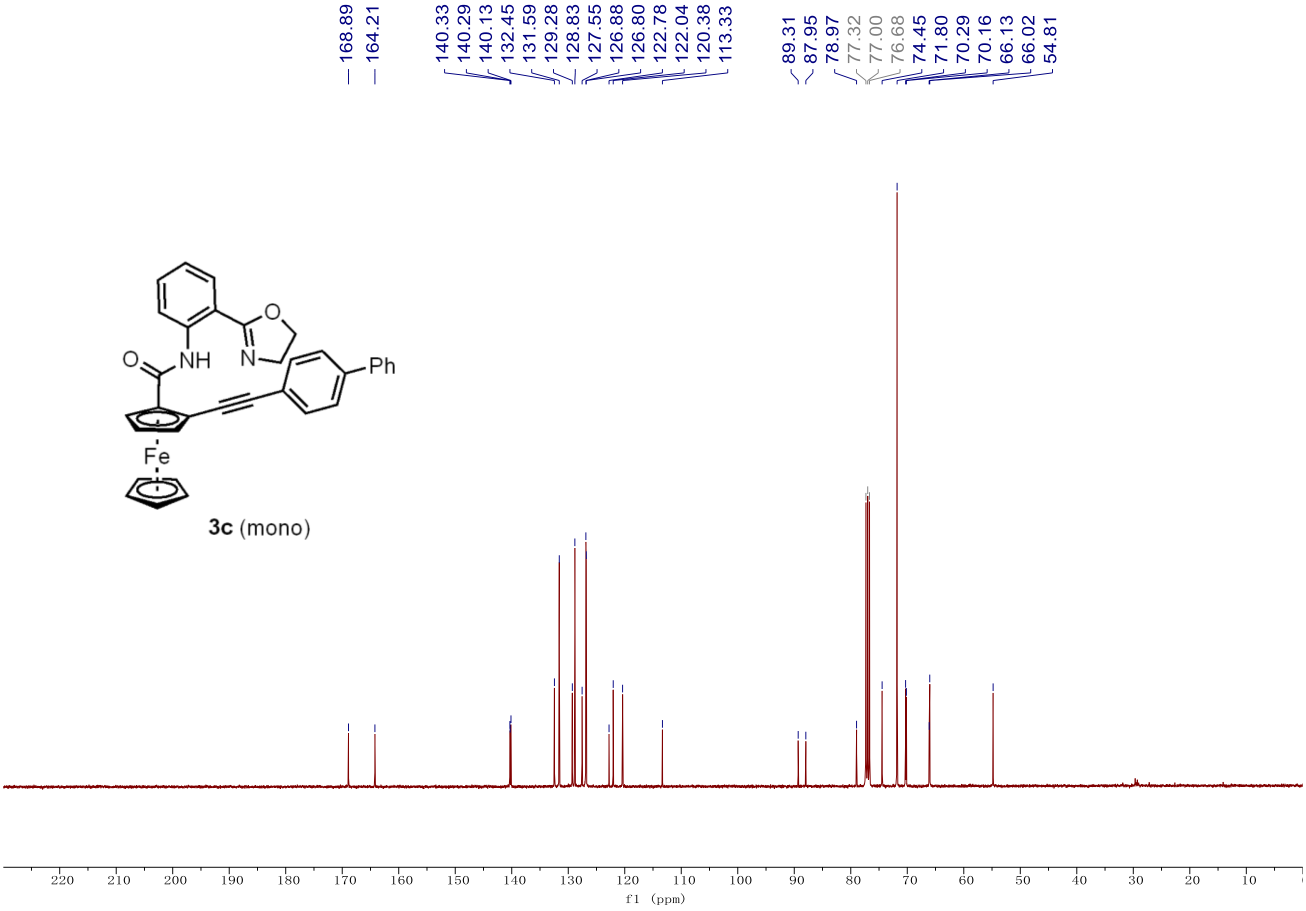
168.89 164.21
140.33 140.29 140.13 132.45 131.59 129.28 128.83 127.55 126.88 126.80 122.78 122.04 120.38 113.33
89.31 87.95 78.97 77.32 77.00 76.68 74.45 71.80 70.29 70.16 66.13 66.02 54.81
O
NH
N
O
Ph
Fe
3c (mono)
f1 (ppm)

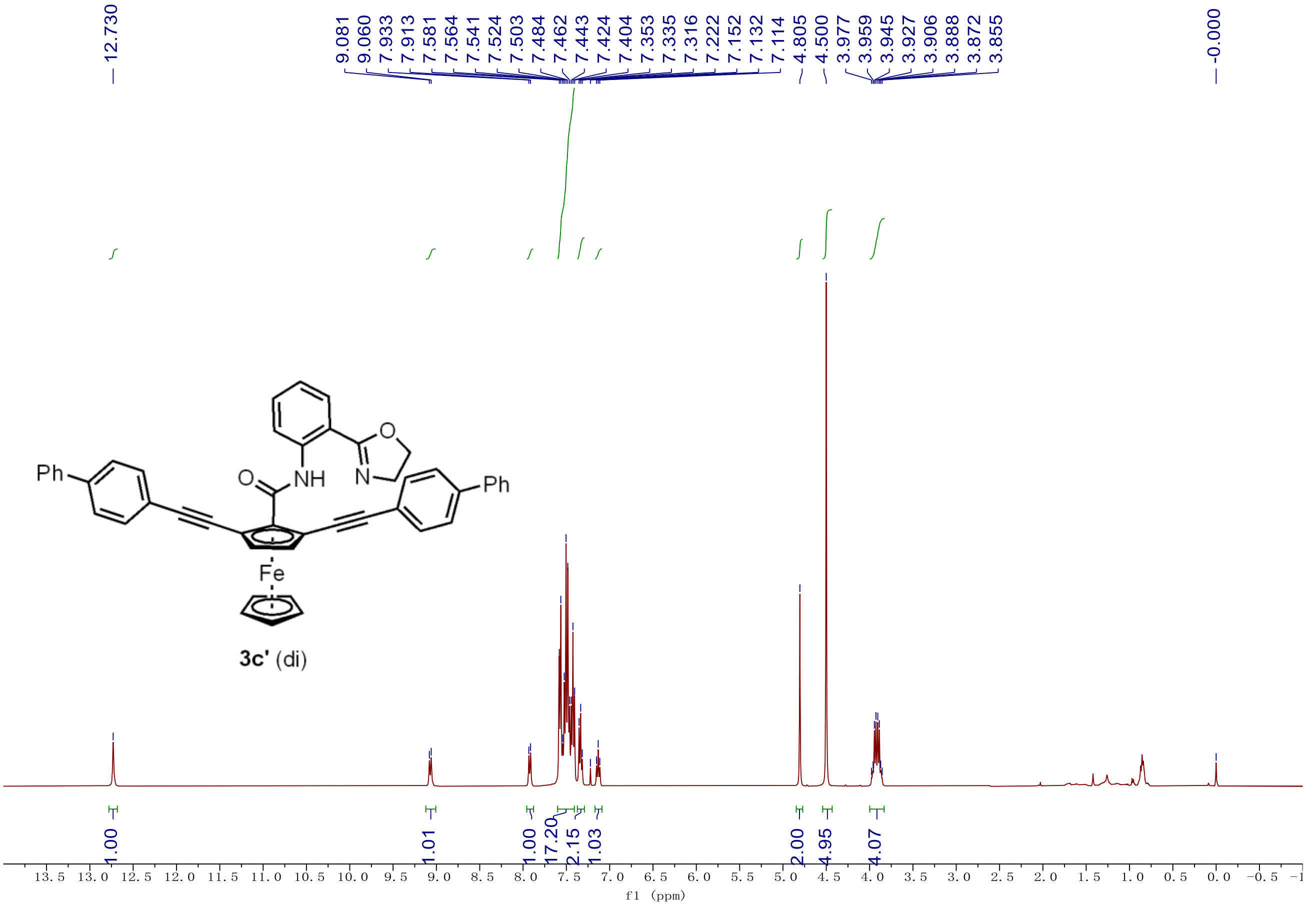
12.730
9.081
9.060
7.933
7.913
7.581
7.564
7.541
7.524
7.503
7.484
7.462
7.443
7.424
7.404
7.353
7.335
7.316
7.222
7.152
7.132
7.114
4.805
4.500
3.977
3.959
3.945
3.927
3.906
3.888
3.872
3.855
-0.000
Ph
O
NH
N
O
Ph
Fe
3c' (di)
1.00
1.01
1.00
17.20
2.15
1.03
2.00
4.95
4.07
f1 (ppm)

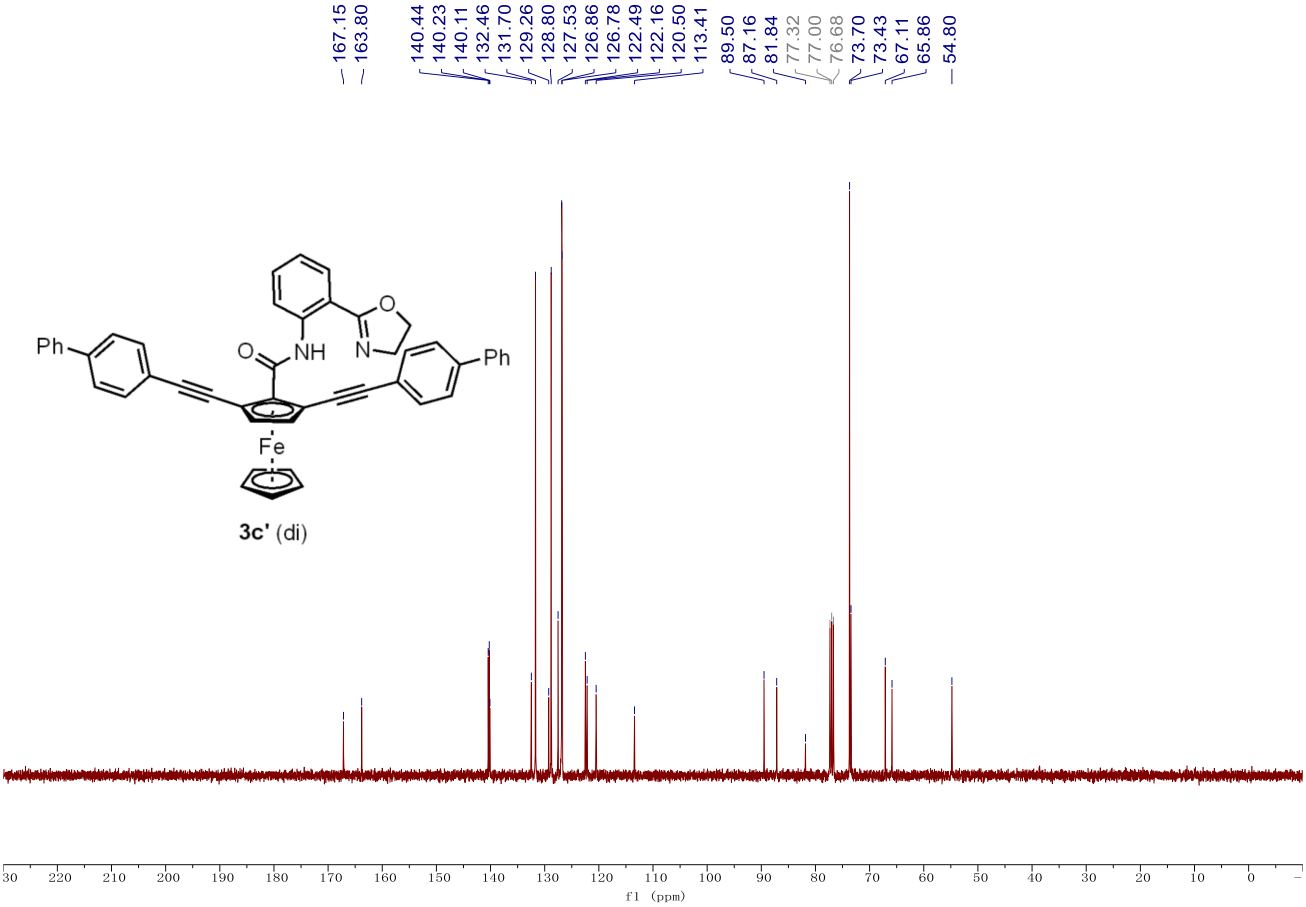
167.15
163.80
140.44
140.23
140.11
132.46
131.70
129.26
128.80
127.53
126.86
126.78
122.49
122.16
120.50
113.41
89.50
87.16
81.84
77.32
77.00
76.68
73.70
73.43
67.11
65.86
54.80
Ph
O
NH
N
O
Ph
Fe
3c' (di)
f1 (ppm)

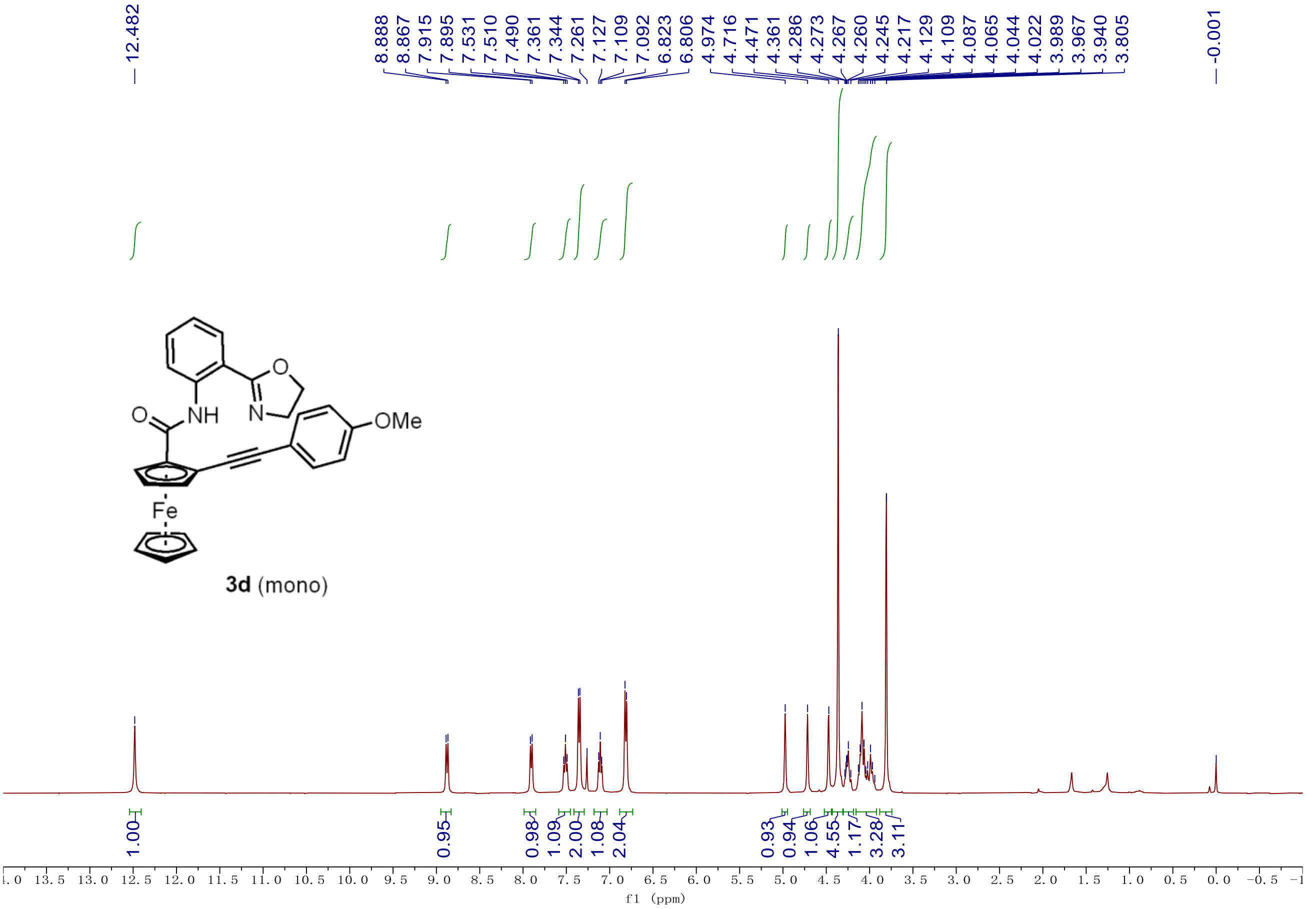
12.482
8.888 8.867 7.915 7.895 7.531 7.510 7.490 7.361 7.344 7.261 7.127 7.109 7.092 6.823 6.806 4.974 4.716 4.471 4.361 4.286 4.273 4.267 4.260 4.245 4.217 4.129 4.109 4.087 4.065 4.044 4.022 3.989 3.967 3.940 3.805
-0.001
O
NH
N
OMe
Fe
3d (mono)
1.00 0.95 0.98 1.09 2.00 1.08 2.04 0.93 0.94 1.06 4.55 1.17 3.28 3.11
f1 (ppm)

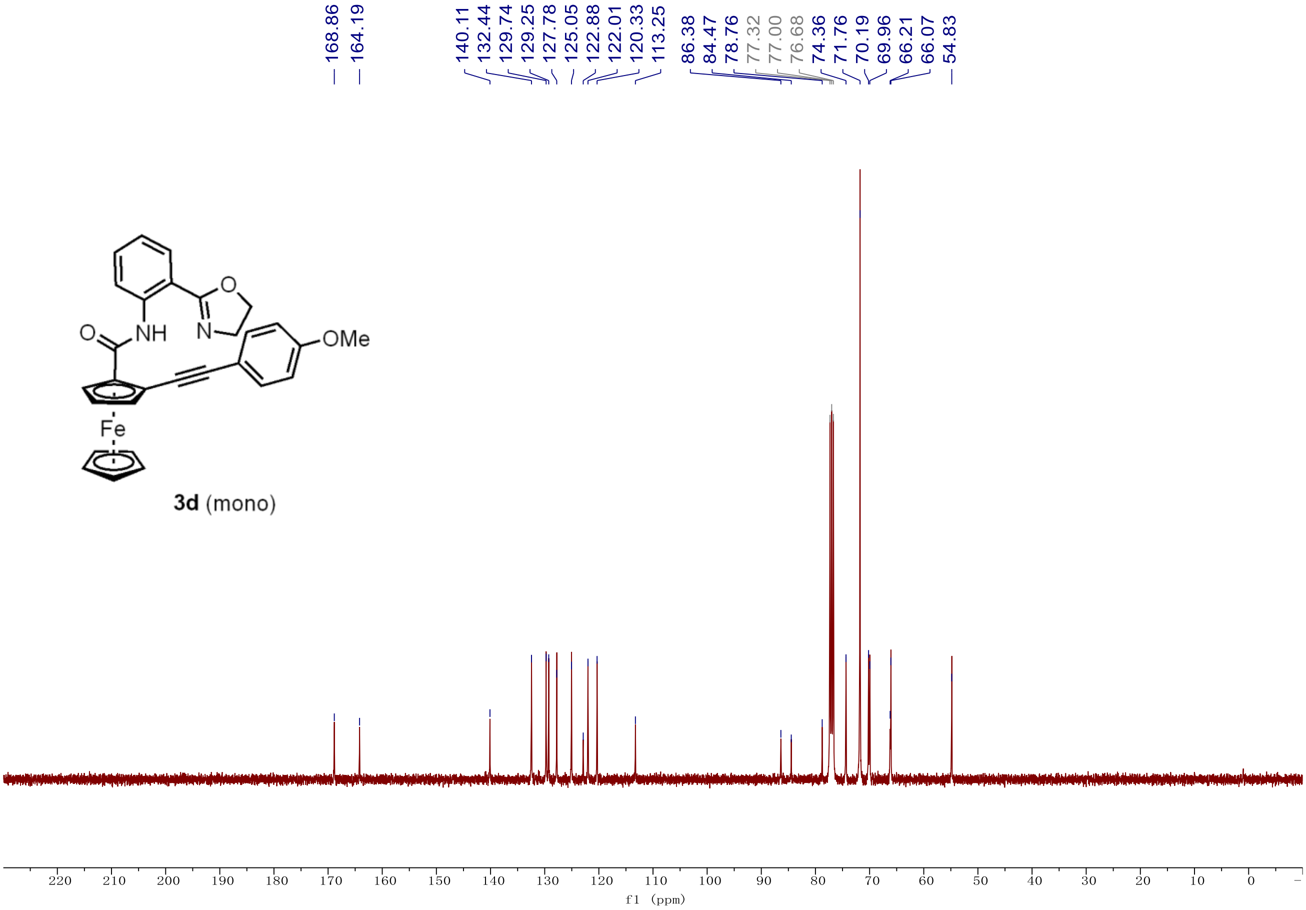
168.86 164.19
140.11 132.44 129.74 129.25 127.78 125.05 122.88 122.01 120.33 113.25
86.38 84.47 78.76 77.32 77.00 76.68 74.36 71.76 70.19 69.96 66.21 66.07
54.83
O
NH
N
OMe
Fe
3d (mono)
f1 (ppm)

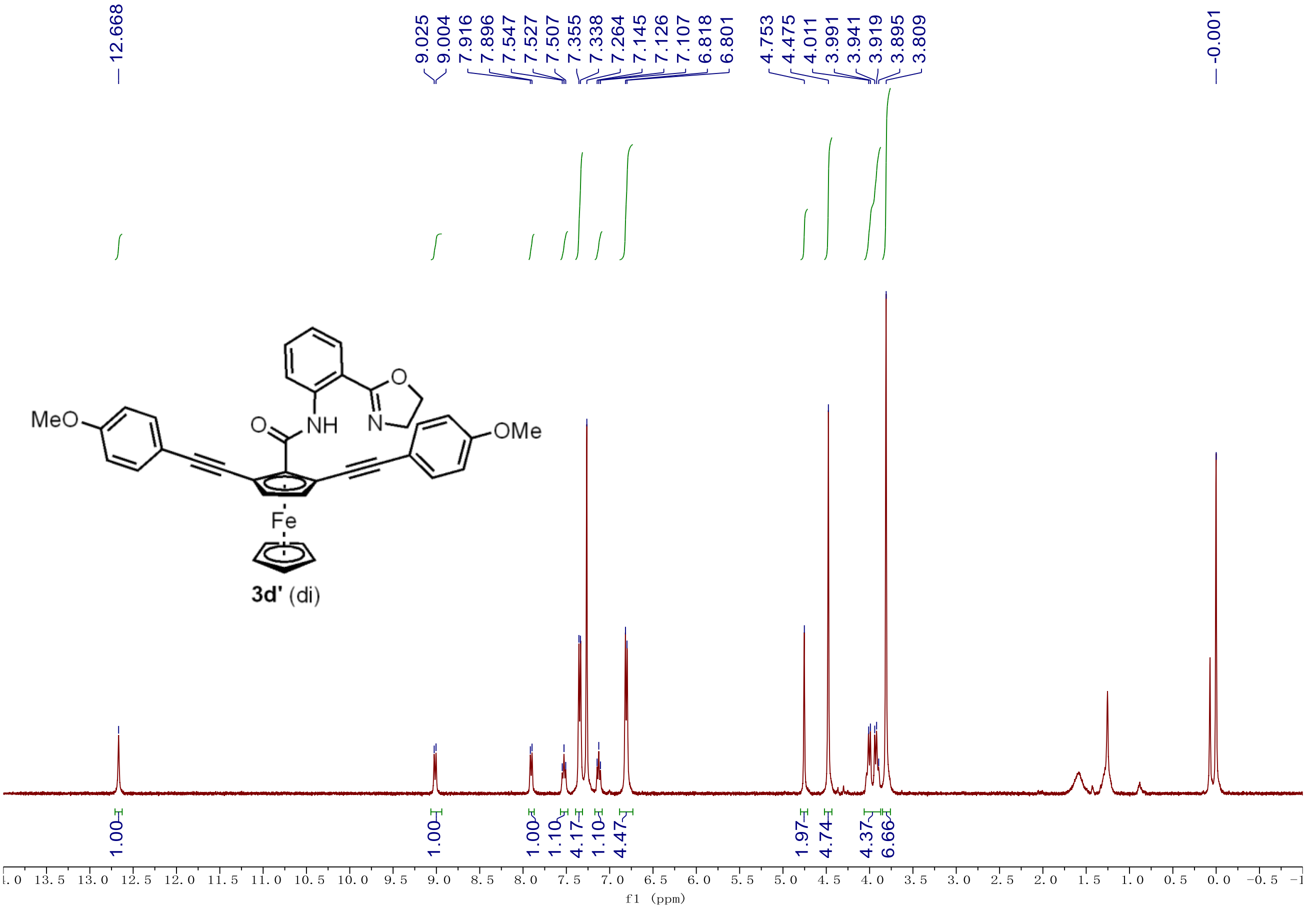
12.668
9.025
9.004
7.916
7.896
7.547
7.527
7.507
7.355
7.338
7.264
7.145
7.126
7.107
6.818
6.801
4.753
4.475
4.011
3.991
3.941
3.919
3.895
3.809
-0.001
MeO
OMe
O
NH
N
Fe
1.00
1.00
1.00
1.10
4.17
1.10
4.47
1.97
4.74
4.37
6.66
f1 (ppm)

3d' (di)

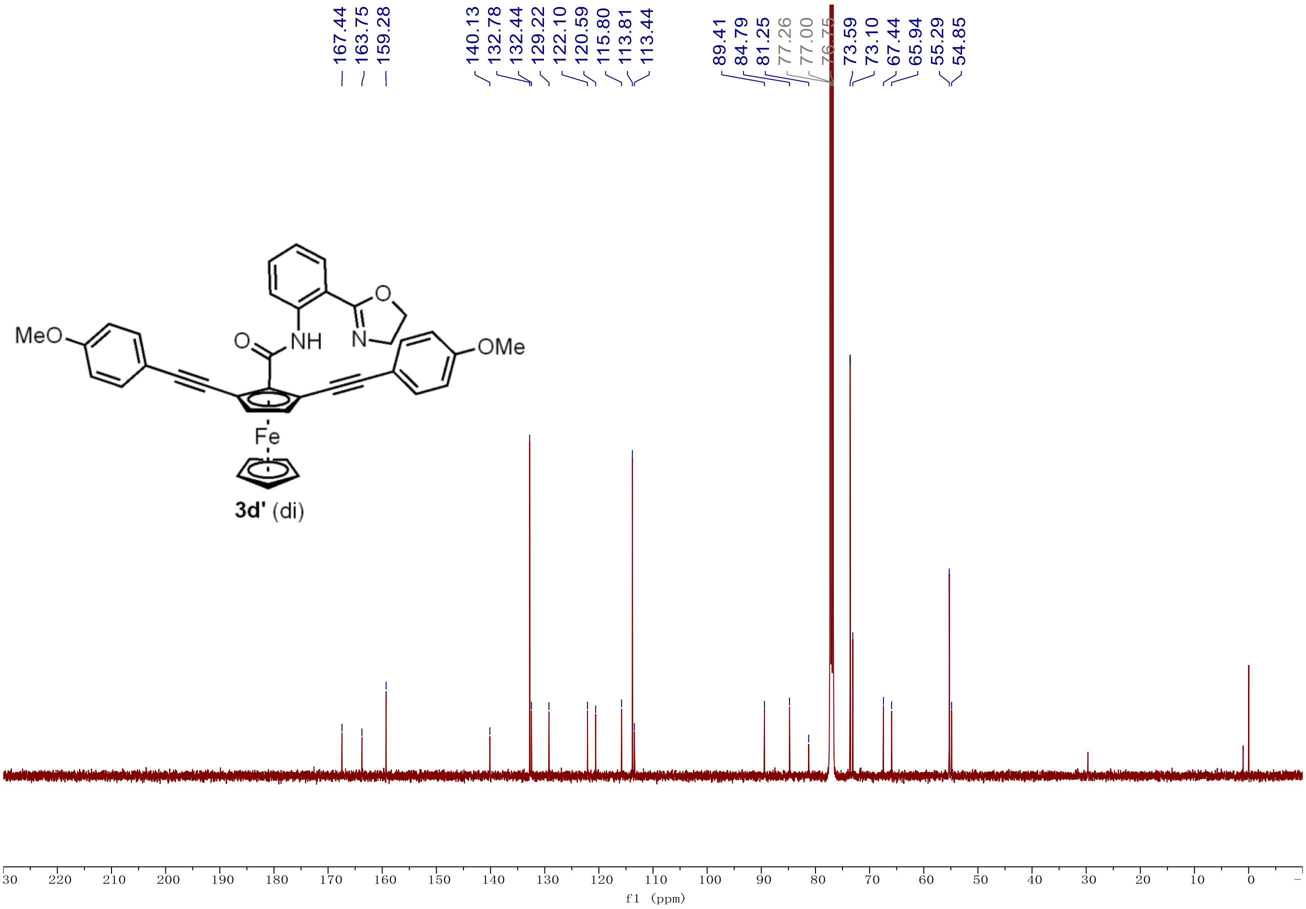
167.44
163.75
159.28
140.13
132.78
132.44
129.22
122.10
120.59
115.80
113.81
113.44
89.41
84.79
81.25
77.26
77.00
76.75
73.59
73.10
67.44
65.94
55.29
54.85
MeO
OMe
O
NH
N
Fe
f1 (ppm)

3d' (di)

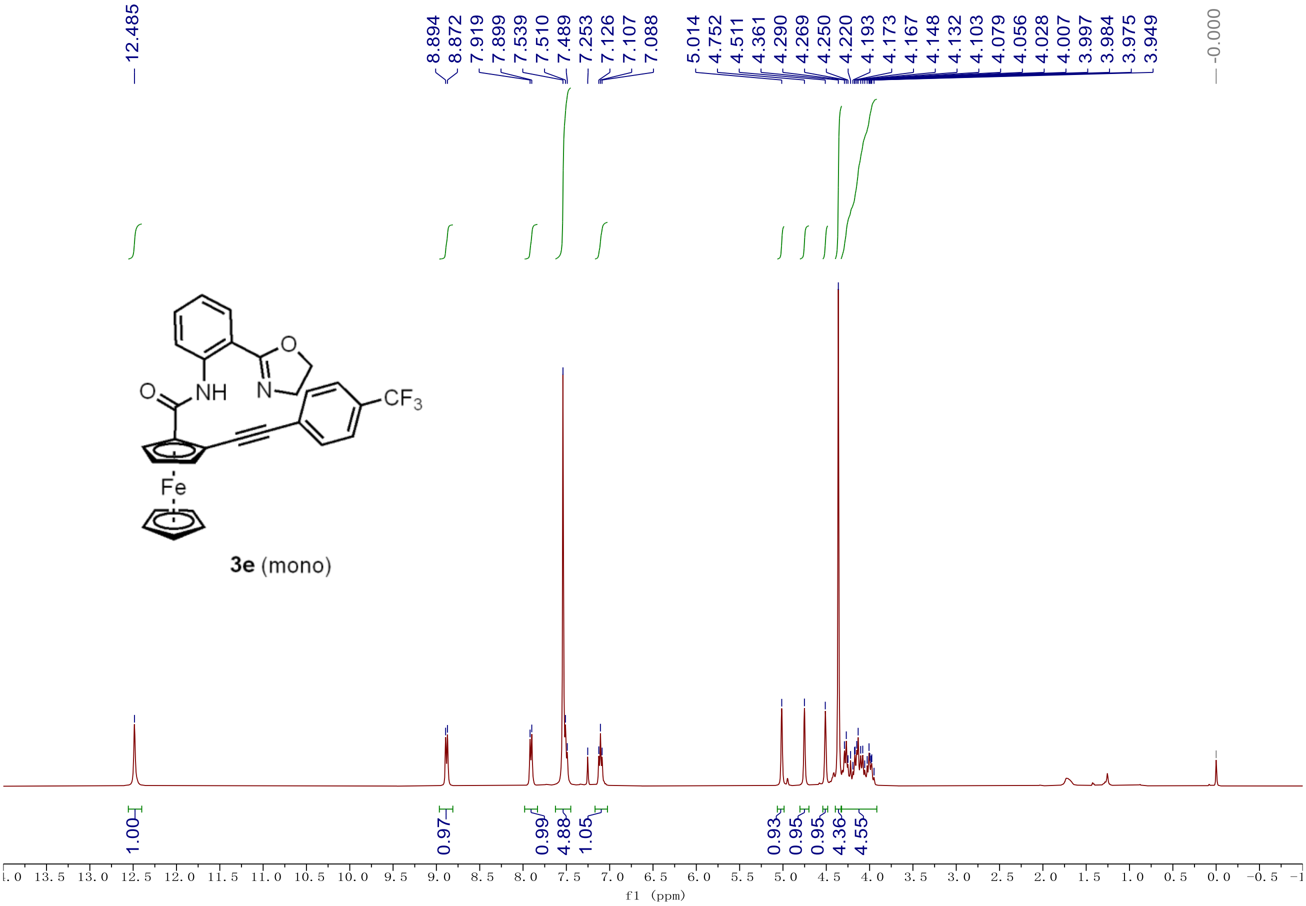
12.485
8.894
8.872
7.919
7.899
7.539
7.510
7.489
7.253
7.126
7.107
7.088
5.014
4.752
4.511
4.361
4.290
4.269
4.250
4.220
4.193
4.173
4.167
4.148
4.132
4.103
4.079
4.056
4.028
4.007
3.997
3.984
3.975
3.949
-0.000
3e (mono)
1.00
0.97
0.99
4.88
1.05
0.93
0.95
0.95
4.36
4.55
f1 (ppm)

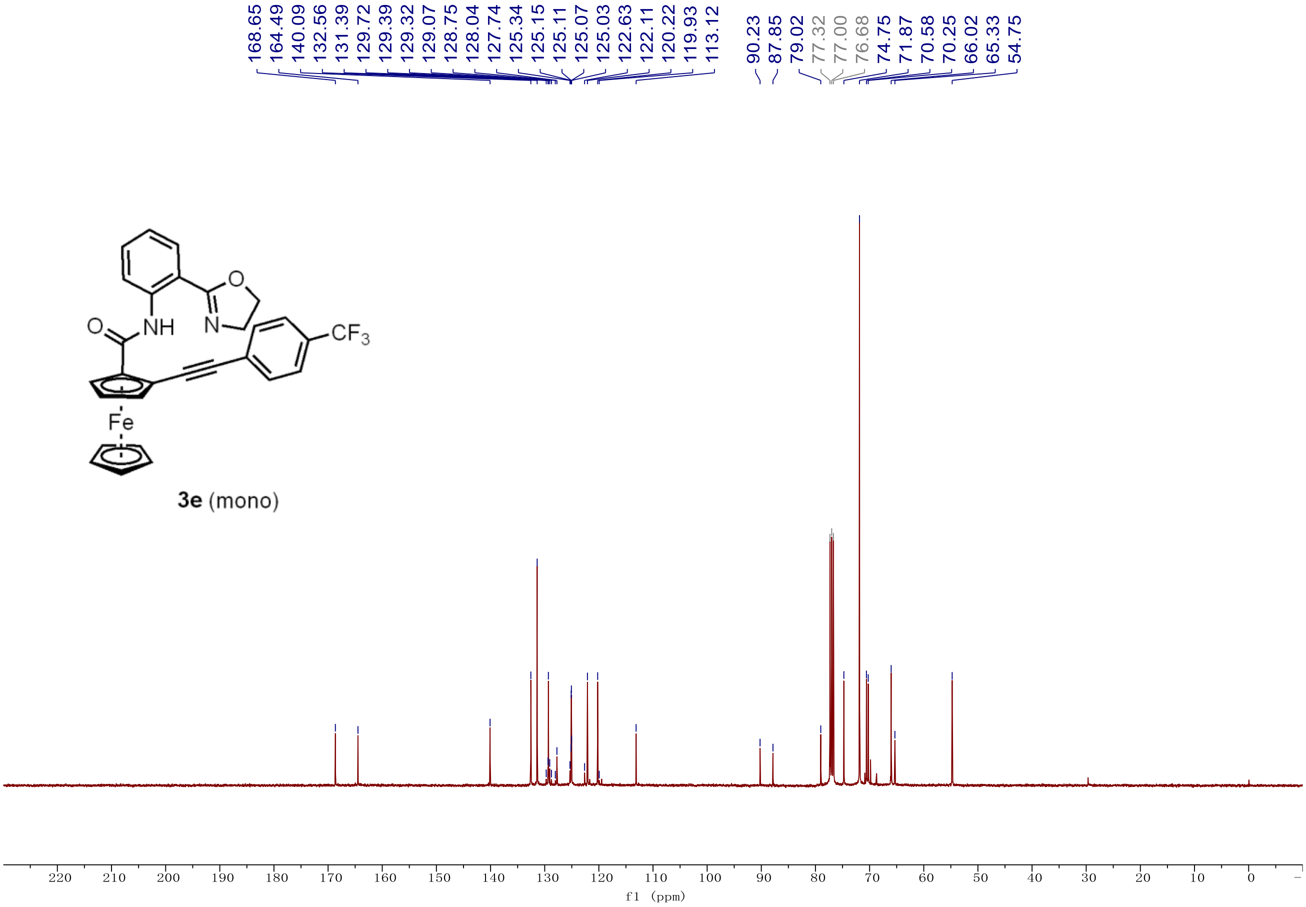
168.65
164.49
140.09
132.56
131.39
129.72
129.39
129.32
129.07
128.75
128.04
127.74
125.34
125.15
125.11
125.07
125.03
122.63
122.11
120.22
119.93
113.12
90.23
87.85
79.02
77.32
77.00
76.68
74.75
71.87
70.58
70.25
66.02
65.33
54.75
3e (mono)
f1 (ppm)

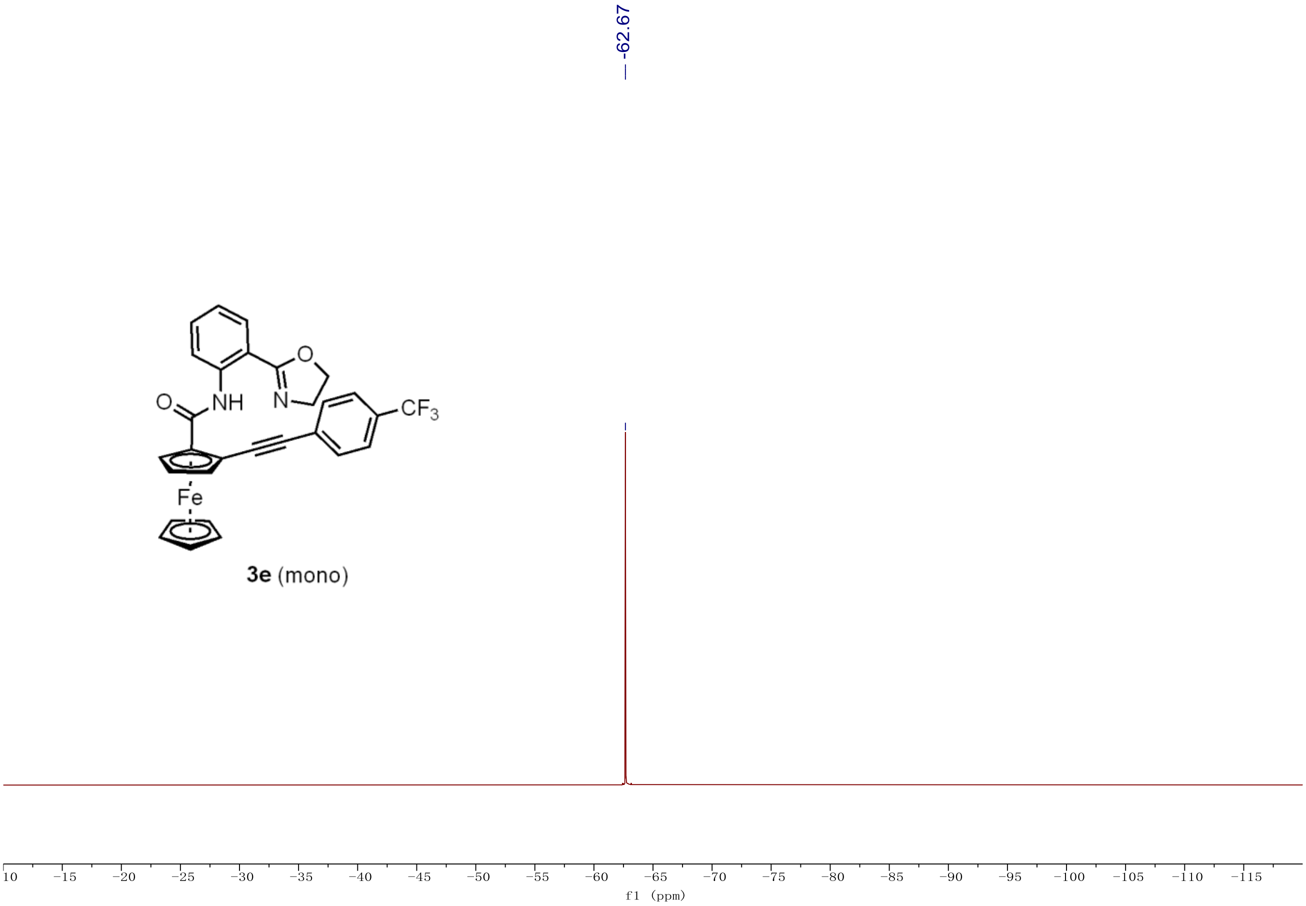
-62.67
CF3
O
NH
N
O
Fe
3e (mono)
f1 (ppm)

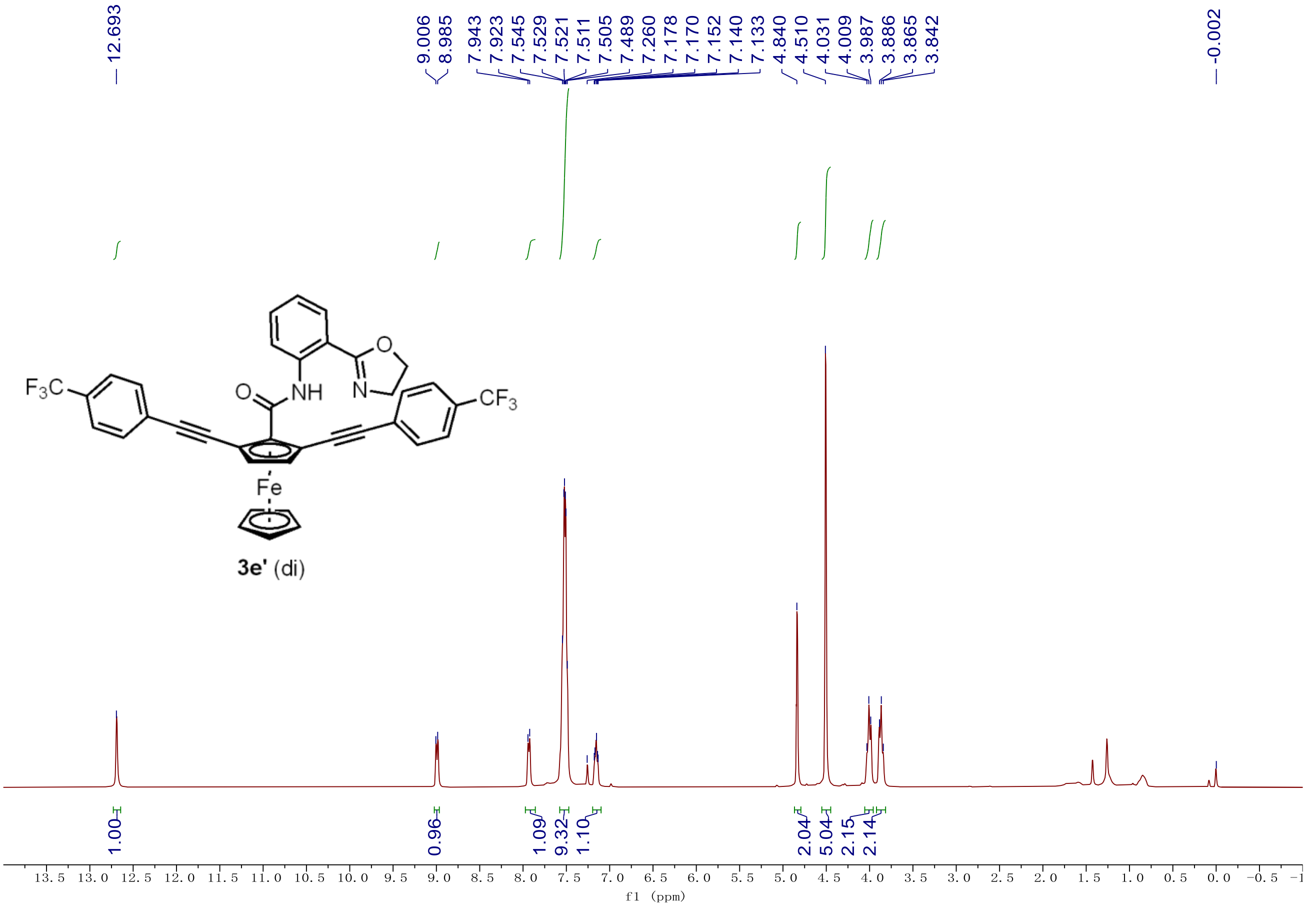
12.693
9.006
8.985
7.943
7.923
7.545
7.529
7.521
7.511
7.505
7.489
7.260
7.178
7.170
7.152
7.140
7.133
4.840
4.510
4.031
4.009
3.987
3.886
3.865
3.842
-0.002
F3C
CF3
O
NH
N
O
Fe
3e' (di)
1.00
0.96
1.09
9.32
1.10
2.04
5.04
2.15
2.14
f1 (ppm)

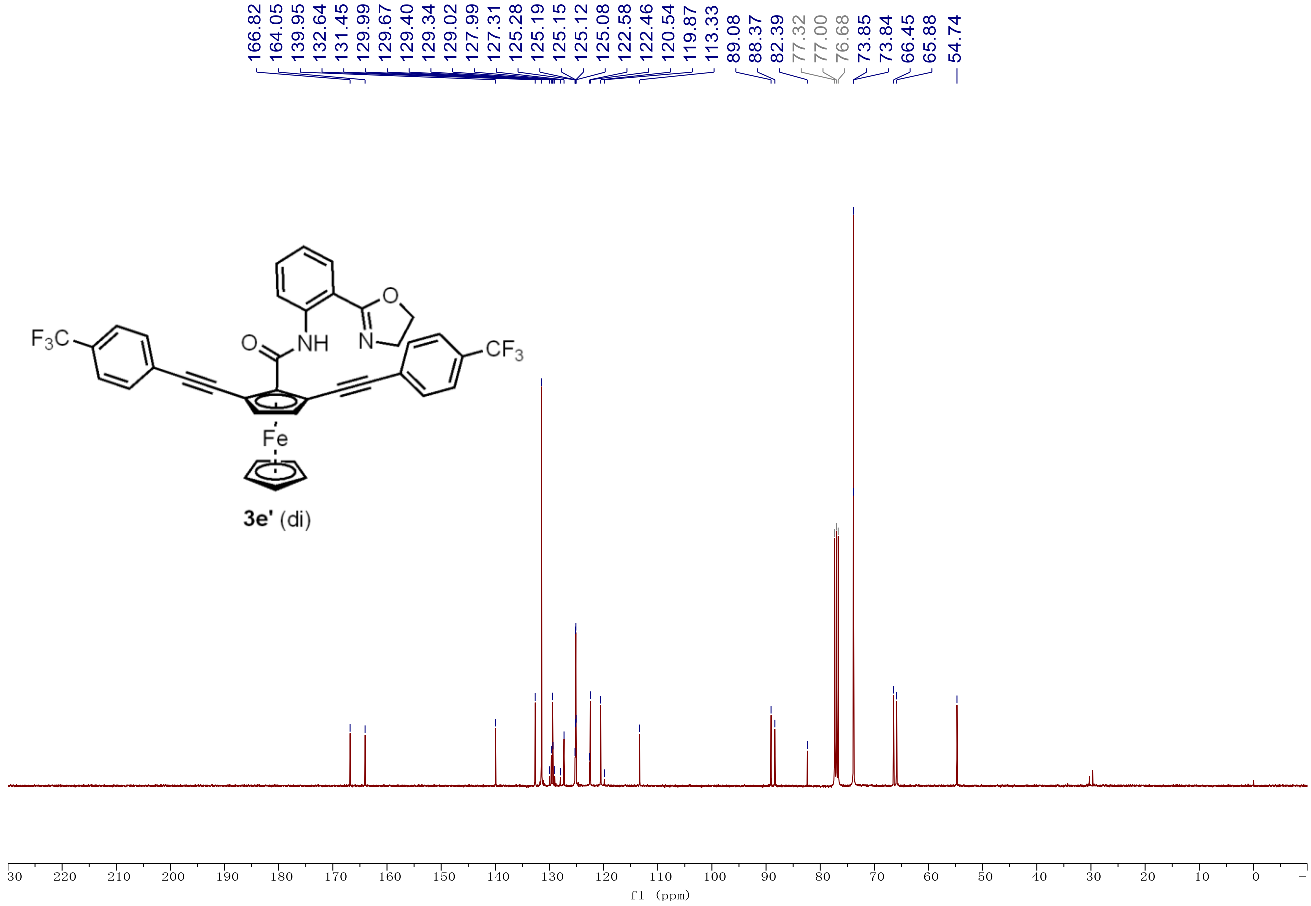
166.82 164.05 139.95 132.64 131.45 129.99 129.67 129.40 129.34 129.02 127.99 127.31 125.28 125.19 125.15 125.12 125.08 122.58 122.46 120.54 119.87 113.33 89.08 88.37 82.39 77.32 77.00 76.68 73.85 73.84 66.45 65.88 54.74
F3C
CF3
O
NH
N
Fe
3e' (di)
f1 (ppm)

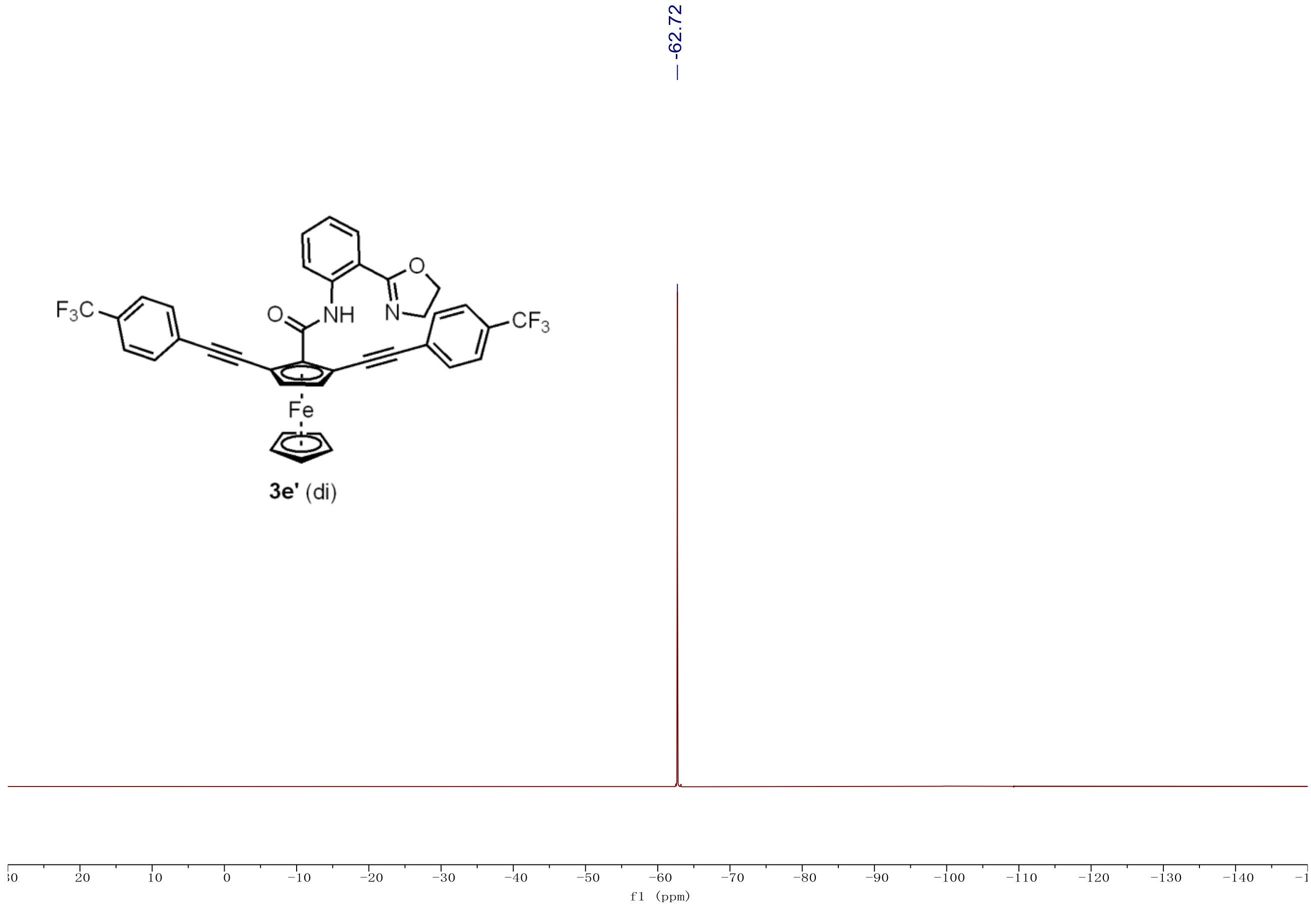
-62.72
F3C
CF3
O
NH
N
Fe
3e' (di)
f1 (ppm)

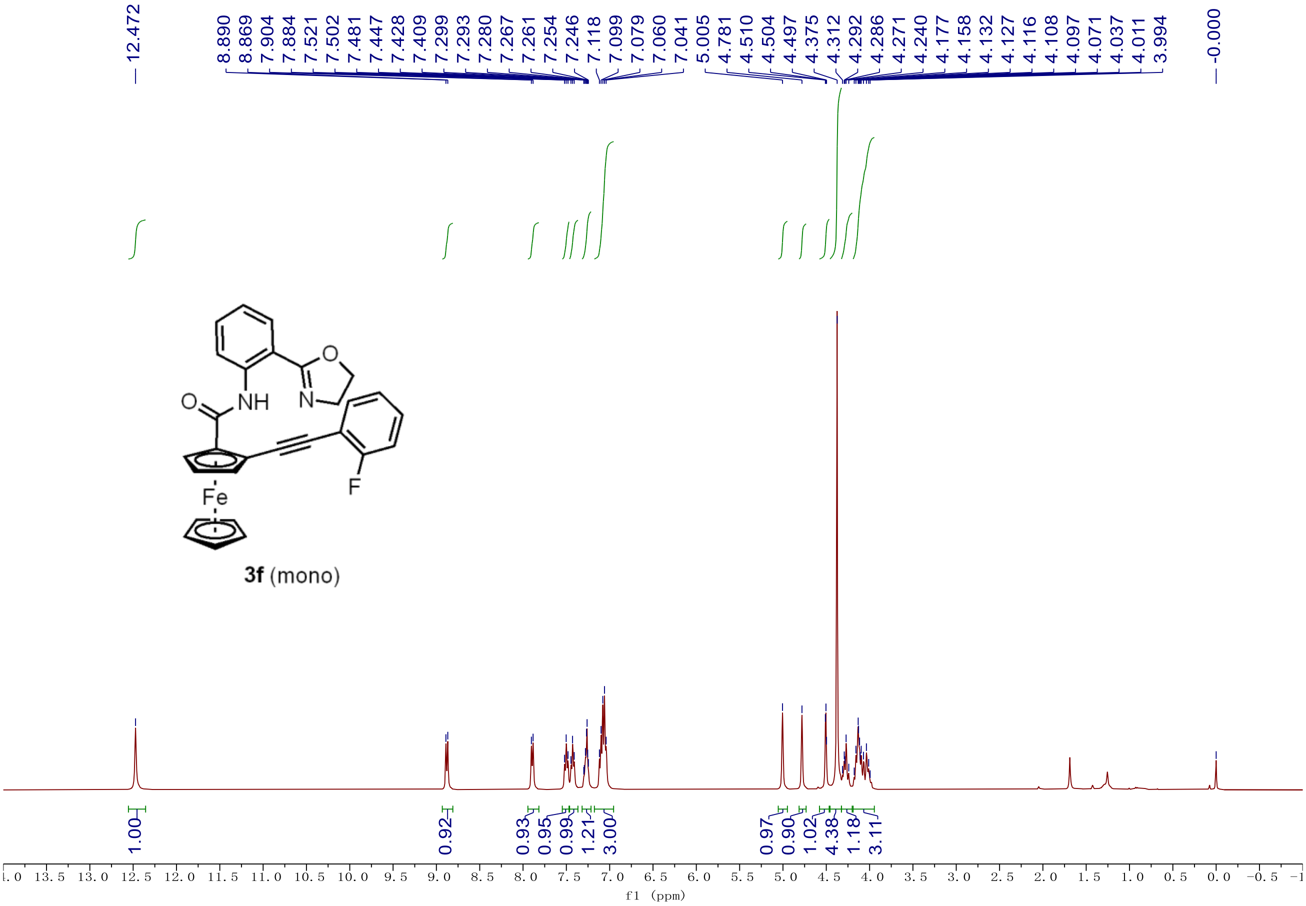

12.472
8.890
8.869
7.904
7.884
7.521
7.502
7.481
7.447
7.428
7.409
7.299
7.293
7.280
7.267
7.261
7.254
7.246
7.118
7.099
7.079
7.060
7.041
5.005
4.781
4.510
4.504
4.497
4.375
4.312
4.292
4.286
4.271
4.240
4.177
4.158
4.132
4.127
4.116
4.108
4.097
4.071
4.037
4.011
3.994
-0.000
NH
O
N
O
Fe
F
3f (mono)
1.00
0.92
0.93
0.95
0.99
1.21
3.00
0.97
0.90
1.02
4.38
1.18
3.11
f1 (ppm)

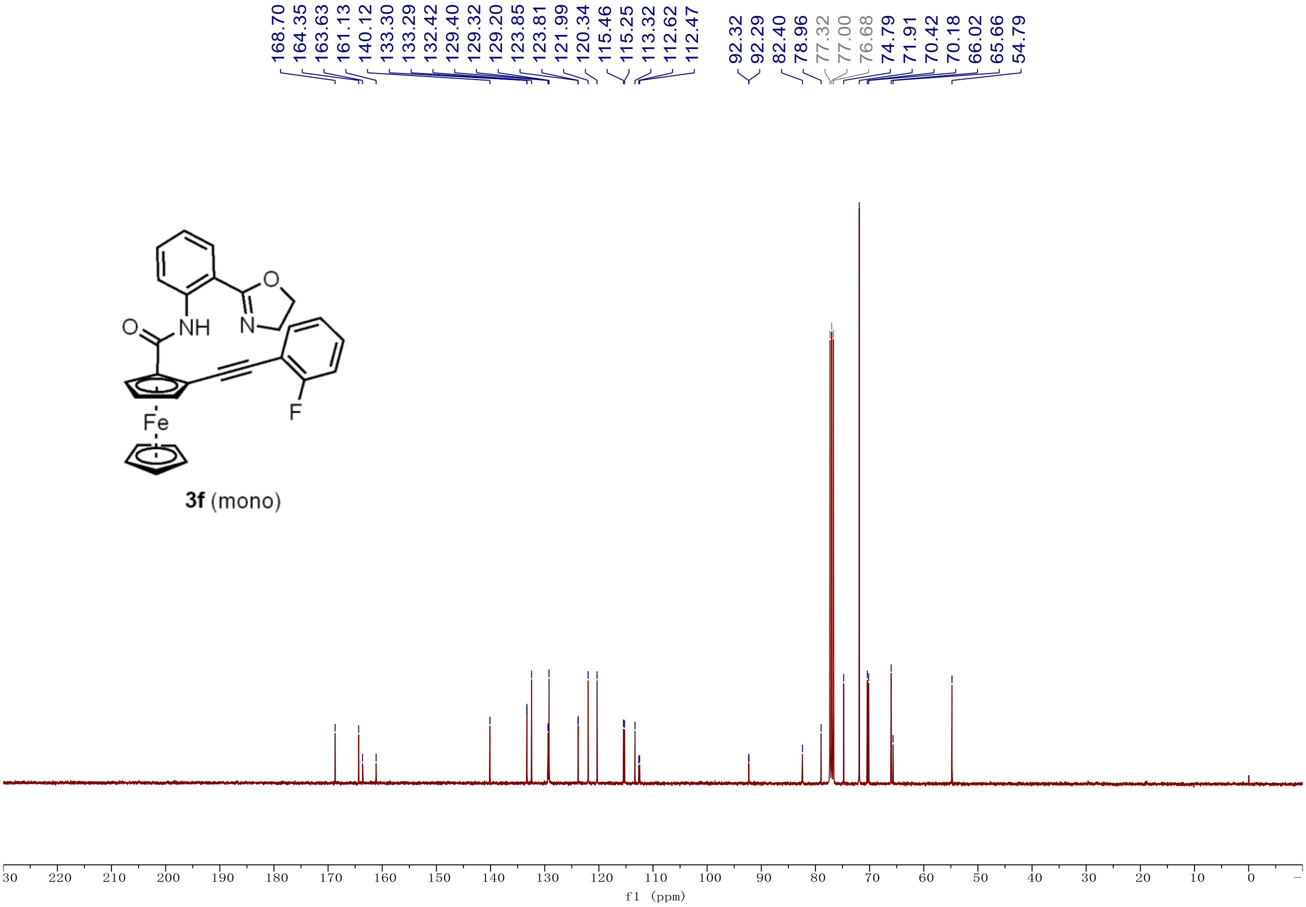

168.70
164.35
163.63
161.13
140.12
133.30
133.29
132.42
129.40
129.32
129.20
123.85
123.81
121.99
120.34
115.46
115.25
113.32
112.62
112.47
92.32
92.29
82.40
78.96
77.32
77.00
76.68
74.79
71.91
70.42
70.18
66.02
65.66
54.79
NH
O
N
O
Fe
F
3f (mono)
f1 (ppm)

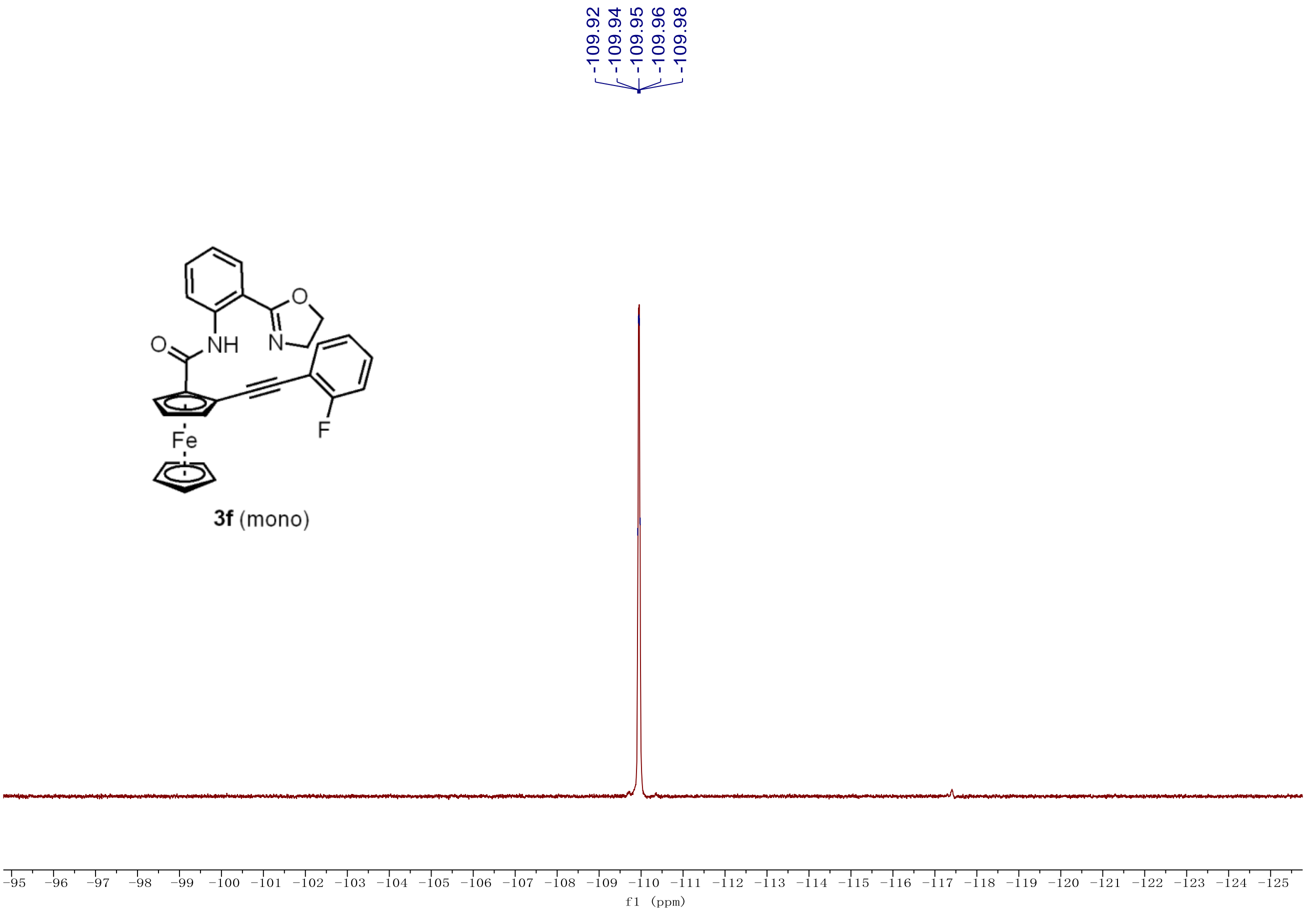
-109.92
-109.94
-109.95
-109.96
-109.98
3f (mono)
f1 (ppm)

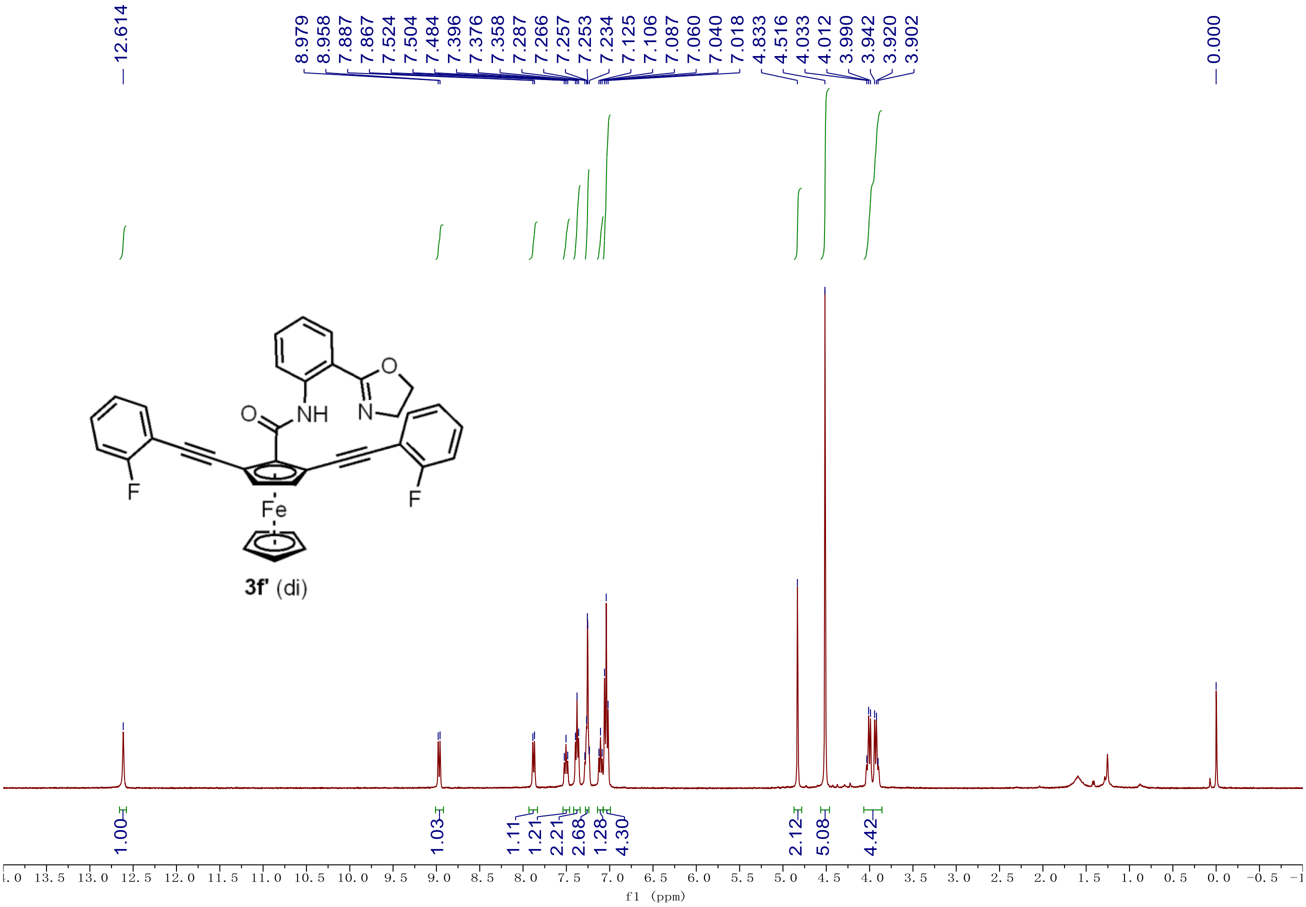
12.614
8.979
8.958
7.887
7.867
7.524
7.504
7.484
7.396
7.376
7.358
7.287
7.266
7.257
7.253
7.234
7.125
7.106
7.087
7.060
7.040
7.018
4.833
4.516
4.033
4.012
3.990
3.942
3.920
3.902
0.000
3f' (di)
1.00
1.03
1.11
1.21
2.21
2.68
1.28
4.30
2.12
5.08
4.42
f1 (ppm)

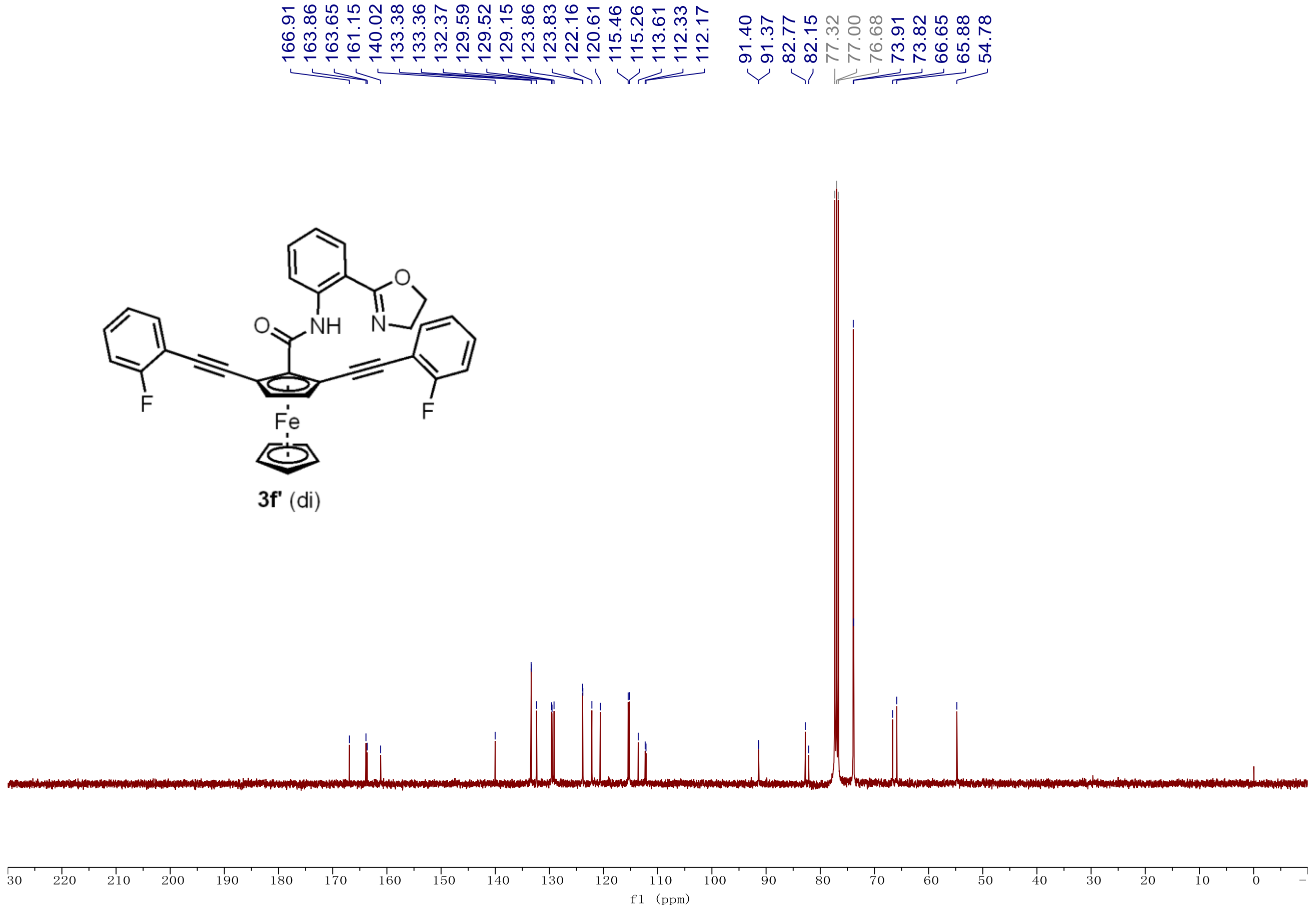
166.91
163.86
163.65
161.15
140.02
133.38
133.36
132.37
129.59
129.52
129.15
123.86
123.83
122.16
120.61
115.46
115.26
113.61
112.33
112.17
91.40
91.37
82.77
82.15
77.32
77.00
76.68
73.91
73.82
66.65
65.88
54.78
NH
O
N
Fe
F
3f' (di)
f1 (ppm)

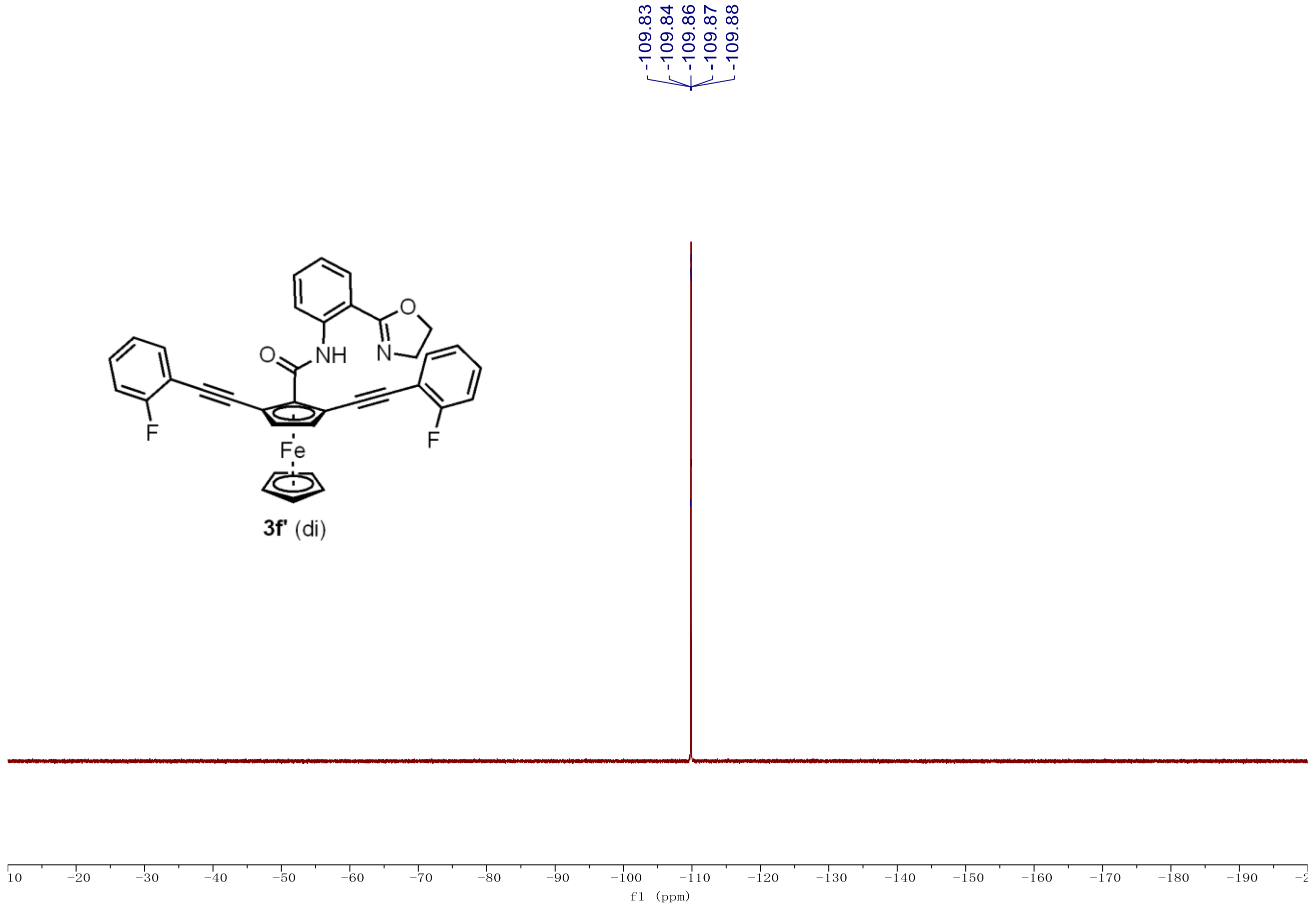
-109.83
-109.84
-109.86
-109.87
-109.88
NH
O
N
Fe
F
3f' (di)
f1 (ppm)

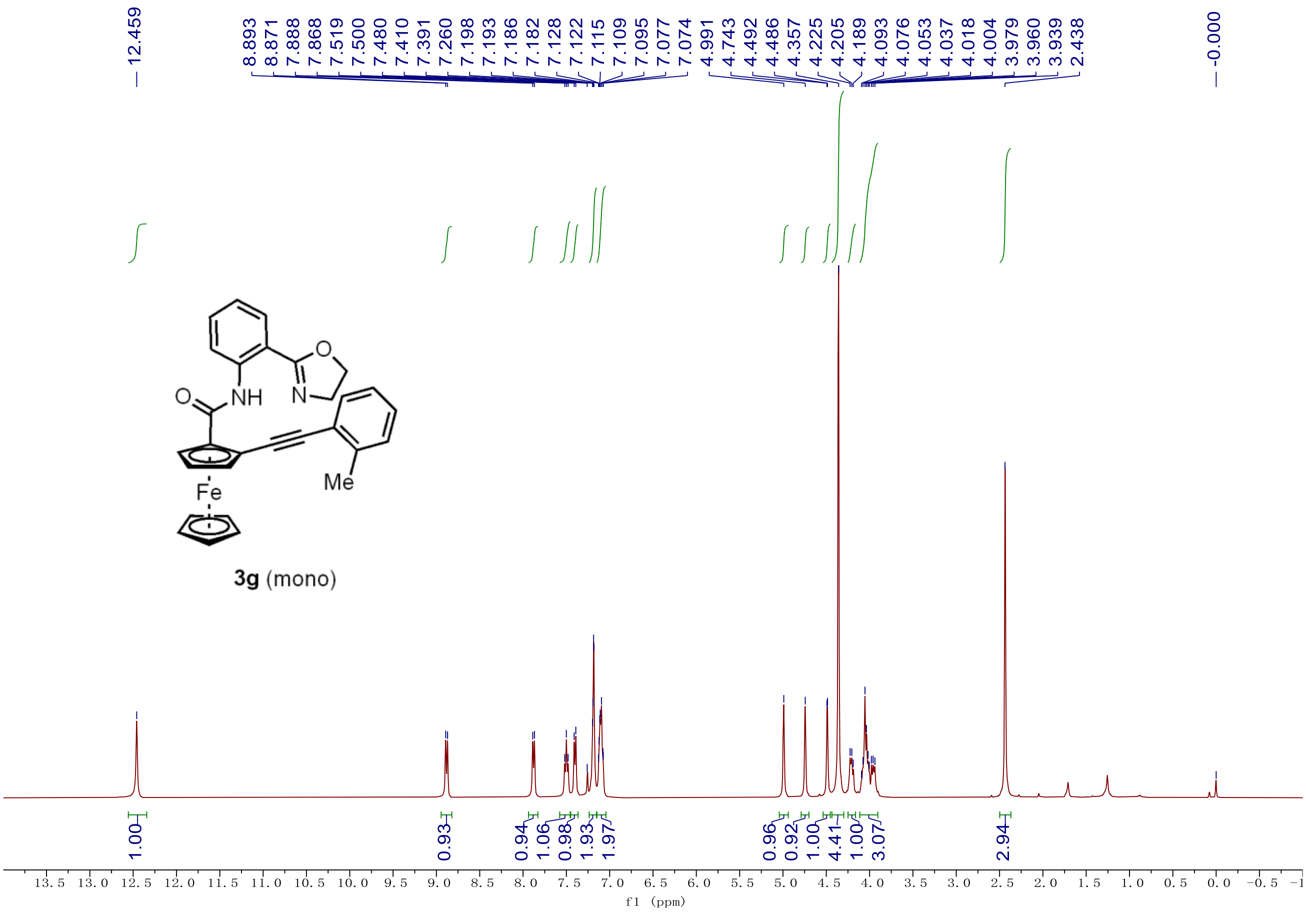
12.459
8.893
8.871
7.888
7.868
7.519
7.500
7.480
7.410
7.391
7.260
7.198
7.193
7.186
7.182
7.128
7.122
7.115
7.109
7.095
7.077
7.074
4.991
4.743
4.492
4.486
4.357
4.225
4.205
4.189
4.093
4.076
4.053
4.037
4.018
4.004
3.979
3.960
3.939
2.438
-0.000
O
NH
N
O
Fe
Me
3g (mono)
1.00
0.93
0.94
1.06
0.98
1.93
1.97
0.96
0.92
1.00
4.41
1.00
3.07
2.94
f1 (ppm)

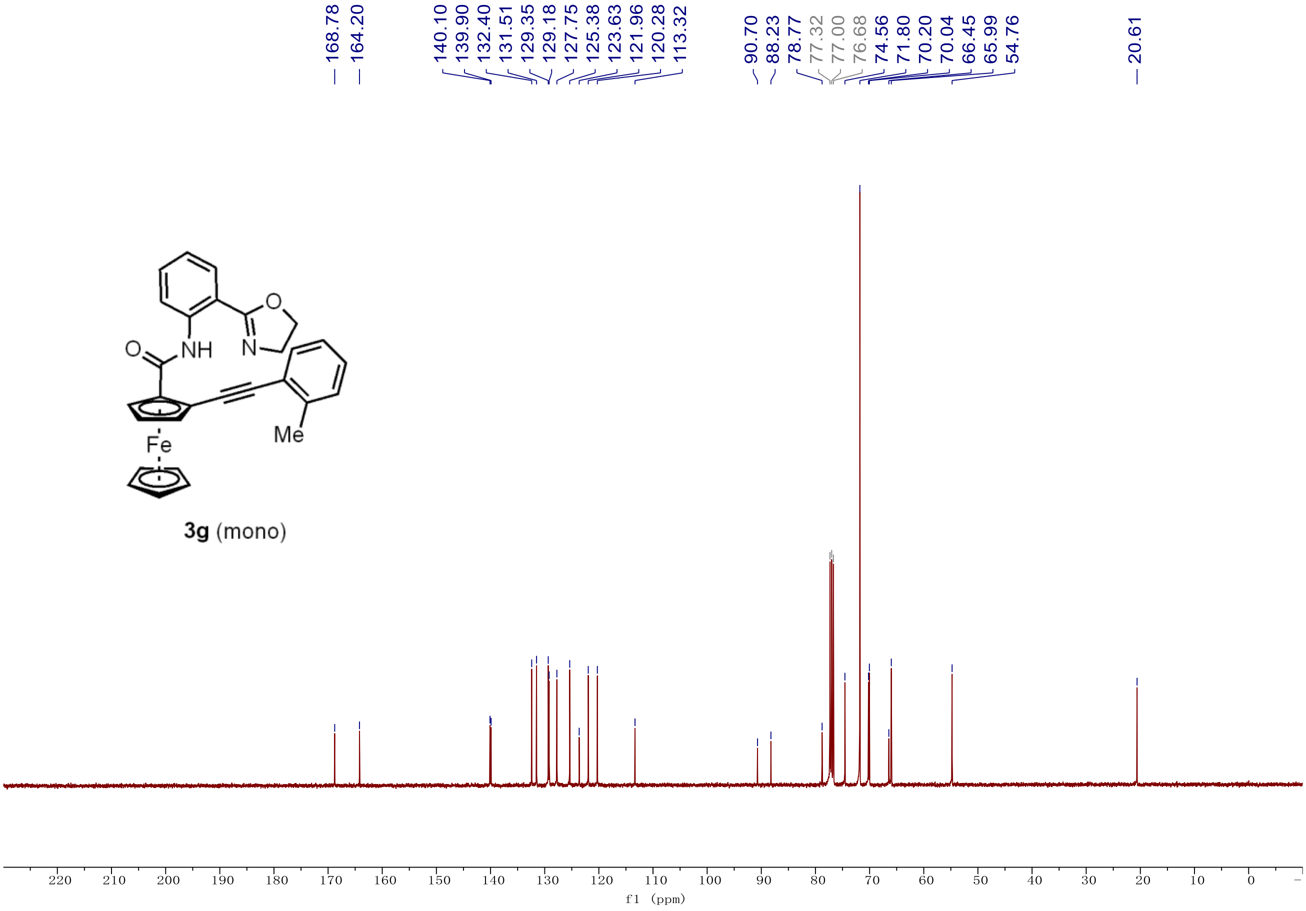
168.78
164.20
140.10
139.90
132.40
131.51
129.35
129.18
127.75
125.38
123.63
121.96
120.28
113.32
90.70
88.23
78.77
77.32
77.00
76.68
74.56
71.80
70.20
70.04
66.45
65.99
54.76
20.61
O
NH
N
O
Fe
Me
3g (mono)
f1 (ppm)

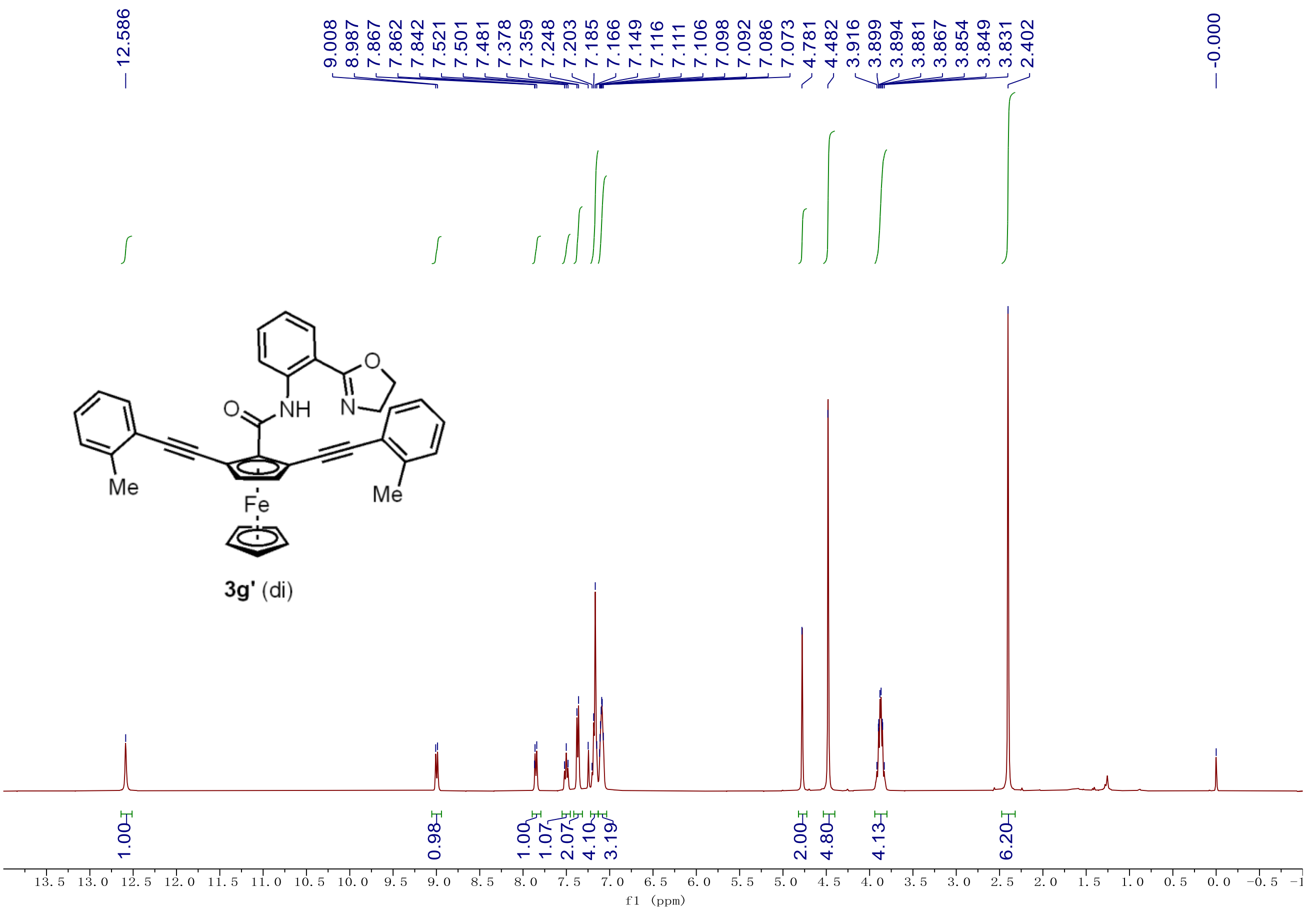
12.586
9.008
8.987
7.867
7.862
7.842
7.521
7.501
7.481
7.378
7.359
7.248
7.203
7.185
7.166
7.149
7.116
7.111
7.106
7.098
7.092
7.086
7.073
4.781
4.482
3.916
3.899
3.894
3.881
3.867
3.854
3.849
3.831
2.402
-0.000
O
NH
N
O
Me
Fe
Me
3g' (di)
1.00
0.98
1.00
1.07
2.07
4.10
3.19
2.00
4.80
4.13
6.20
f1 (ppm)

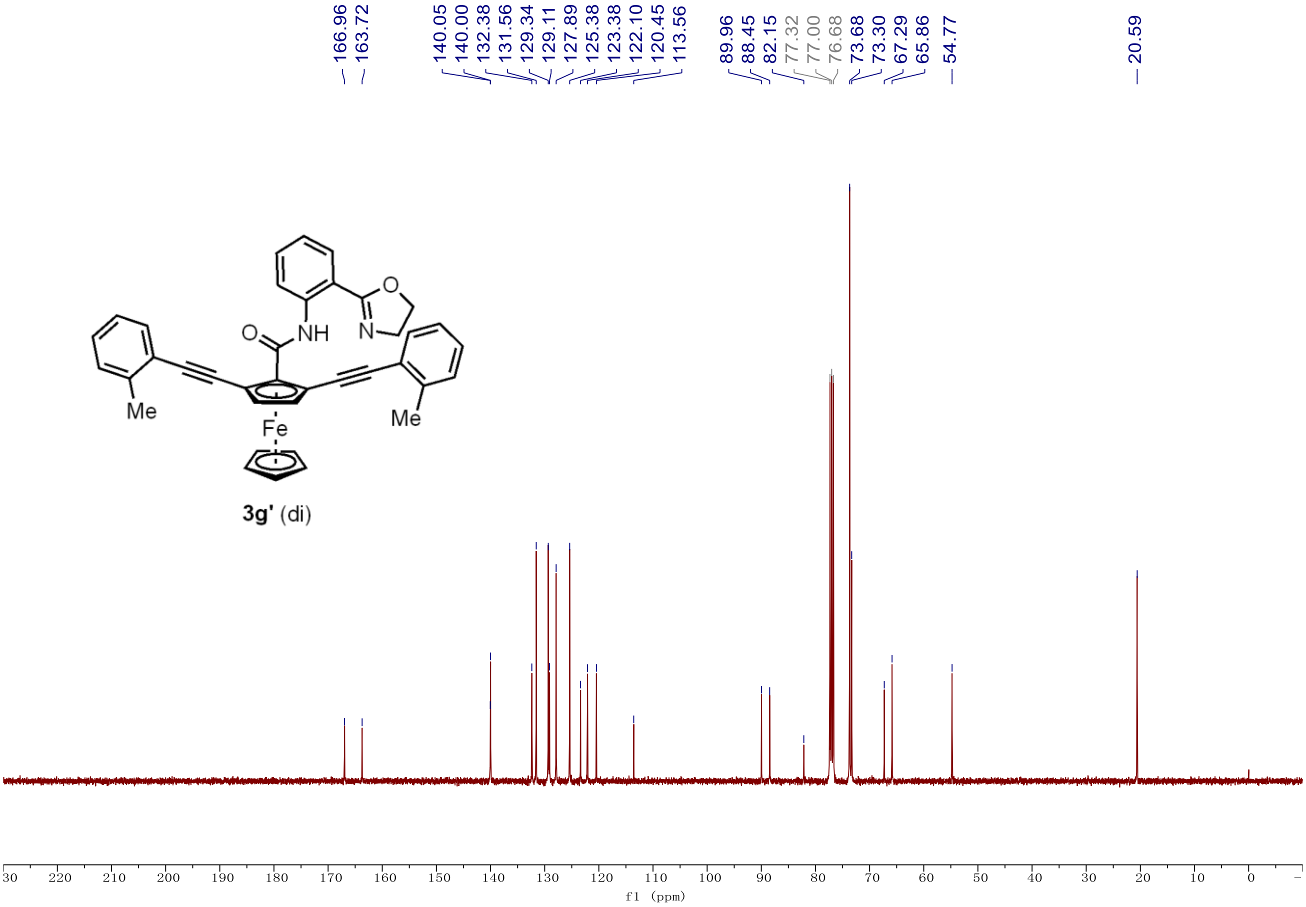
166.96
163.72
140.05
140.00
132.38
131.56
129.34
129.11
127.89
125.38
123.38
122.10
120.45
113.56
89.96
88.45
82.15
77.32
77.00
76.68
73.68
73.30
67.29
65.86
54.77
20.59
O
NH
N
O
Me
Fe
Me
3g' (di)
f1 (ppm)

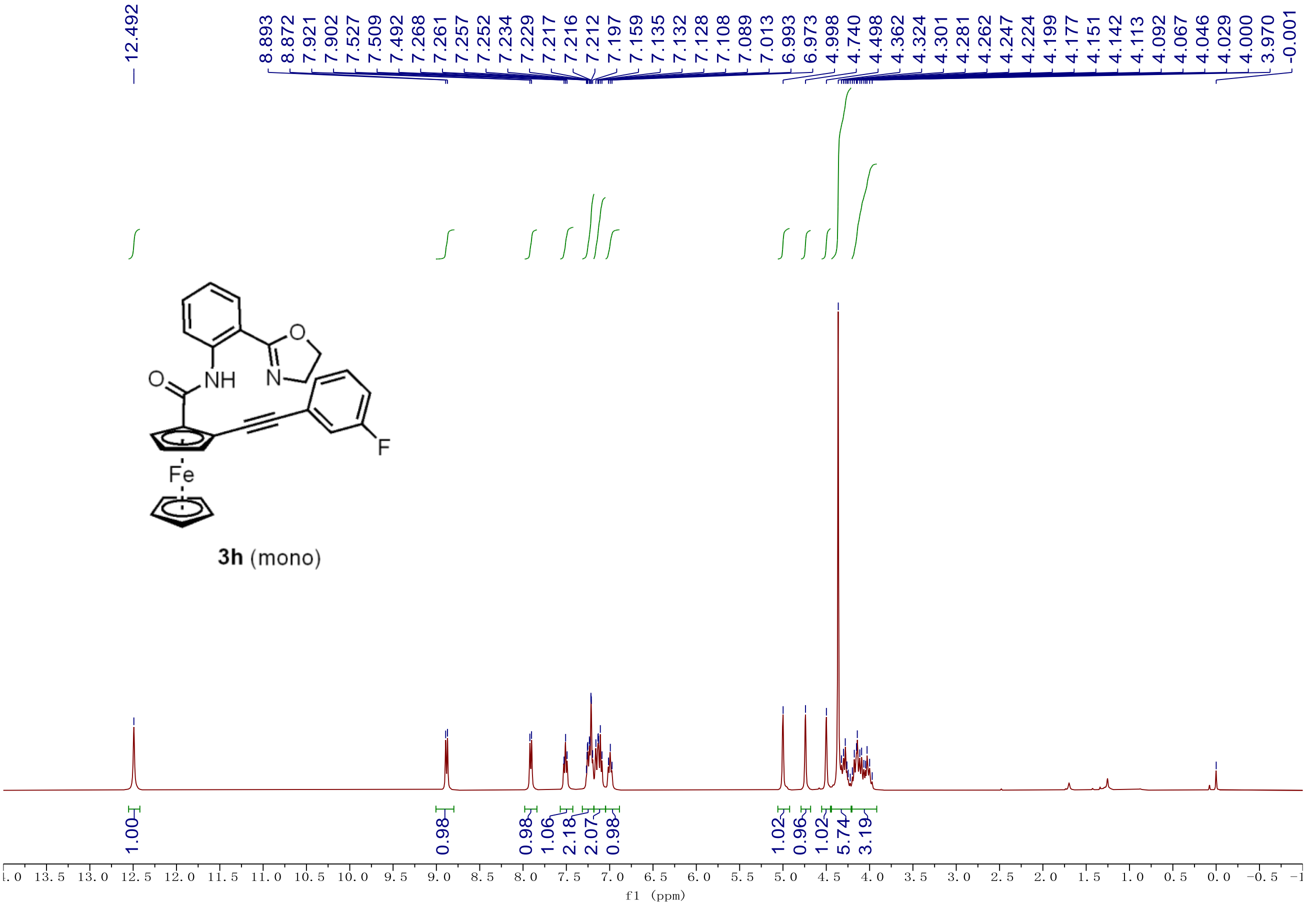

3h (mono)

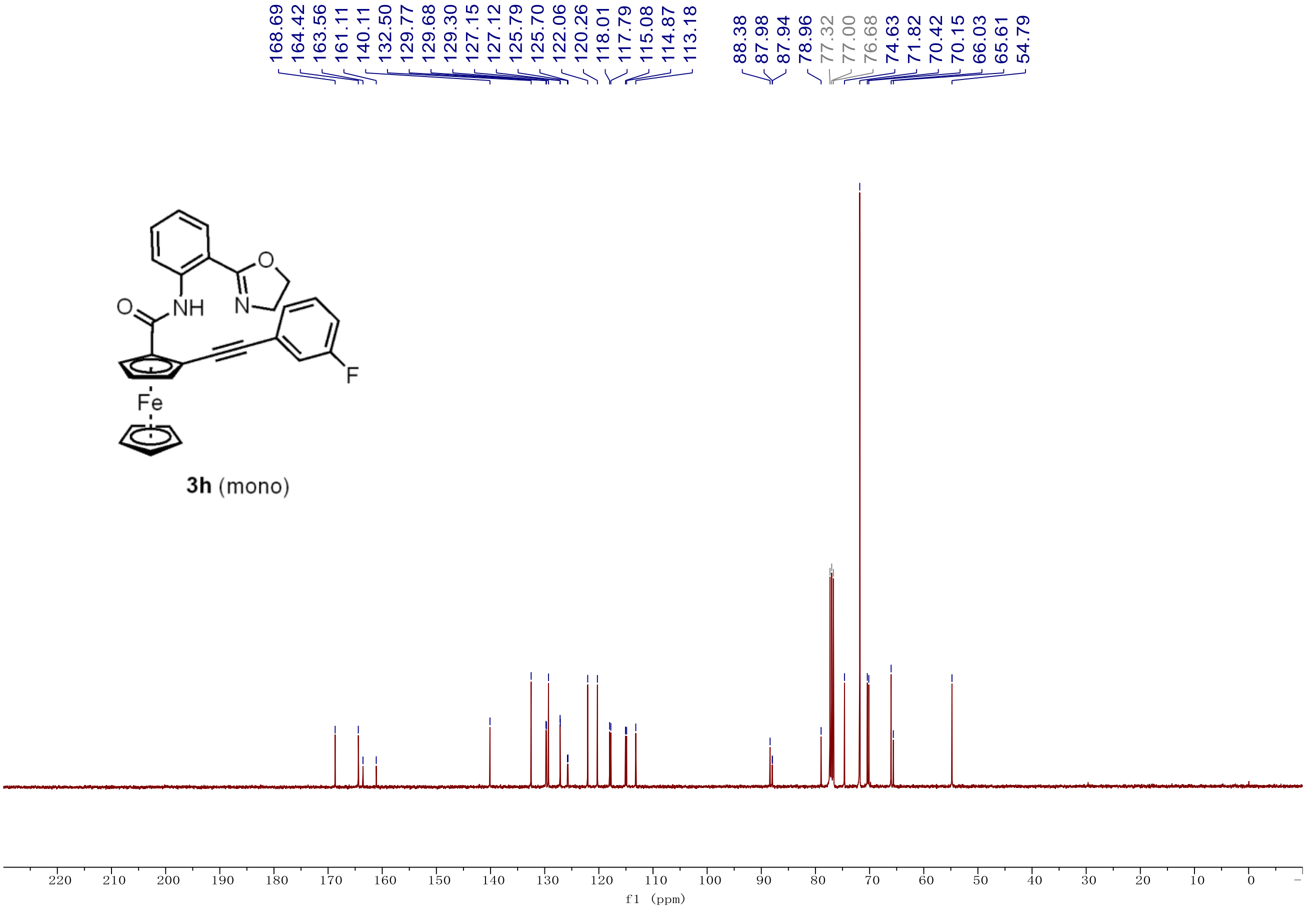

3h (mono)

3h (mono)

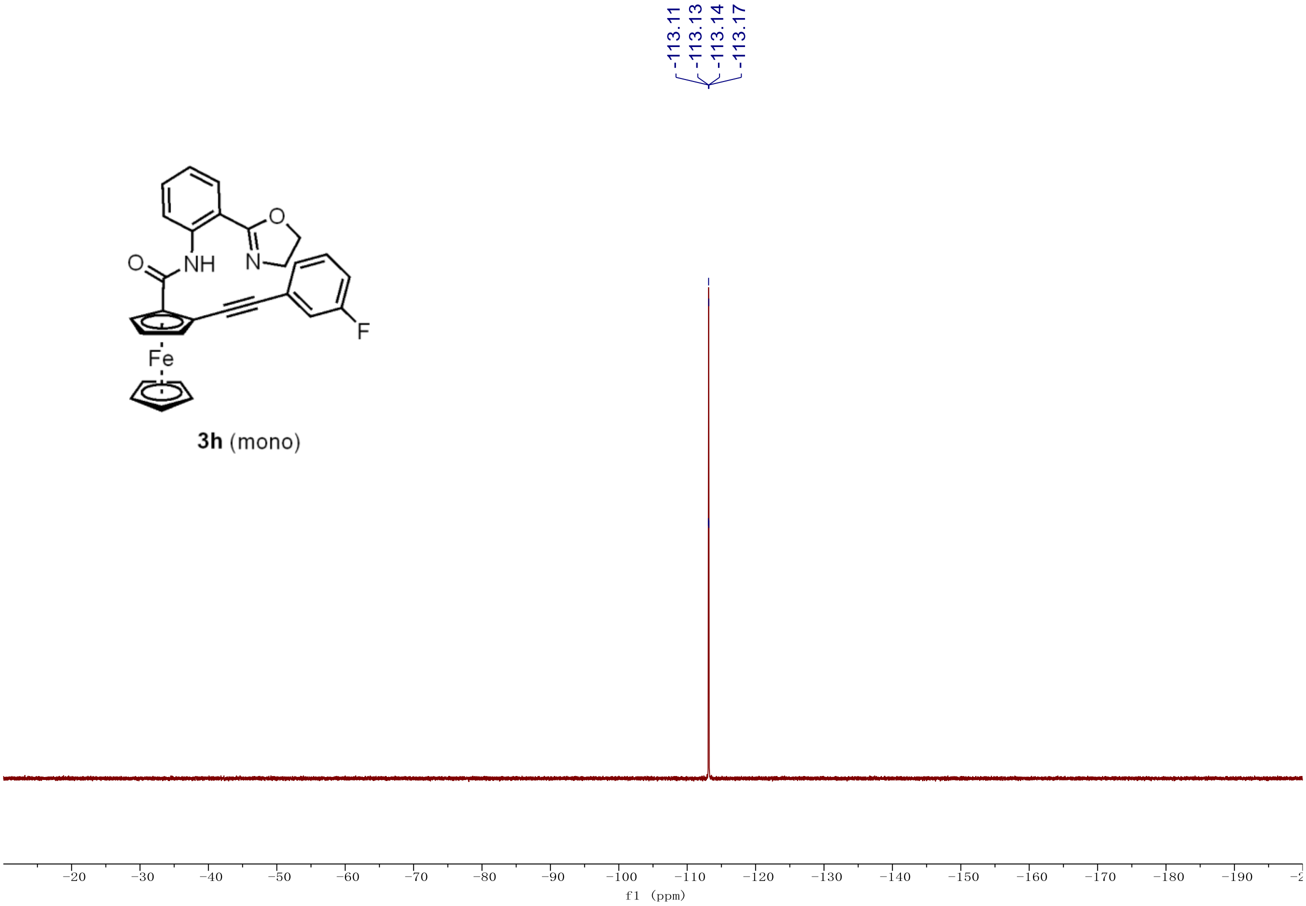
-113.11
-113.13
-113.14
-113.17
f1 (ppm)

3h' (di)

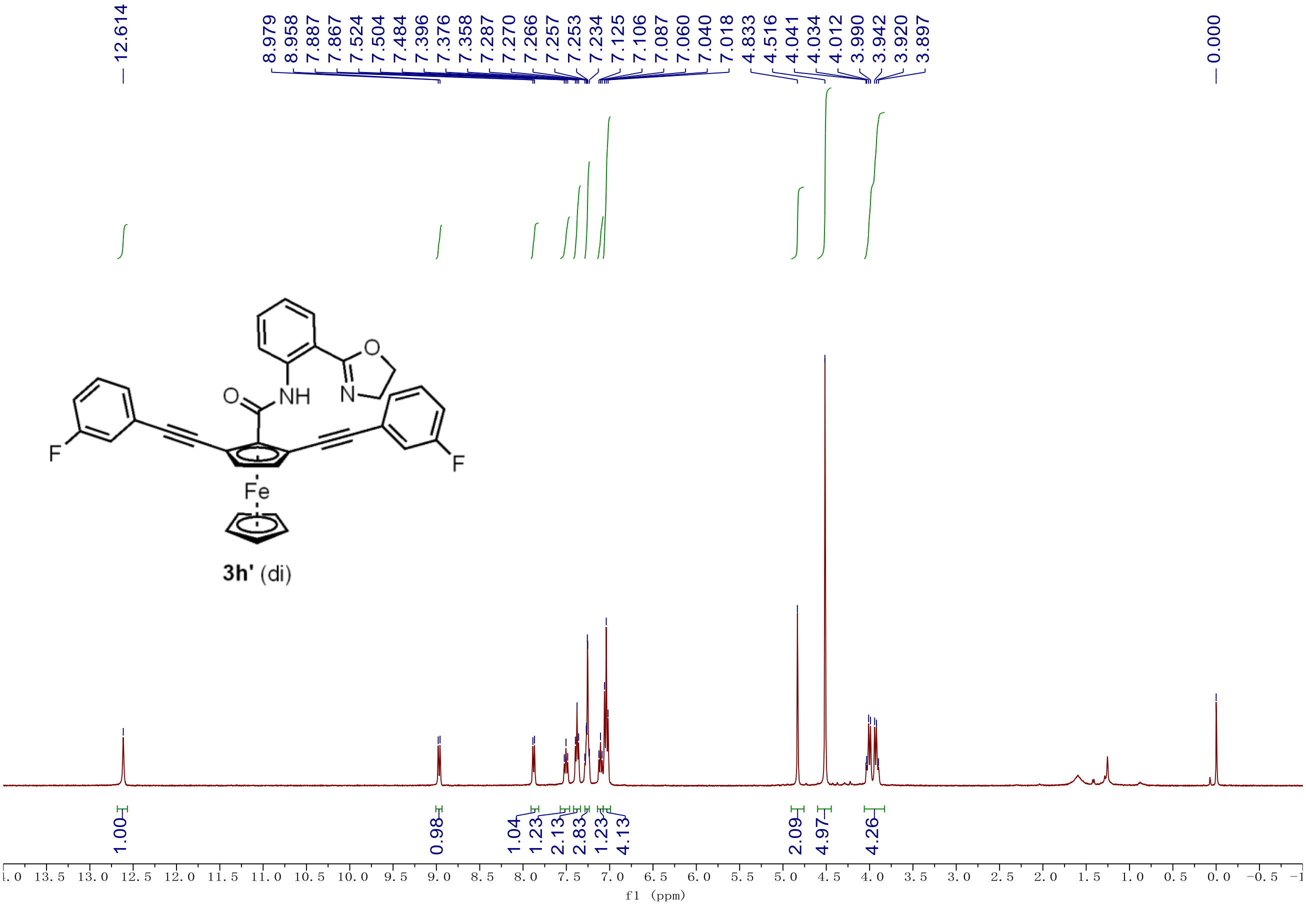
12.614
8.979
8.958
7.887
7.867
7.524
7.504
7.484
7.396
7.376
7.358
7.287
7.270
7.266
7.257
7.253
7.234
7.125
7.106
7.087
7.060
7.040
7.018
4.833
4.516
4.041
4.034
4.012
3.990
3.942
3.920
3.897
0.000
1.00
0.98
1.04
1.23
2.13
2.83
1.23
4.13
2.09
4.97
4.26
f1 (ppm)

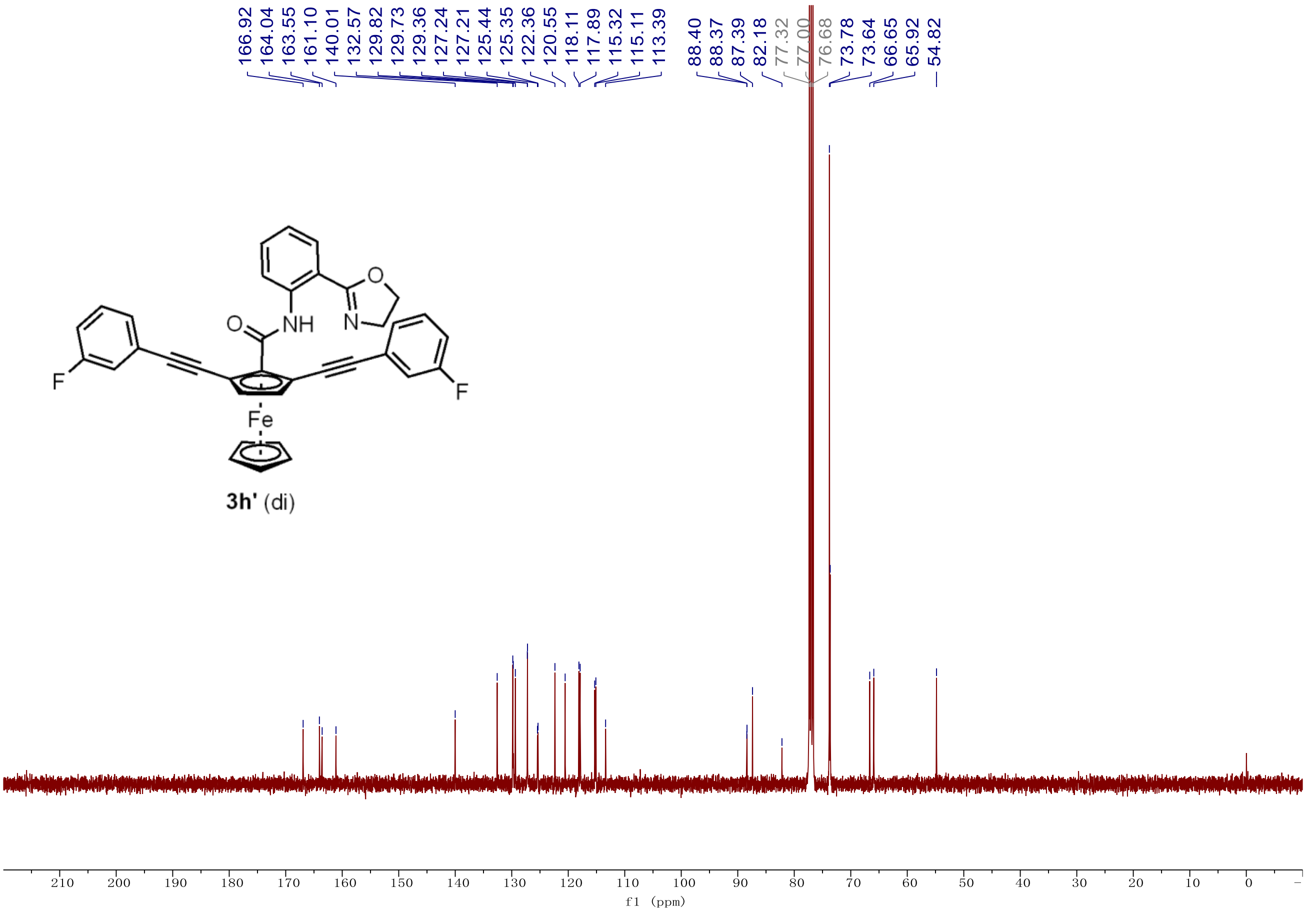
166.92
164.04
163.55
161.10
140.01
132.57
129.82
129.73
129.36
127.24
127.21
125.44
125.35
122.36
120.55
118.11
117.89
115.32
115.11
113.39
88.40
88.37
87.39
82.18
77.32
77.00
76.68
73.78
73.64
66.65
65.92
54.82
O
NH
O
N
F
F
Fe
3h' (di)
210 200 190 180 170 160 150 140 130 120 110 100 90 80 70 60 50 40 30 20 10 0
f1 (ppm)

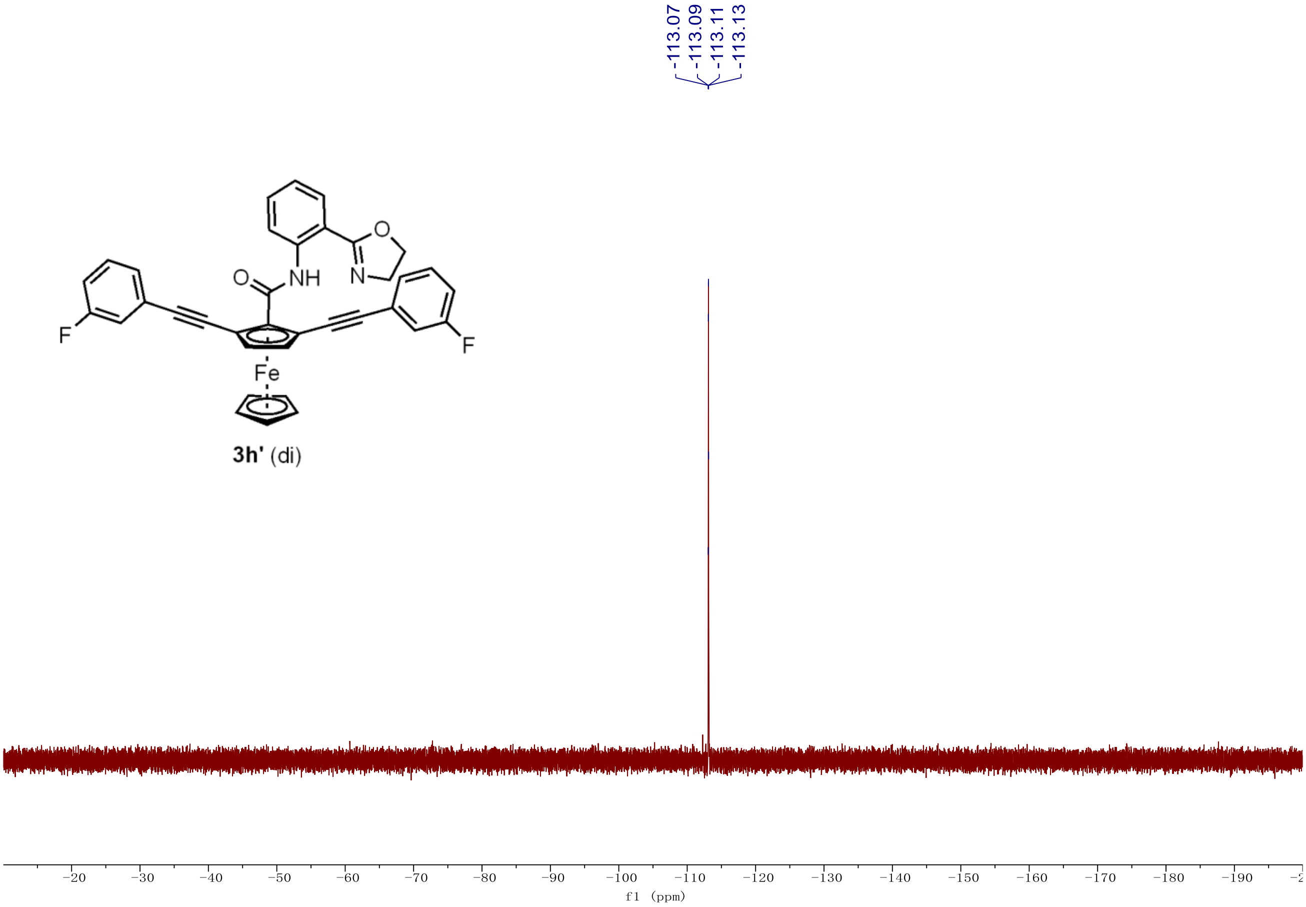
-113.07
-113.09
-113.11
-113.13
O
NH
O
N
F
F
Fe
3h' (di)
−20 −30 −40 −50 −60 −70 −80 −90 −100 −110 −120 −130 −140 −150 −160 −170 −180 −190
f1 (ppm)

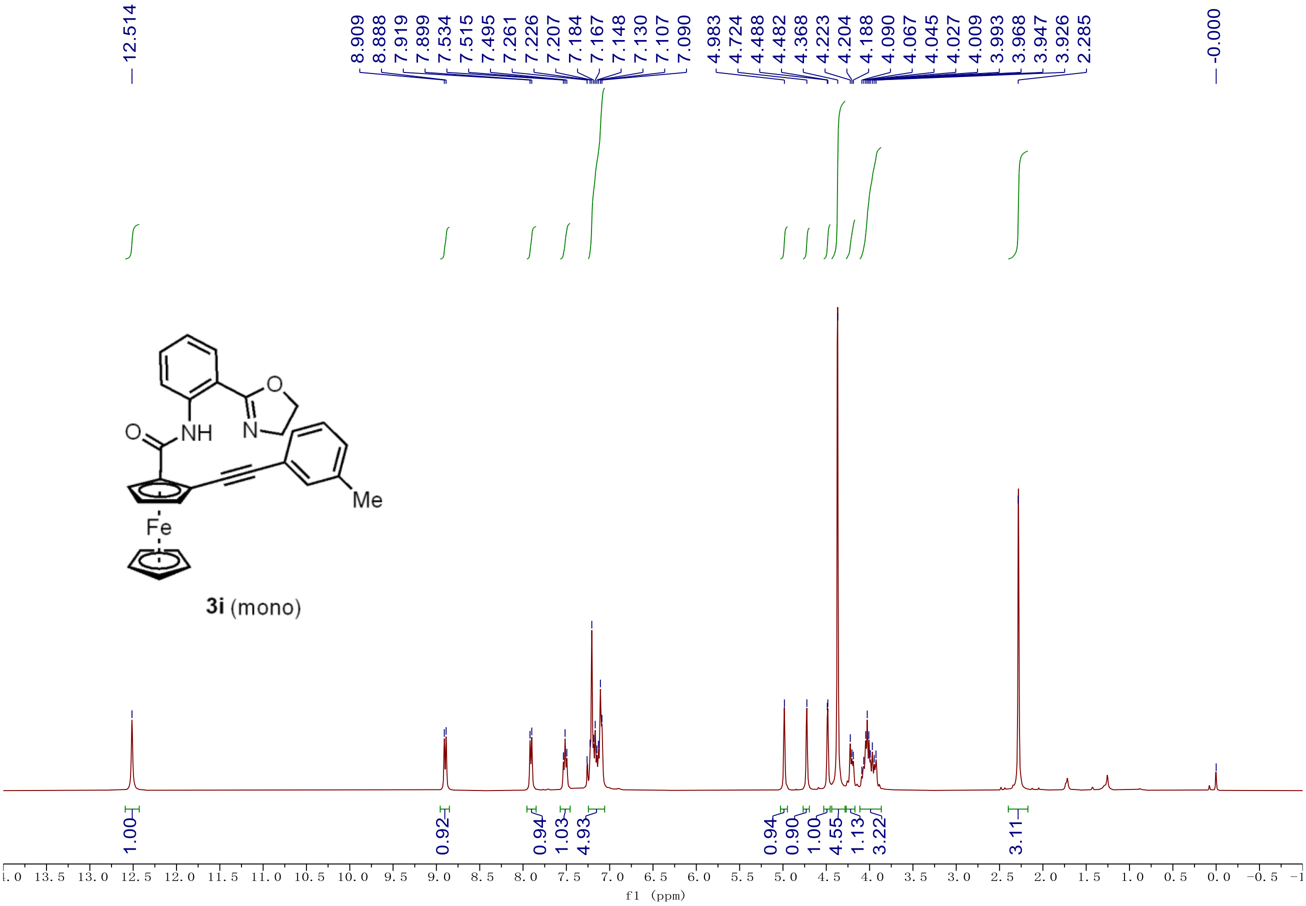
12.514
8.909 8.888 7.919 7.899 7.534 7.515 7.495 7.261 7.226 7.207 7.184 7.167 7.148 7.130 7.107 7.090
4.983 4.724 4.488 4.482 4.368 4.223 4.204 4.188 4.090 4.067 4.045 4.027 4.009 3.993 3.968 3.947 3.926 2.285
-0.000
Fe
O
NH
N
Me
3i (mono)
1.00 0.92 0.94 1.03 4.93 0.94 0.90 1.00 4.55 1.13 3.22 3.11
f1 (ppm)

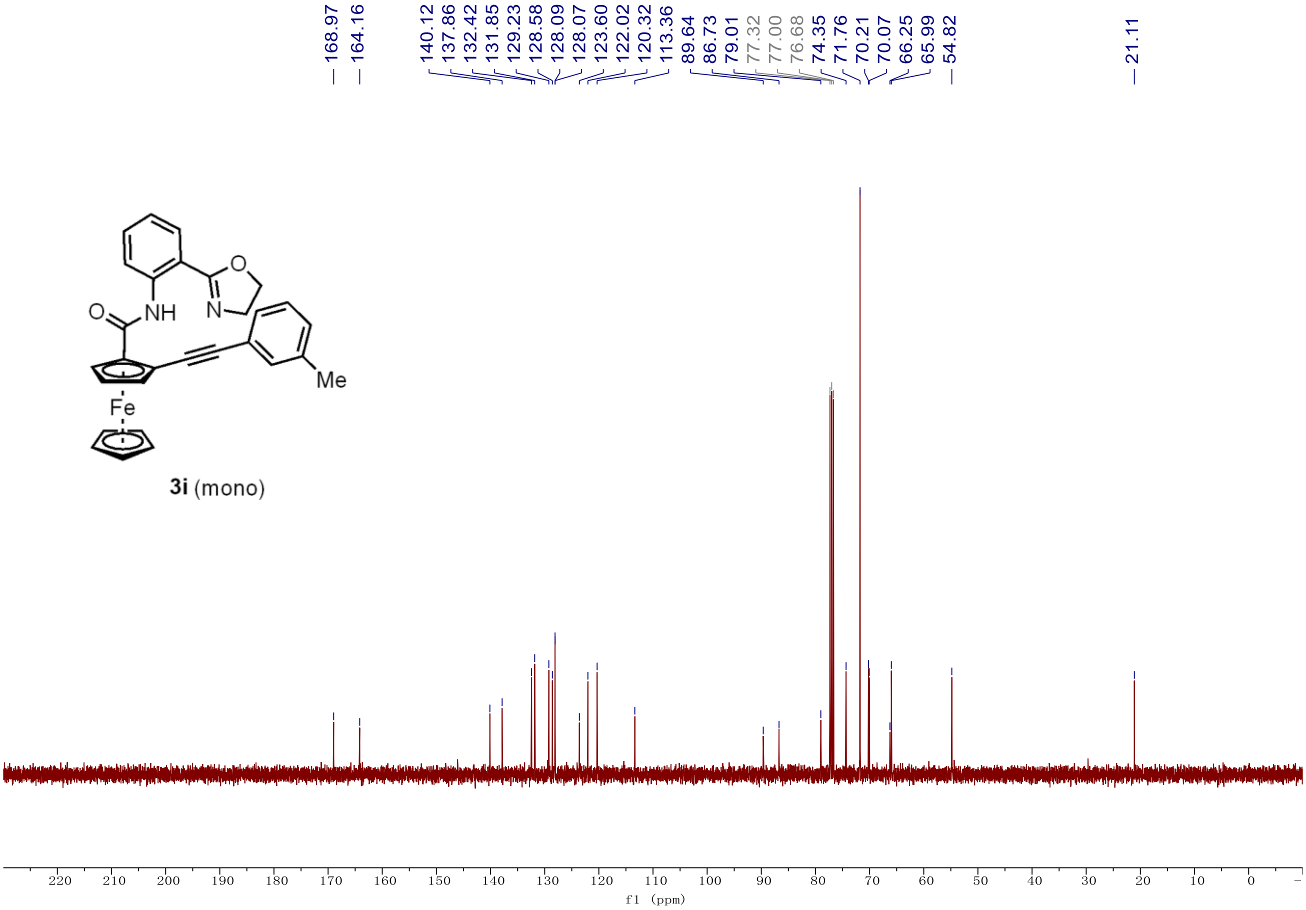
168.97 164.16
140.12 137.86 132.42 131.85 129.23 128.58 128.09 128.07 123.60 122.02 120.32 113.36
89.64 86.73 79.01 77.32 77.00 76.68 74.35 71.76 70.21 70.07 66.25 65.99 54.82
21.11
Fe
O
NH
N
Me
3i (mono)
f1 (ppm)

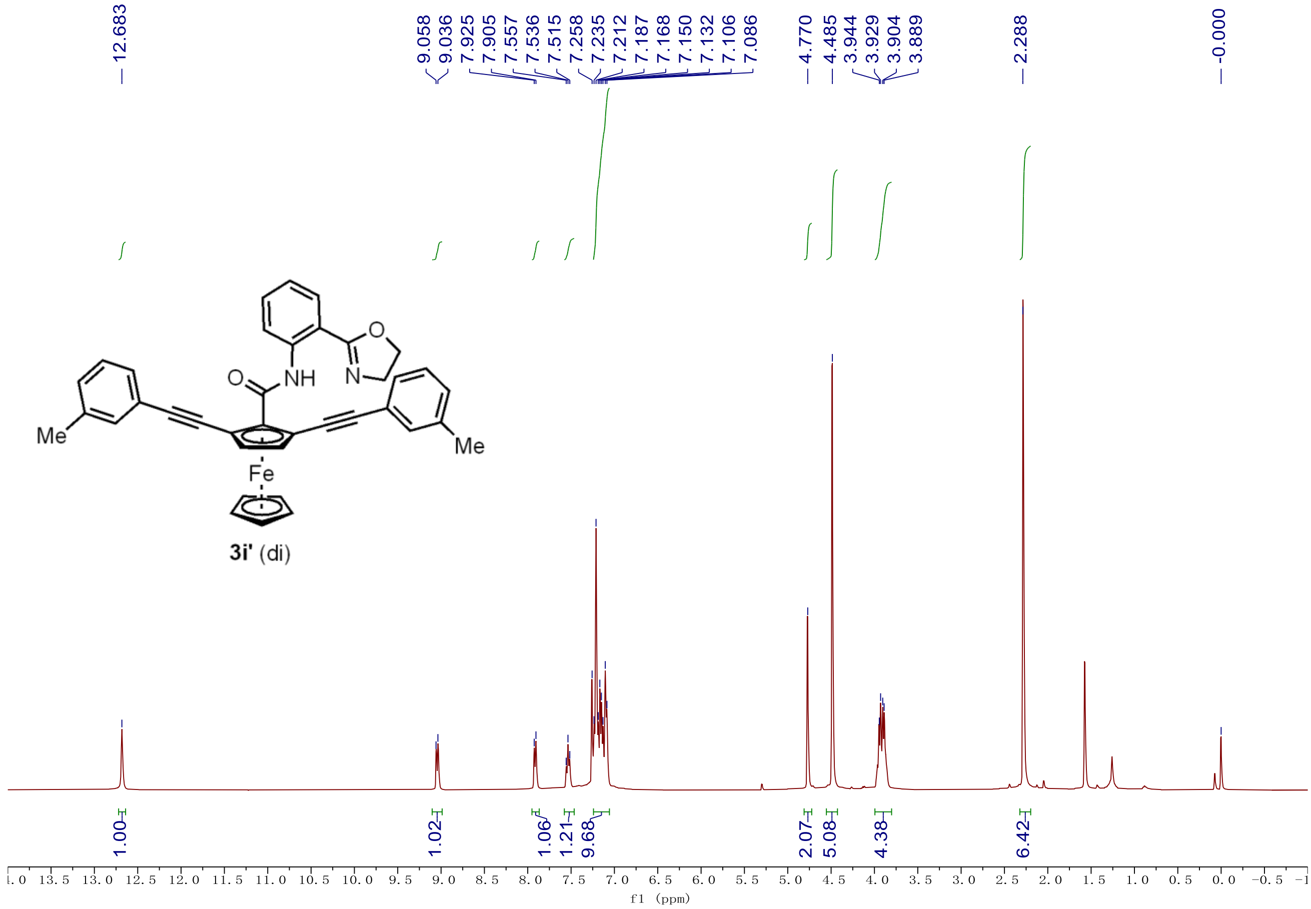
12.683
9.058
9.036
7.925
7.905
7.557
7.536
7.515
7.258
7.235
7.212
7.187
7.168
7.150
7.132
7.106
7.086
4.770
4.485
3.944
3.929
3.904
3.889
2.288
-0.000
O
NH
O
N
Me
Me
Fe
3i' (di)
1.00
1.02
1.06
1.21
9.68
2.07
5.08
4.38
6.42
f1 (ppm)

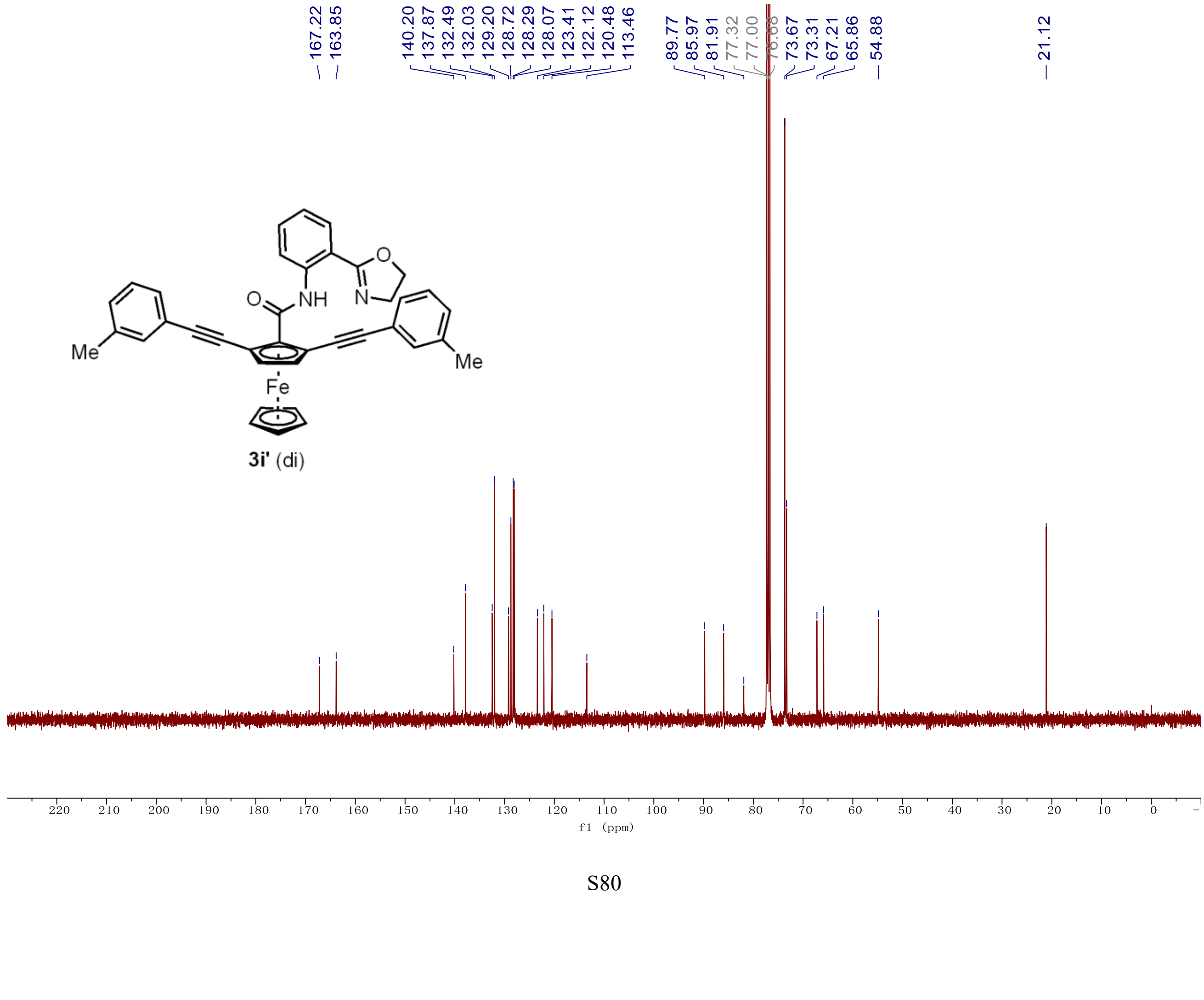
167.22
163.85
140.20
137.87
132.49
132.03
129.20
128.72
128.29
128.07
123.41
122.12
120.48
113.46
89.77
85.97
81.91
77.32
77.00
76.68
73.67
73.31
67.21
65.86
54.88
21.12
O
NH
O
N
Me
Me
Fe
3i' (di)
f1 (ppm)

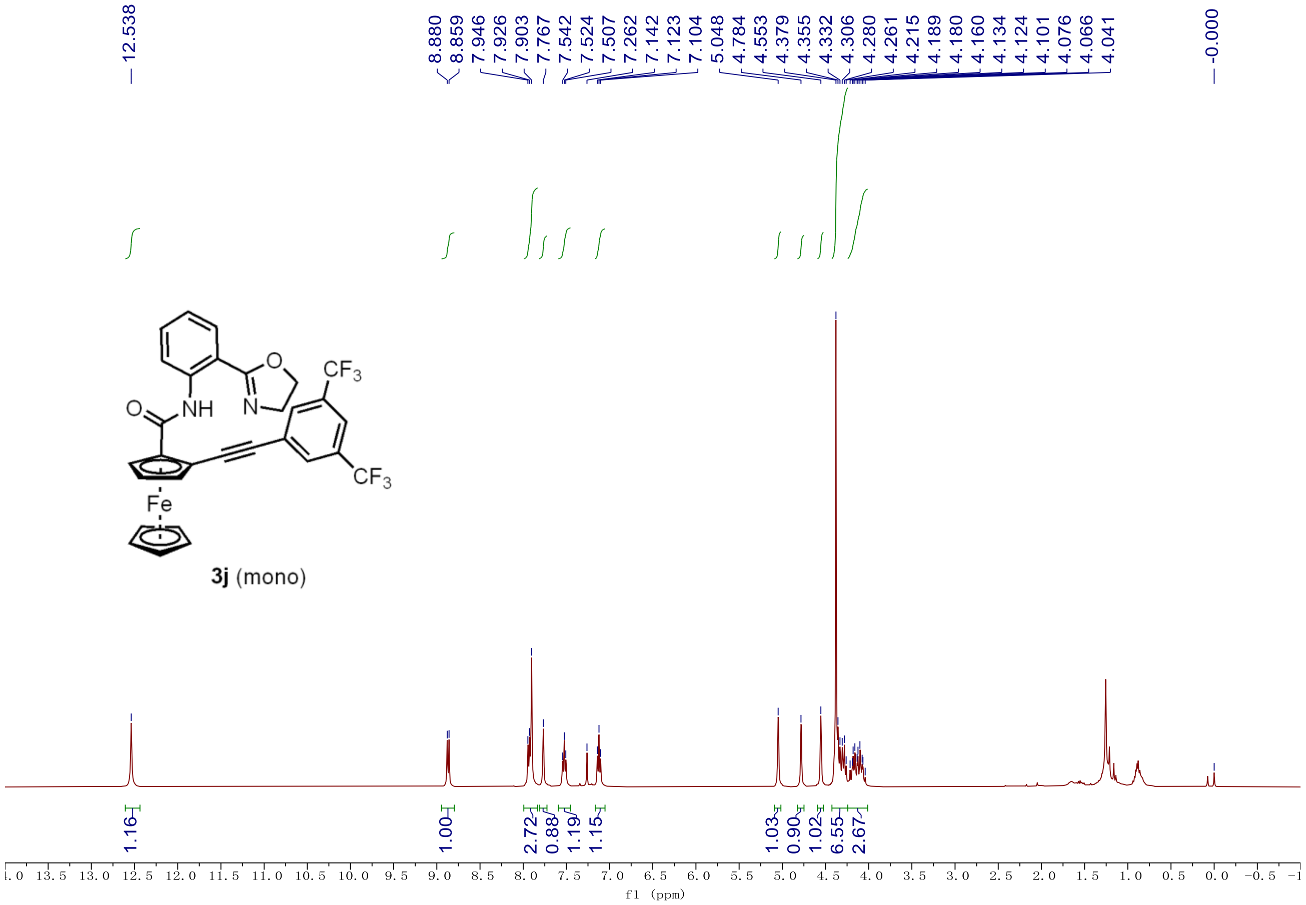
12.538
8.880
8.859
7.946
7.926
7.903
7.767
7.542
7.524
7.507
7.262
7.142
7.123
7.104
5.048
4.784
4.553
4.379
4.355
4.332
4.306
4.280
4.261
4.215
4.189
4.180
4.160
4.134
4.124
4.101
4.076
4.066
4.041
-0.000
CF3
O
NH
N
O
CF3
Fe
3j (mono)
1.16
1.00
2.72
0.88
1.19
1.15
1.03
0.90
1.02
6.55
2.67
f1 (ppm)

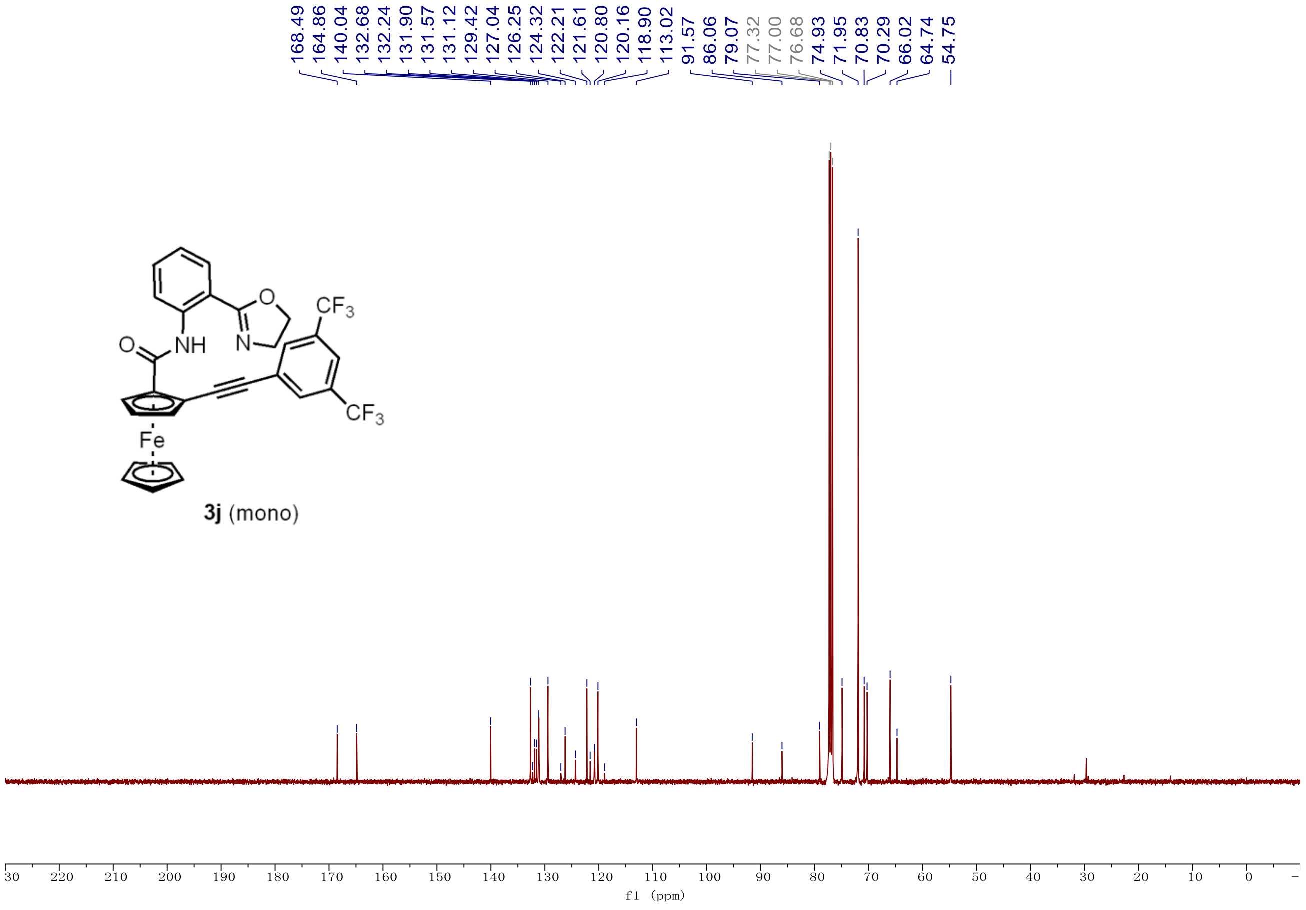
168.49
164.86
140.04
132.68
132.24
131.90
131.57
131.12
129.42
127.04
126.25
124.32
122.21
121.61
120.80
120.16
118.90
113.02
91.57
86.06
79.07
77.32
77.00
76.68
74.93
71.95
70.83
70.29
66.02
64.74
54.75
CF3
O
NH
N
O
CF3
Fe
3j (mono)
f1 (ppm)

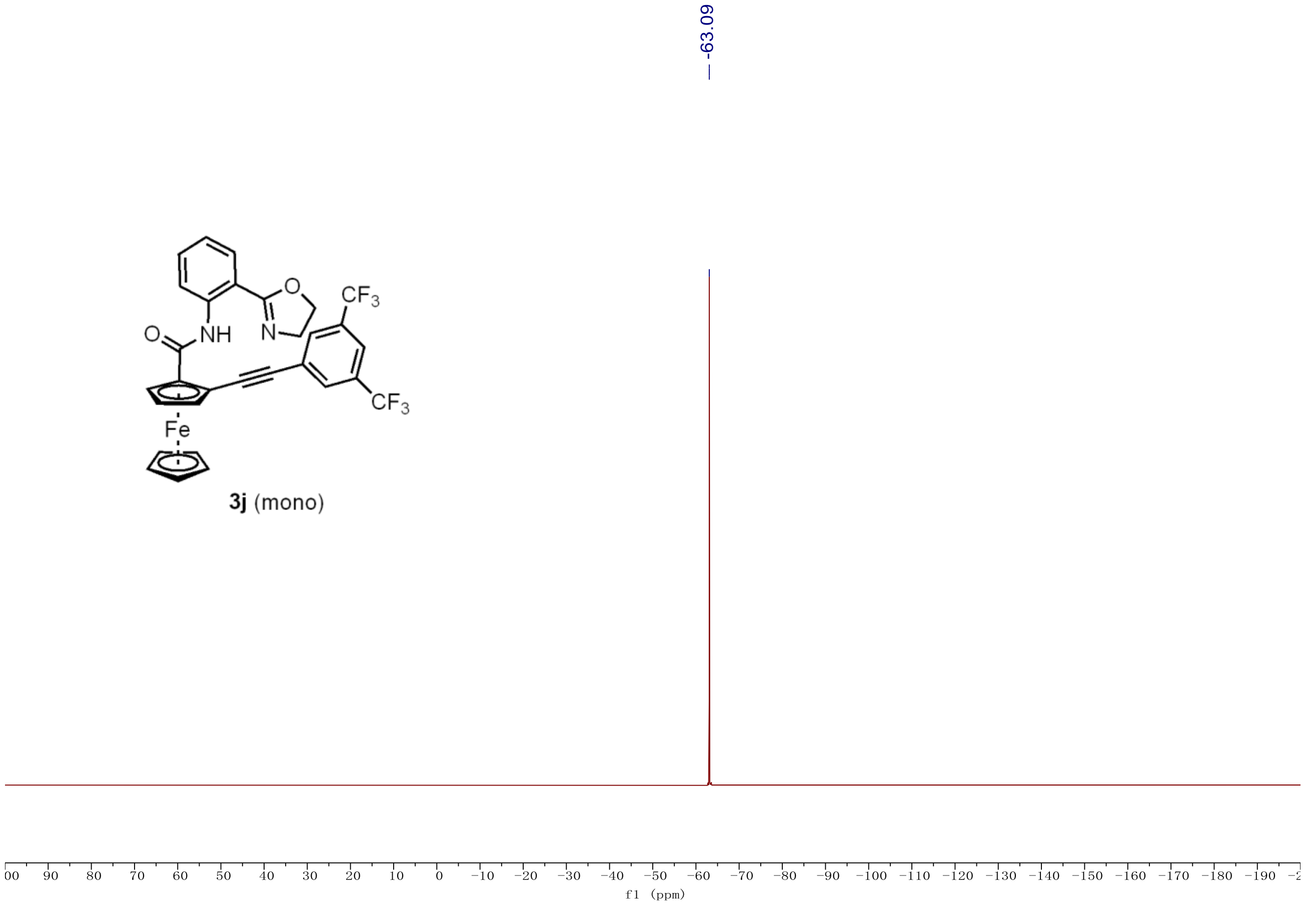

-63.09
CF3
CF3
O
N
O
NH
Fe
3j (mono)
f1 (ppm)

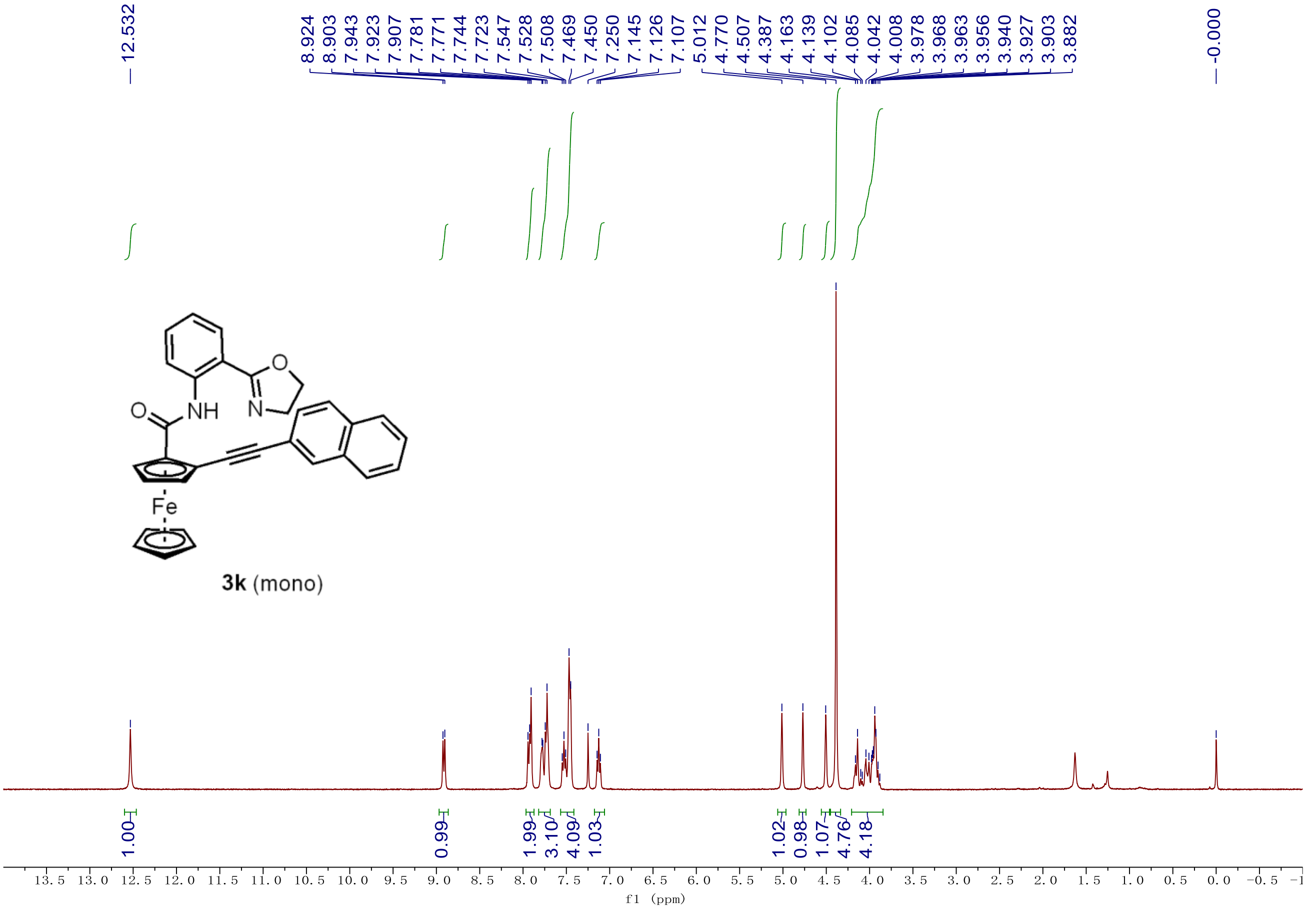

12.532
8.924 8.903 7.943 7.923 7.907 7.781 7.771 7.744 7.723 7.547 7.528 7.508 7.469 7.450 7.250 7.145 7.126 7.107 5.012 4.770 4.507 4.387 4.163 4.139 4.102 4.085 4.042 4.008 3.978 3.968 3.963 3.956 3.940 3.927 3.903 3.882
-0.000
O
N
O
NH
Fe
3k (mono)
1.00 0.99 1.99 3.10 4.09 1.03 1.02 0.98 1.07 4.76 4.18
f1 (ppm)

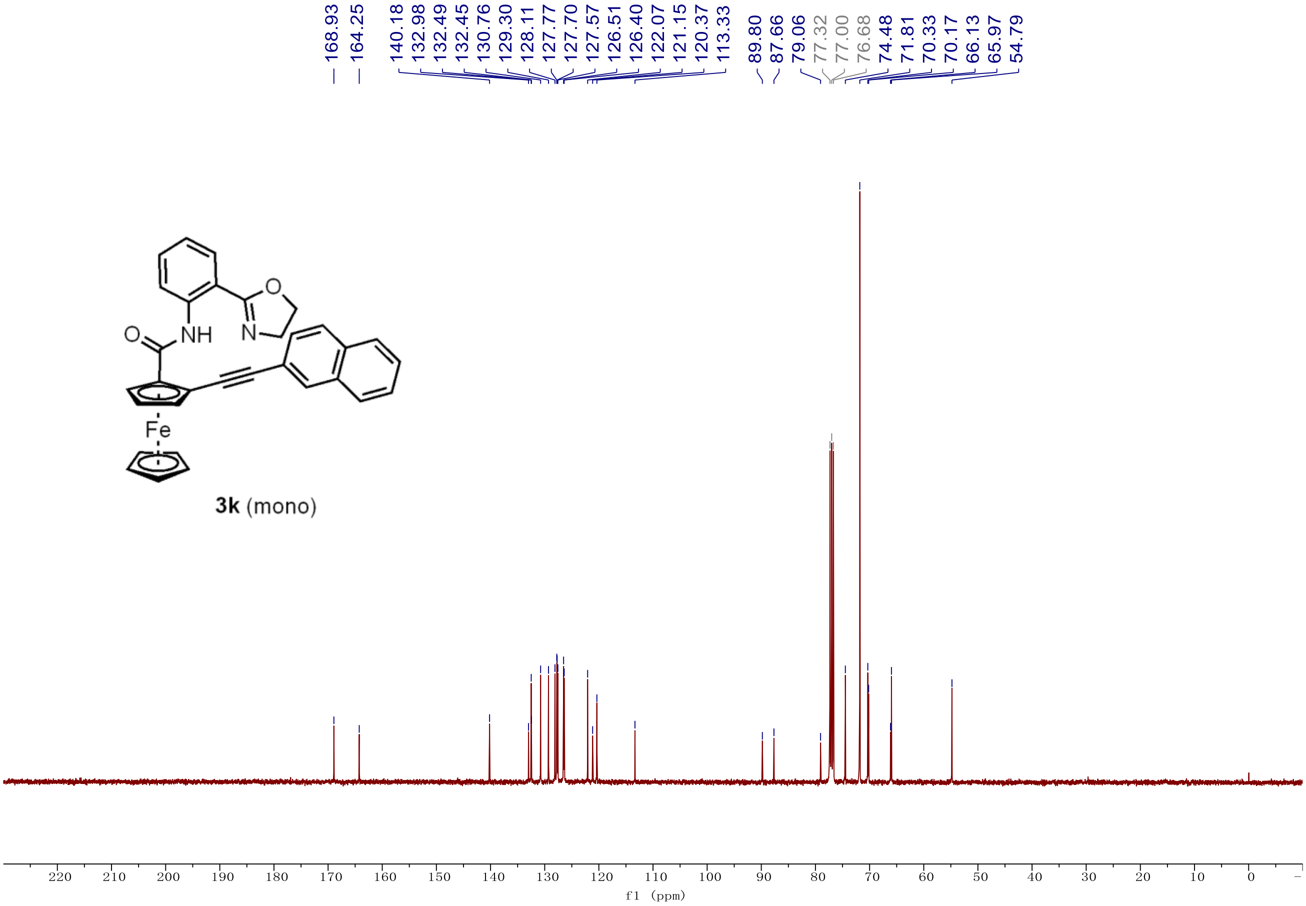
168.93
164.25
140.18
132.98
132.49
132.45
130.76
129.30
128.11
127.77
127.70
127.57
126.51
126.40
122.07
121.15
120.37
113.33
89.80
87.66
79.06
77.32
77.00
76.68
74.48
71.81
70.33
70.17
66.13
65.97
54.79
Fe
O
NH
N
O
220 210 200 190 180 170 160 150 140 130 120 110 100 90 80 70 60 50 40 30 20 10 0
f1 (ppm)

3k (mono)

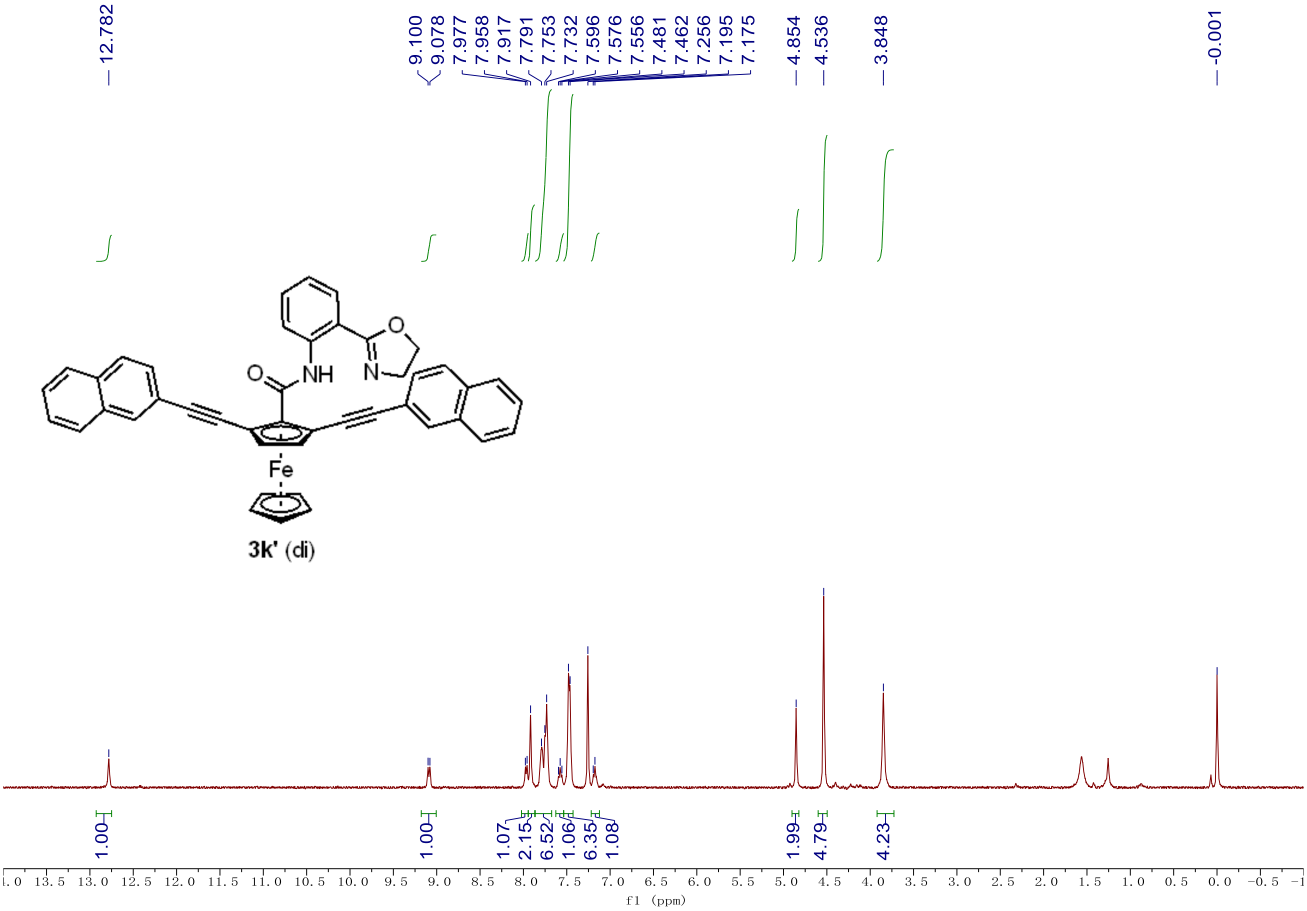
12.782
9.100
9.078
7.977
7.958
7.917
7.791
7.753
7.732
7.596
7.576
7.556
7.481
7.462
7.256
7.195
7.175
4.854
4.536
3.848
-0.001
O
NH
N
O
Fe
1.00
1.00
1.07
2.15
6.52
1.06
6.35
1.08
1.99
4.79
4.23
13.5 13.0 12.5 12.0 11.5 11.0 10.5 10.0 9.5 9.0 8.5 8.0 7.5 7.0 6.5 6.0 5.5 5.0 4.5 4.0 3.5 3.0 2.5 2.0 1.5 1.0 0.5 0.0 -0.5
f1 (ppm)

3k' (di)

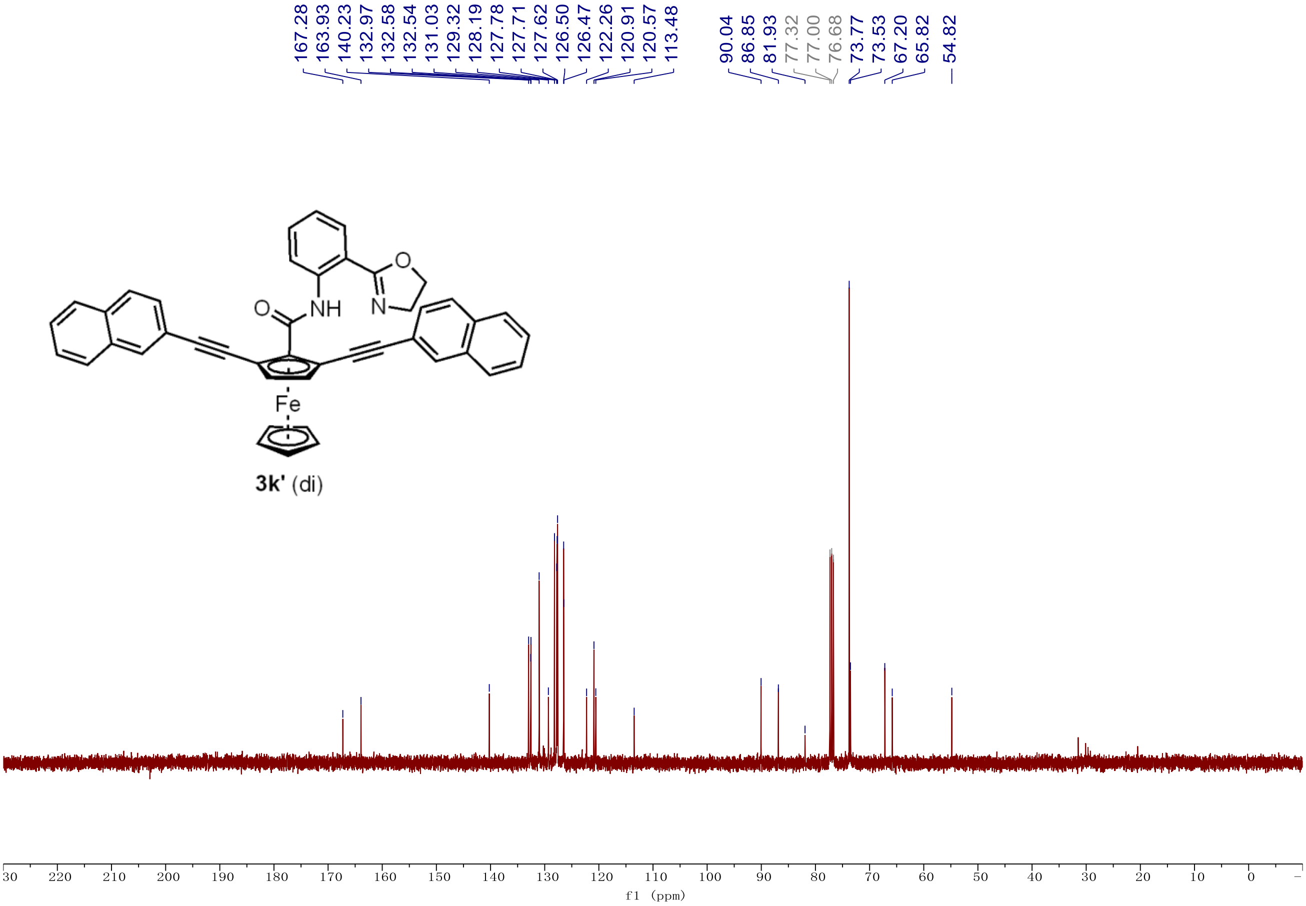
167.28
163.93
140.23
132.97
132.58
132.54
131.03
129.32
128.19
127.78
127.71
127.62
126.50
126.47
122.26
120.91
120.57
113.48
90.04
86.85
81.93
77.32
77.00
76.68
73.77
73.53
67.20
65.82
54.82
O
NH
N
O
Fe
3k' (di)
f1 (ppm)

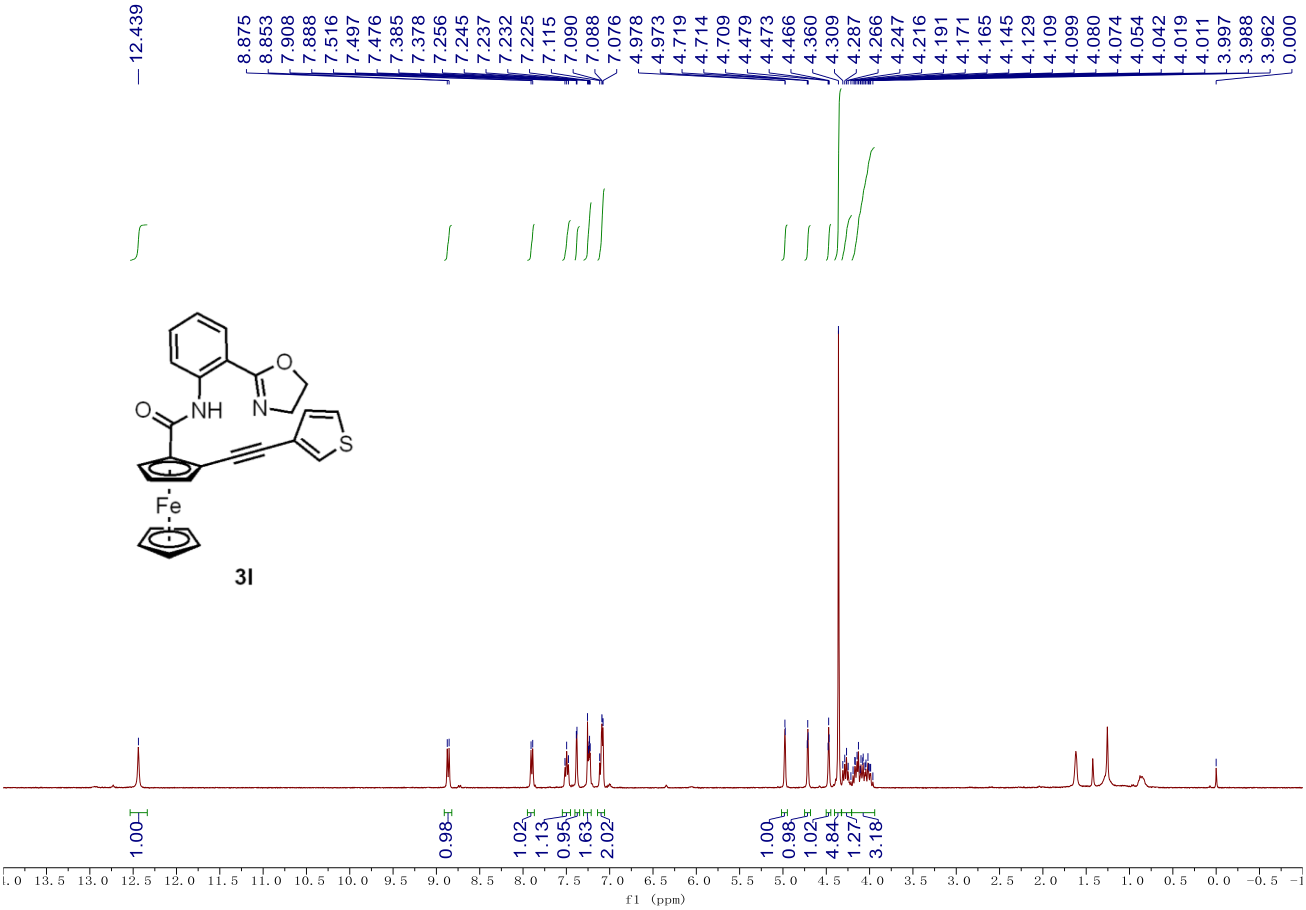
12.439
8.875
8.853
7.908
7.888
7.516
7.497
7.476
7.385
7.378
7.256
7.245
7.237
7.232
7.225
7.115
7.090
7.088
7.076
4.978
4.973
4.719
4.714
4.709
4.479
4.473
4.466
4.360
4.309
4.287
4.266
4.247
4.216
4.191
4.171
4.165
4.145
4.129
4.109
4.099
4.080
4.074
4.054
4.042
4.019
4.011
3.997
3.988
3.962
0.000
O
NH
N
O
S
Fe
3l
1.00
0.98
1.02
1.13
0.95
1.63
2.02
1.00
0.98
1.02
4.84
1.27
3.18
f1 (ppm)

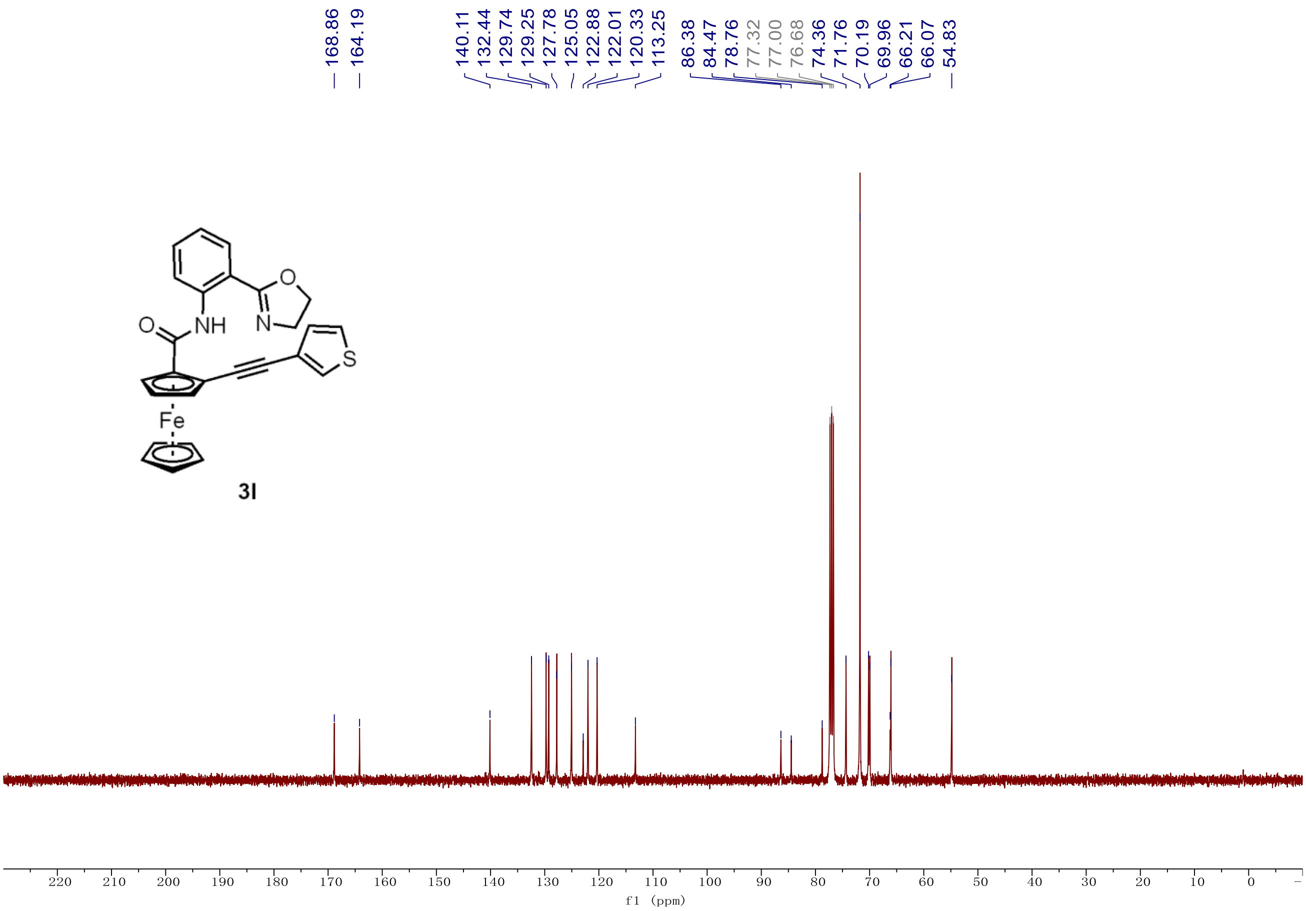
168.86
164.19
140.11
132.44
129.74
129.25
127.78
125.05
122.88
122.01
120.33
113.25
86.38
84.47
78.76
77.32
77.00
76.68
74.36
71.76
70.19
69.96
66.21
66.07
54.83
O
NH
O
N
S
Fe
3l
f1 (ppm)

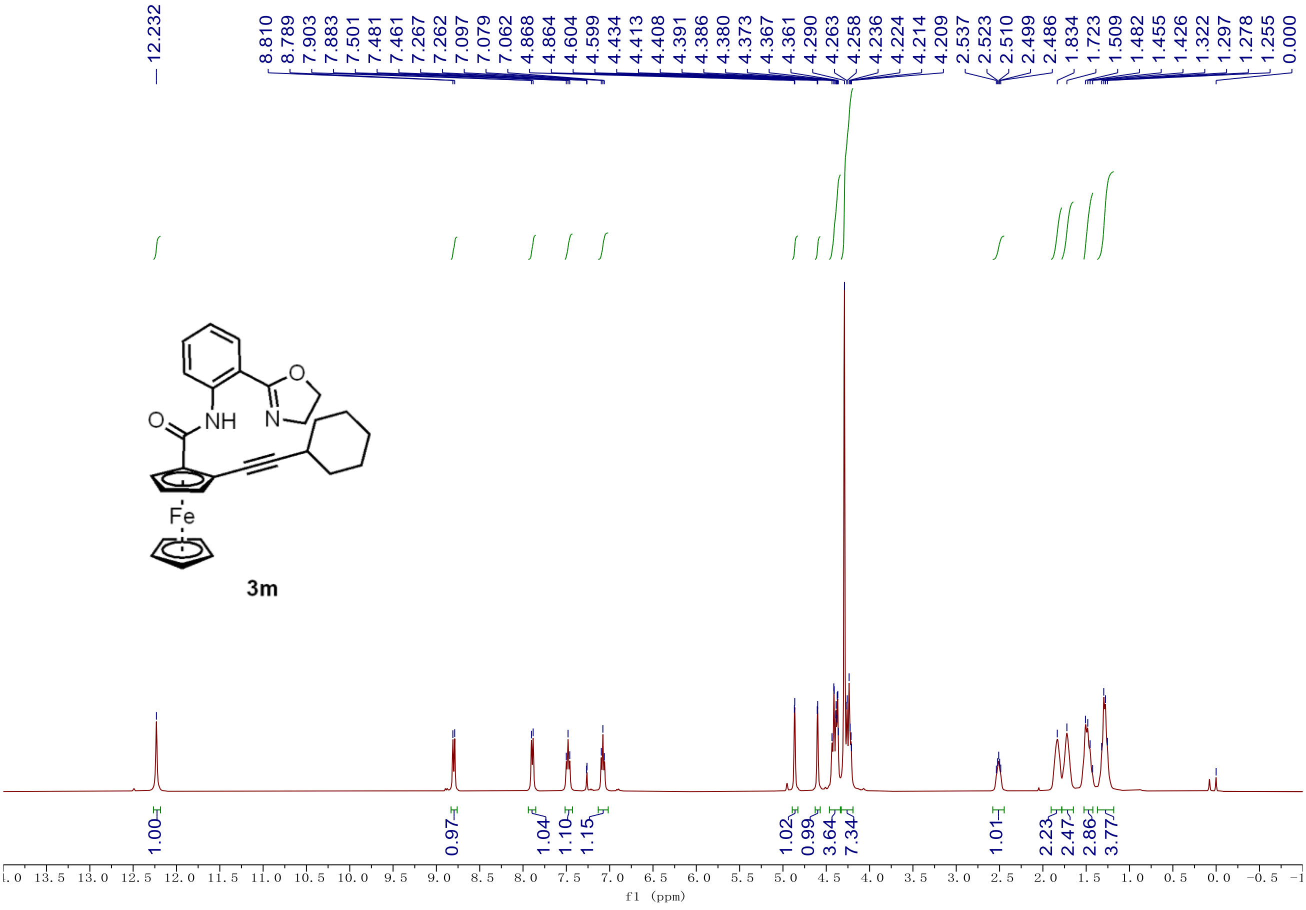
12.232
8.810
8.789
7.903
7.883
7.501
7.481
7.461
7.267
7.262
7.097
7.079
7.062
4.868
4.864
4.604
4.599
4.434
4.413
4.408
4.391
4.386
4.380
4.373
4.367
4.361
4.290
4.263
4.258
4.236
4.224
4.214
4.209
2.537
2.523
2.510
2.499
2.486
1.834
1.723
1.509
1.482
1.455
1.426
1.322
1.297
1.278
1.255
0.000
O
NH
O
N
Fe
3m
1.00
0.97
1.04
1.10
1.15
1.02
0.99
3.64
7.34
1.01
2.23
2.47
2.86
3.77
f1 (ppm)

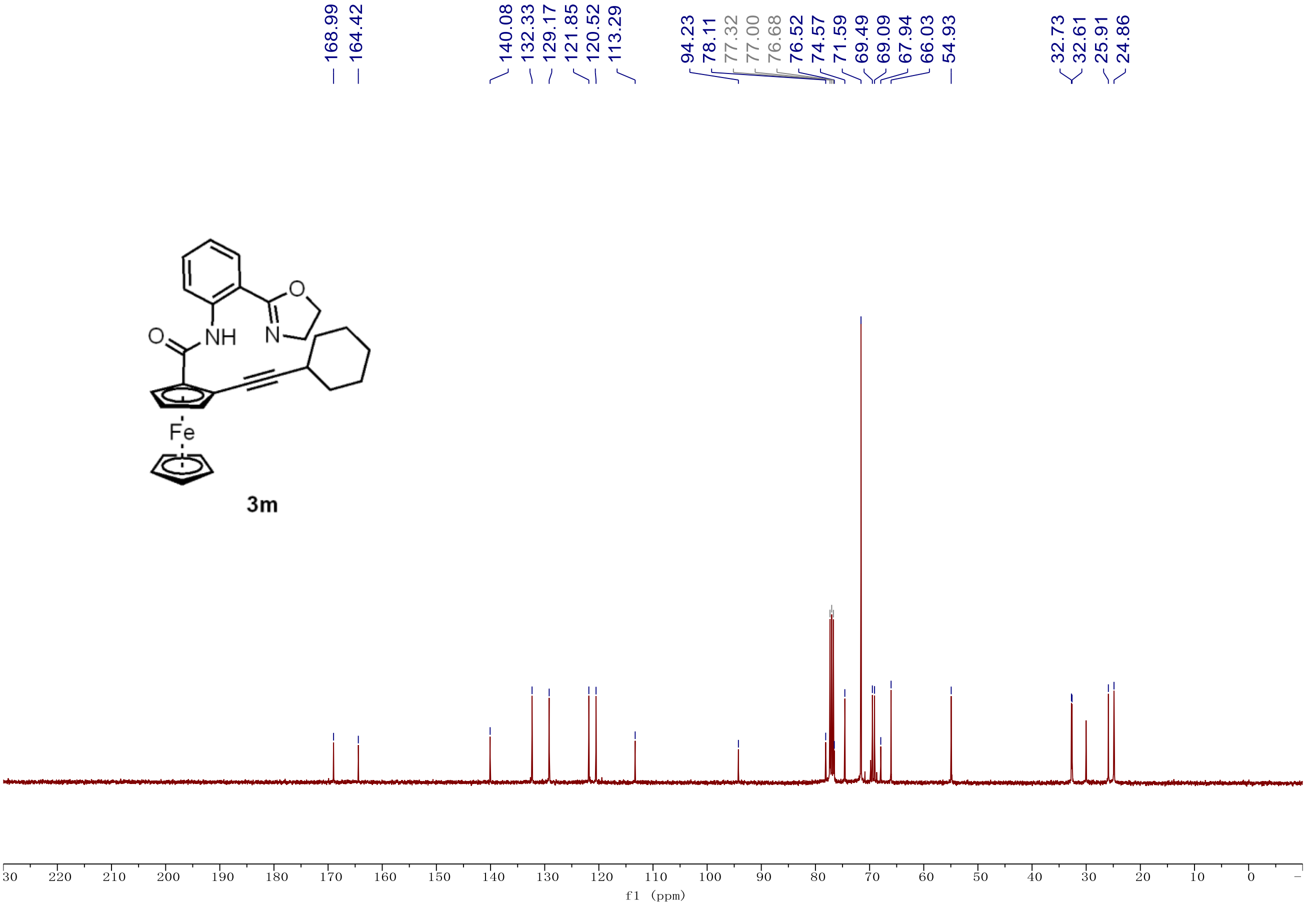
168.99
164.42
140.08
132.33
129.17
121.85
120.52
113.29
94.23
78.11
77.32
77.00
76.68
76.52
74.57
71.59
69.49
69.09
67.94
66.03
54.93
32.73
32.61
25.91
24.86
O
NH
N
O
Fe
3m
f1 (ppm)

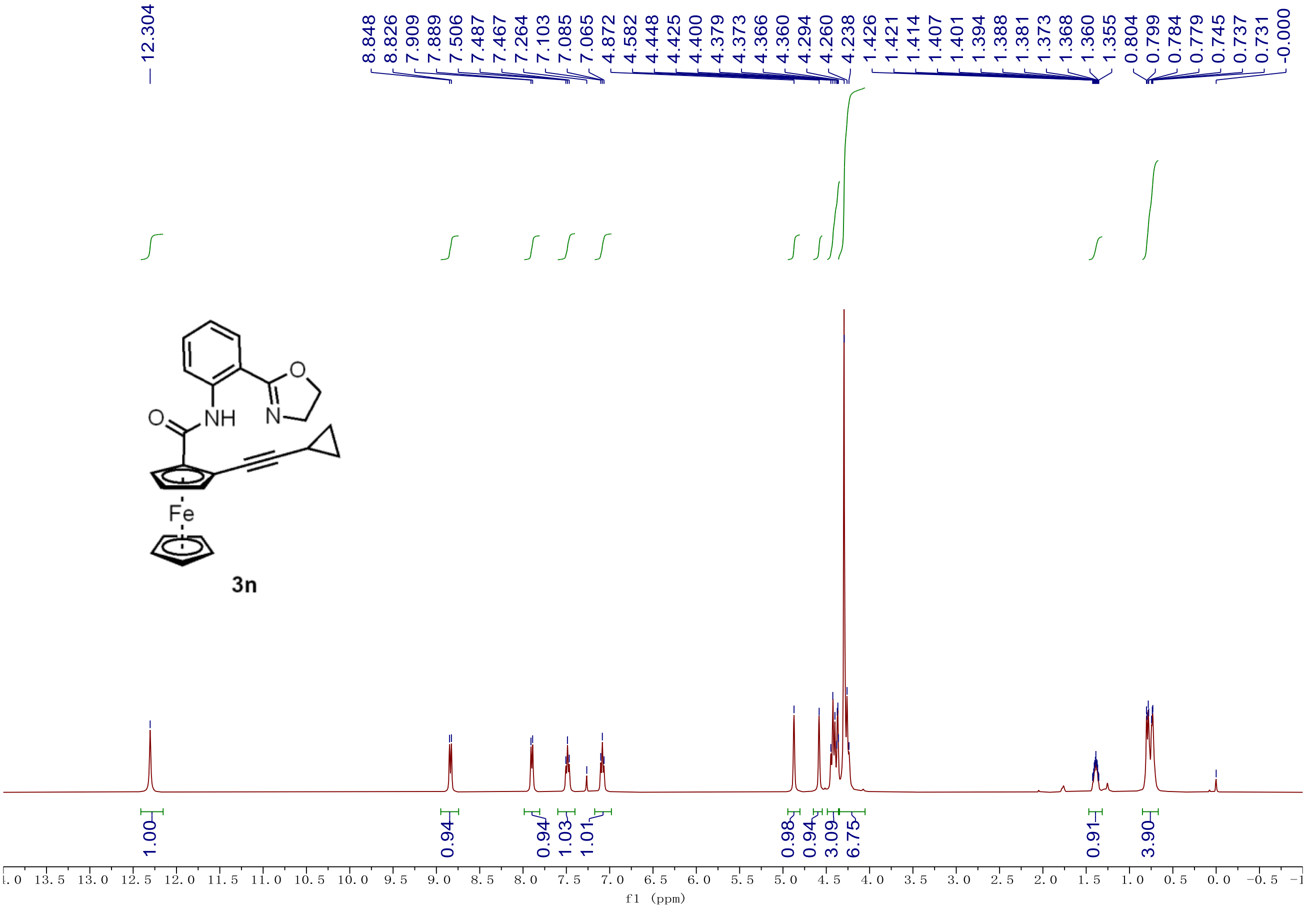
12.304
8.848
8.826
7.909
7.889
7.506
7.487
7.467
7.264
7.103
7.085
7.065
4.872
4.582
4.448
4.425
4.400
4.379
4.373
4.366
4.360
4.294
4.260
4.238
1.426
1.421
1.414
1.407
1.401
1.394
1.388
1.381
1.373
1.368
1.360
1.355
0.804
0.799
0.784
0.779
0.745
0.737
0.731
-0.000
O
NH
N
O
Fe
3n
1.00
0.94
0.94
1.03
1.01
0.98
0.94
3.09
6.75
0.91
3.90
f1 (ppm)

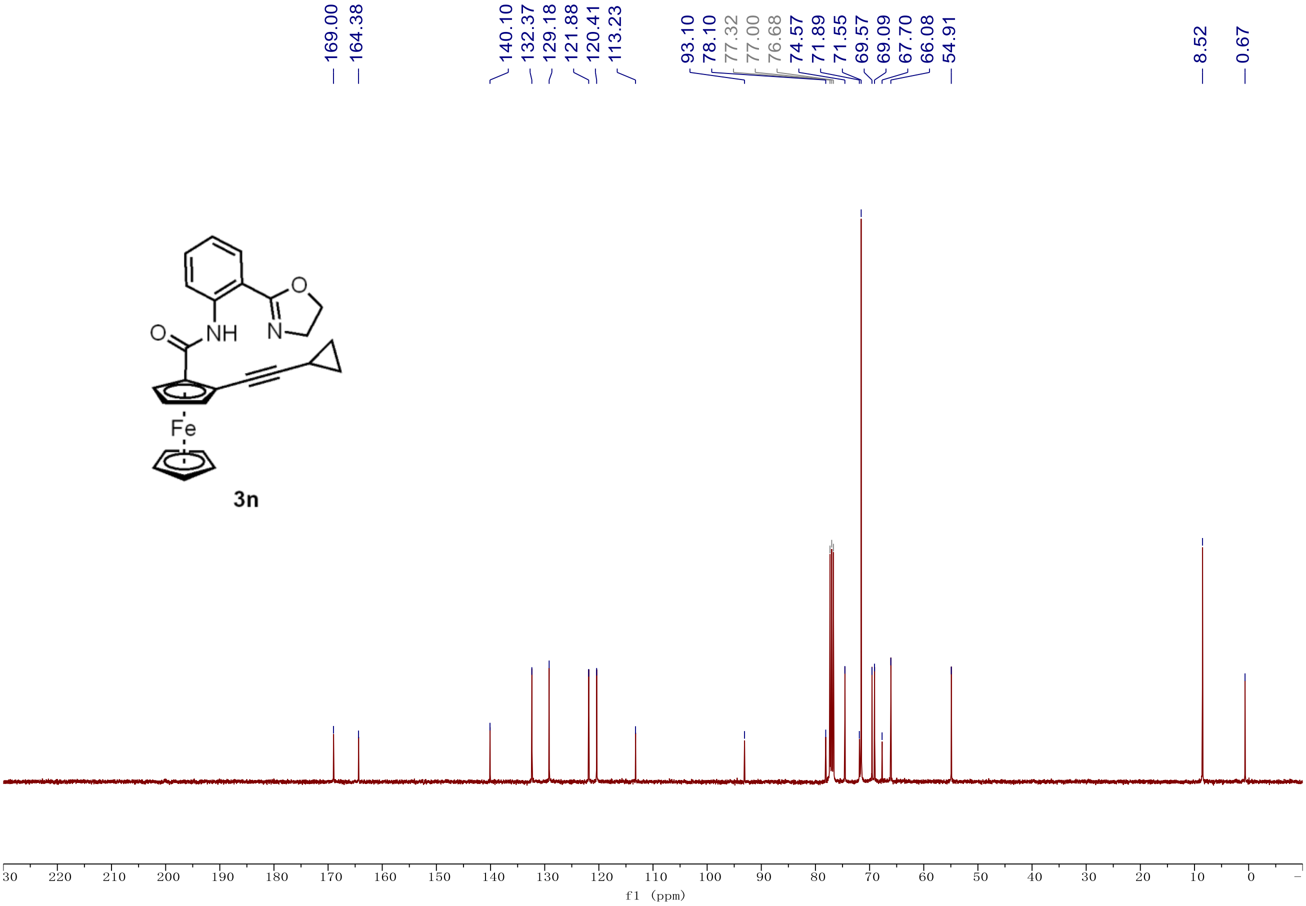
169.00
164.38
140.10
132.37
129.18
121.88
120.41
113.23
93.10
78.10
77.32
77.00
76.68
74.57
71.89
71.55
69.57
69.09
67.70
66.08
54.91
8.52
0.67
O
NH
N
O
Fe
3n
f1 (ppm)

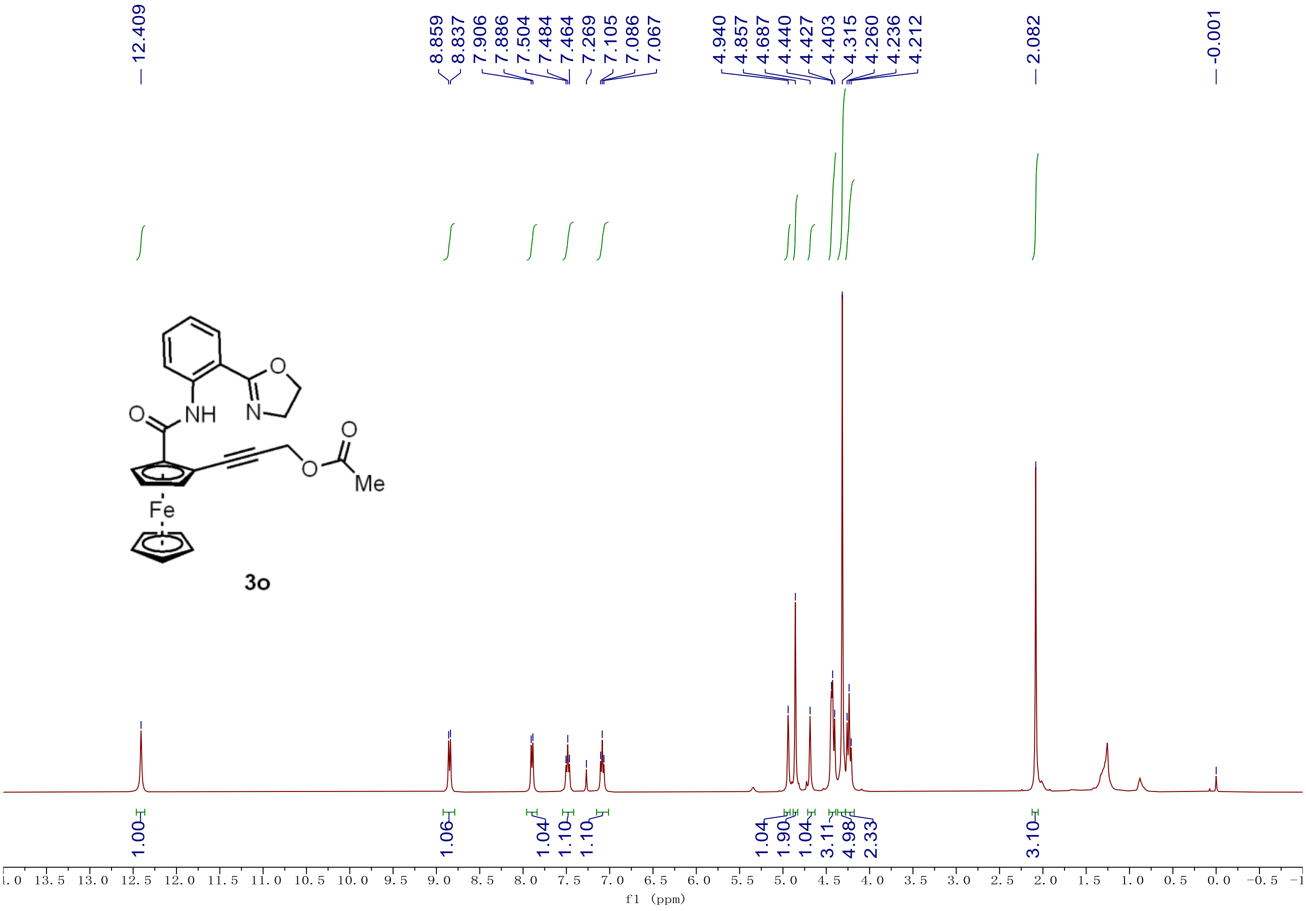
12.409
8.859
8.837
7.906
7.886
7.504
7.484
7.464
7.269
7.105
7.086
7.067
4.940
4.857
4.687
4.440
4.427
4.403
4.315
4.260
4.236
4.212
2.082
-0.001
O
NH
N
O
O
O
Me
Fe
3o
1.00
1.06
1.04
1.10
1.10
1.04
1.90
1.04
3.11
4.98
2.33
3.10
f1 (ppm)

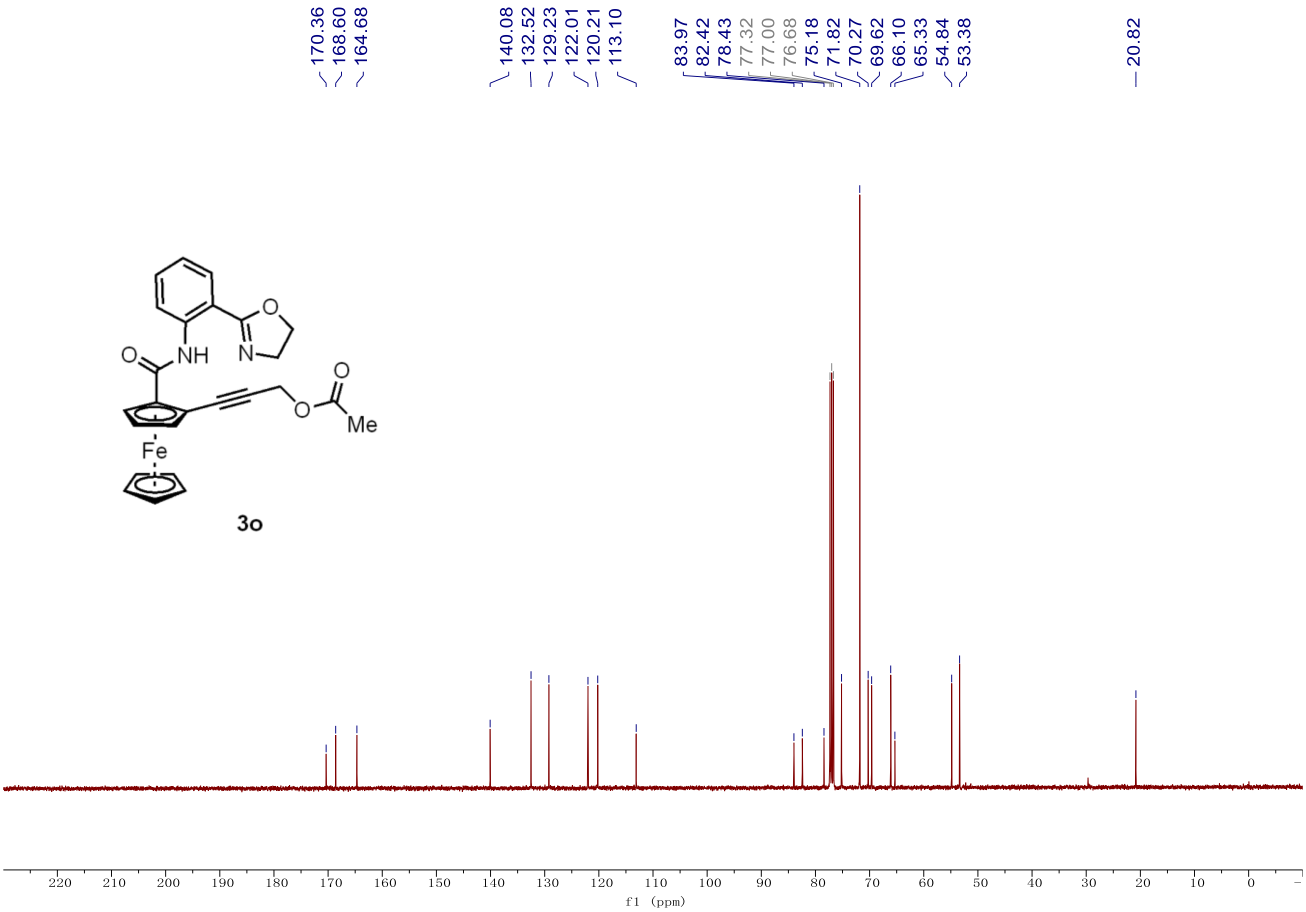
170.36
168.60
164.68
140.08
132.52
129.23
122.01
120.21
113.10
83.97
82.42
78.43
77.32
77.00
76.68
75.18
71.82
70.27
69.62
66.10
65.33
54.84
53.38
20.82
NH
O
N
O
O
Me
Fe
220 210 200 190 180 170 160 150 140 130 120 110 100 90 80 70 60 50 40 30 20 10 0
f1 (ppm)

3o

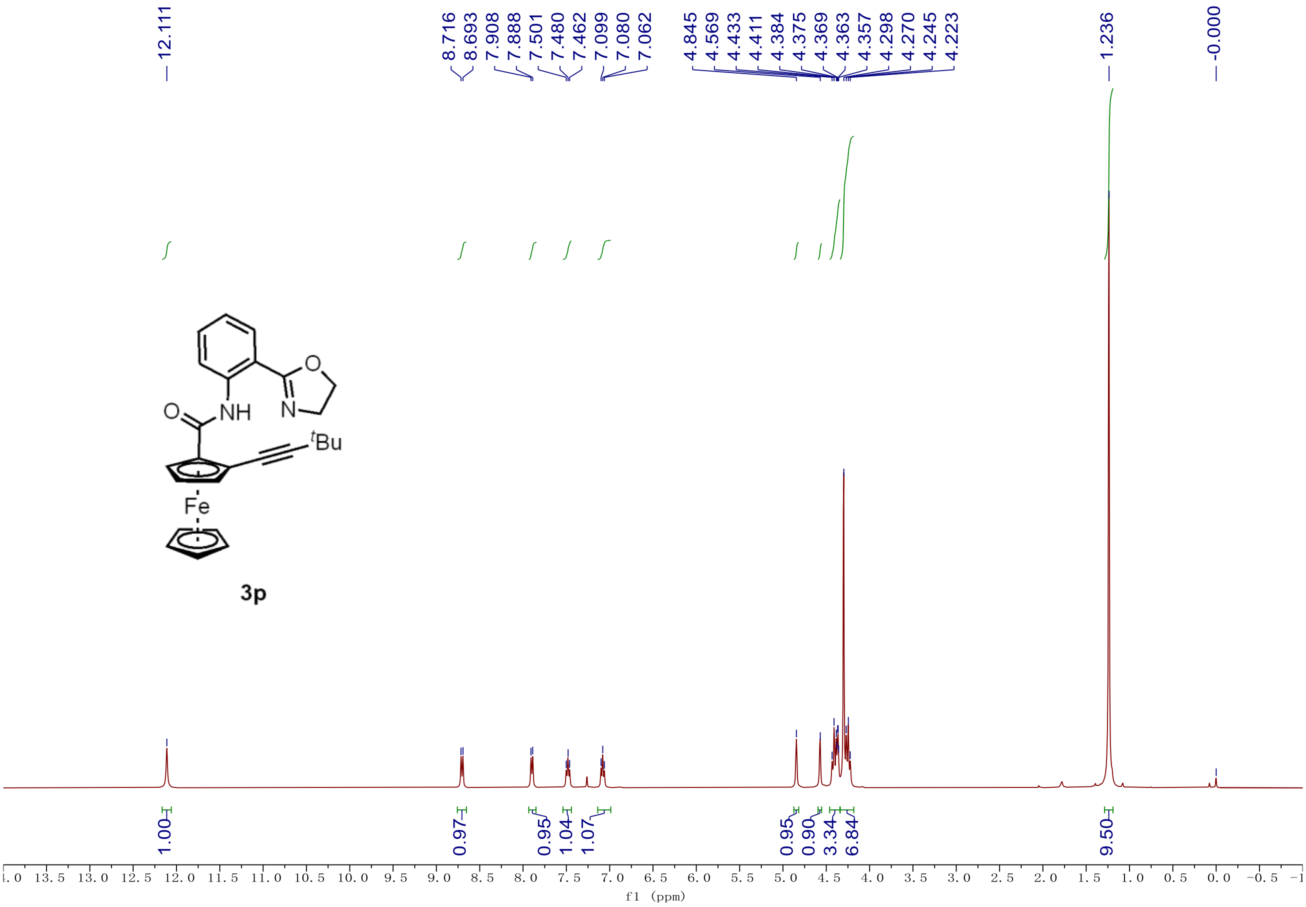
12.111
8.716
8.693
7.908
7.888
7.501
7.480
7.462
7.099
7.080
7.062
4.845
4.569
4.433
4.411
4.384
4.375
4.369
4.363
4.357
4.298
4.270
4.245
4.223
1.236
-0.000
O
NH
N
O
tBu
Fe
1.00
0.97
0.95
1.04
1.07
0.95
0.90
3.34
6.84
9.50
13.5 13.0 12.5 12.0 11.5 11.0 10.5 10.0 9.5 9.0 8.5 8.0 7.5 7.0 6.5 6.0 5.5 5.0 4.5 4.0 3.5 3.0 2.5 2.0 1.5 1.0 0.5 0.0 -0.5
f1 (ppm)

3p

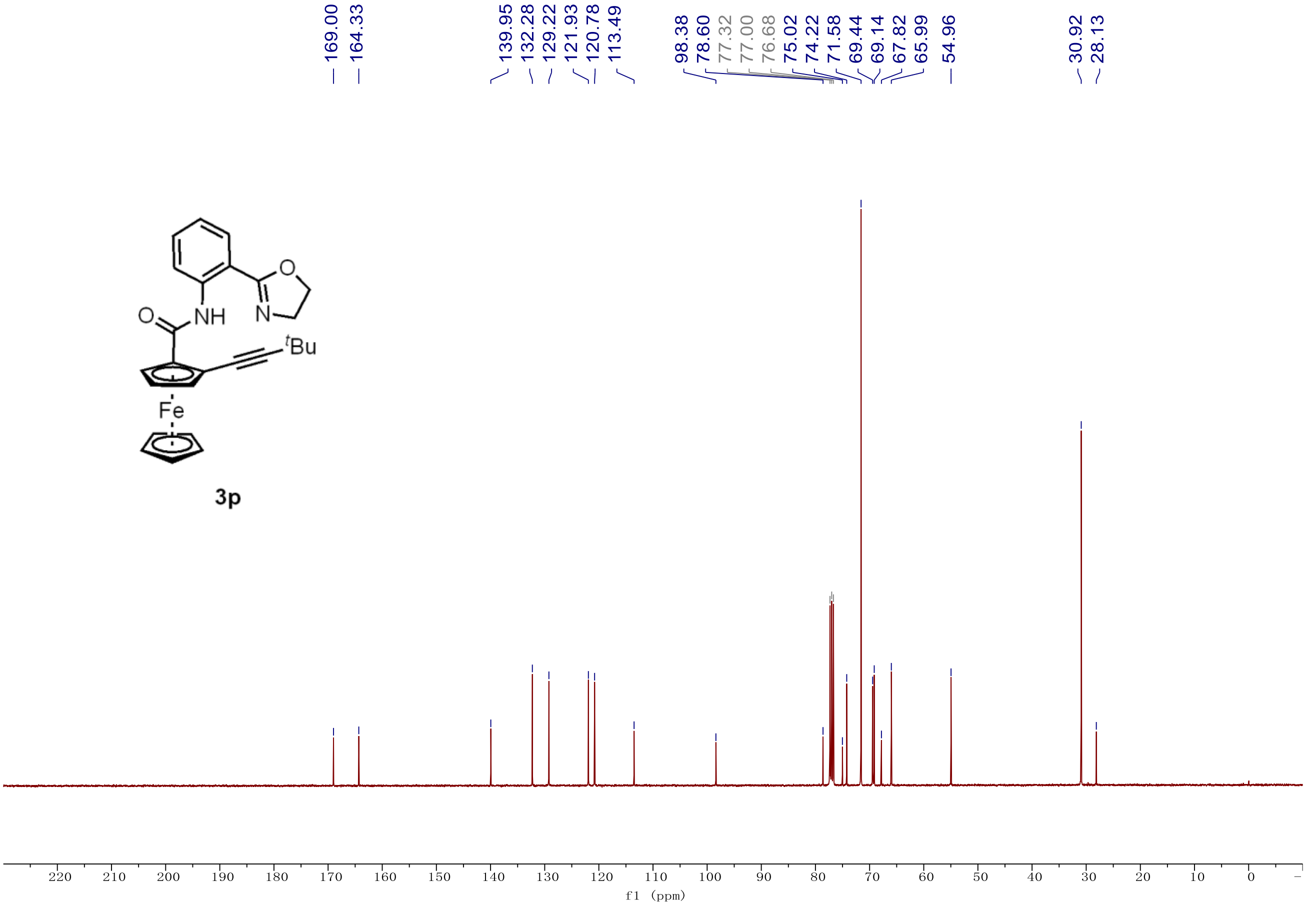
169.00
164.33
139.95
132.28
129.22
121.93
120.78
113.49
98.38
78.60
77.32
77.00
76.68
75.02
74.22
71.58
69.44
69.14
67.82
65.99
54.96
30.92
28.13
O
NH
N
O
tBu
Fe
3p
220 210 200 190 180 170 160 150 140 130 120 110 100 90 80 70 60 50 40 30 20 10 0
f1 (ppm)

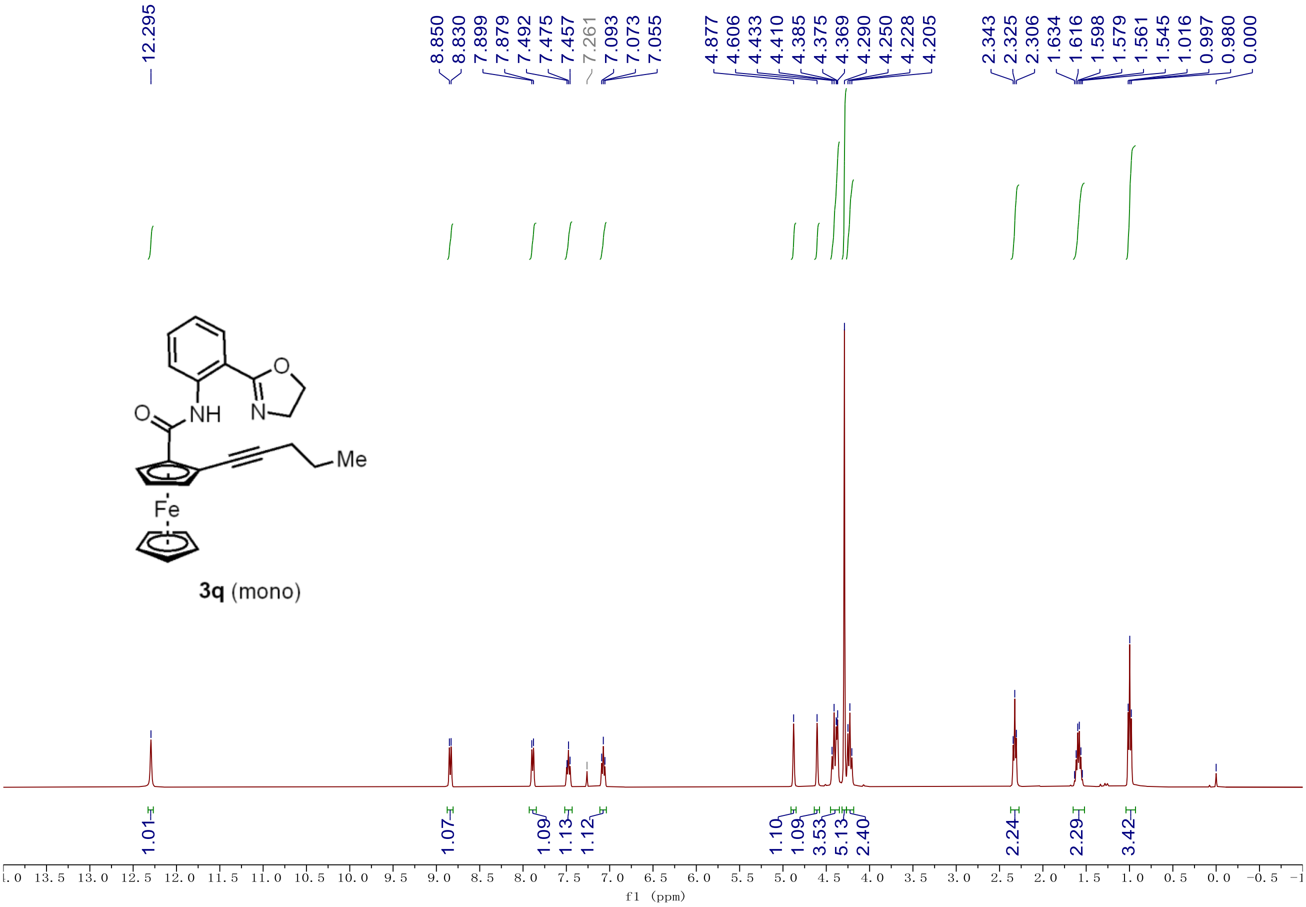
12.295
8.850
8.830
7.899
7.879
7.492
7.475
7.457
7.261
7.093
7.073
7.055
4.877
4.606
4.433
4.410
4.385
4.375
4.369
4.290
4.250
4.228
4.205
2.343
2.325
2.306
1.634
1.616
1.598
1.579
1.561
1.545
1.016
0.997
0.980
0.000
O
NH
N
O
Me
Fe
3q (mono)
1.01
1.07
1.09
1.13
1.12
1.10
1.09
3.53
5.13
2.40
2.24
2.29
3.42
13.5 13.0 12.5 12.0 11.5 11.0 10.5 10.0 9.5 9.0 8.5 8.0 7.5 7.0 6.5 6.0 5.5 5.0 4.5 4.0 3.5 3.0 2.5 2.0 1.5 1.0 0.5 0.0 -0.5
f1 (ppm)

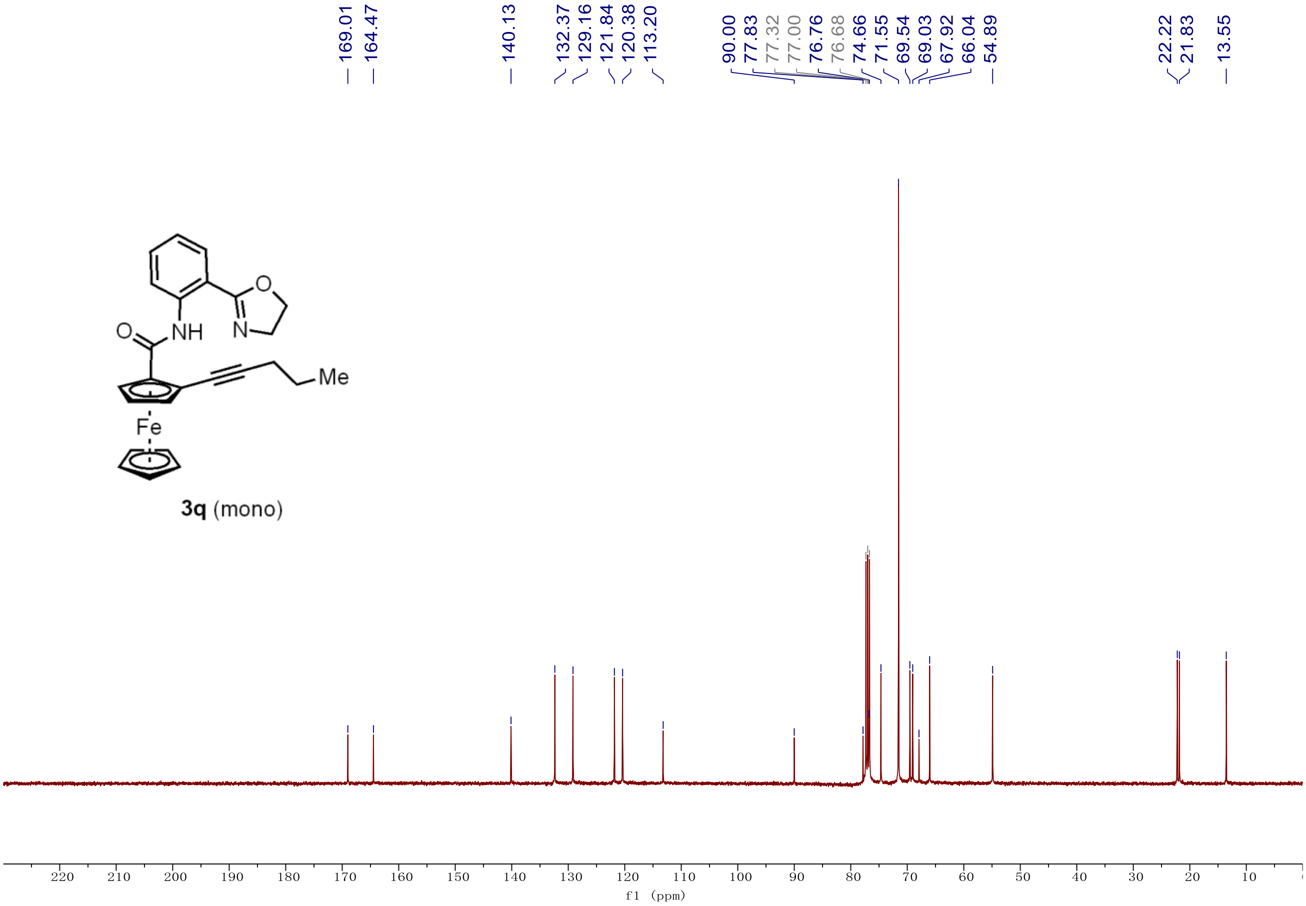
169.01
164.47
140.13
132.37
129.16
121.84
120.38
113.20
90.00
77.83
77.32
77.00
76.76
76.68
74.66
71.55
69.54
69.03
67.92
66.04
54.89
22.22
21.83
13.55
O
NH
O
N
Me
Fe
3q (mono)
220 210 200 190 180 170 160 150 140 130 120 110 100 90 80 70 60 50 40 30 20 10
f1 (ppm)

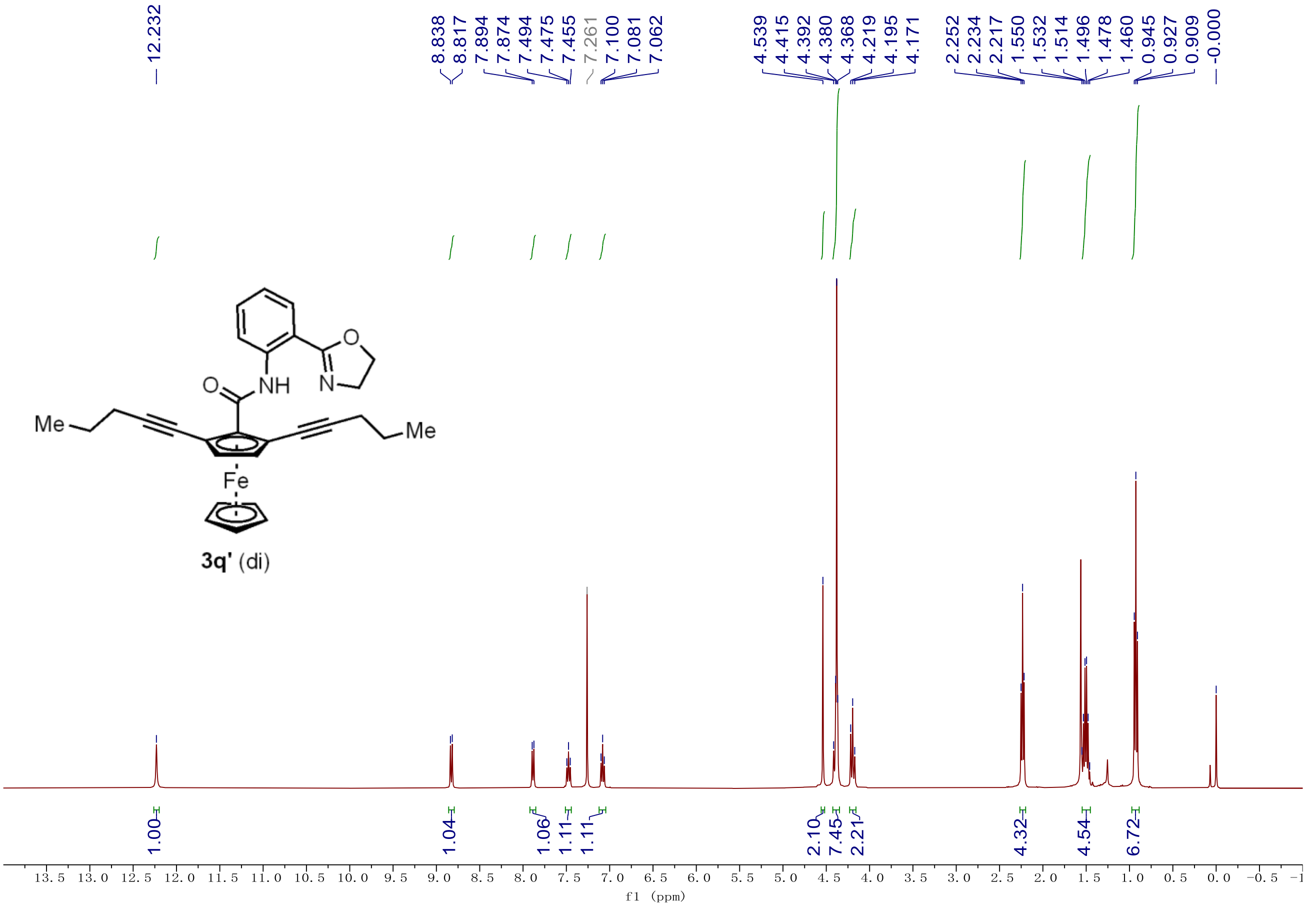
12.232
8.838
8.817
7.894
7.874
7.494
7.475
7.455
7.261
7.100
7.081
7.062
4.539
4.415
4.392
4.380
4.368
4.219
4.195
4.171
2.252
2.234
2.217
1.550
1.532
1.514
1.496
1.478
1.460
0.945
0.927
0.909
-0.000
O
NH
O
N
Me
Me
Fe
3q' (di)
1.00
1.04
1.06
1.11
1.11
2.10
7.45
2.21
4.32
4.54
6.72
13.5 13.0 12.5 12.0 11.5 11.0 10.5 10.0 9.5 9.0 8.5 8.0 7.5 7.0 6.5 6.0 5.5 5.0 4.5 4.0 3.5 3.0 2.5 2.0 1.5 1.0 0.5 0.0 -0.5 -1
f1 (ppm)

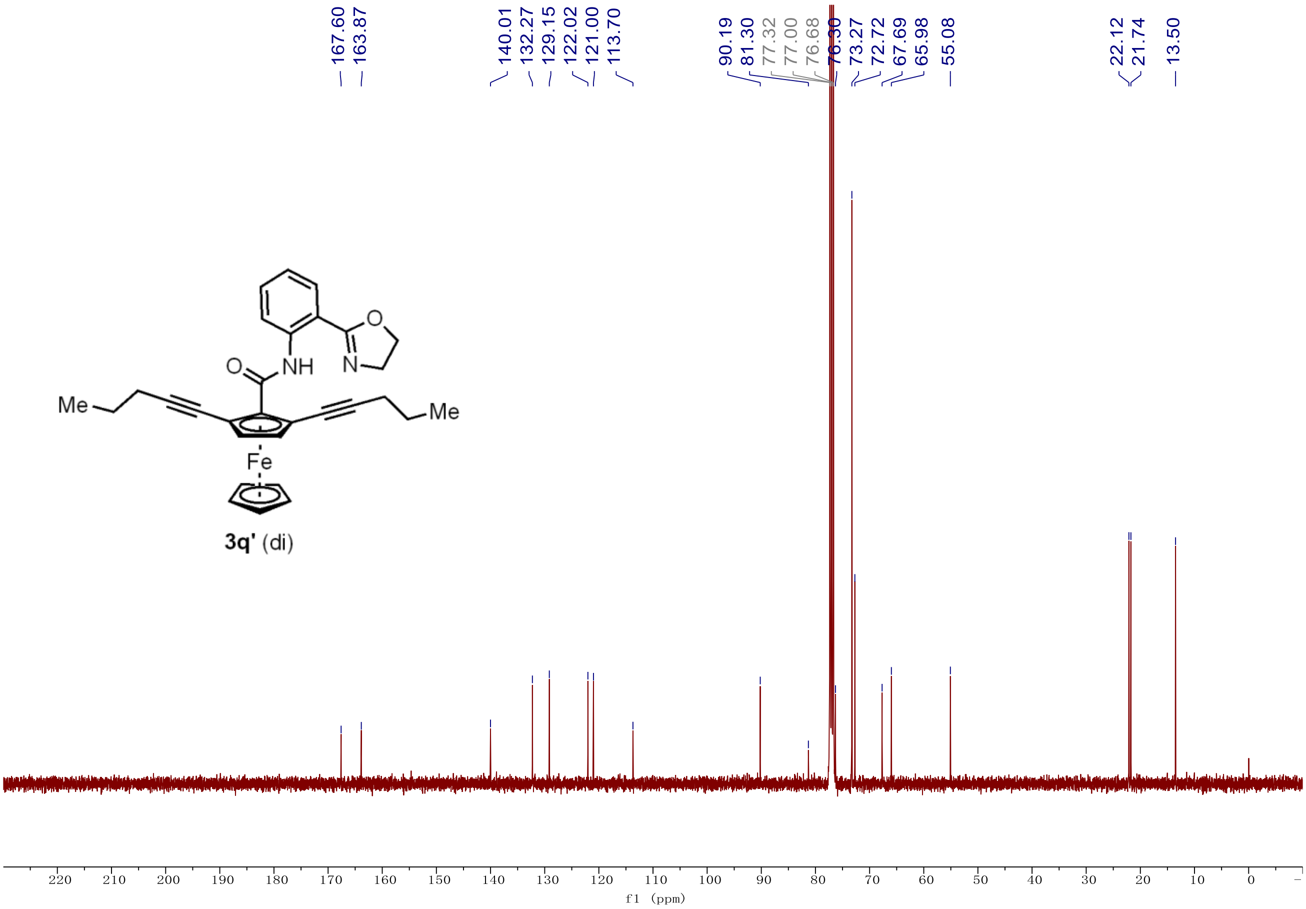
167.60
163.87
140.01
132.27
129.15
122.02
121.00
113.70
90.19
81.30
77.32
77.00
76.68
76.30
73.27
72.72
67.69
65.98
55.08
22.12
21.74
13.50
Me
Me
O
NH
N
O
Fe
3q' (di)
f1 (ppm)

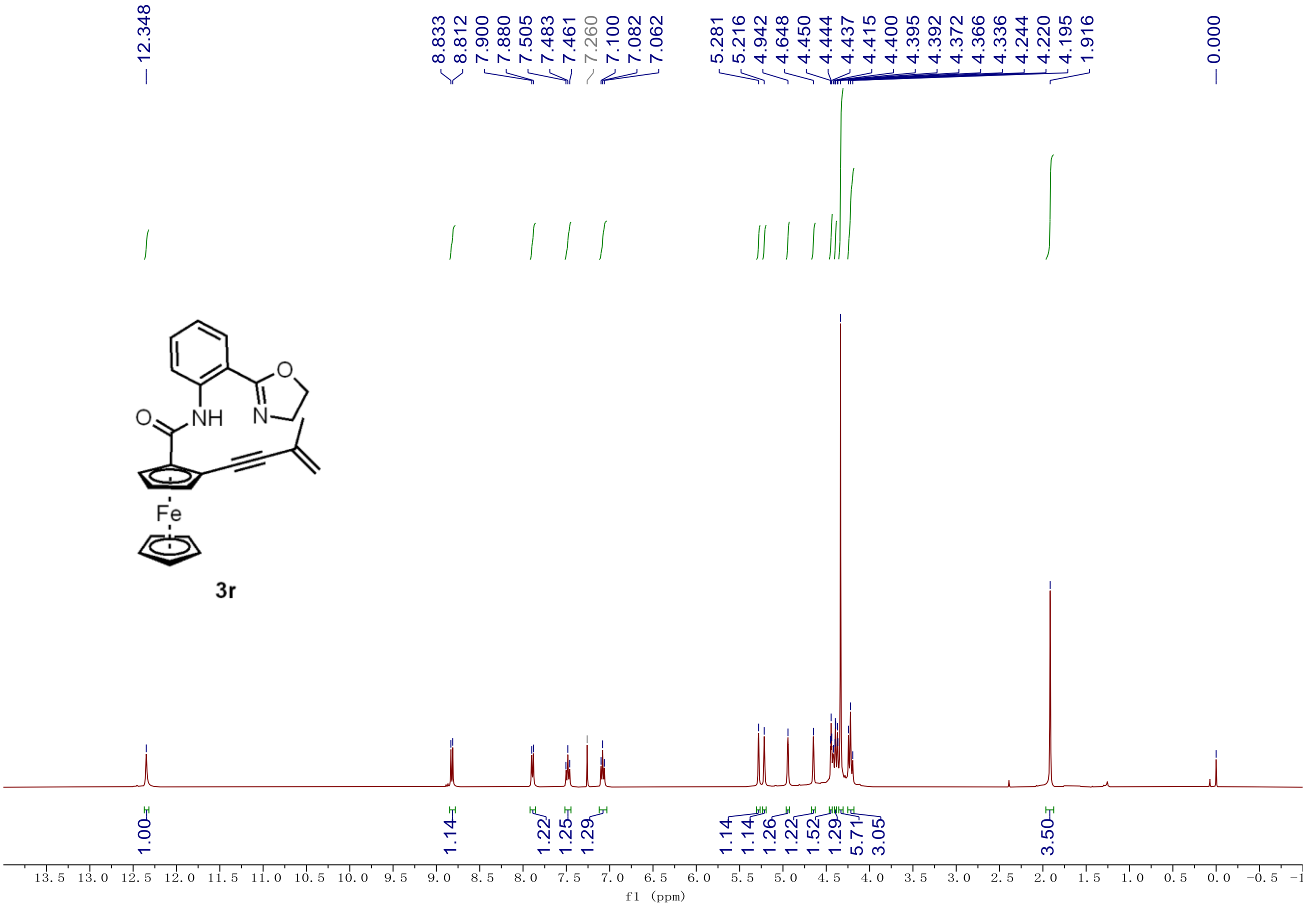
12.348
8.833
8.812
7.900
7.880
7.505
7.483
7.461
7.260
7.100
7.082
7.062
5.281
5.216
4.942
4.648
4.450
4.444
4.437
4.415
4.400
4.395
4.392
4.372
4.366
4.336
4.244
4.220
4.195
1.916
0.000
O
NH
N
O
Fe
3r
1.00
1.14
1.22
1.25
1.29
1.14
1.14
1.26
1.22
1.52
1.29
5.71
3.05
3.50
f1 (ppm)

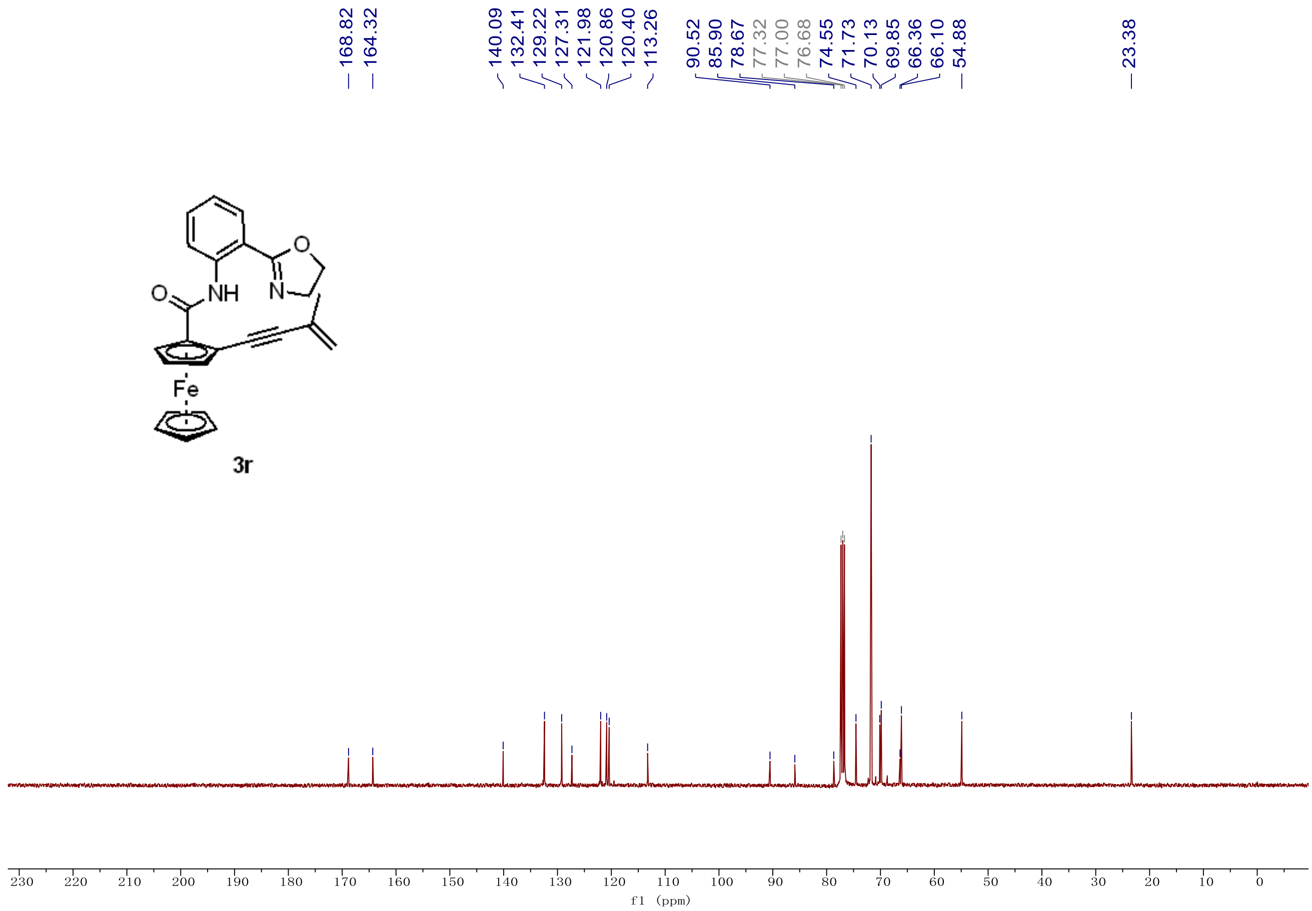
168.82
164.32
140.09
132.41
129.22
127.31
121.98
120.86
120.40
113.26
90.52
85.90
78.67
77.32
77.00
76.68
74.55
71.73
70.13
69.85
66.36
66.10
54.88
23.38
O
NH
N
O
Fe
3r
f1 (ppm)

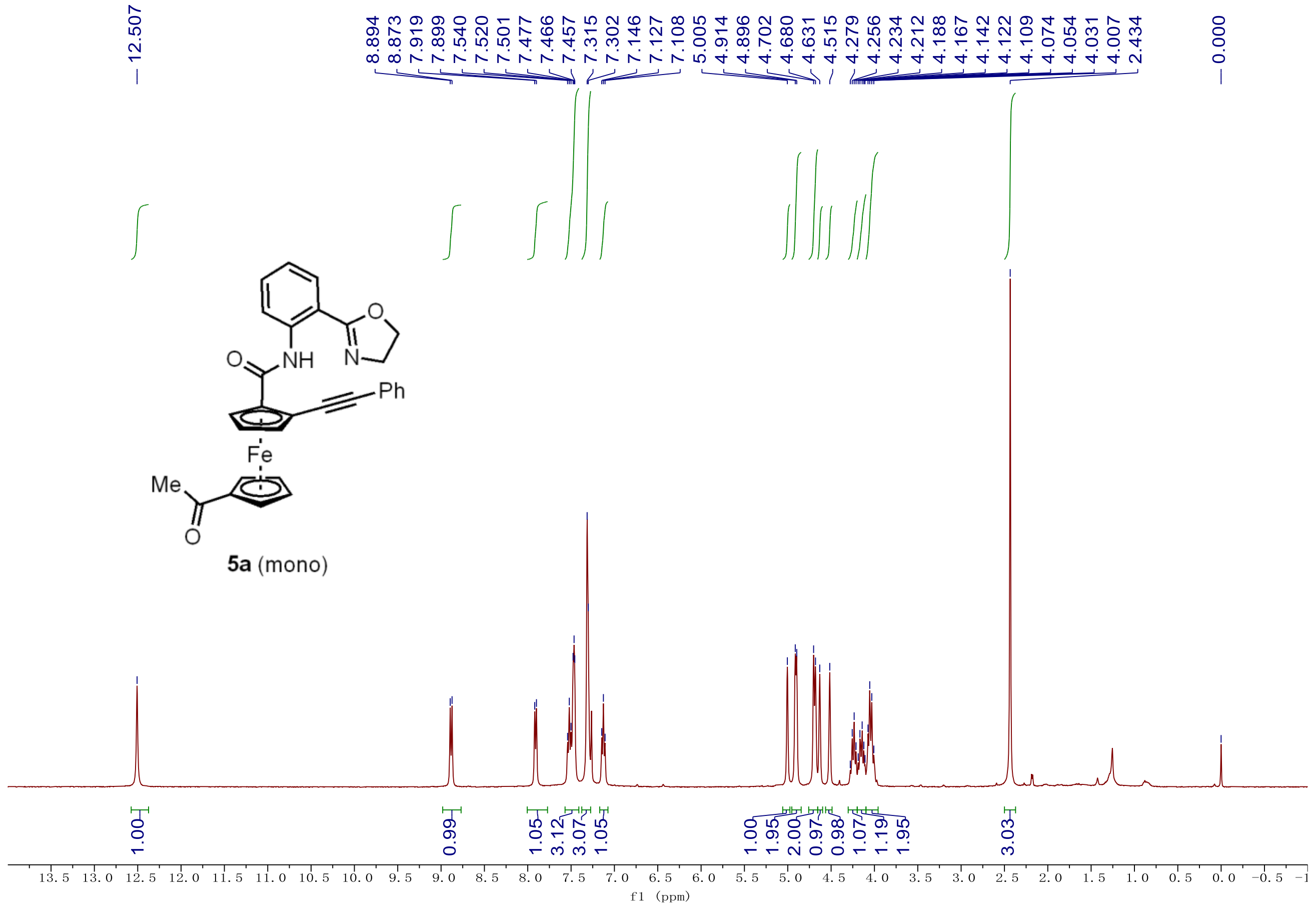
12.507
8.894
8.873
7.919
7.899
7.540
7.520
7.501
7.477
7.466
7.457
7.315
7.302
7.146
7.127
7.108
5.005
4.914
4.896
4.702
4.680
4.631
4.515
4.279
4.256
4.234
4.212
4.188
4.167
4.142
4.122
4.109
4.074
4.054
4.031
4.007
2.434
0.000
O
NH
N
O
Ph
Fe
Me
O
5a (mono)
1.00
0.99
1.05
3.12
3.07
1.05
1.00
1.95
2.00
0.97
0.98
1.07
1.19
1.95
3.03
f1 (ppm)

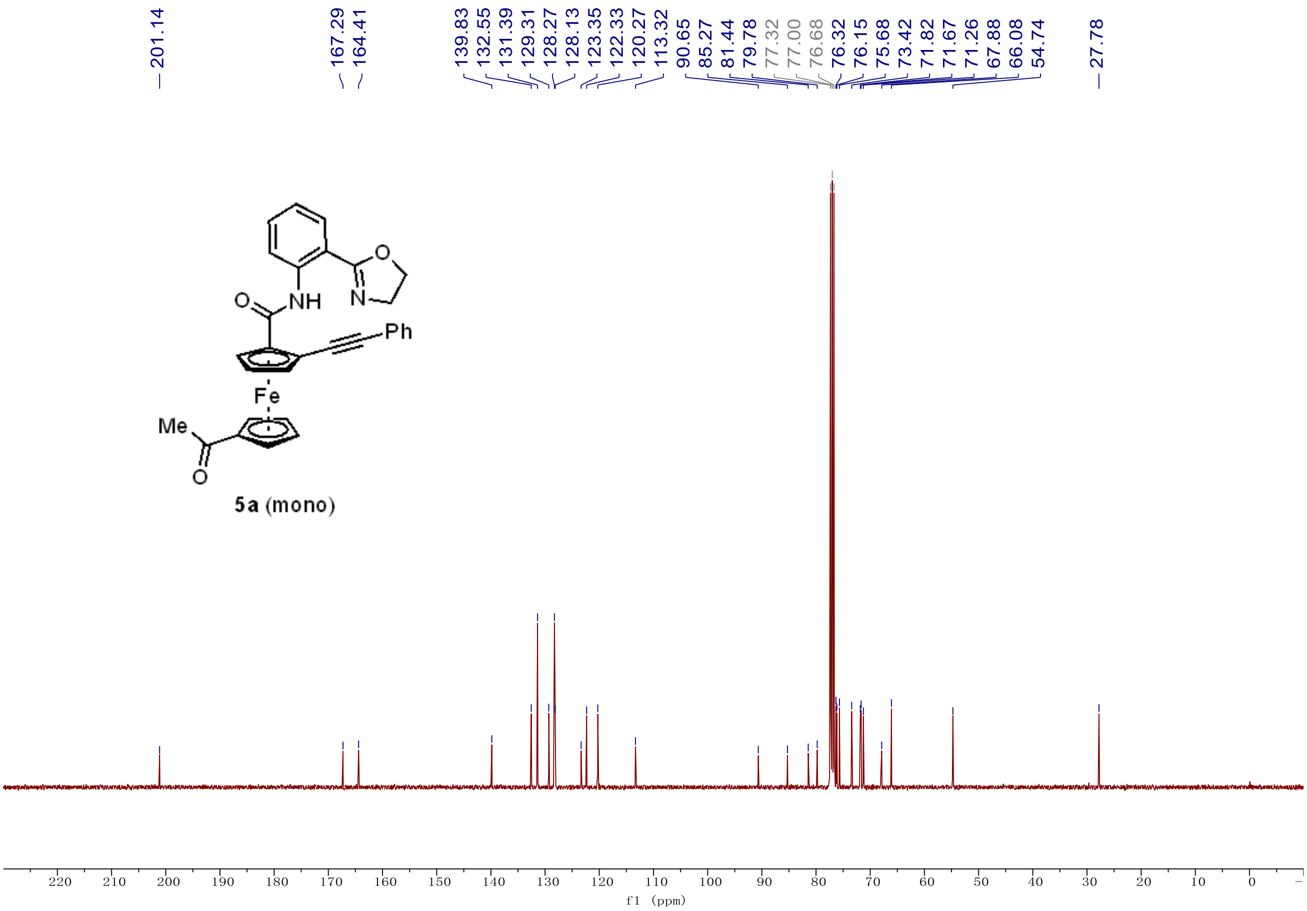
201.14
167.29
164.41
139.83
132.55
131.39
129.31
128.27
128.13
123.35
122.33
120.27
113.32
90.65
85.27
81.44
79.78
77.32
77.00
76.68
76.32
76.15
75.68
73.42
71.82
71.67
71.26
67.88
66.08
54.74
27.78
O
NH
N
O
Ph
Fe
Me
O
5a (mono)
220 210 200 190 180 170 160 150 140 130 120 110 100 90 80 70 60 50 40 30 20 10 0
f1 (ppm)

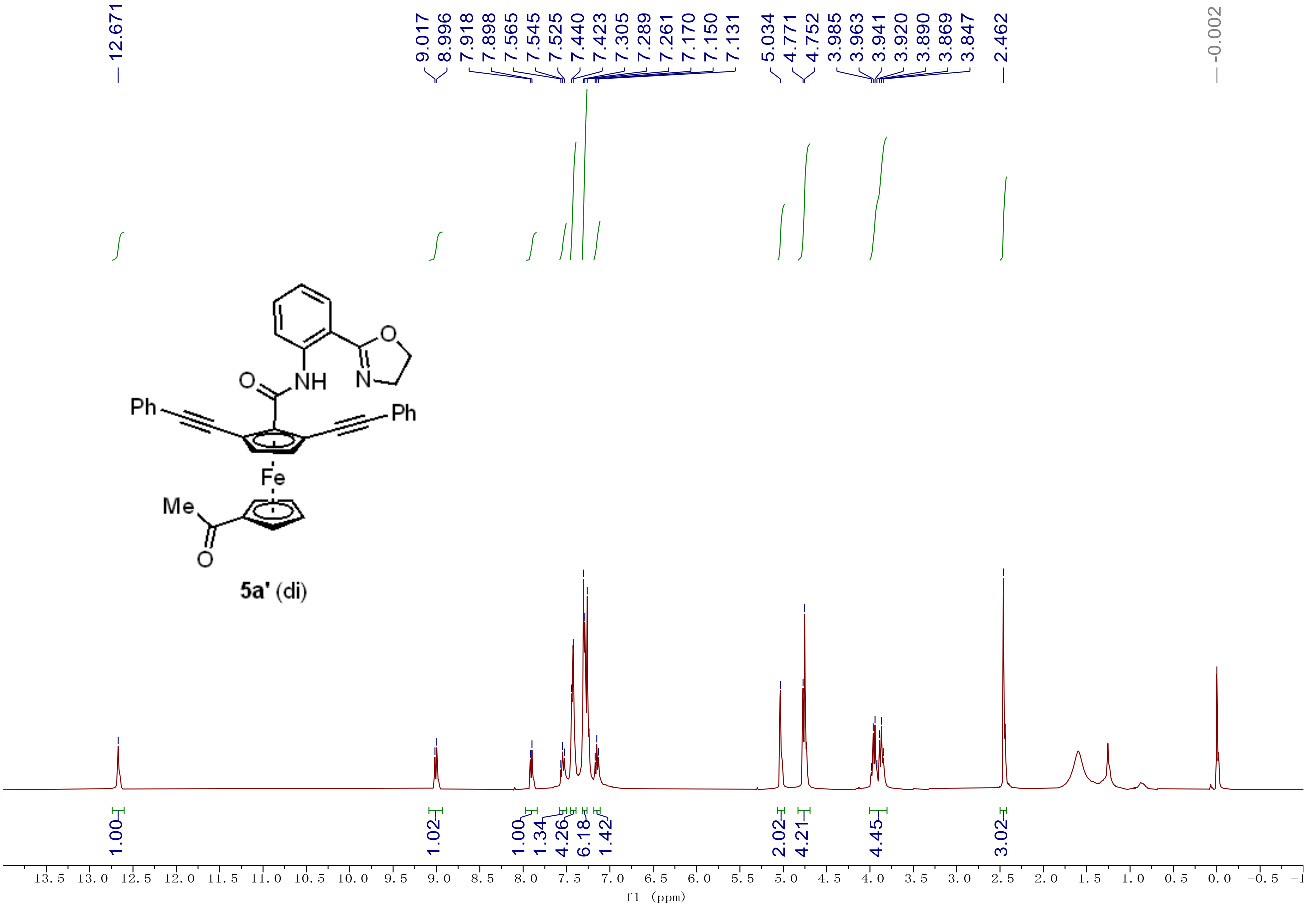
12.671
9.017
8.996
7.918
7.898
7.565
7.545
7.525
7.440
7.423
7.305
7.289
7.261
7.170
7.150
7.131
5.034
4.771
4.752
3.985
3.963
3.941
3.920
3.890
3.869
3.847
2.462
-0.002
O
NH
N
O
Ph
Ph
Fe
Me
O
5a' (di)
1.00
1.02
1.00
1.34
4.26
6.18
1.42
2.02
4.21
4.45
3.02
13.5 13.0 12.5 12.0 11.5 11.0 10.5 10.0 9.5 9.0 8.5 8.0 7.5 7.0 6.5 6.0 5.5 5.0 4.5 4.0 3.5 3.0 2.5 2.0 1.5 1.0 0.5 0.0 -0.5 -1
f1 (ppm)

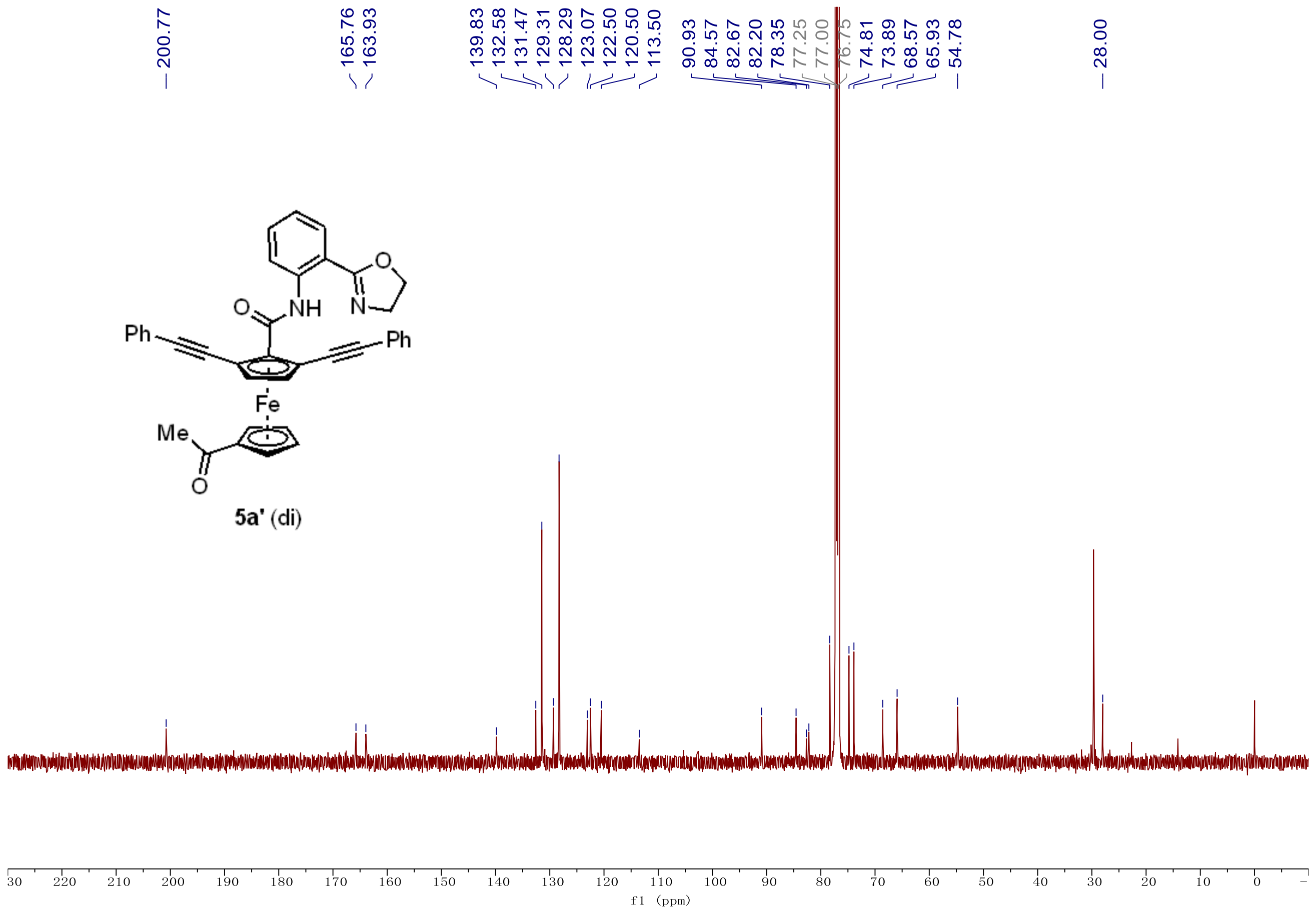
200.77
165.76
163.93
139.83
132.58
131.47
129.31
128.29
123.07
122.50
120.50
113.50
90.93
84.57
82.67
82.20
78.35
77.25
77.00
76.75
74.81
73.89
68.57
65.93
54.78
28.00
Ph
Ph
O
NH
O
N
Fe
Me
O
5a' (di)
f1 (ppm)

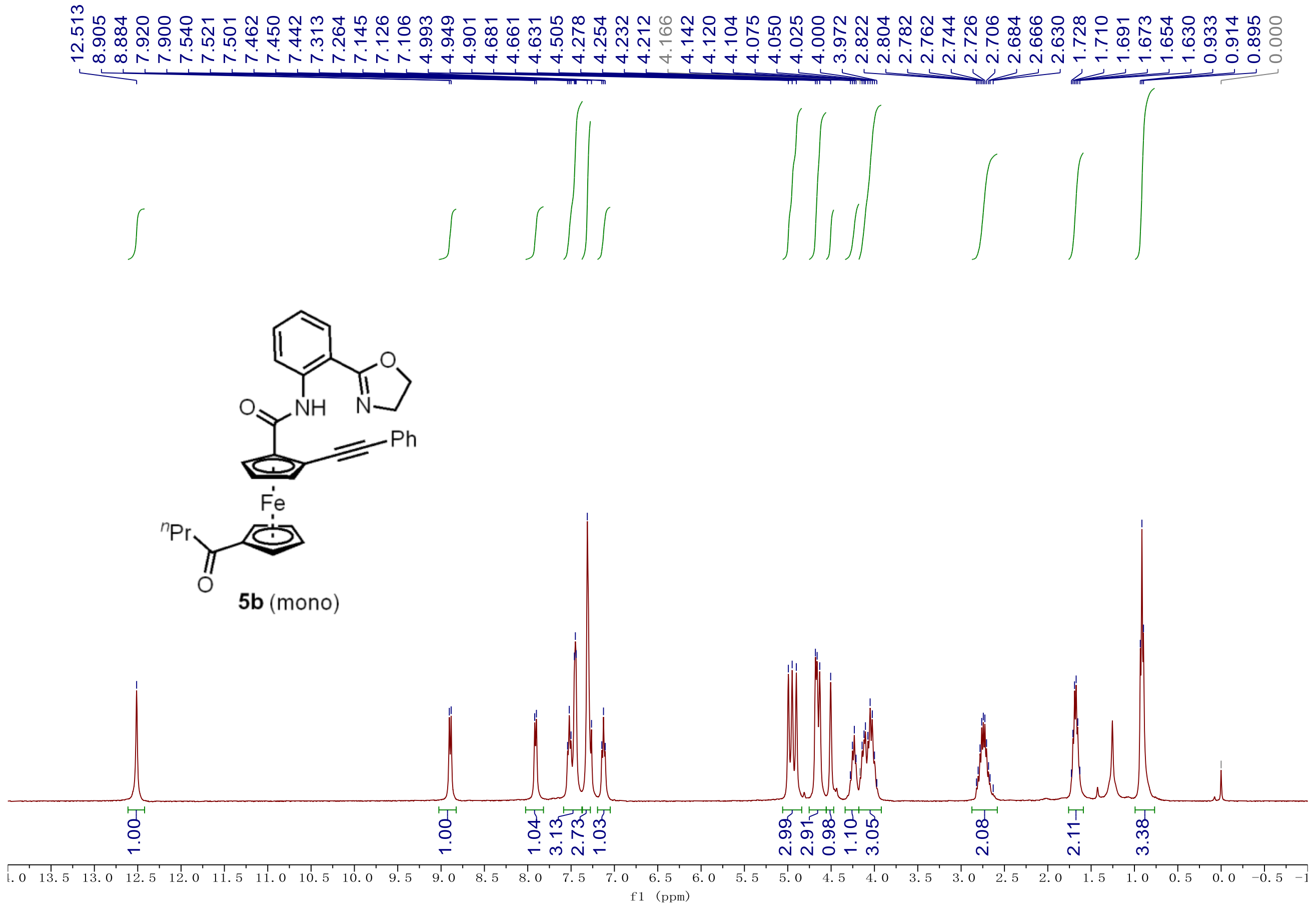
12.513
8.905
8.884
7.920
7.900
7.540
7.521
7.501
7.462
7.450
7.442
7.313
7.264
7.145
7.126
7.106
4.993
4.949
4.901
4.681
4.661
4.631
4.505
4.278
4.254
4.232
4.212
4.166
4.142
4.120
4.104
4.075
4.050
4.025
4.000
3.972
2.822
2.804
2.782
2.762
2.744
2.726
2.706
2.684
2.666
2.630
1.728
1.710
1.691
1.673
1.654
1.630
0.933
0.914
0.895
0.000
O
NH
O
N
Ph
Fe
^{n}Pr
O
5b (mono)
1.00
1.00
1.04
3.13
2.73
1.03
2.99
2.91
0.98
1.10
3.05
2.08
2.11
3.38
f1 (ppm)

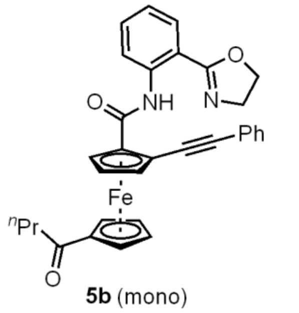

5b (mono)

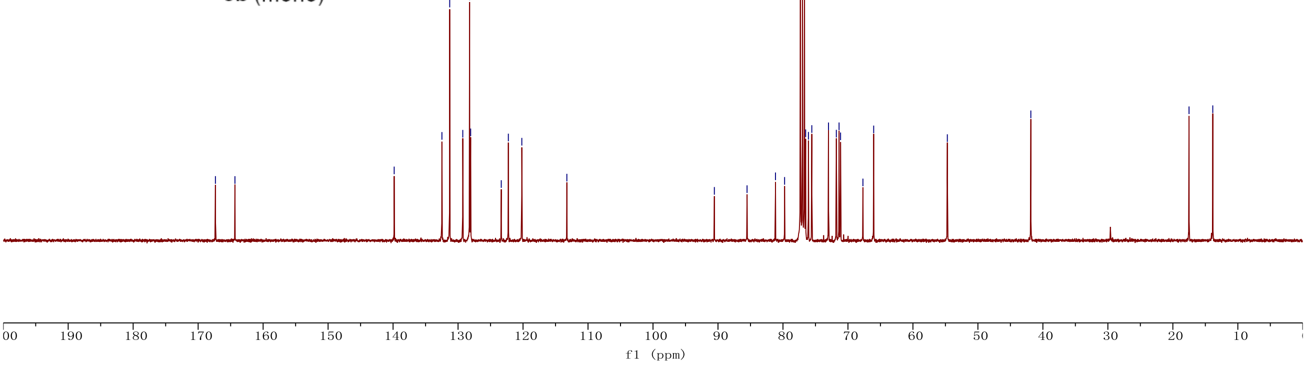
167.34
164.34
139.83
132.48
131.29
129.28
128.23
128.06
123.35
122.26
120.19
113.26
90.56
85.53
81.15
79.75
77.32
77.00
76.68
76.51
76.08
75.56
72.99
71.78
71.37
71.13
67.69
66.04
54.70
41.85
17.51
13.85
f1 (ppm)

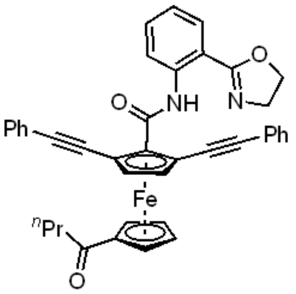

5b' (di)

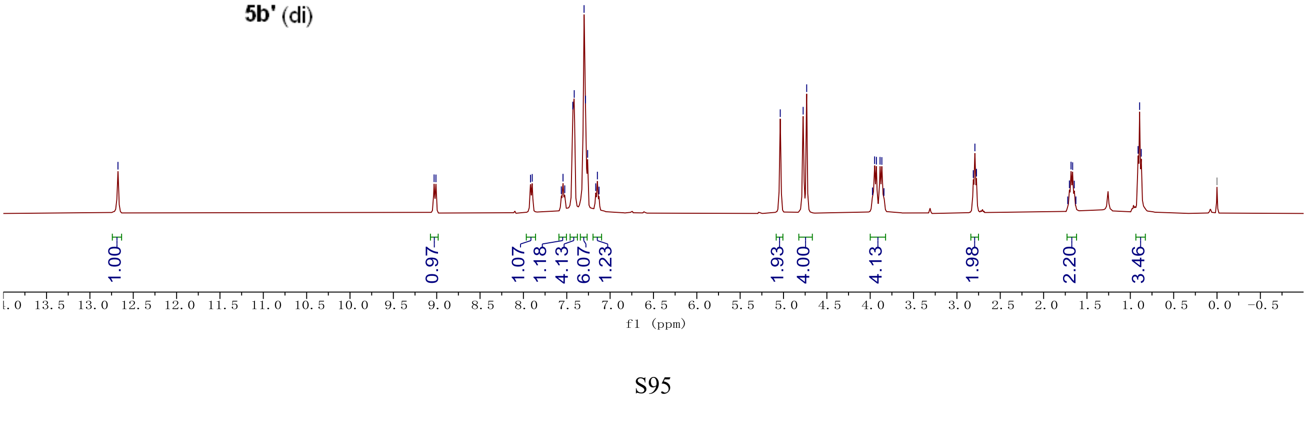
12.676
9.030
9.009
7.918
7.563
7.543
7.523
7.430
7.414
7.300
7.284
7.259
7.166
7.147
7.128
5.038
4.773
4.733
3.974
3.949
3.928
3.886
3.866
3.841
2.811
2.793
2.774
1.722
1.702
1.684
1.665
1.647
1.628
0.910
0.893
0.874
0.000
1.00
0.97
1.07
1.18
4.13
6.07
1.23
1.93
4.00
4.13
1.98
2.20
3.46
f1 (ppm)

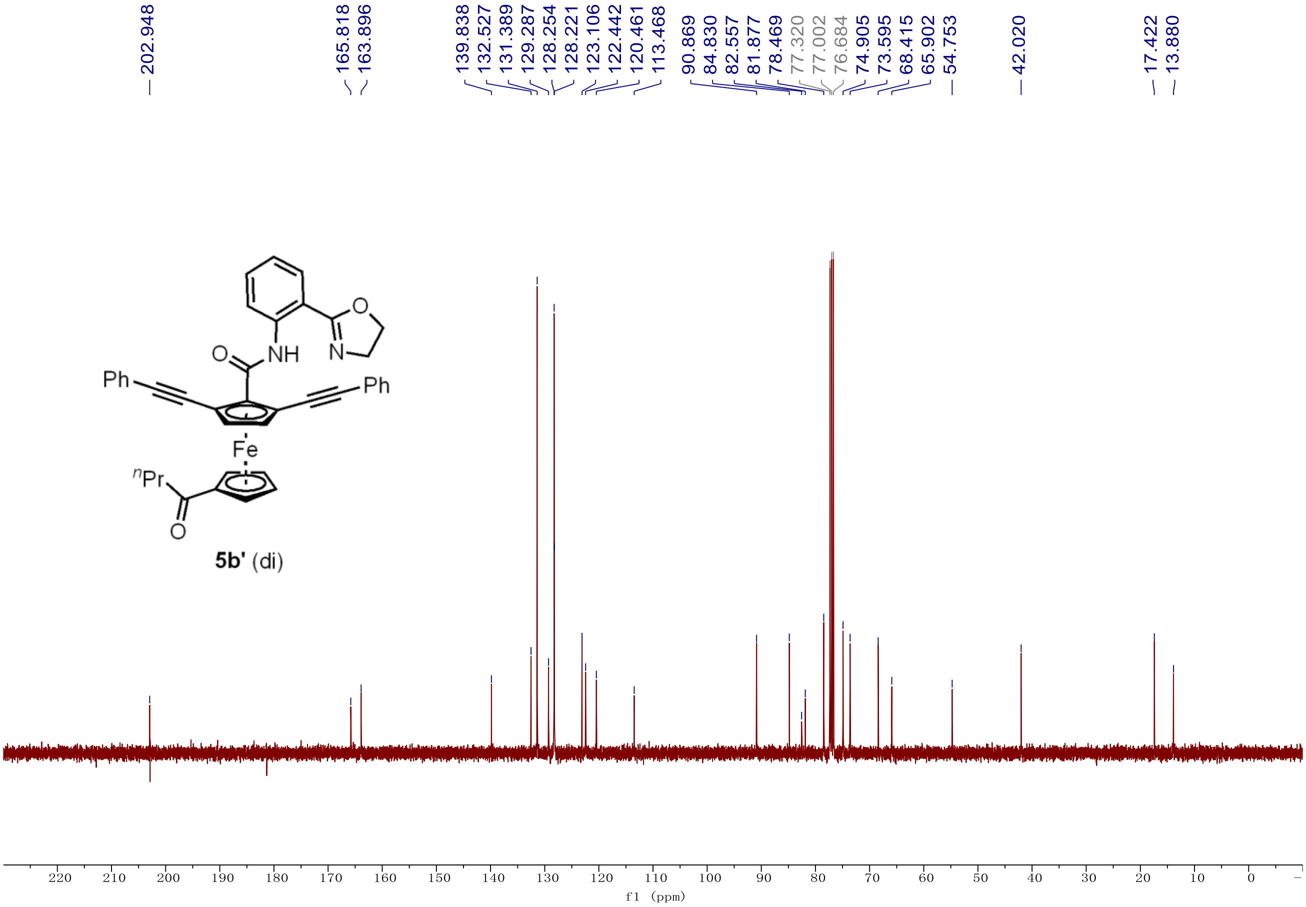
202.948
165.818
163.896
139.838
132.527
131.389
129.287
128.254
128.221
123.106
122.442
120.461
113.468
90.869
84.830
82.557
81.877
78.469
77.320
77.002
76.684
74.905
73.595
68.415
65.902
54.753
42.020
17.422
13.880
Ph
O
NH
N
O
Ph
Fe
nPr
O
5b' (di)
f1 (ppm)

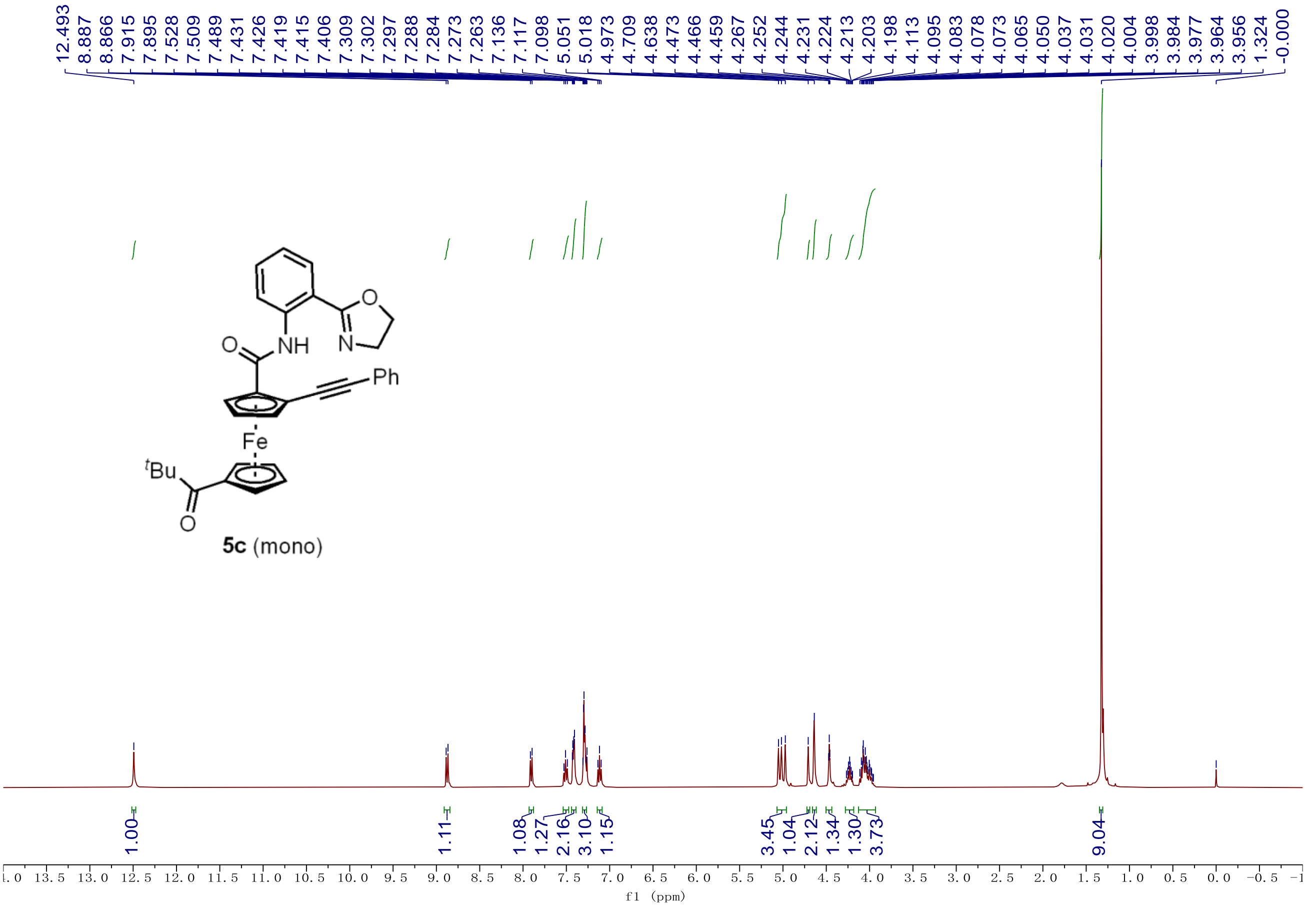
12.493
8.887
8.866
7.915
7.895
7.528
7.509
7.489
7.431
7.426
7.419
7.415
7.406
7.309
7.302
7.297
7.288
7.284
7.273
7.263
7.136
7.117
7.098
5.051
5.018
4.973
4.709
4.638
4.473
4.466
4.459
4.267
4.252
4.244
4.231
4.224
4.213
4.203
4.198
4.113
4.095
4.083
4.078
4.073
4.065
4.050
4.037
4.031
4.020
4.004
3.998
3.984
3.977
3.964
3.956
1.324
-0.000
O
NH
N
O
Ph
Fe
tBu
O
5c (mono)
1.00
1.11
1.08
1.27
2.16
3.10
1.15
3.45
1.04
2.12
1.34
1.30
3.73
9.04
f1 (ppm)

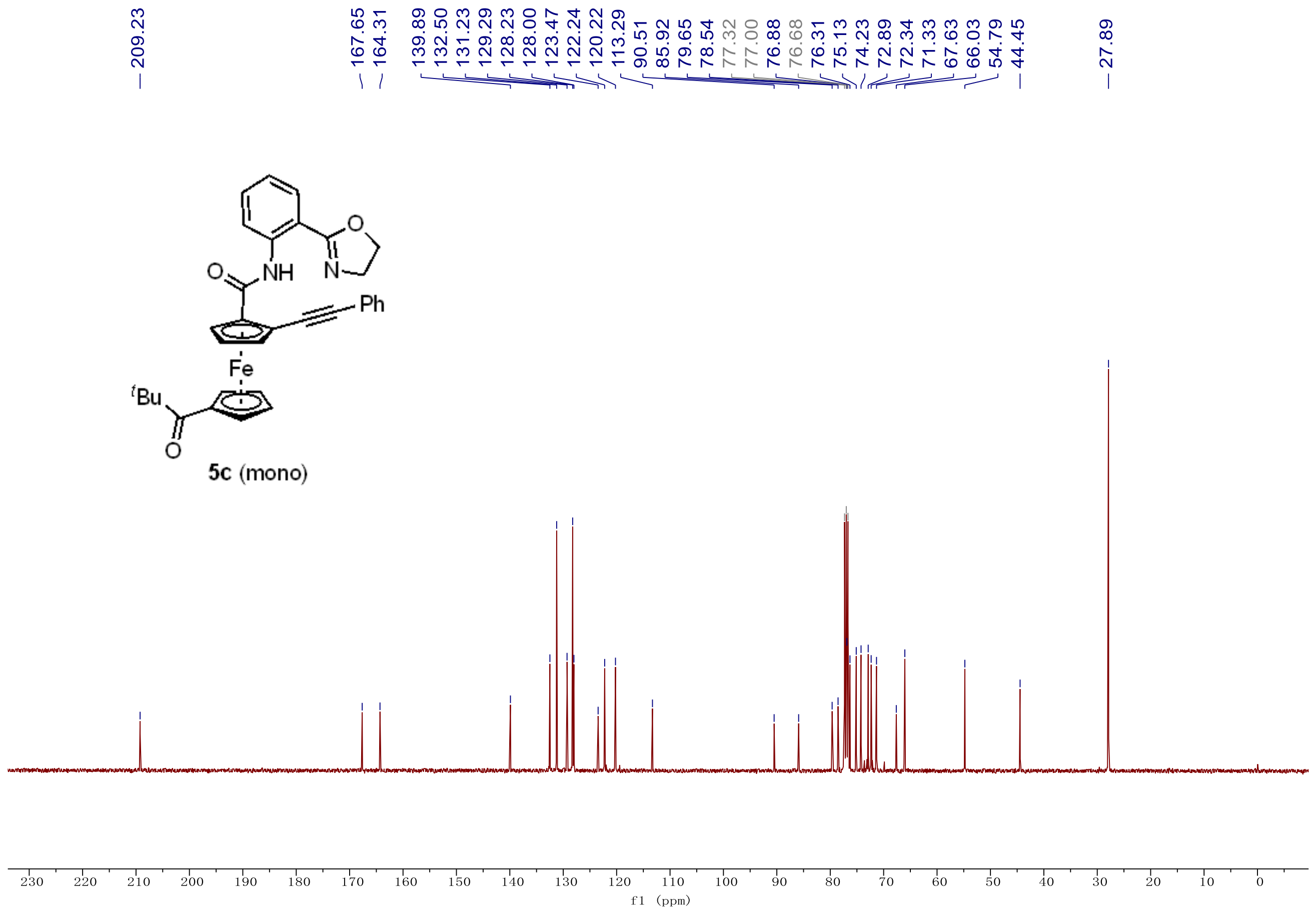
209.23
167.65
164.31
139.89
132.50
131.23
129.29
128.23
128.00
123.47
122.24
120.22
113.29
90.51
85.92
79.65
78.54
77.32
77.00
76.88
76.68
76.31
75.13
74.23
72.89
72.34
71.33
67.63
66.03
54.79
44.45
27.89
O
NH
N
O
Ph
Fe
tBu
O
5c (mono)
230 220 210 200 190 180 170 160 150 140 130 120 110 100 90 80 70 60 50 40 30 20 10 0
f1 (ppm)

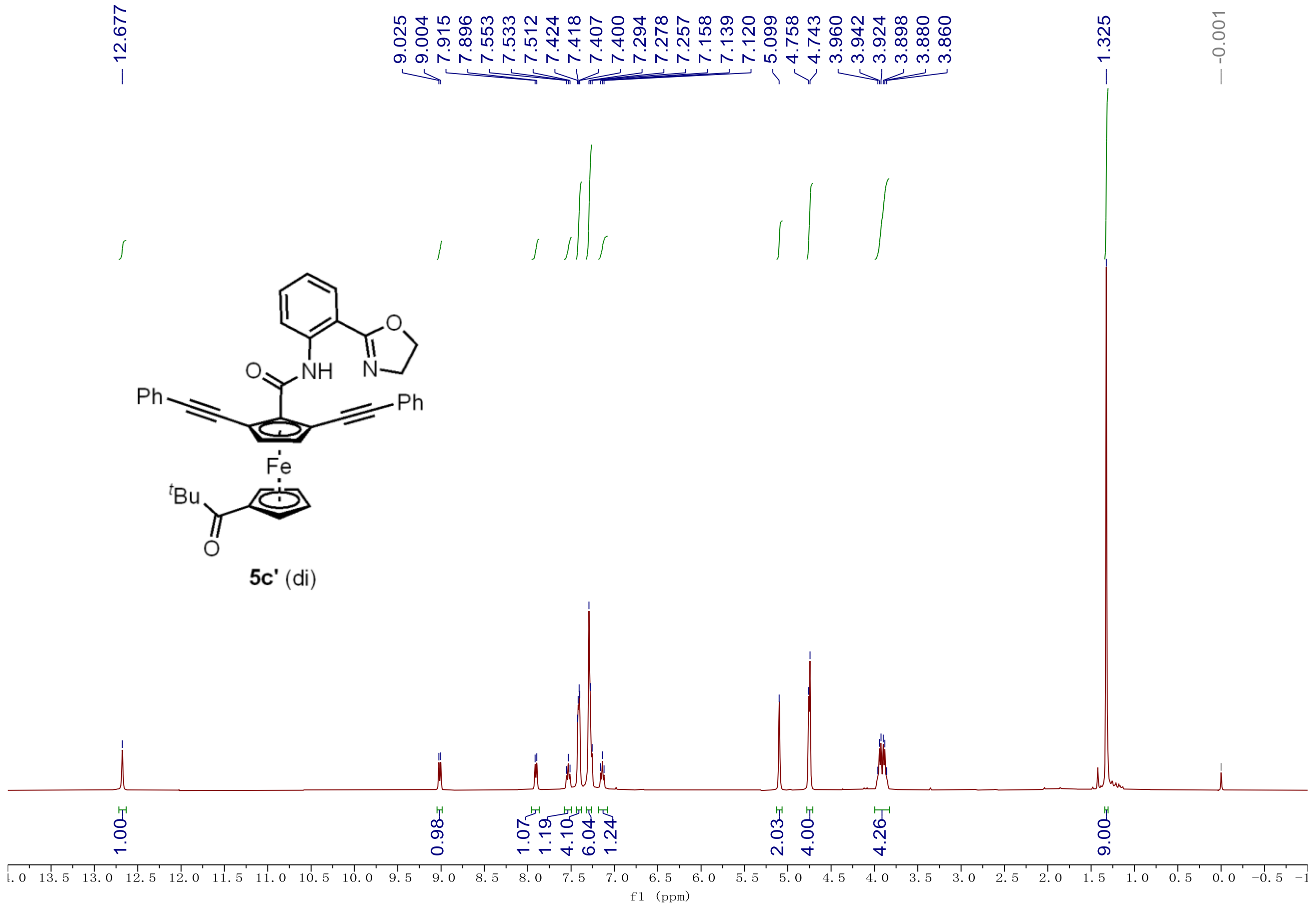
12.677
9.025
9.004
7.915
7.896
7.553
7.533
7.512
7.424
7.418
7.407
7.400
7.294
7.278
7.257
7.158
7.139
7.120
5.099
4.758
4.743
3.960
3.942
3.924
3.898
3.880
3.860
1.325
-0.001
O
NH
N
O
Ph
Ph
Fe
tBu
O
5c' (di)
1.00
0.98
1.07
1.19
4.10
6.04
1.24
2.03
4.00
4.26
9.00
13.5 13.0 12.5 12.0 11.5 11.0 10.5 10.0 9.5 9.0 8.5 8.0 7.5 7.0 6.5 6.0 5.5 5.0 4.5 4.0 3.5 3.0 2.5 2.0 1.5 1.0 0.5 0.0 -0.5
f1 (ppm)

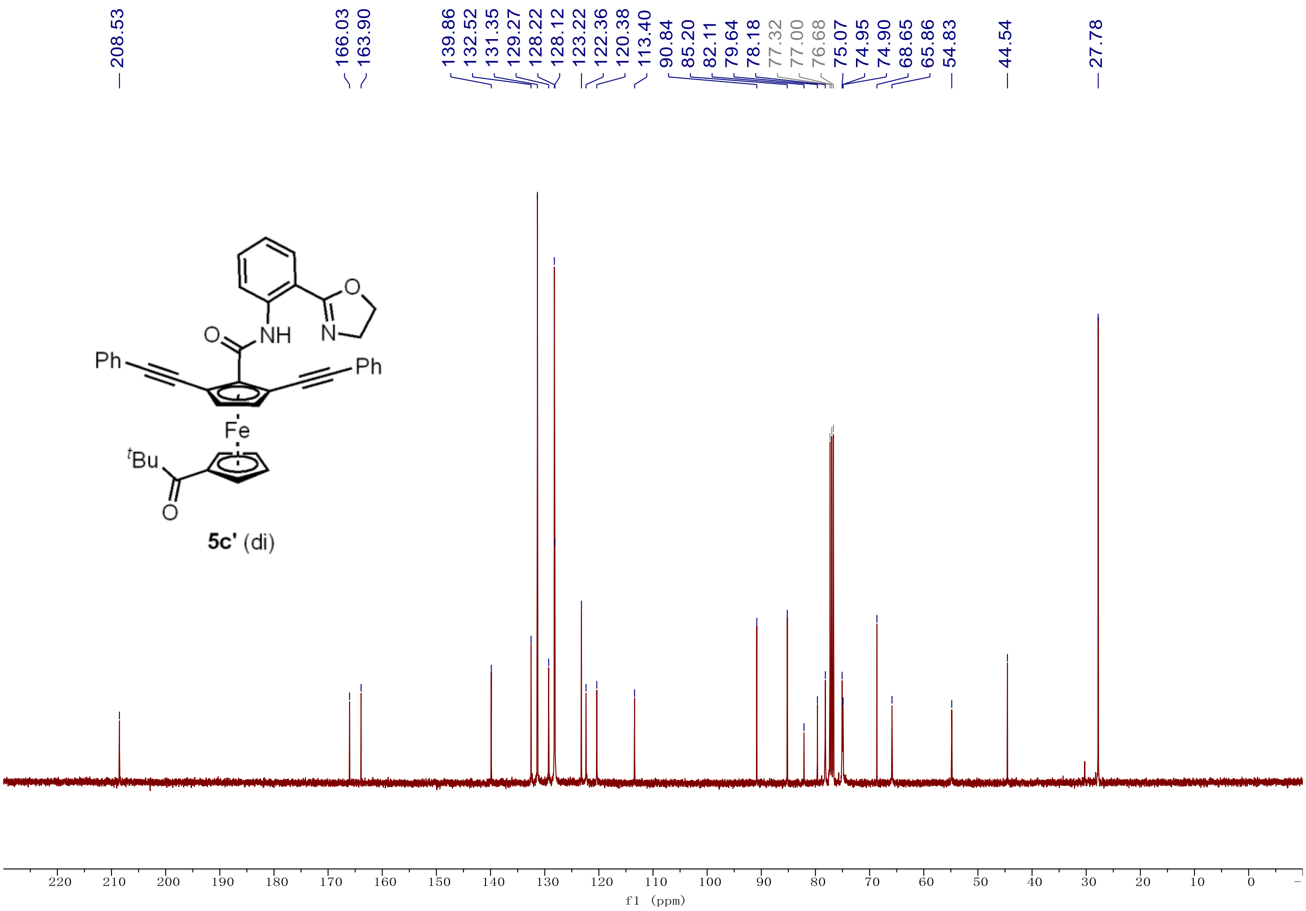
208.53
166.03
163.90
139.86
132.52
131.35
129.27
128.22
128.12
123.22
122.36
120.38
113.40
90.84
85.20
82.11
79.64
78.18
77.32
77.00
76.68
75.07
74.95
74.90
68.65
65.86
54.83
44.54
27.78
Ph
O
NH
N
O
Ph
Fe
^{t}Bu
O
5c' (di)
f1 (ppm)

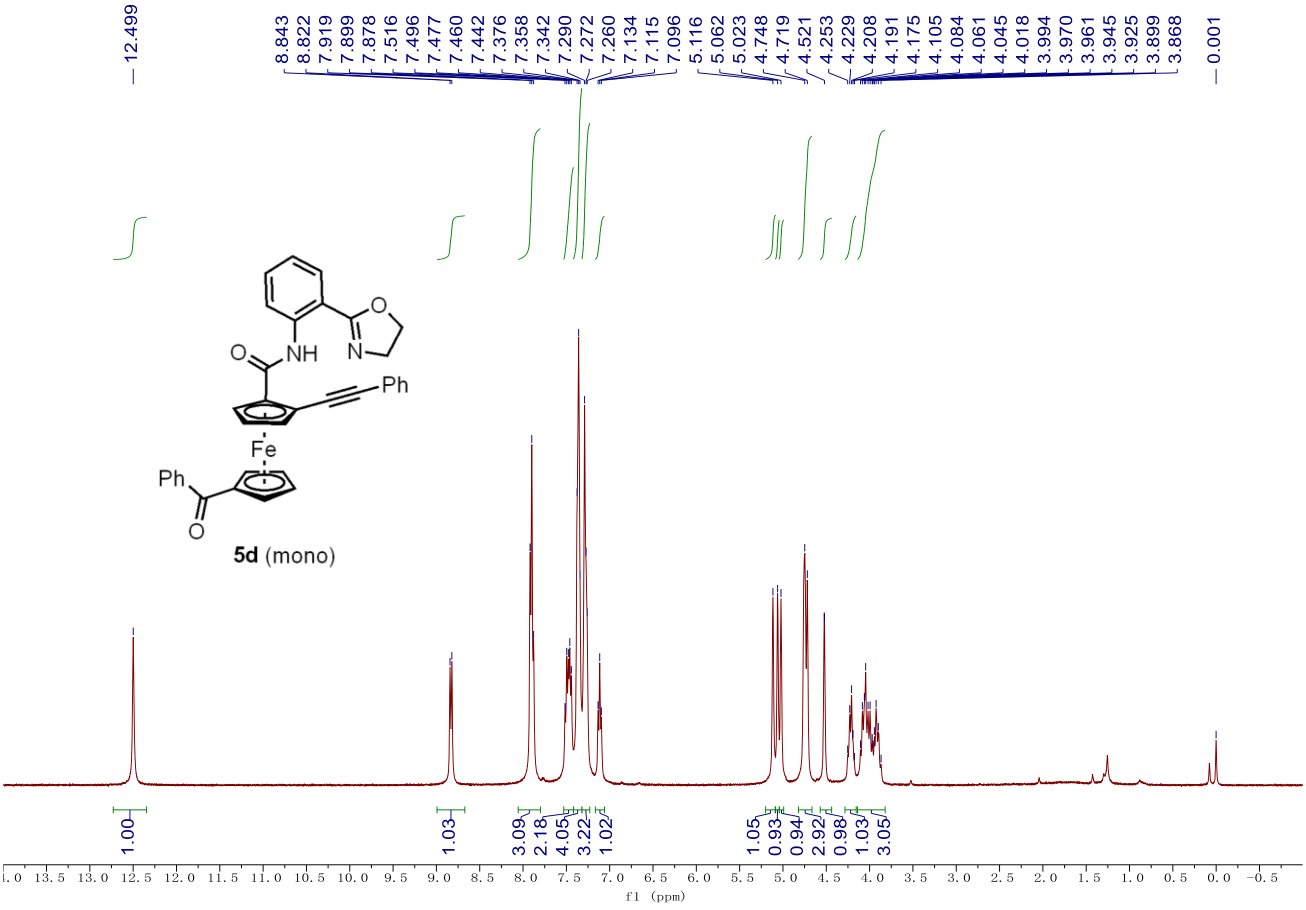
12.499
8.843
8.822
7.919
7.899
7.878
7.516
7.496
7.477
7.460
7.442
7.376
7.358
7.342
7.290
7.272
7.260
7.134
7.115
7.096
5.116
5.062
5.023
4.748
4.719
4.521
4.253
4.229
4.208
4.191
4.175
4.105
4.084
4.061
4.045
4.018
3.994
3.970
3.961
3.945
3.925
3.899
3.868
0.001
O
NH
N
O
Ph
Fe
Ph
O
5d (mono)
1.00
1.03
3.09
2.18
4.05
3.22
1.02
1.05
0.93
0.94
2.92
0.98
1.03
3.05
f1 (ppm)

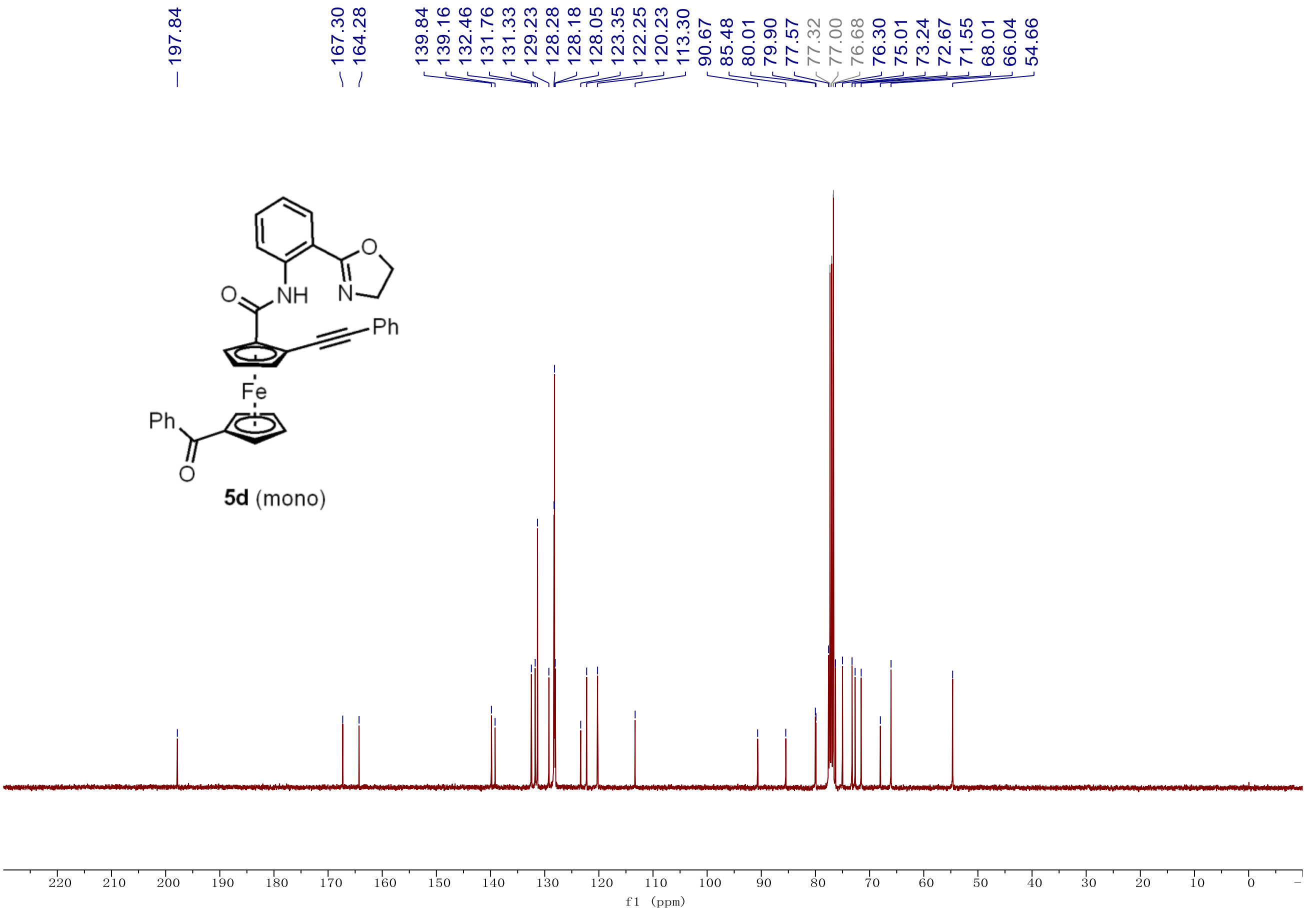
197.84
167.30
164.28
139.84
139.16
132.46
131.76
131.33
129.23
128.28
128.18
128.05
123.35
122.25
120.23
113.30
90.67
85.48
80.01
79.90
77.57
77.32
77.00
76.68
76.30
75.01
73.24
72.67
71.55
68.01
66.04
54.66
5d (mono)
f1 (ppm)

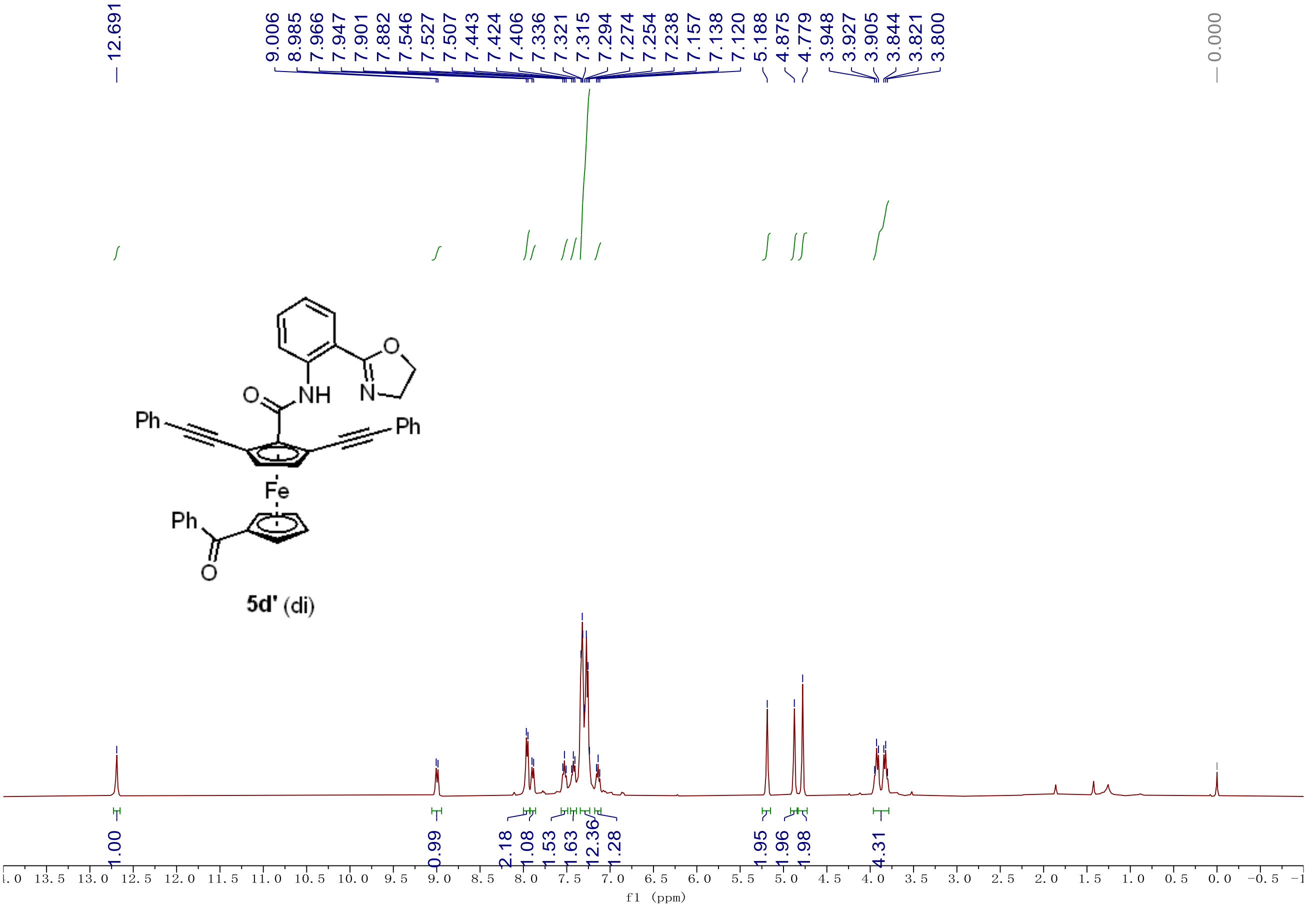
12.691
9.006
8.985
7.966
7.947
7.901
7.882
7.546
7.527
7.507
7.443
7.424
7.406
7.336
7.321
7.315
7.294
7.274
7.254
7.238
7.157
7.138
7.120
5.188
4.875
4.779
3.948
3.927
3.905
3.844
3.821
3.800
0.000
5d' (di)
1.00
0.99
2.18
1.08
1.53
1.63
12.36
1.28
1.95
1.96
1.98
4.31
f1 (ppm)

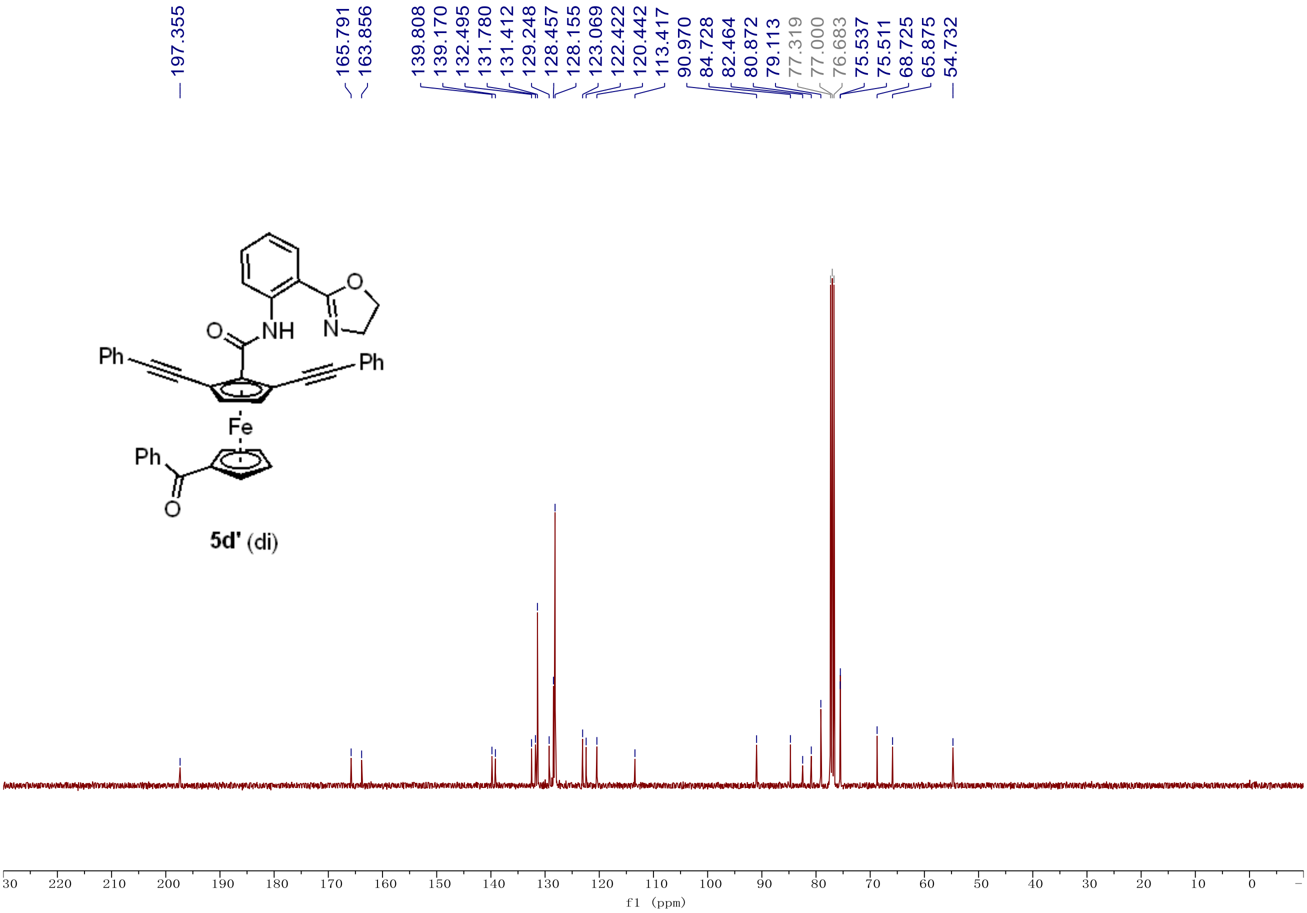
197.355
165.791
163.856
139.808
139.170
132.495
131.780
131.412
129.248
128.457
128.155
123.069
122.422
120.442
113.417
90.970
84.728
82.464
80.872
79.113
77.319
77.000
76.683
75.537
75.511
68.725
65.875
54.732
Ph
O
NH
N
O
Ph
Fe
Ph
O
5d' (di)
f1 (ppm)

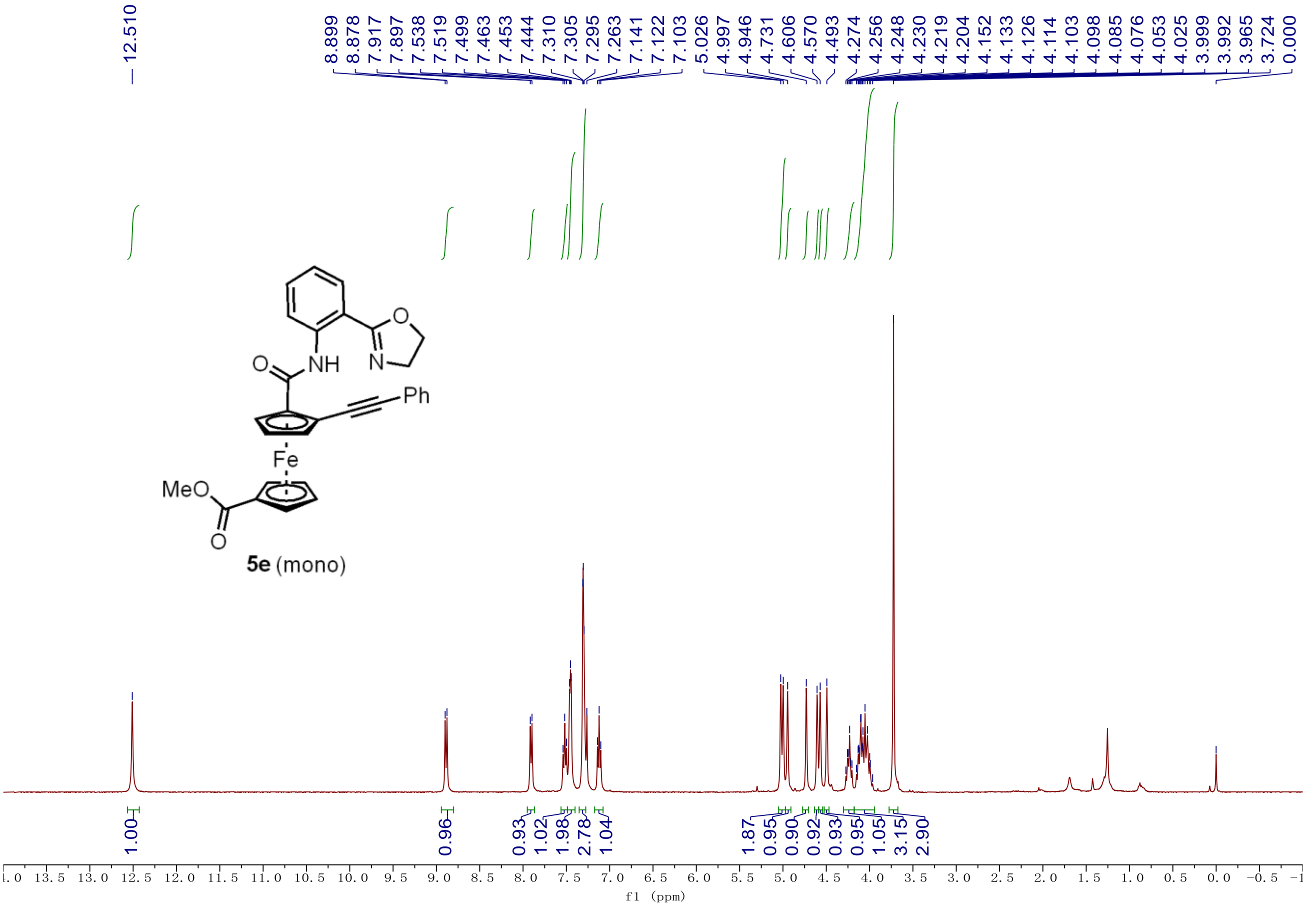
12.510
8.899
8.878
7.917
7.897
7.538
7.519
7.499
7.463
7.453
7.444
7.310
7.305
7.295
7.263
7.141
7.122
7.103
5.026
4.997
4.946
4.731
4.606
4.570
4.493
4.274
4.256
4.248
4.230
4.219
4.204
4.152
4.133
4.126
4.114
4.103
4.098
4.085
4.076
4.053
4.025
3.999
3.992
3.965
3.724
0.000
O
NH
N
O
Ph
Fe
MeO
O
5e (mono)
1.00
0.96
0.93
1.02
1.98
2.78
1.04
1.87
0.95
0.90
0.92
0.93
0.95
1.05
3.15
2.90
f1 (ppm)

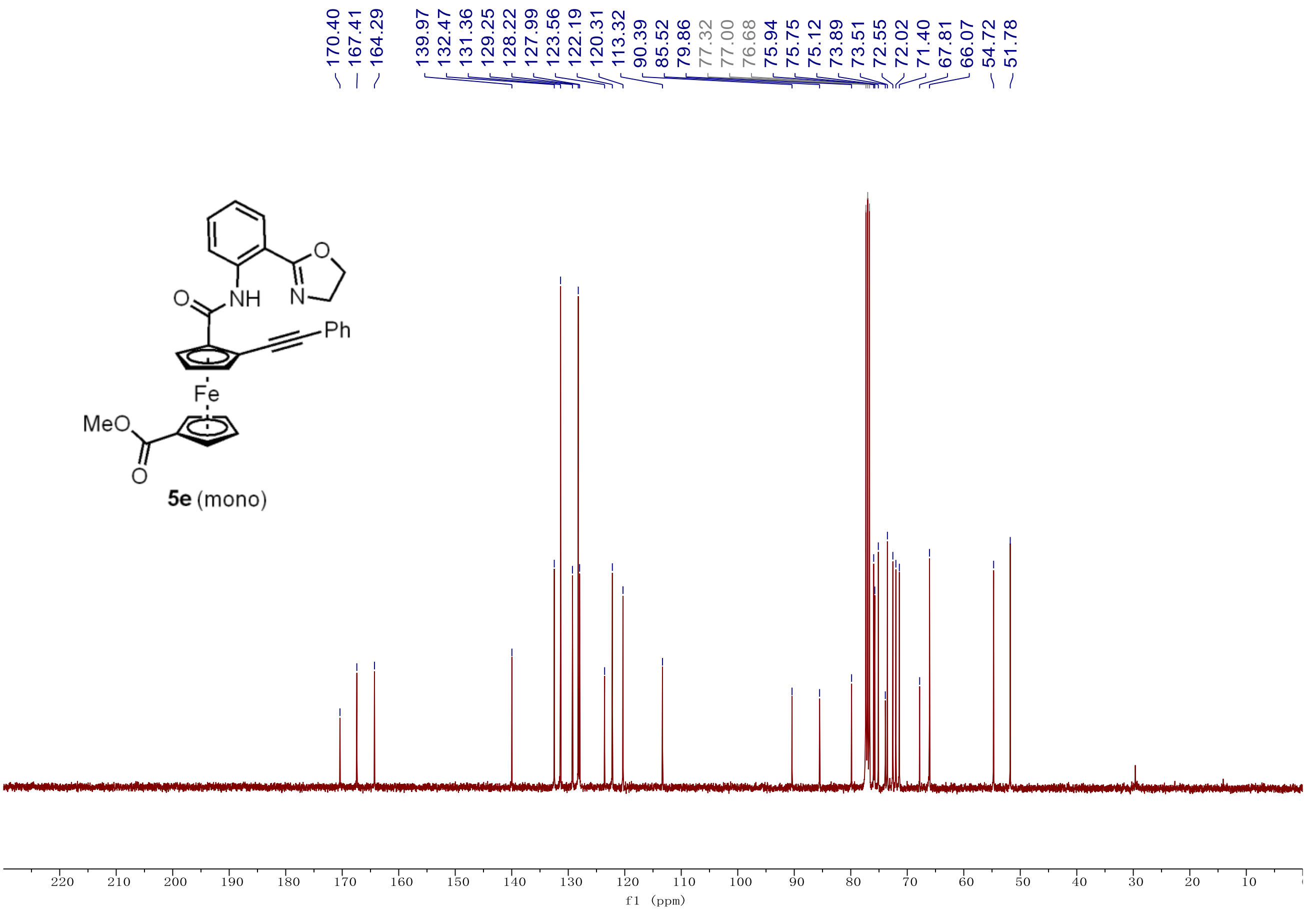
170.40
167.41
164.29
139.97
132.47
131.36
129.25
128.22
127.99
123.56
122.19
120.31
113.32
90.39
85.52
79.86
77.32
77.00
76.68
75.94
75.75
75.12
73.89
73.51
72.55
72.02
71.40
67.81
66.07
54.72
51.78
O
NH
O
N
Ph
Fe
MeO
O
5e (mono)
f1 (ppm)

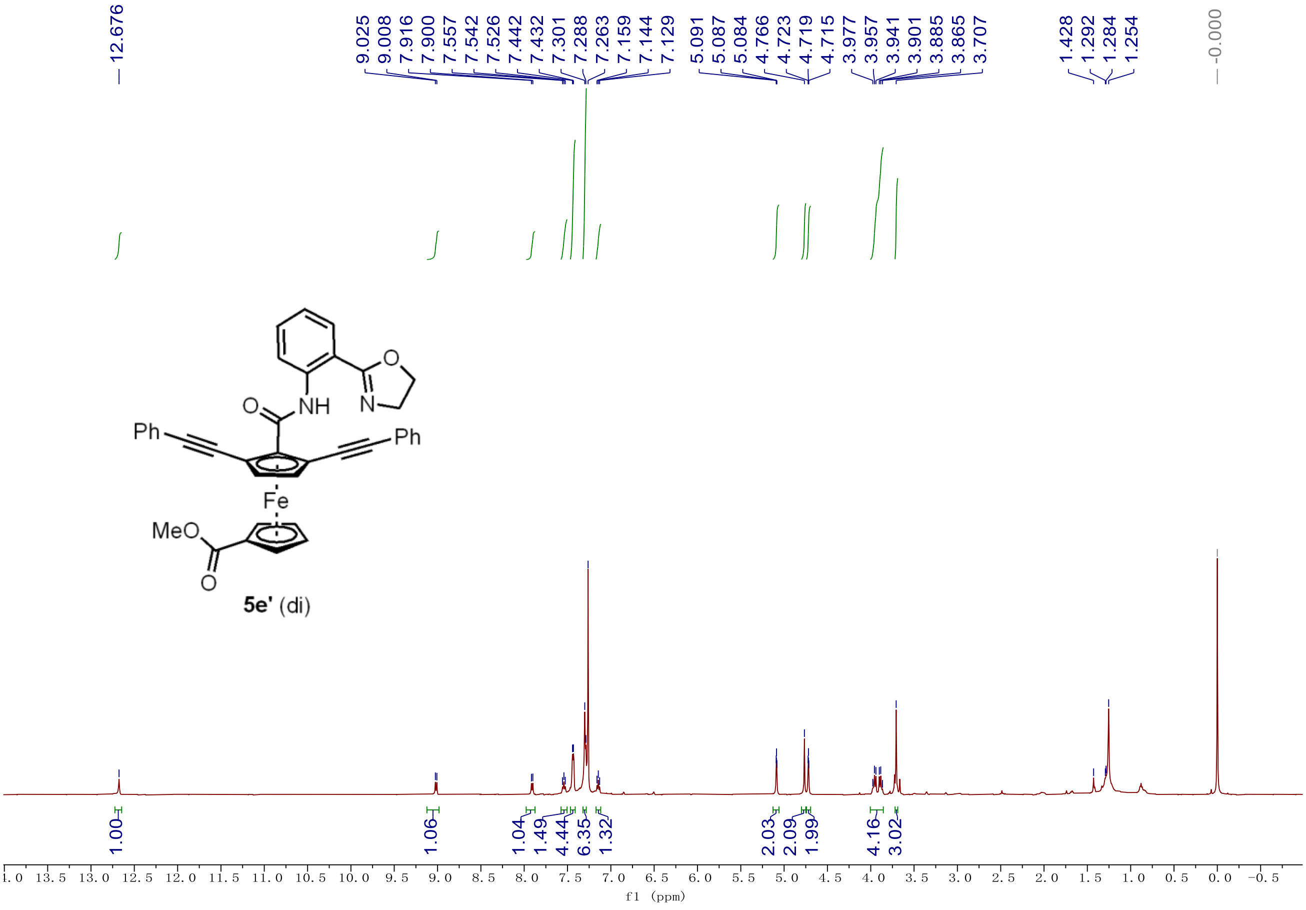
12.676
9.025
9.008
7.916
7.900
7.557
7.542
7.526
7.442
7.432
7.301
7.288
7.263
7.159
7.144
7.129
5.091
5.087
5.084
4.766
4.723
4.719
4.715
3.977
3.957
3.941
3.901
3.885
3.865
3.707
1.428
1.292
1.284
1.254
-0.000
O
NH
O
N
Ph
Ph
Fe
MeO
O
5e' (di)
1.00
1.06
1.04
1.49
4.44
6.35
1.32
2.03
2.09
1.99
4.16
3.02
f1 (ppm)

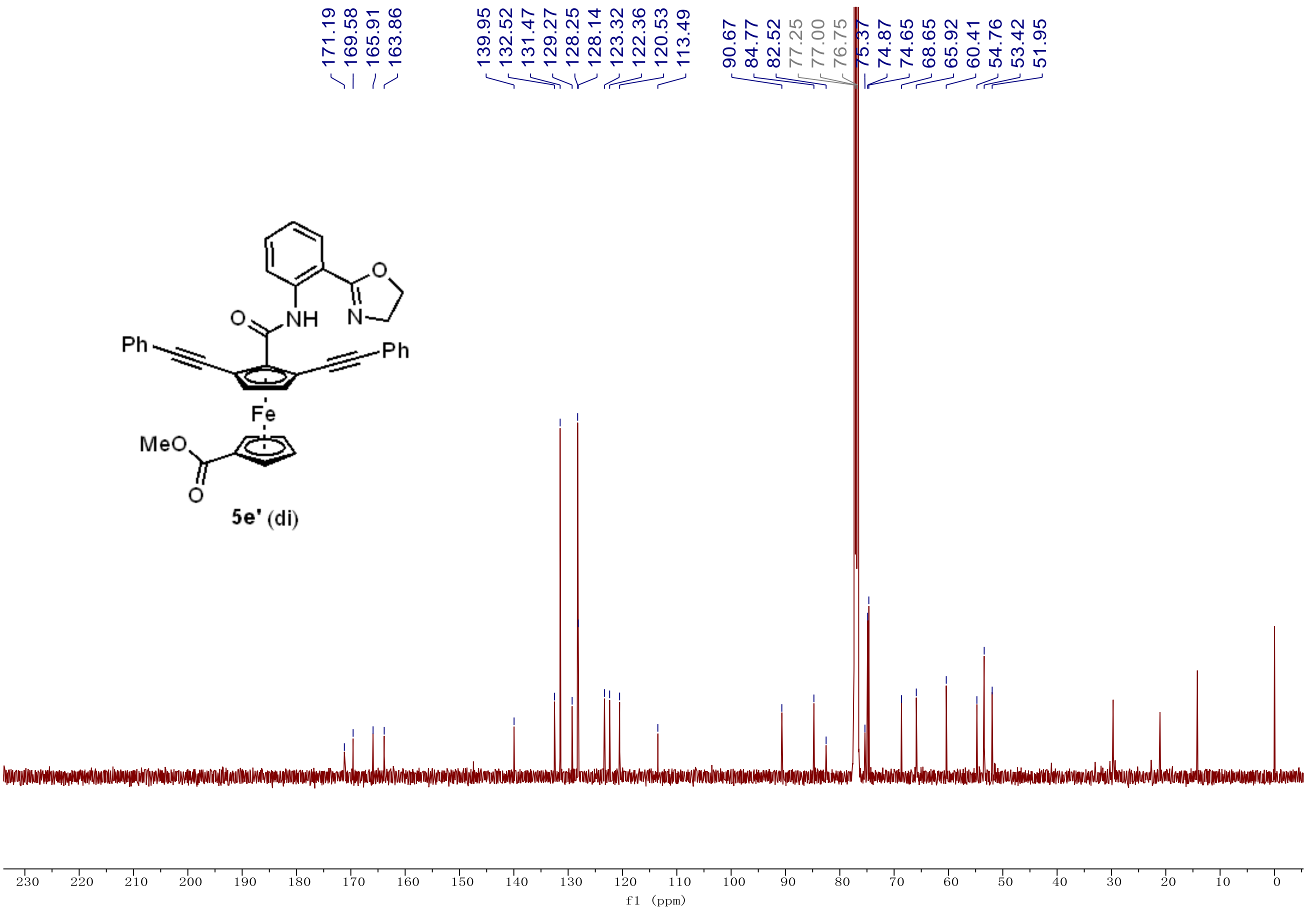
171.19
169.58
165.91
163.86
139.95
132.52
131.47
129.27
128.25
128.14
123.32
122.36
120.53
113.49
90.67
84.77
82.52
77.25
77.00
76.75
75.37
74.87
74.65
68.65
65.92
60.41
54.76
53.42
51.95
Ph
Ph
O
NH
O
N
Fe
MeO
O
5e' (di)
230 220 210 200 190 180 170 160 150 140 130 120 110 100 90 80 70 60 50 40 30 20 10 0
f1 (ppm)

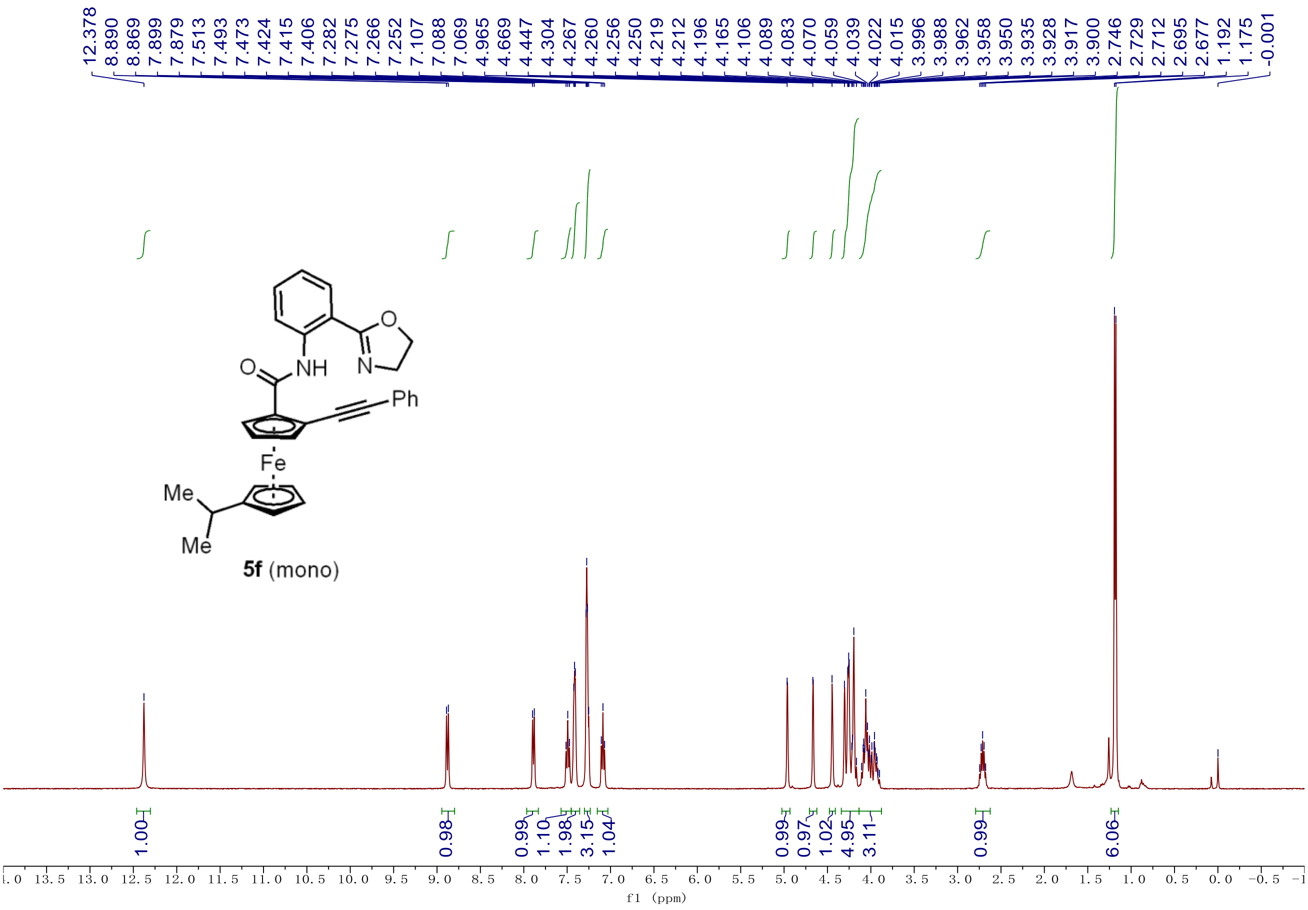
12.378
8.890
8.869
7.899
7.879
7.513
7.493
7.473
7.424
7.415
7.406
7.282
7.275
7.266
7.252
7.107
7.088
7.069
4.965
4.669
4.447
4.304
4.267
4.260
4.256
4.250
4.219
4.212
4.196
4.165
4.106
4.089
4.083
4.070
4.059
4.039
4.022
4.015
3.996
3.988
3.962
3.958
3.950
3.935
3.928
3.917
3.900
2.746
2.729
2.712
2.695
2.677
1.192
1.175
-0.001
O
NH
O
N
Ph
Fe
Me
Me
5f (mono)
1.00
0.98
0.99
1.10
1.98
3.15
1.04
0.99
0.97
1.02
4.95
3.11
0.99
6.06
f1 (ppm)

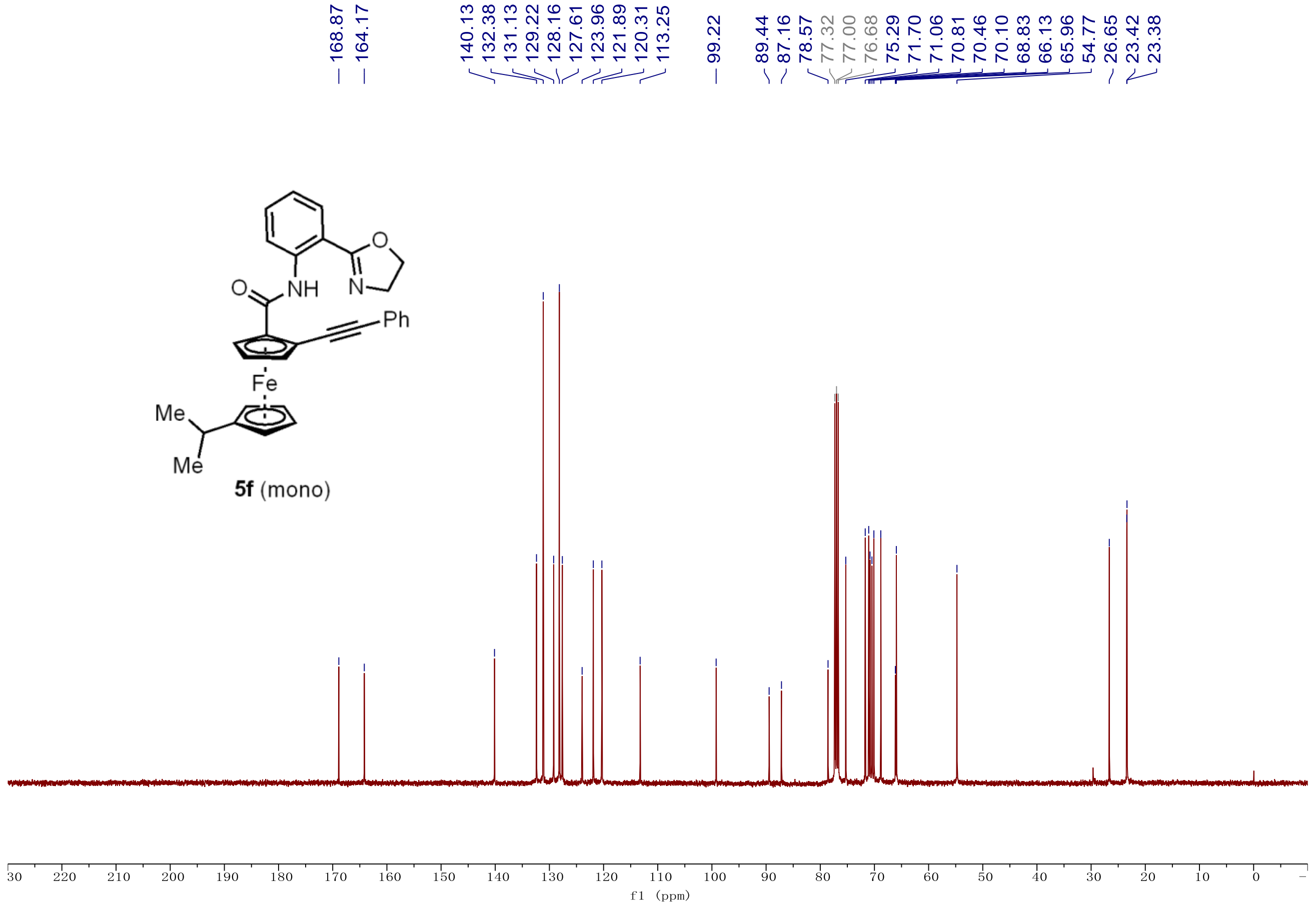
168.87
164.17
140.13
132.38
131.13
129.22
128.16
127.61
123.96
121.89
120.31
113.25
99.22
89.44
87.16
78.57
77.32
77.00
76.68
75.29
71.70
71.06
70.81
70.46
70.10
68.83
66.13
65.96
54.77
26.65
23.42
23.38
O
NH
N
Ph
Fe
Me
Me
5f (mono)
f1 (ppm)

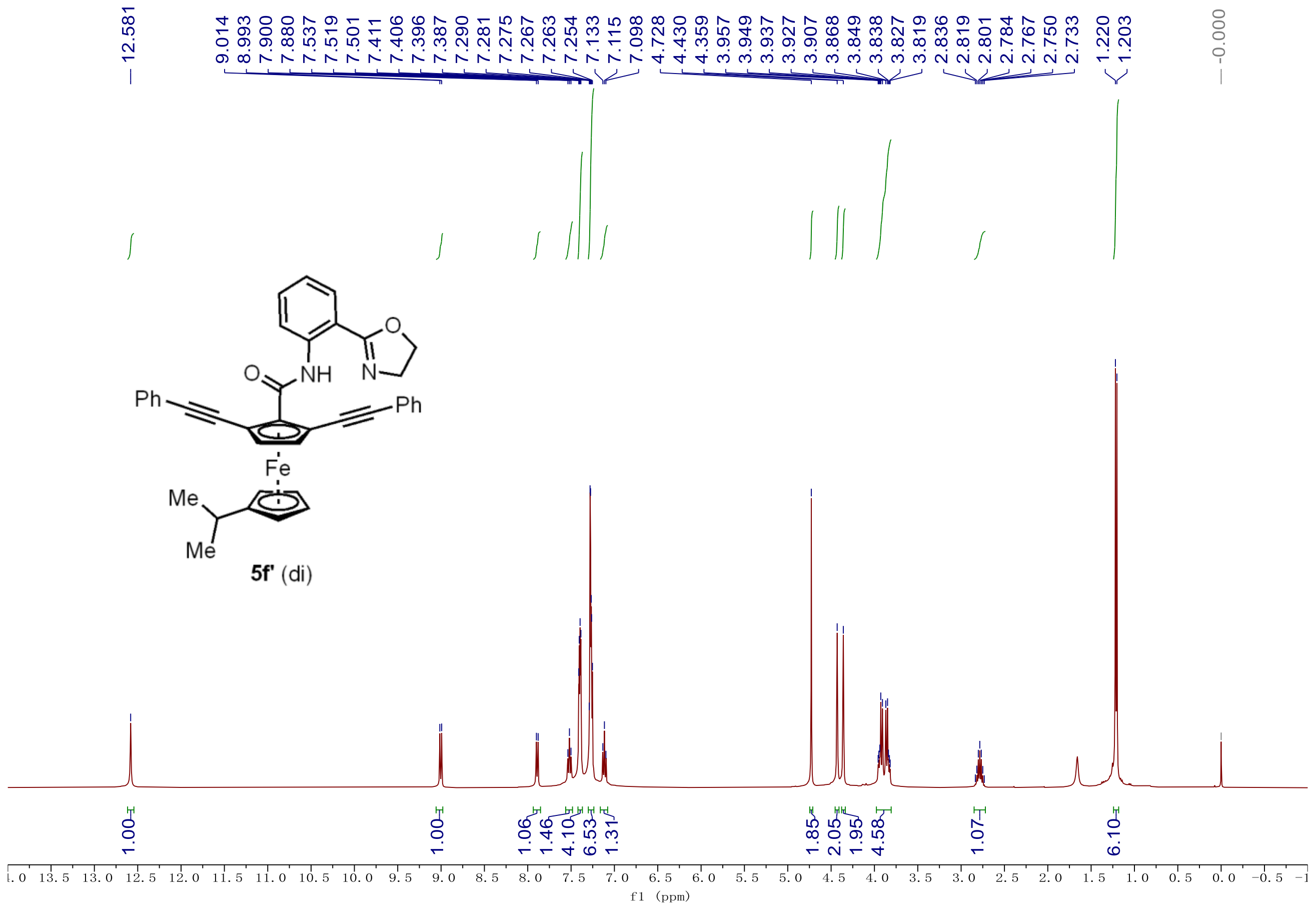
12.581
9.014
8.993
7.900
7.880
7.537
7.519
7.501
7.411
7.406
7.396
7.387
7.290
7.281
7.275
7.267
7.263
7.254
7.133
7.115
7.098
4.728
4.430
4.359
3.957
3.949
3.937
3.927
3.907
3.868
3.849
3.838
3.827
3.819
2.836
2.819
2.801
2.784
2.767
2.750
2.733
1.220
1.203
-0.000
Ph
Ph
O
NH
N
Fe
Me
Me
5f' (di)
1.00
1.00
1.06
1.46
4.10
6.53
1.31
1.85
2.05
1.95
4.58
1.07
6.10
f1 (ppm)

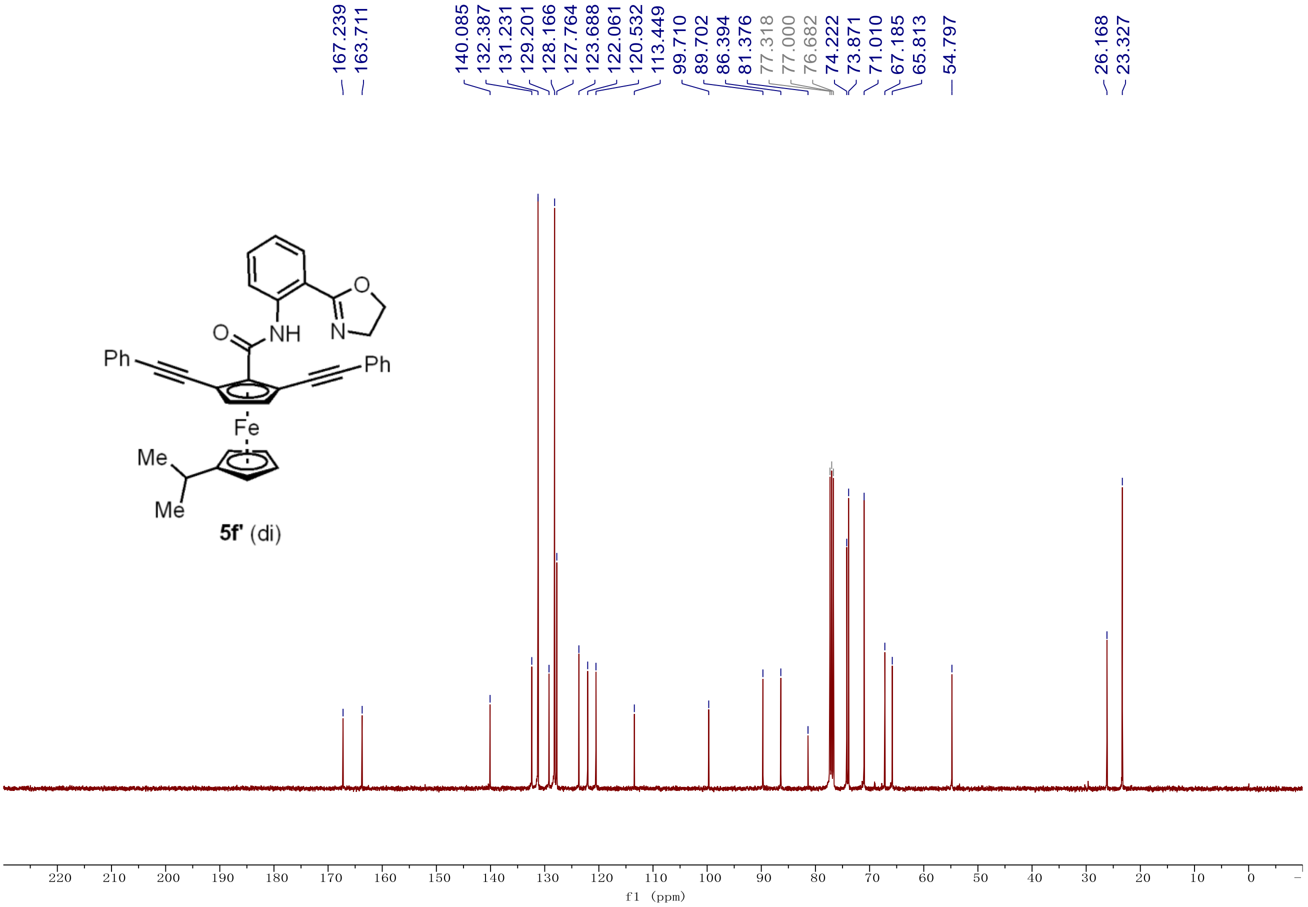

167.239
163.711
140.085
132.387
131.231
129.201
128.166
127.764
123.688
122.061
120.532
113.449
99.710
89.702
86.394
81.376
77.318
77.000
76.682
74.222
73.871
71.010
67.185
65.813
54.797
26.168
23.327
Ph
O
NH
O
N
Ph
Fe
Me
Me
5f' (di)
f1 (ppm)

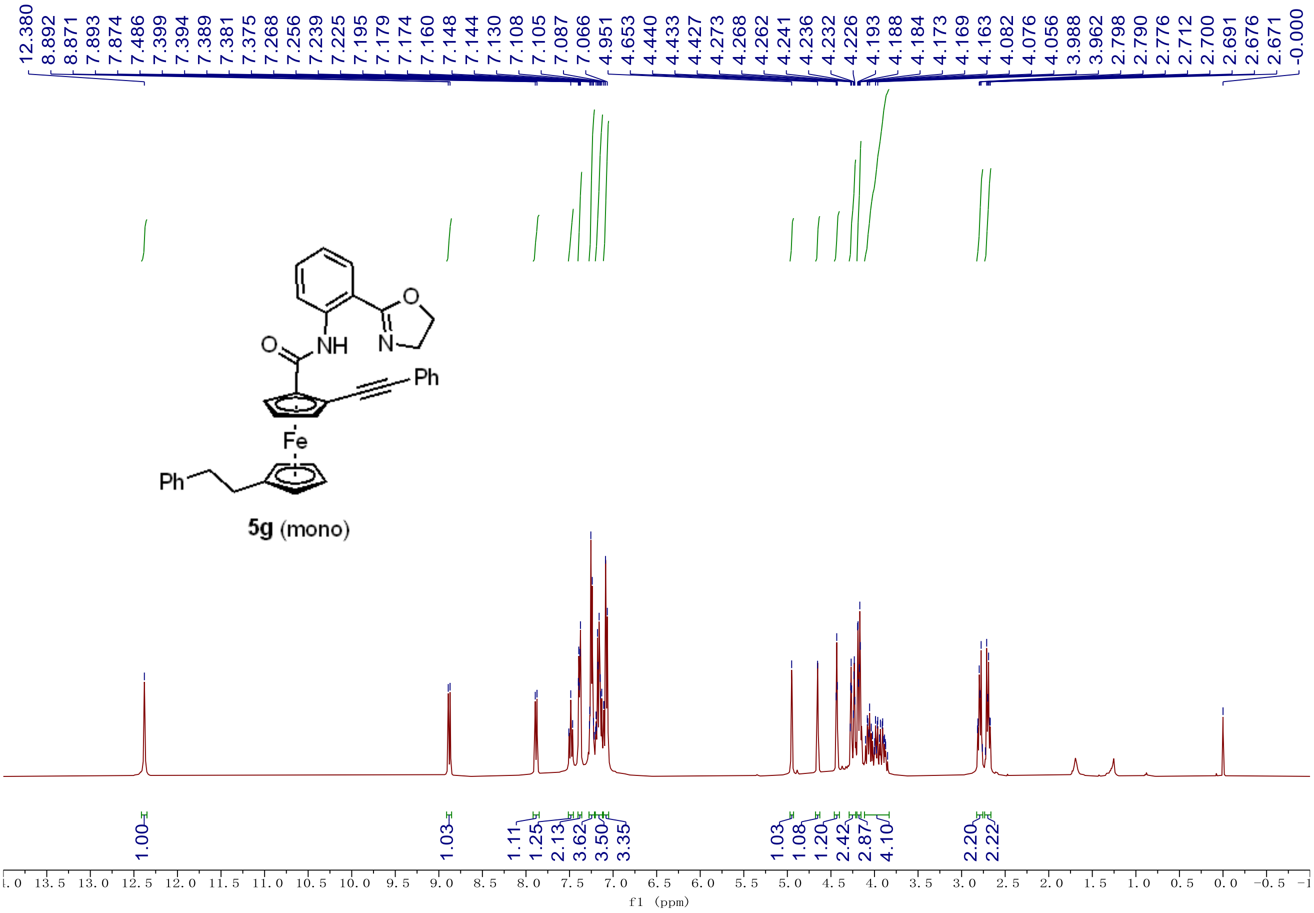

12.380
8.892
8.871
7.893
7.874
7.486
7.399
7.394
7.389
7.381
7.375
7.268
7.256
7.239
7.225
7.195
7.179
7.174
7.160
7.148
7.144
7.130
7.108
7.105
7.087
7.066
4.951
4.653
4.440
4.433
4.427
4.273
4.268
4.262
4.241
4.236
4.232
4.226
4.193
4.188
4.184
4.173
4.169
4.163
4.082
4.076
4.056
3.988
3.962
2.798
2.790
2.776
2.712
2.700
2.691
2.676
2.671
-0.000
O
NH
O
N
Ph
Fe
Ph
5g (mono)
1.00
1.03
1.11
1.25
2.13
3.62
3.50
3.35
1.03
1.08
1.20
2.42
2.87
4.10
2.20
2.22
f1 (ppm)

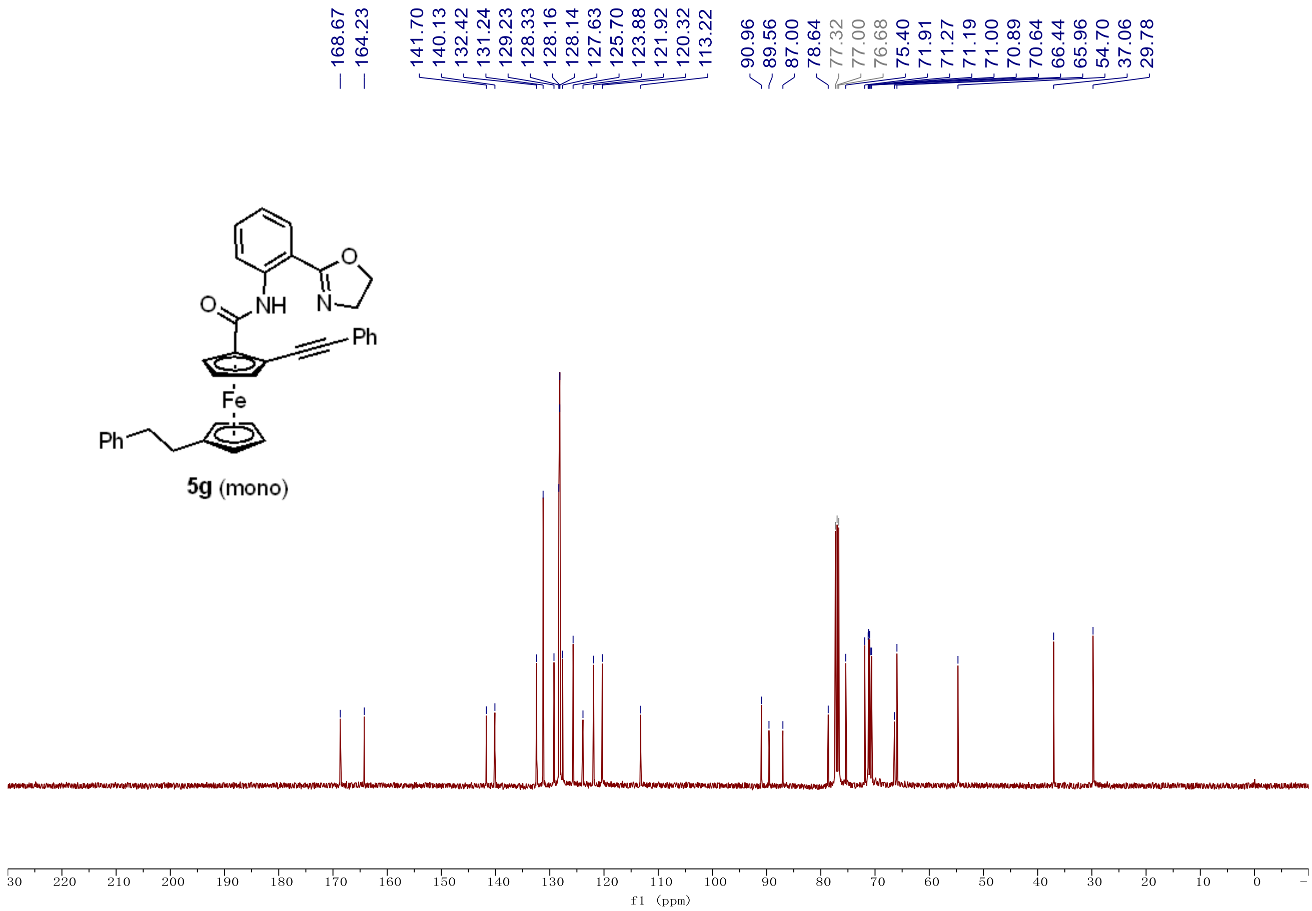
168.67
164.23
141.70
140.13
132.42
131.24
129.23
128.33
128.16
128.14
127.63
125.70
123.88
121.92
120.32
113.22
90.96
89.56
87.00
78.64
77.32
77.00
76.68
75.40
71.91
71.27
71.19
71.00
70.89
70.64
66.44
65.96
54.70
37.06
29.78
O
NH
O
N
Ph
Fe
Ph
5g (mono)
f1 (ppm)

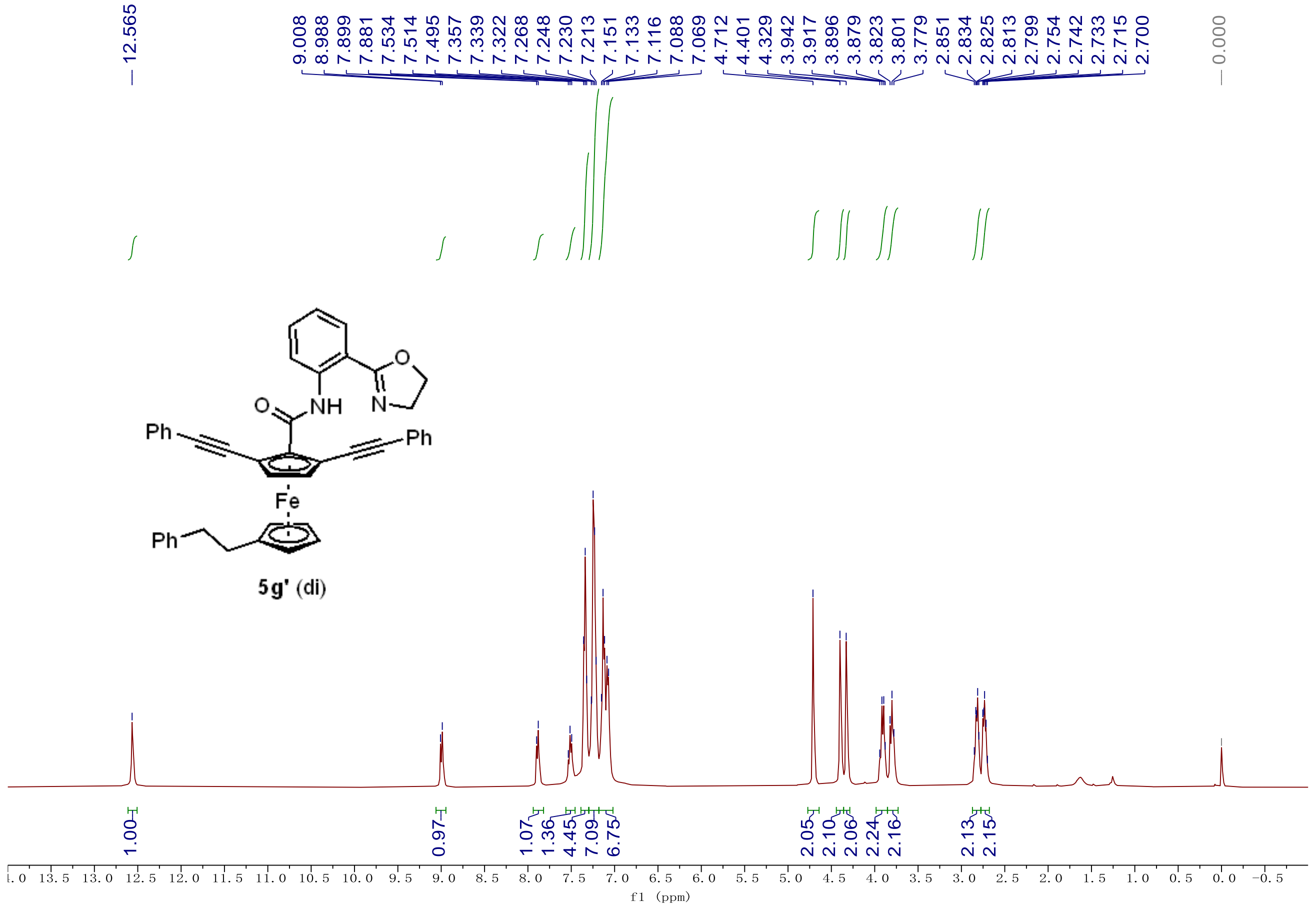
12.565
9.008
8.988
7.899
7.881
7.534
7.495
7.357
7.339
7.322
7.268
7.248
7.230
7.213
7.151
7.133
7.116
7.088
7.069
4.712
4.401
4.329
3.942
3.917
3.896
3.879
3.823
3.801
3.779
2.851
2.834
2.825
2.813
2.799
2.754
2.742
2.733
2.715
2.700
0.000
O
NH
O
N
Ph
Ph
Fe
Ph
5g' (di)
1.00
0.97
1.07
1.36
4.45
7.09
6.75
2.05
2.10
2.06
2.24
2.16
2.13
2.15
f1 (ppm)

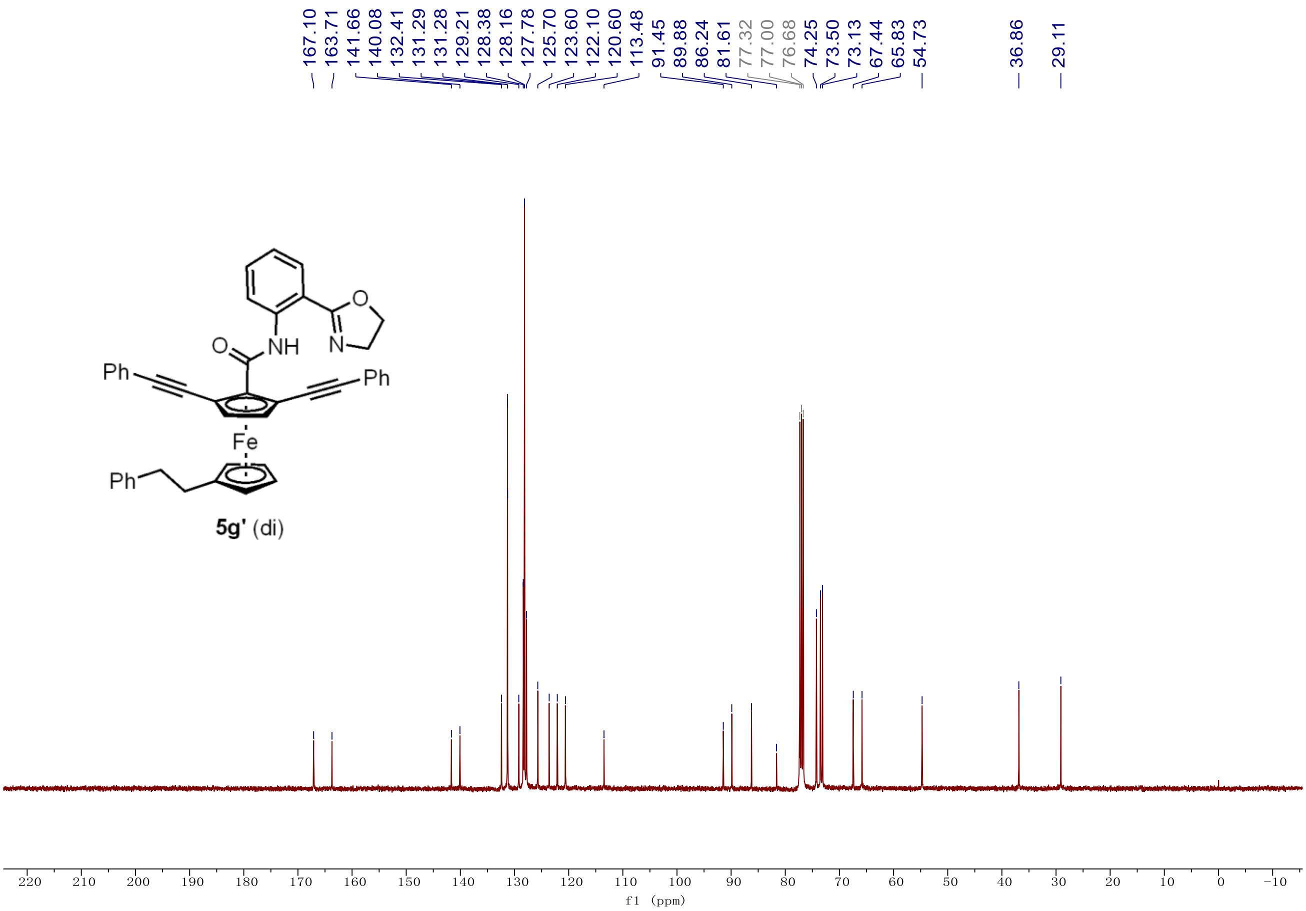
167.10
163.71
141.66
140.08
132.41
131.29
131.28
129.21
128.38
128.16
127.78
125.70
123.60
122.10
120.60
113.48
91.45
89.88
86.24
81.61
77.32
77.00
76.68
74.25
73.50
73.13
67.44
65.83
54.73
36.86
29.11
Ph
Ph
Fe
Ph
O
NH
N
O
5g' (di)
220 210 200 190 180 170 160 150 140 130 120 110 100 90 80 70 60 50 40 30 20 10 0 -10
f1 (ppm)

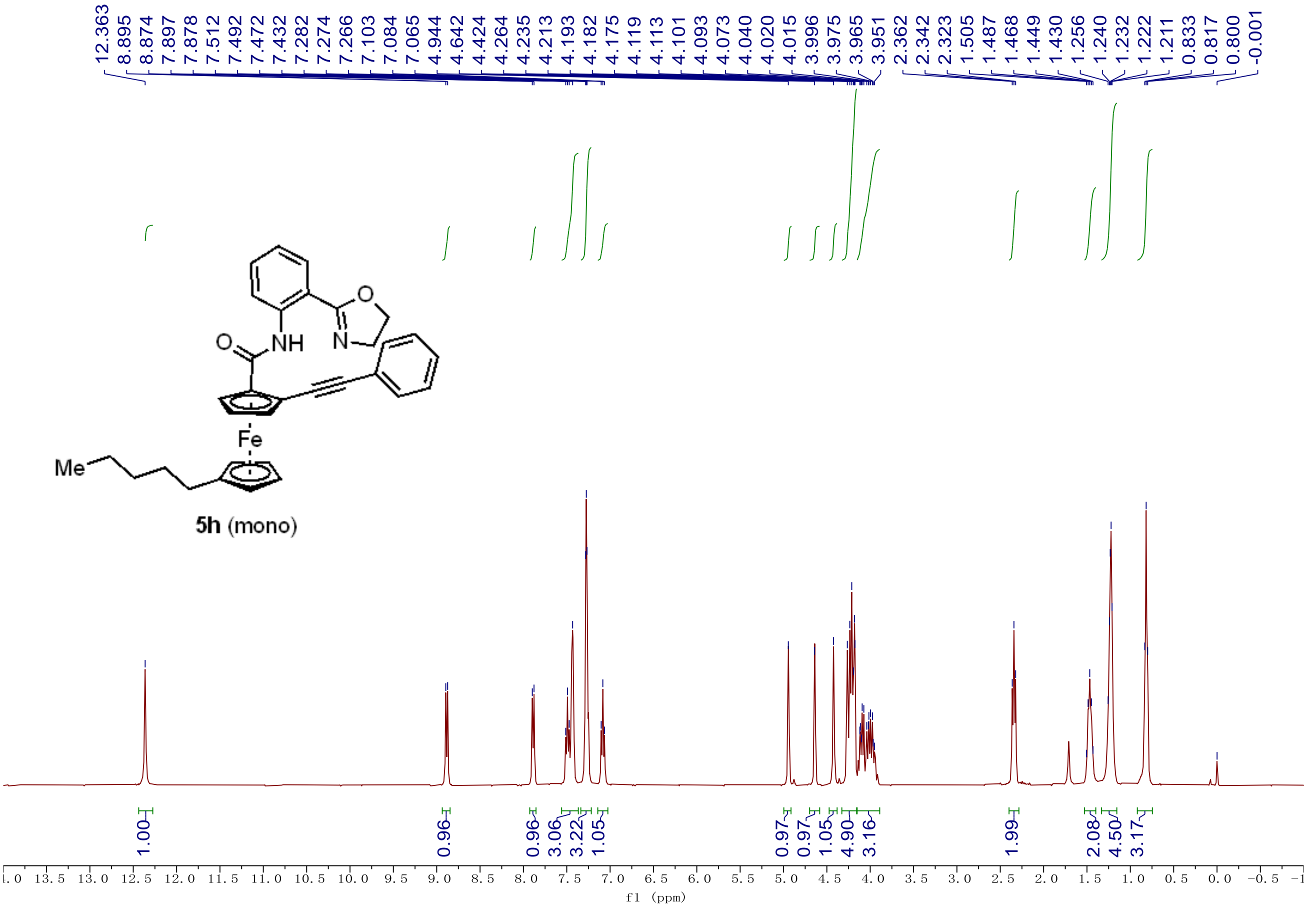
12.363
8.895
8.874
7.897
7.878
7.512
7.492
7.472
7.432
7.282
7.274
7.266
7.103
7.084
7.065
4.944
4.642
4.424
4.264
4.235
4.213
4.193
4.182
4.175
4.119
4.113
4.101
4.093
4.073
4.040
4.020
4.015
3.996
3.975
3.965
3.951
2.362
2.342
2.323
1.505
1.487
1.468
1.449
1.430
1.256
1.240
1.232
1.222
1.211
0.833
0.817
0.800
-0.001
O
NH
N
O
Fe
Me
5h (mono)
1.00
0.96
0.96
3.06
3.22
1.05
0.97
0.97
1.05
4.90
3.16
1.99
2.08
4.50
3.17
13.5 13.0 12.5 12.0 11.5 11.0 10.5 10.0 9.5 9.0 8.5 8.0 7.5 7.0 6.5 6.0 5.5 5.0 4.5 4.0 3.5 3.0 2.5 2.0 1.5 1.0 0.5 0.0 -0.5
f1 (ppm)

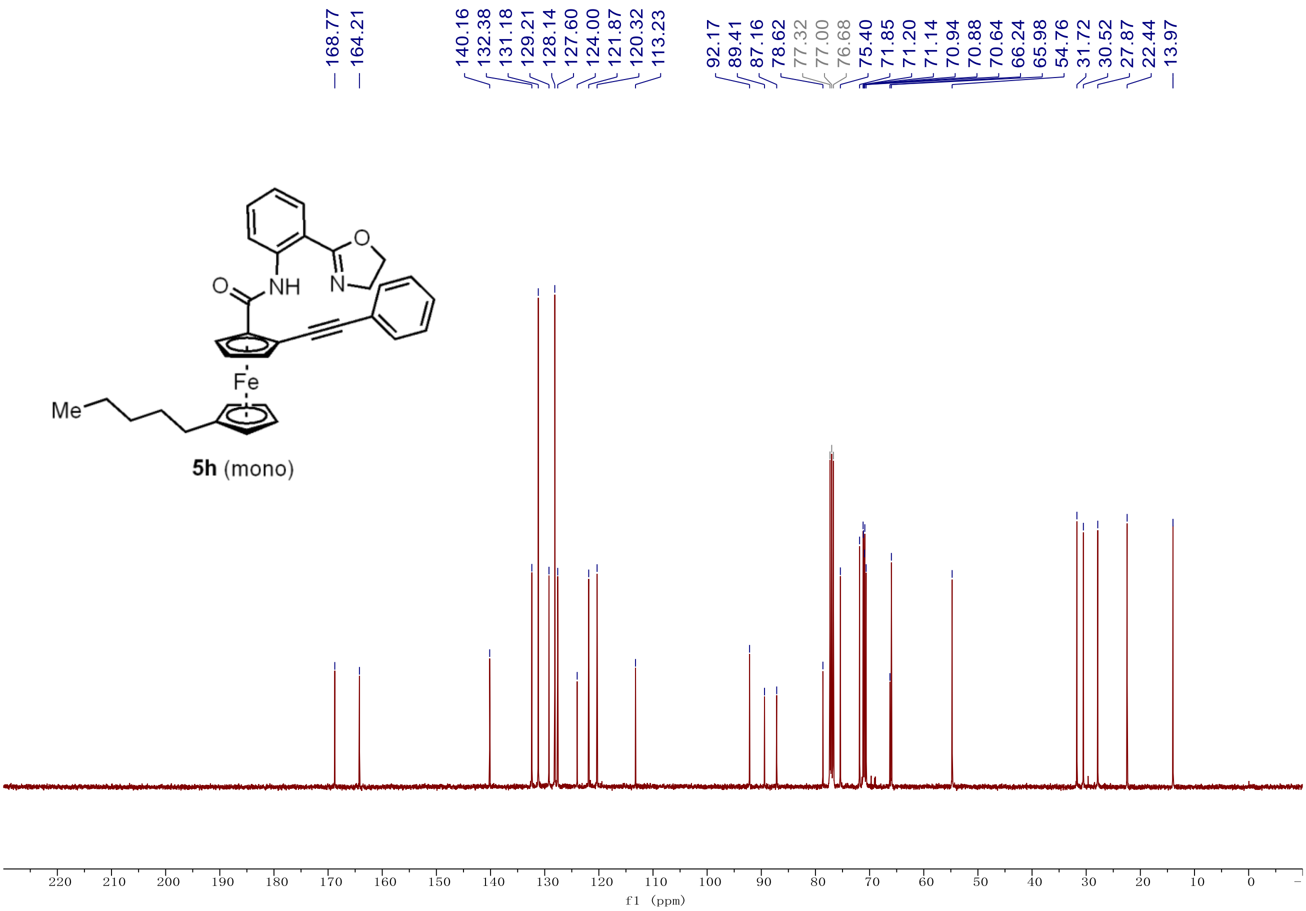
168.77
164.21
140.16
132.38
131.18
129.21
128.14
127.60
124.00
121.87
120.32
113.23
92.17
89.41
87.16
78.62
77.32
77.00
76.68
75.40
71.85
71.20
71.14
70.94
70.88
70.64
66.24
65.98
54.76
31.72
30.52
27.87
22.44
13.97
O
NH
N
Fe
Me
5h (mono)
f1 (ppm)

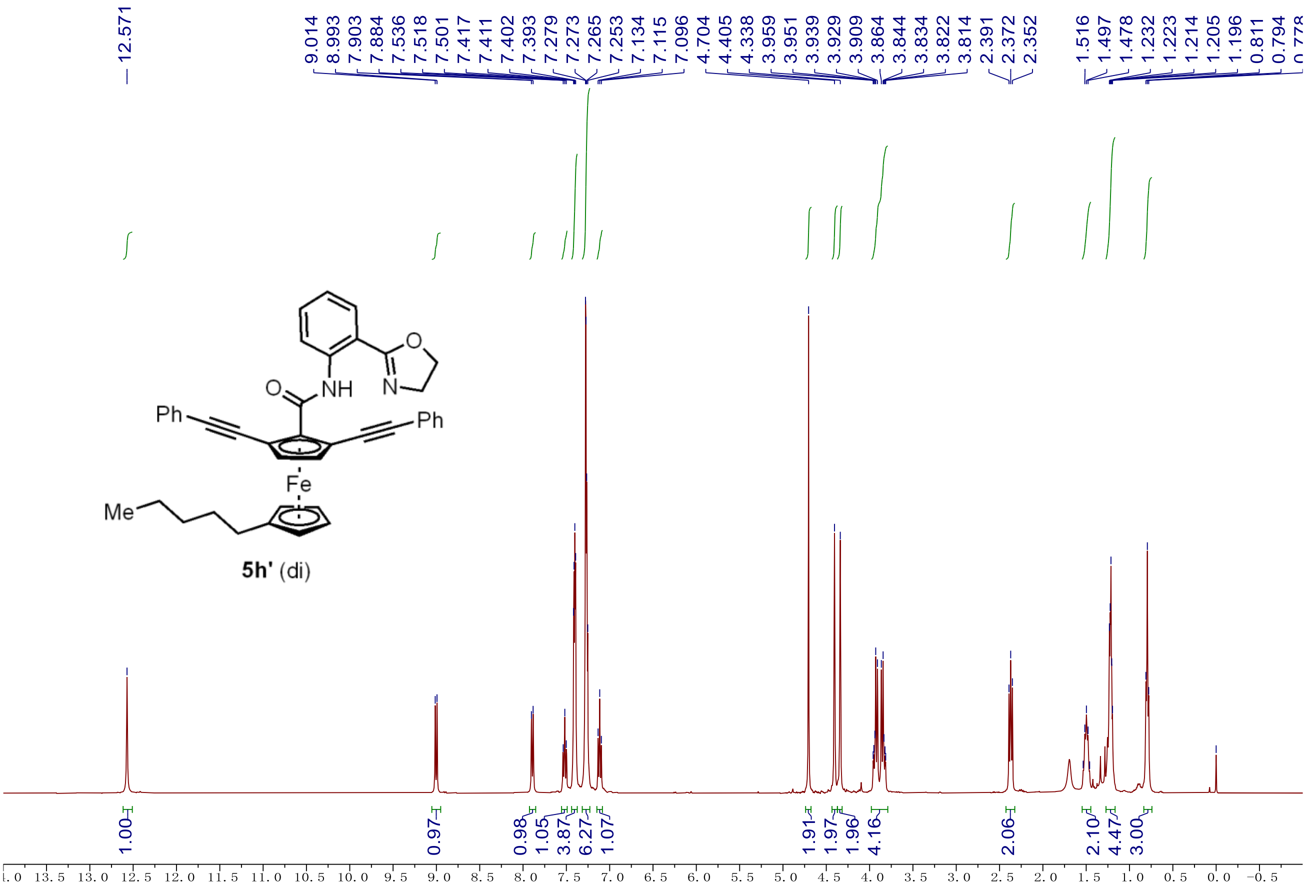
12.571
9.014
8.993
7.903
7.884
7.536
7.518
7.501
7.417
7.411
7.402
7.393
7.279
7.273
7.265
7.253
7.134
7.115
7.096
4.704
4.405
4.338
3.959
3.951
3.939
3.929
3.909
3.864
3.844
3.834
3.822
3.814
2.391
2.372
2.352
1.516
1.497
1.478
1.232
1.223
1.214
1.205
1.196
0.811
0.794
O
NH
N
Ph
Ph
Fe
Me
5h' (di)
1.00
0.97
0.98
1.05
3.87
6.27
1.07
1.91
1.97
1.96
4.16
2.06
2.10
4.47
3.00
f1 (ppm)

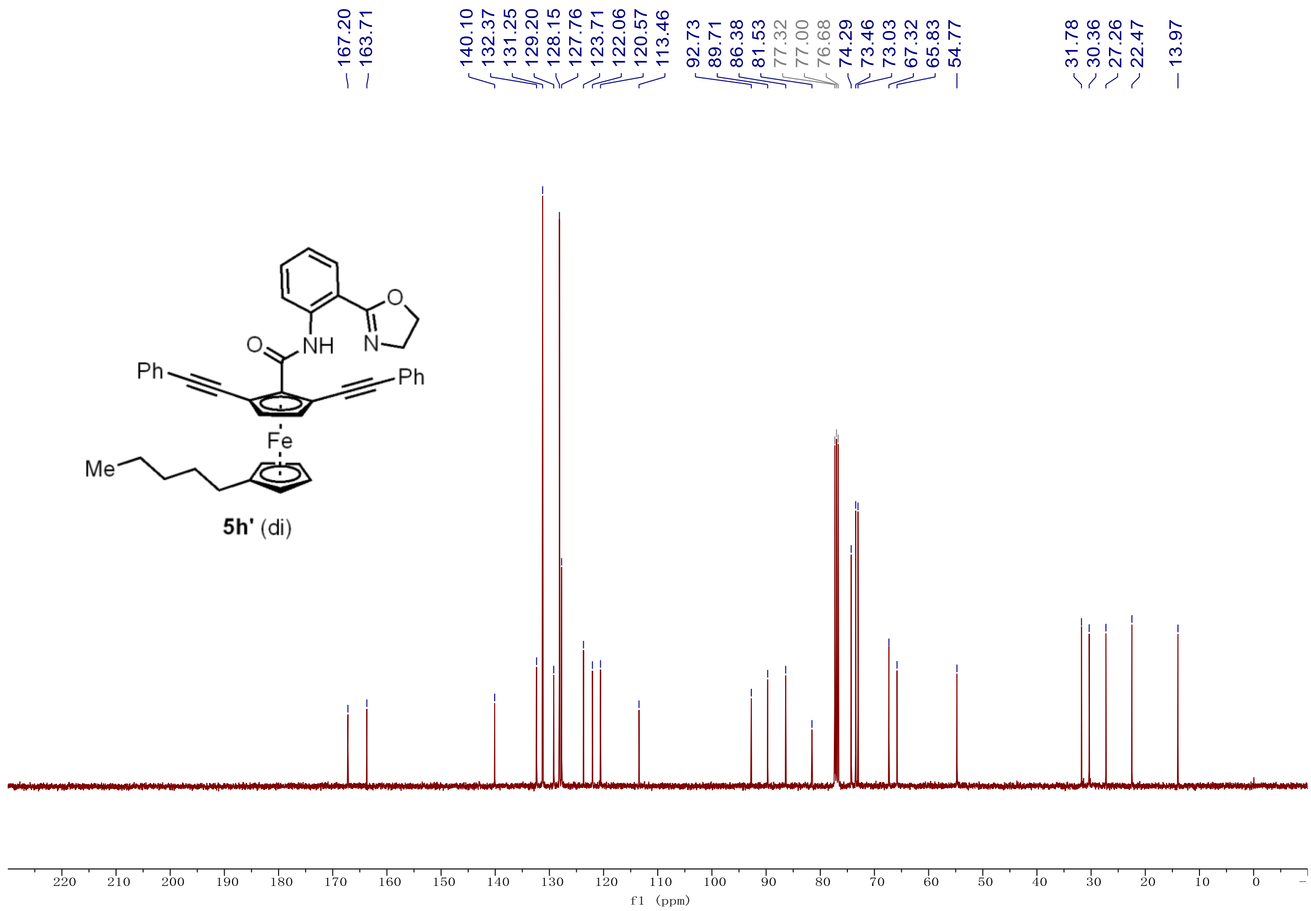
167.20
163.71
140.10
132.37
131.25
129.20
128.15
127.76
123.71
122.06
120.57
113.46
92.73
89.71
86.38
81.53
77.32
77.00
76.68
74.29
73.46
73.03
67.32
65.83
54.77
31.78
30.36
27.26
22.47
13.97
Ph
O
NH
N
O
Ph
Fe
Me
5h' (di)
220 210 200 190 180 170 160 150 140 130 120 110 100 90 80 70 60 50 40 30 20 10 0
f1 (ppm)

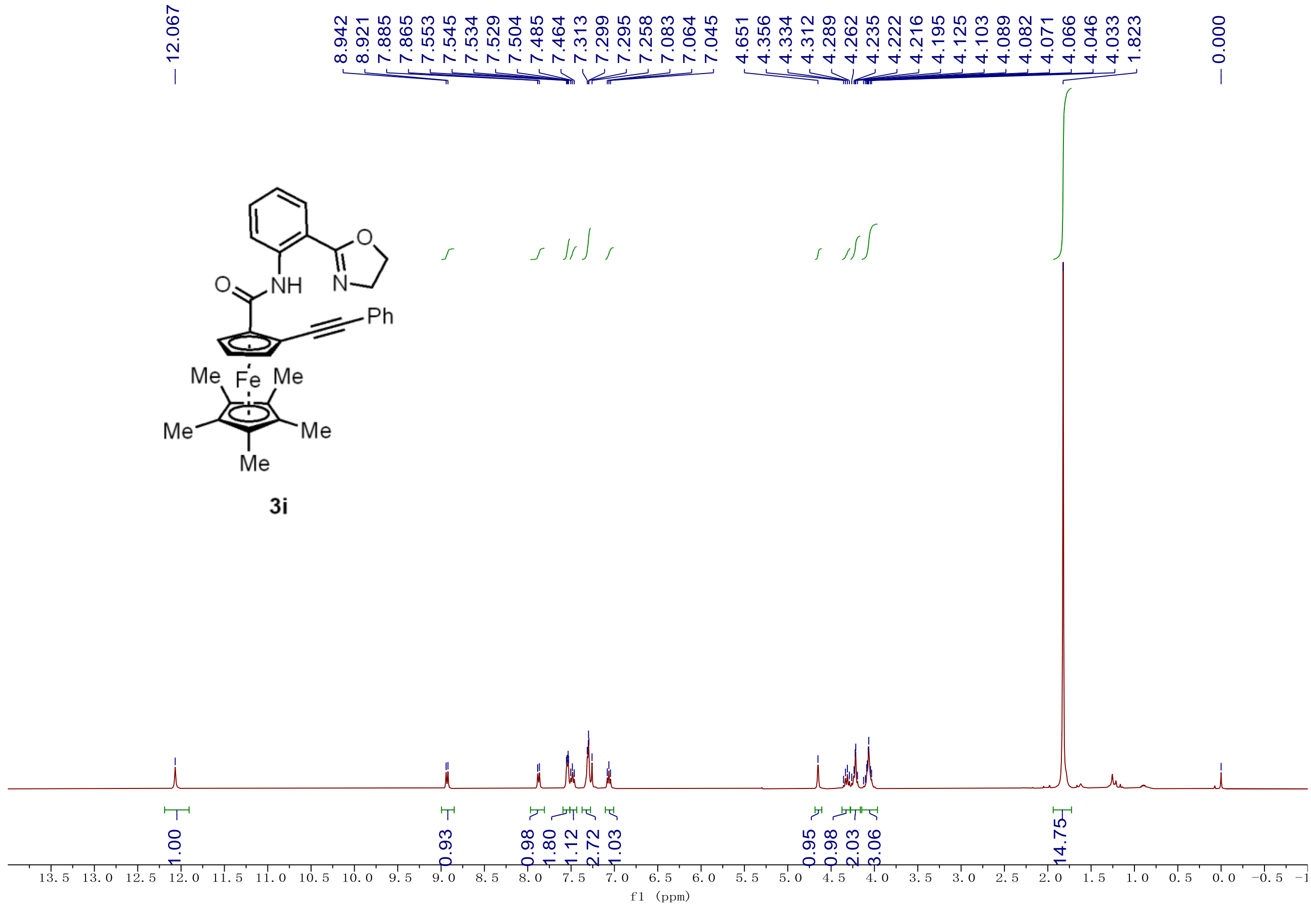
12.067
8.942
8.921
7.885
7.865
7.553
7.545
7.534
7.529
7.504
7.485
7.464
7.313
7.299
7.295
7.258
7.083
7.064
7.045
4.651
4.356
4.334
4.312
4.289
4.262
4.235
4.222
4.216
4.195
4.125
4.103
4.089
4.082
4.071
4.066
4.046
4.033
1.823
0.000
O
NH
N
O
Ph
Me
Fe
Me
Me
Me
Me
3i
1.00
0.93
0.98
1.80
1.12
2.72
1.03
0.95
0.98
2.03
3.06
14.75
13.5 13.0 12.5 12.0 11.5 11.0 10.5 10.0 9.5 9.0 8.5 8.0 7.5 7.0 6.5 6.0 5.5 5.0 4.5 4.0 3.5 3.0 2.5 2.0 1.5 1.0 0.5 0.0 -0.5 -1
f1 (ppm)

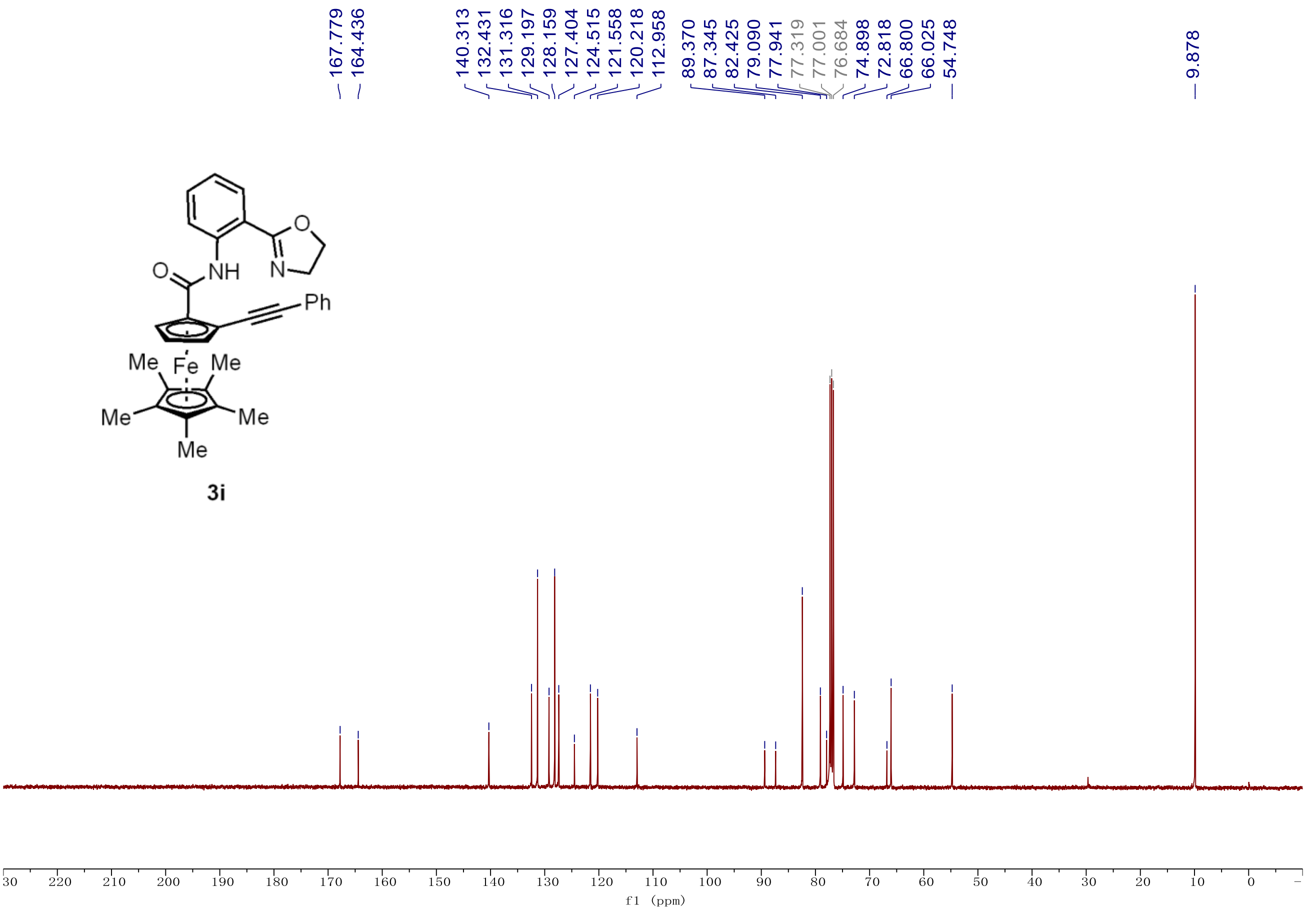

167.779
164.436
140.313
132.431
131.316
129.197
128.159
127.404
124.515
121.558
120.218
112.958
89.370
87.345
82.425
79.090
77.941
77.319
77.001
76.684
74.898
72.818
66.800
66.025
54.748
9.878
O
NH
O
N
Ph
Fe
Me
Me
Me
Me
Me
f1 (ppm)

3i

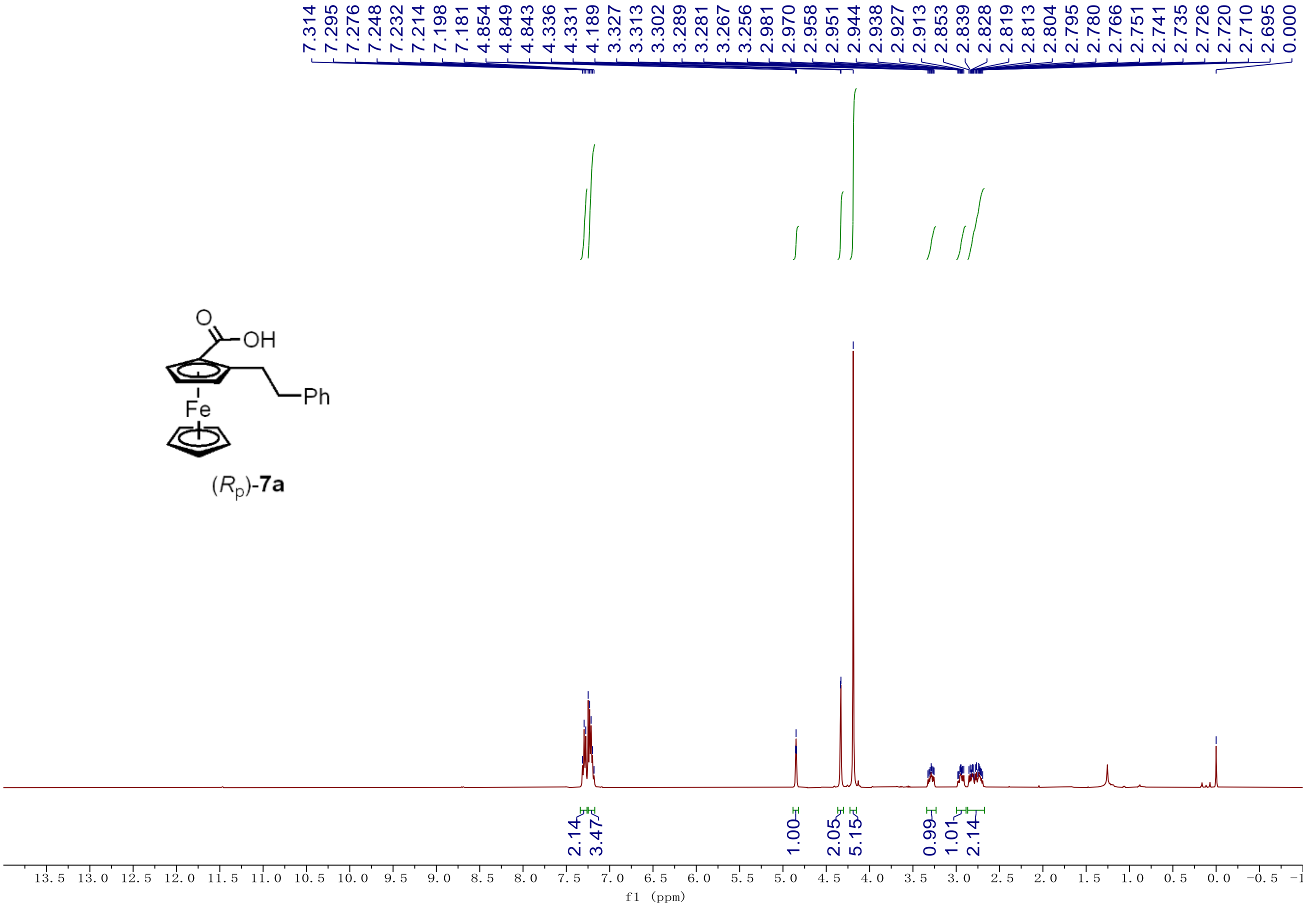

7.314
7.295
7.276
7.248
7.232
7.214
7.198
7.181
4.854
4.849
4.843
4.336
4.331
4.189
3.327
3.313
3.302
3.289
3.281
3.267
3.256
2.981
2.970
2.958
2.951
2.944
2.938
2.927
2.913
2.853
2.839
2.828
2.819
2.813
2.804
2.795
2.780
2.766
2.751
2.741
2.735
2.726
2.720
2.710
2.695
0.000
O
OH
Ph
Fe
2.14
3.47
1.00
2.05
5.15
0.99
1.01
2.14
f1 (ppm)

(R_p)-**7a**

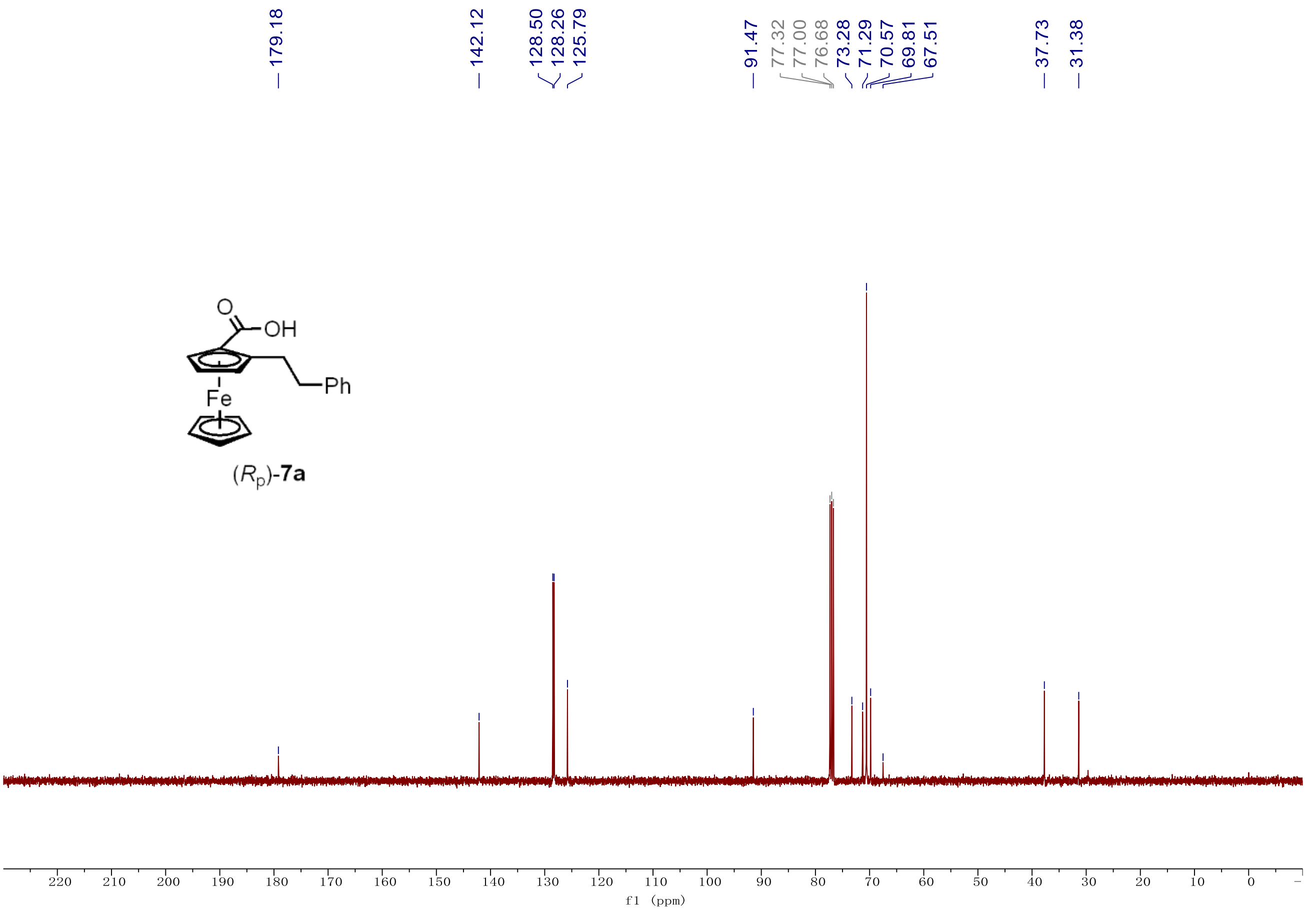
179.18
142.12
128.50
128.26
125.79
91.47
77.32
77.00
76.68
73.28
71.29
70.57
69.81
67.51
37.73
31.38
OH
Fe
Ph
(Rp)-7a
f1 (ppm)

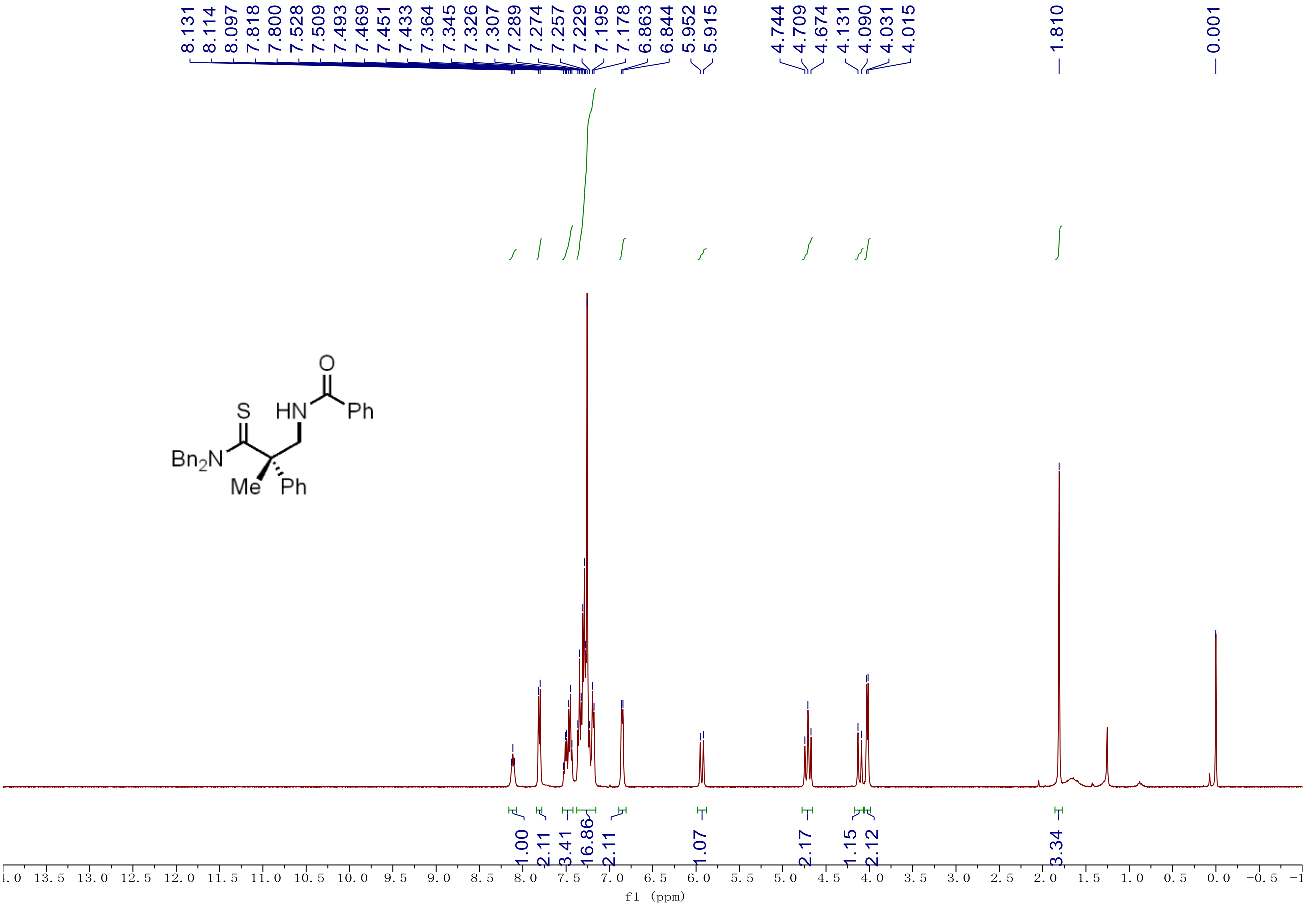
8.131
8.114
8.097
7.818
7.800
7.528
7.509
7.493
7.469
7.451
7.433
7.364
7.345
7.326
7.307
7.289
7.274
7.257
7.229
7.195
7.178
6.863
6.844
5.952
5.915
4.744
4.709
4.674
4.131
4.090
4.031
4.015
1.810
0.001
S
HN
O
Ph
Bn2N
Me
Ph
1.00
2.11
3.41
16.86
2.11
1.07
2.17
1.15
2.12
3.34
f1 (ppm)

5. Chiral HPLC Chromatograms

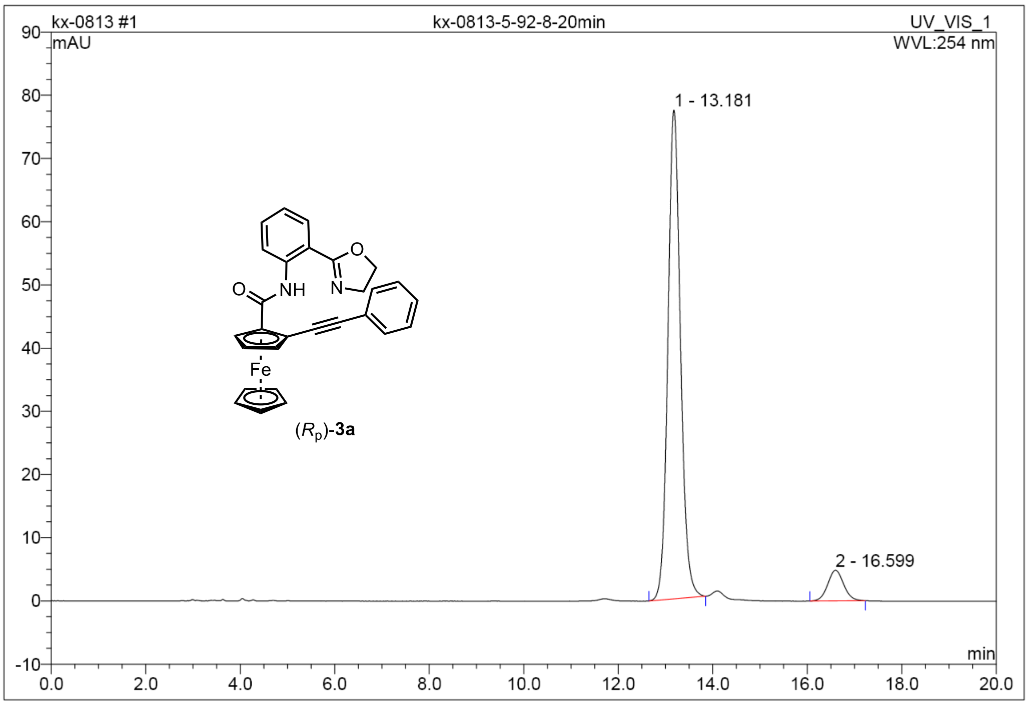

No.	Ret.Time min	Peak Name	Height mAU	Area mAU*min	Rel.Area %	Amount	Type
1	13.18	n.a.	77.329	23.808	92.61	n.a.	BMB
2	16.60	n.a.	4.849	1.901	7.39	n.a.	BMB
Total:			82.178	25.708	100.00	0.000	

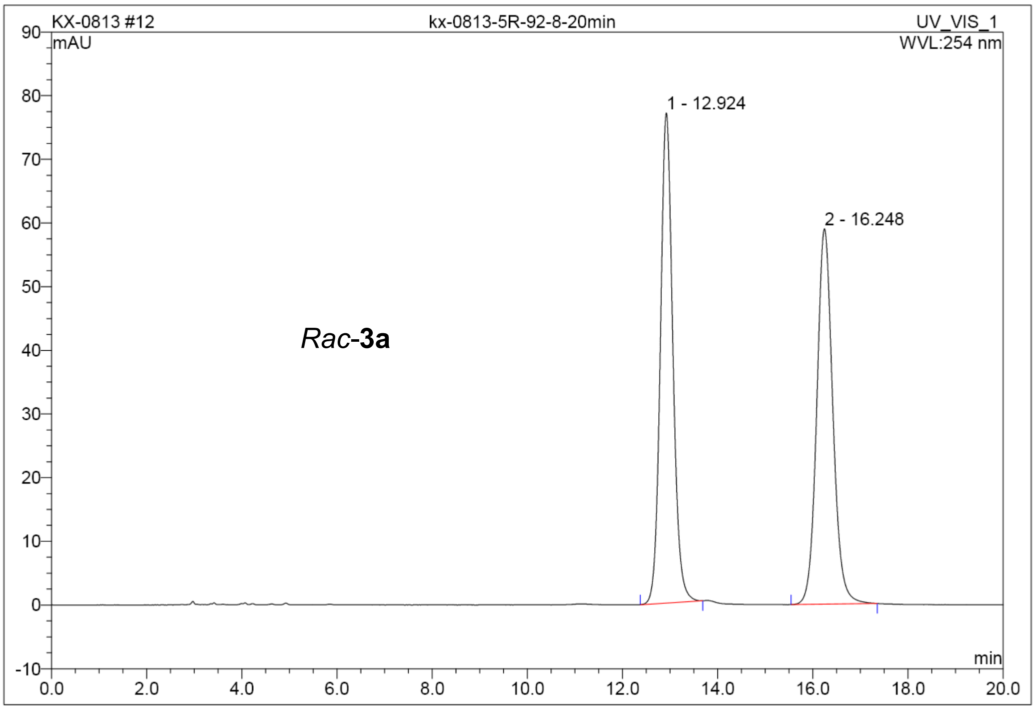

No.	Ret.Time min	Peak Name	Height mAU	Area mAU*min	Rel.Area %	Amount	Type
1	12.92	n.a.	76.975	23.356	50.37	n.a.	BMB
2	16.25	n.a.	58.964	23.014	49.63	n.a.	BMB
Total:			135.939	46.369	100.00	0.000	

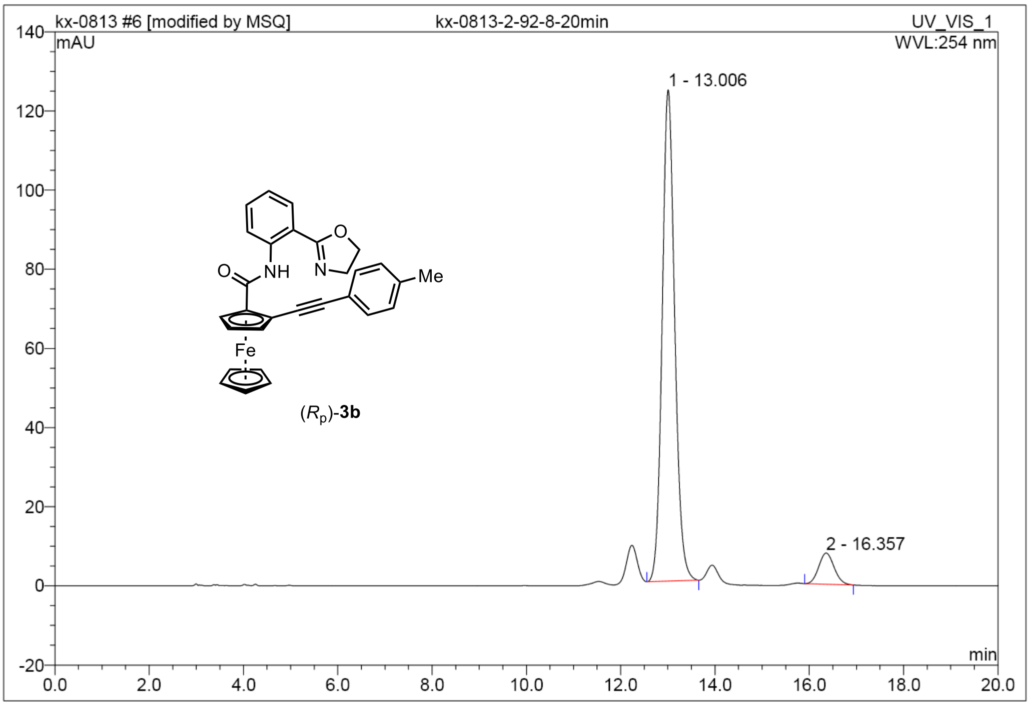

No.	Ret.Time min	Peak Name	Height mAU	Area mAU*min	Rel.Area %	Amount	Type
1	13.01	n.a.	124.167	38.176	92.75	n.a.	BMB*
2	16.36	n.a.	7.877	2.984	7.25	n.a.	BMB
**Total:**			132.044	41.161	100.00	0.000	

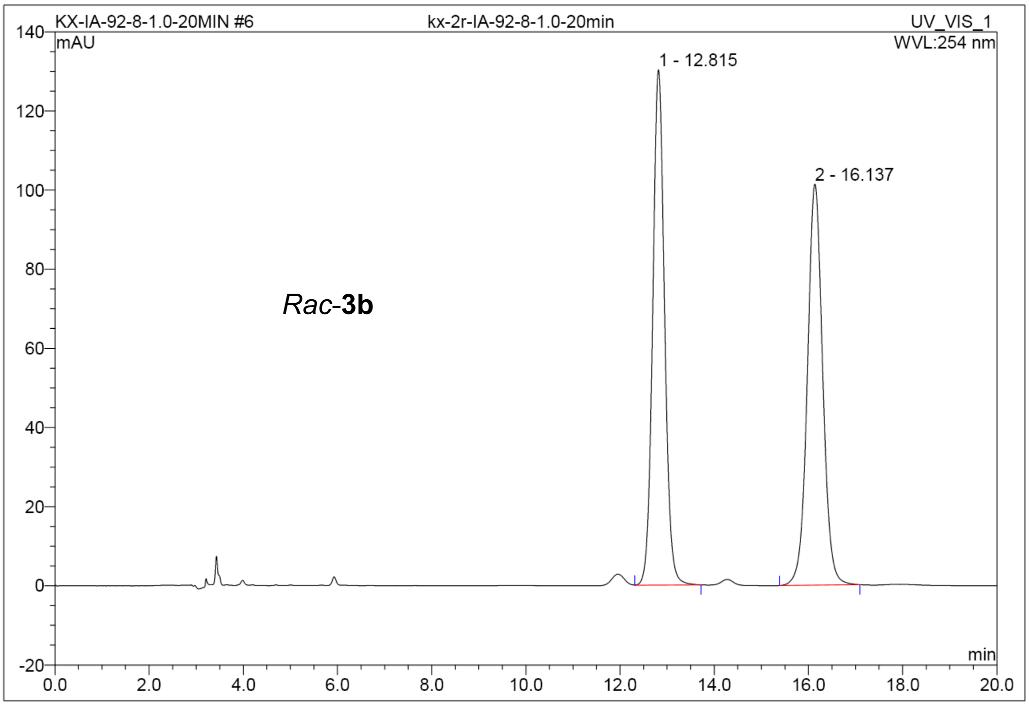

No.	Ret.Time min	Peak Name	Height mAU	Area mAU*min	Rel.Area %	Amount	Type
1	12.82	n.a.	130.192	37.882	49.77	n.a.	BMB
2	16.14	n.a.	101.330	38.235	50.23	n.a.	BMB
**Total:**			231.522	76.117	100.00	0.000	

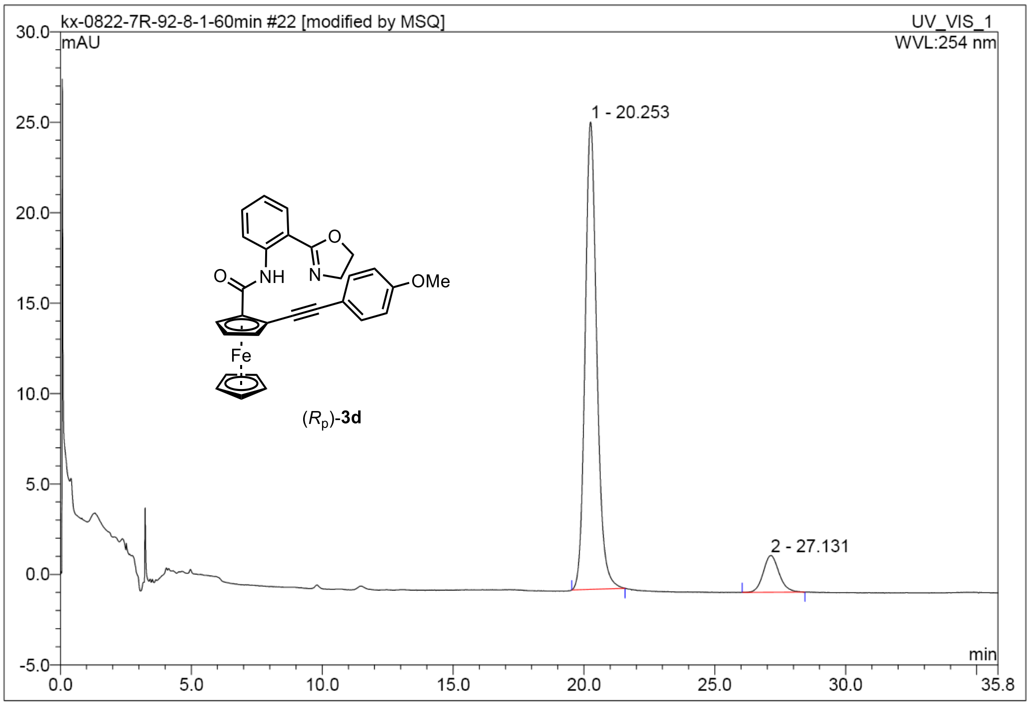

No.	Ret.Time min	Peak Name	Height mAU	Area mAU*min	Rel.Area %	Amount	Type
1	20.25	n.a.	25.842	13.358	90.40	n.a.	BMB
2	27.13	n.a.	2.026	1.419	9.60	n.a.	BMB*
**Total:**			27.868	14.777	100.00	0.000	

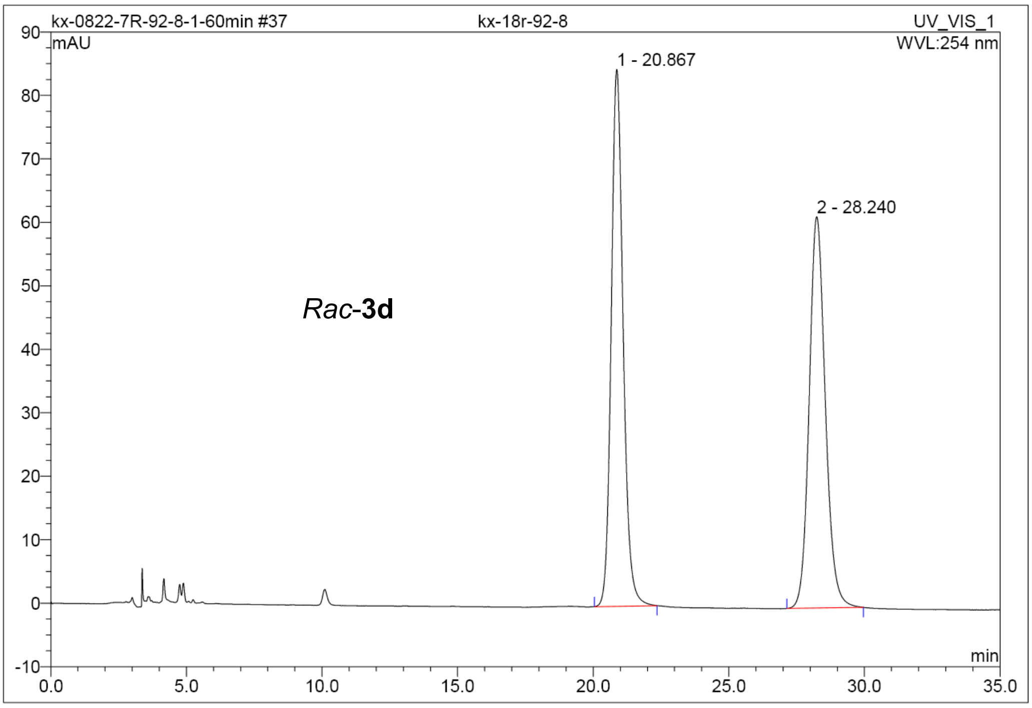

No.	Ret.Time min	Peak Name	Height mAU	Area mAU*min	Rel.Area %	Amount	Type
1	20.87	n.a.	84.609	42.927	50.22	n.a.	BMB
2	28.24	n.a.	61.650	42.544	49.78	n.a.	BMB
**Total:**			146.259	85.471	100.00	0.000	

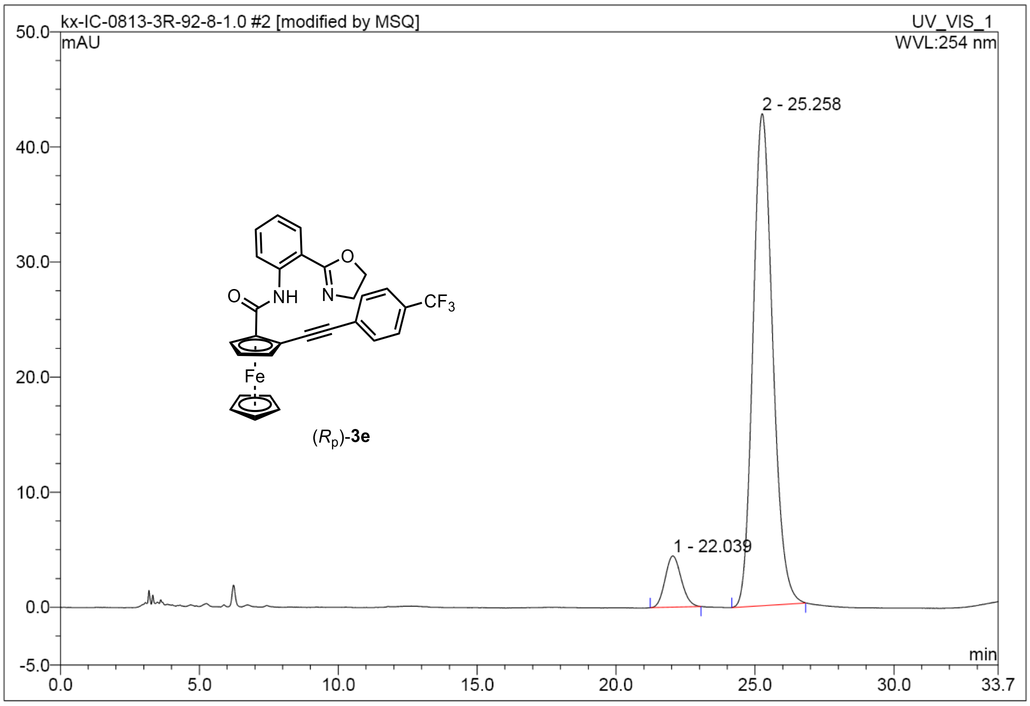

No.	Ret.Time min	Peak Name	Height mAU	Area mAU*min	Rel.Area %	Amount	Type
1	22.04	n.a.	4.451	2.995	7.86	n.a.	BMB*
2	25.26	n.a.	42.738	35.118	92.14	n.a.	BMB
**Total:**			47.189	38.113	100.00	0.000	

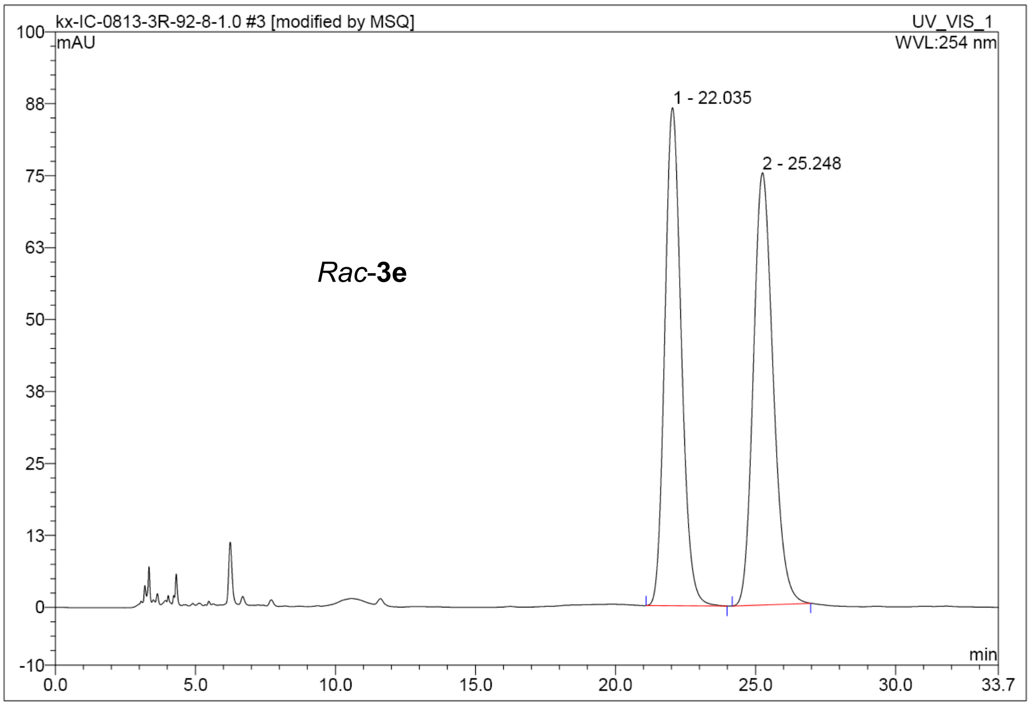

No.	Ret.Time min	Peak Name	Height mAU	Area mAU*min	Rel.Area %	Amount	Type
1	22.04	n.a.	86.525	59.223	49.11	n.a.	BMB*
2	25.25	n.a.	75.089	61.377	50.89	n.a.	BMB
**Total:**			161.614	120.600	100.00	0.000	

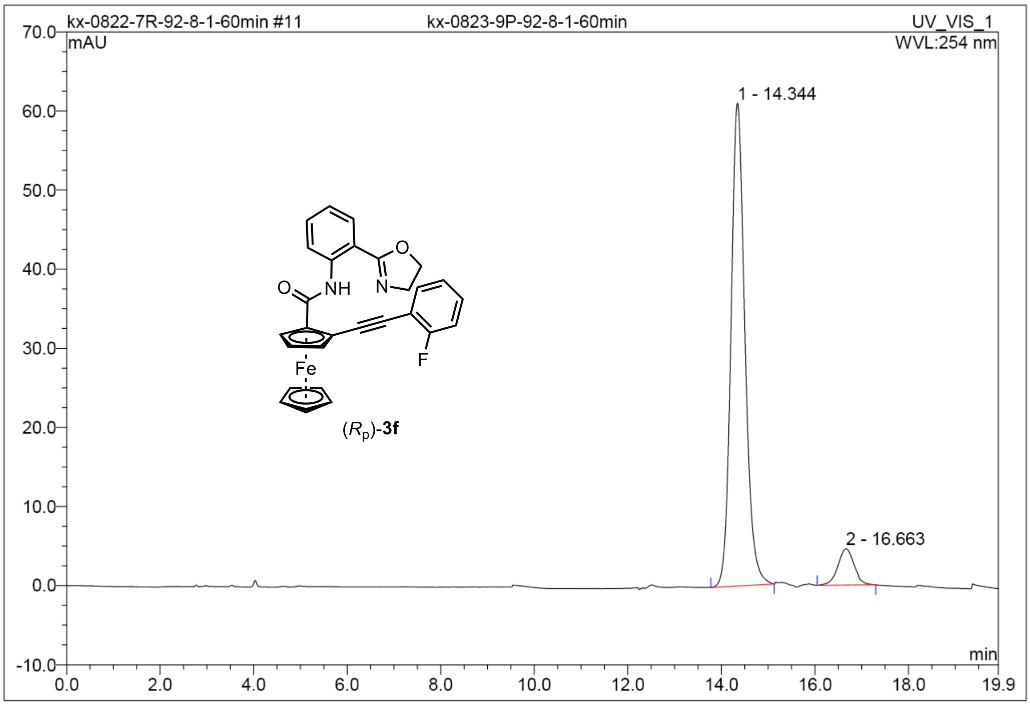

No.	Ret.Time min	Peak Name	Height mAU	Area mAU*min	Rel.Area %	Amount	Type
1	14.34	n.a.	61.022	21.075	91.93	n.a.	BMB
2	16.66	n.a.	4.585	1.850	8.07	n.a.	BMB
Total:			65.607	22.925	100.00	0.000	

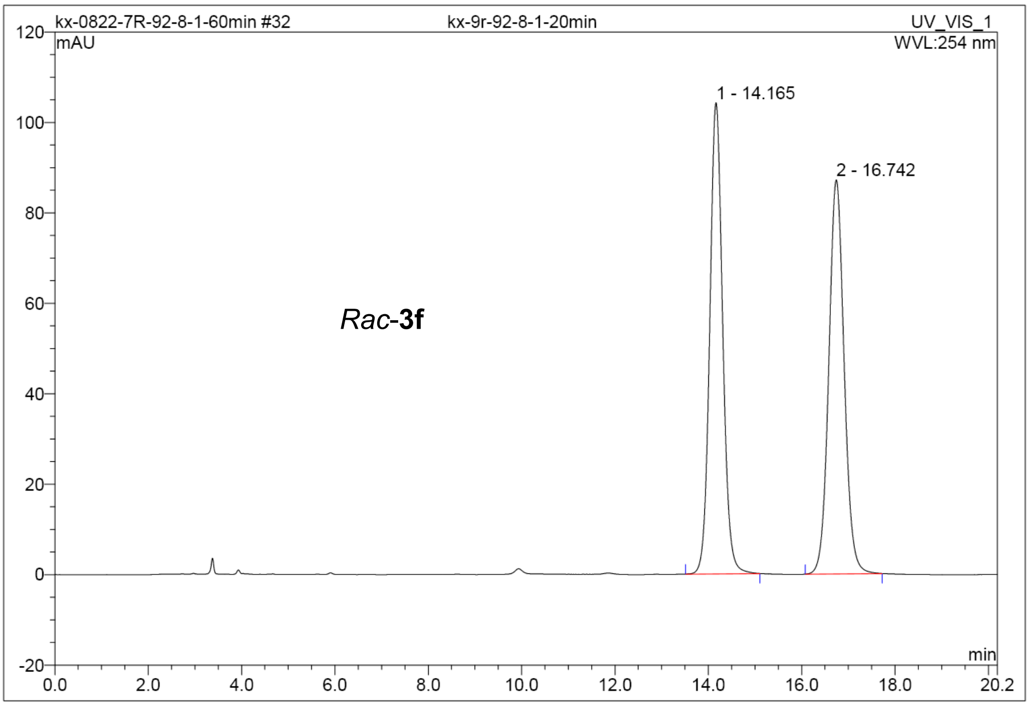

No.	Ret.Time min	Peak Name	Height mAU	Area mAU*min	Rel.Area %	Amount	Type
1	14.17	n.a.	104.190	33.373	50.14	n.a.	BMB
2	16.74	n.a.	87.144	33.189	49.86	n.a.	BMB
Total:			191.334	66.562	100.00	0.000	

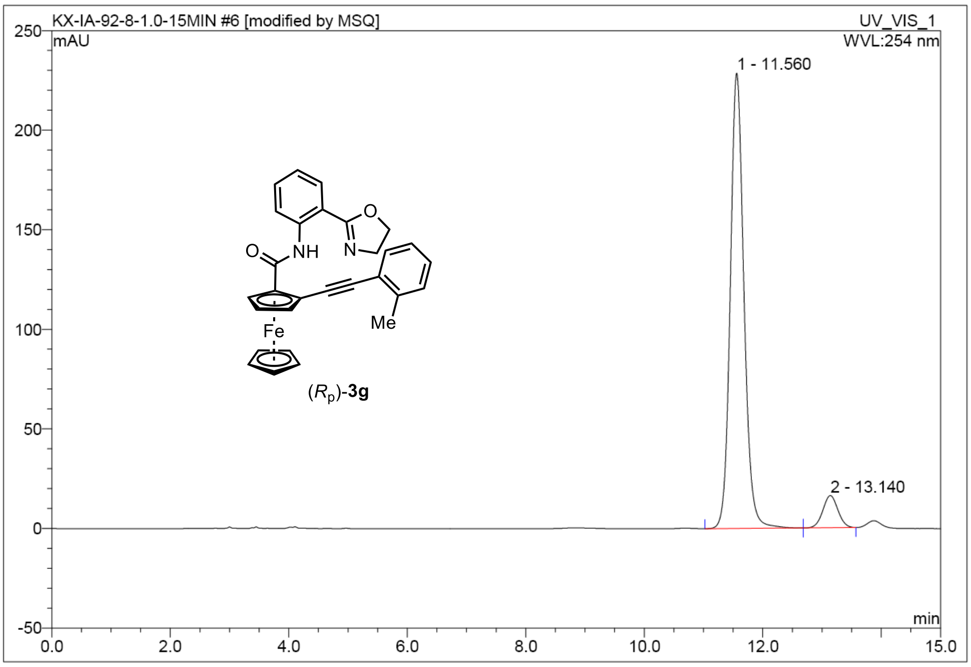

No.	Ret.Time min	Peak Name	Height mAU	Area mAU*min	Rel.Area %	Amount	Type
1	11.56	n.a.	228.622	60.909	92.75	n.a.	BM *
2	13.14	n.a.	16.173	4.762	7.25	n.a.	MB*
Total:			244.795	65.670	100.00	0.000	

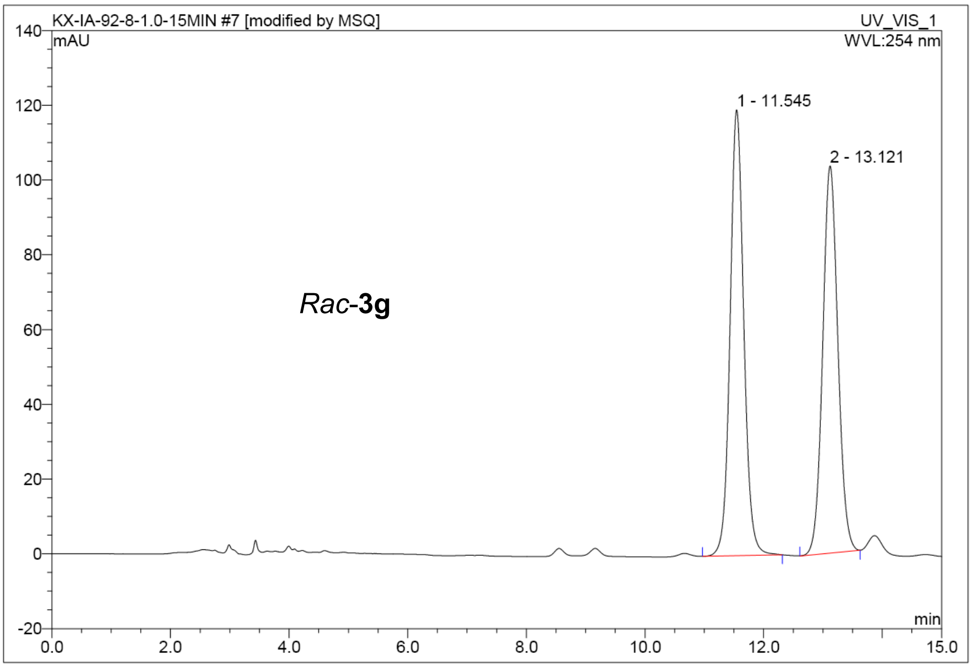

No.	Ret.Time min	Peak Name	Height mAU	Area mAU*min	Rel.Area %	Amount	Type
1	11.55	n.a.	119.287	31.858	50.94	n.a.	BMB*
2	13.12	n.a.	103.574	30.687	49.06	n.a.	BMB*
Total:			222.861	62.545	100.00	0.000	

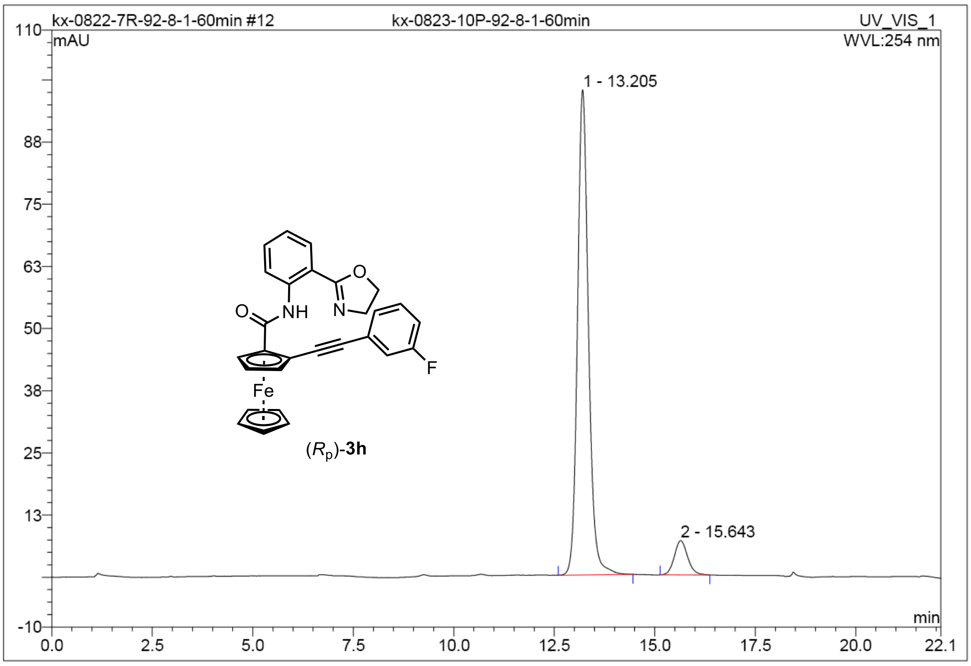

No.	Ret.Time min	Peak Name	Height mAU	Area mAU*min	Rel.Area %	Amount	Type
1	13.21	n.a.	97.539	30.826	92.08	n.a.	BMB
2	15.64	n.a.	6.903	2.650	7.92	n.a.	BMB
Total:			104.442	33.476	100.00	0.000	

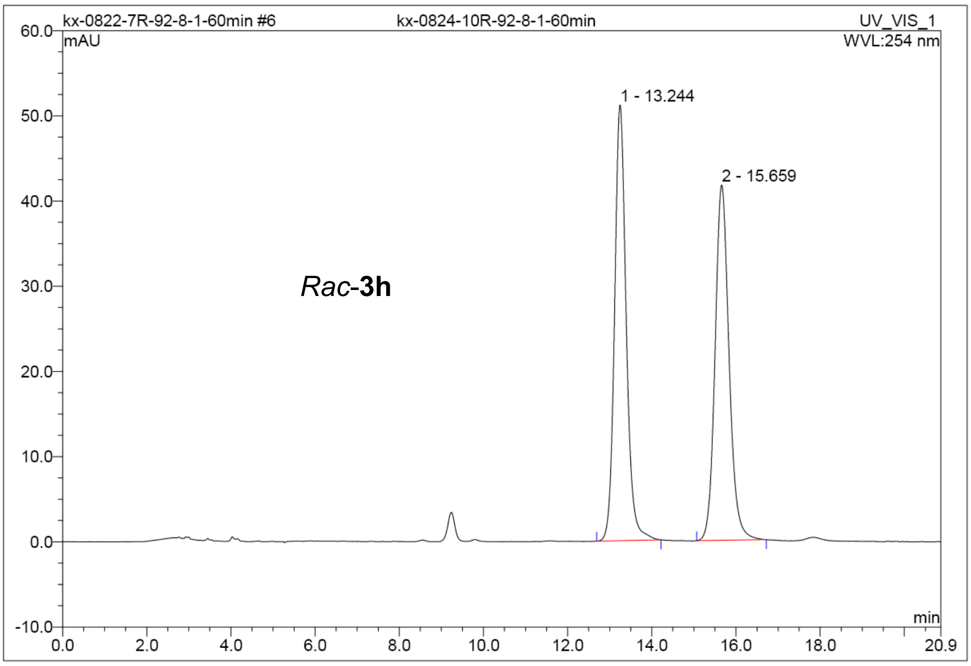

No.	Ret.Time min	Peak Name	Height mAU	Area mAU*min	Rel.Area %	Amount	Type
1	13.24	n.a.	51.137	16.357	50.50	n.a.	BMB
2	15.66	n.a.	41.700	16.033	49.50	n.a.	BMB
Total:			92.837	32.390	100.00	0.000	

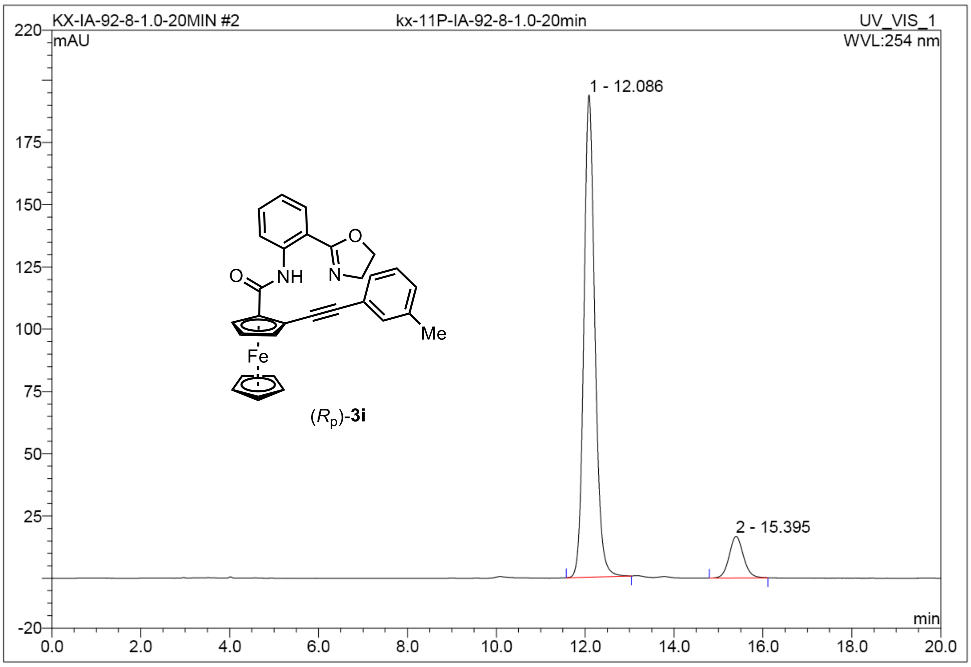

No.	Ret.Time min	Peak Name	Height mAU	Area mAU*min	Rel.Area %	Amount	Type
1	12.09	n.a.	193.627	55.520	89.96	n.a.	BMB
2	15.40	n.a.	16.718	6.196	10.04	n.a.	BMB
**Total:**			210.345	61.717	100.00	0.000	

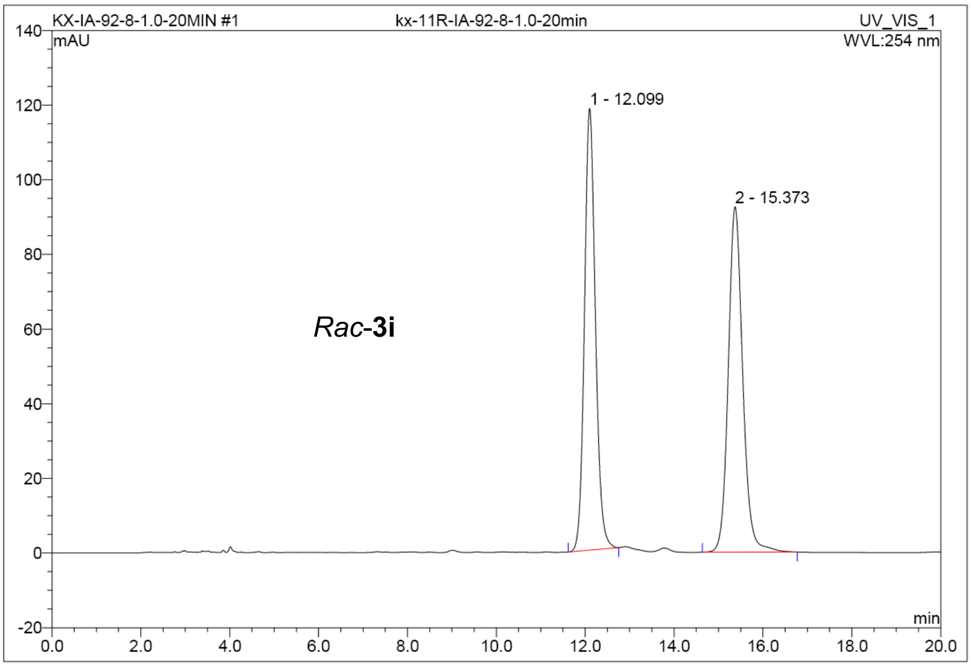

No.	Ret.Time min	Peak Name	Height mAU	Area mAU*min	Rel.Area %	Amount	Type
1	12.10	n.a.	118.357	33.724	49.19	n.a.	BMB
2	15.37	n.a.	92.507	34.839	50.81	n.a.	BMB
**Total:**			210.864	68.563	100.00	0.000	

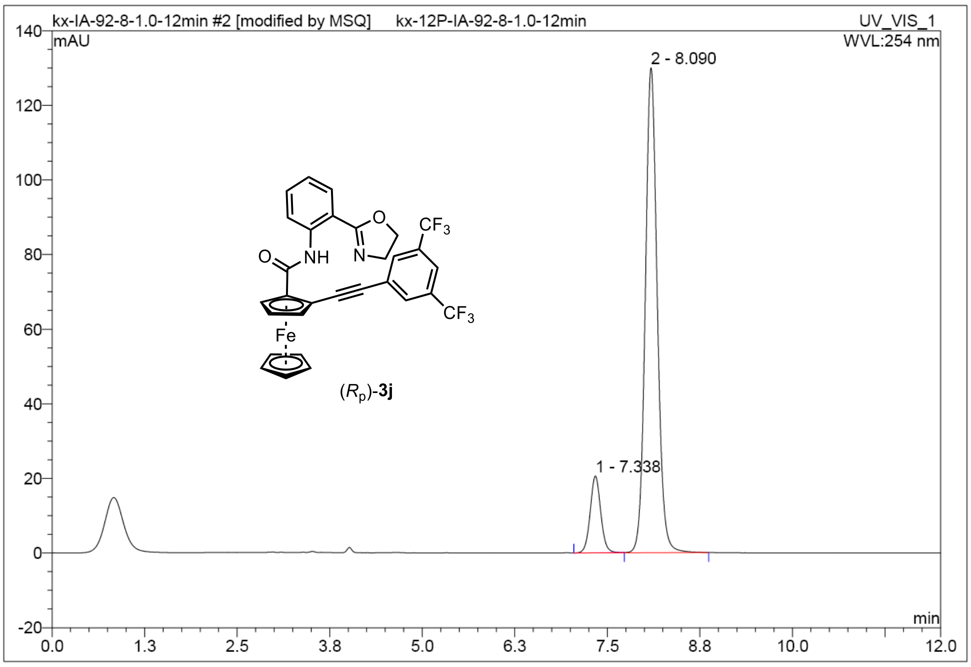

No.	Ret.Time min	Peak Name	Height mAU	Area mAU*min	Rel.Area %	Amount	Type
1	7.34	n.a.	20.582	3.399	12.26	n.a.	Ru
2	8.09	n.a.	129.979	24.327	87.74	n.a.	BMB
Total:			150.562	27.726	100.00	0.000	

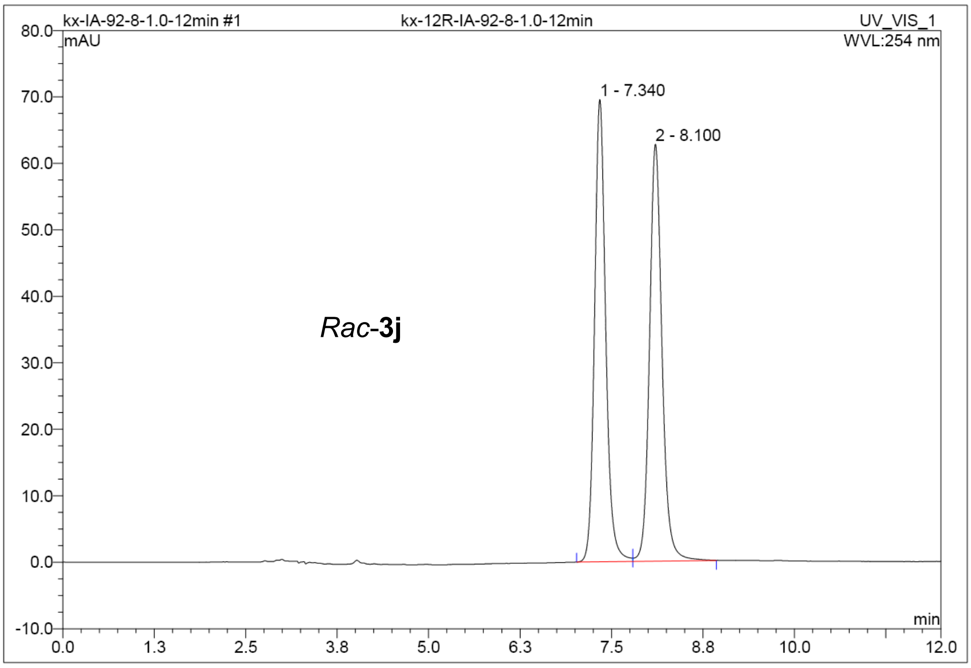

No.	Ret.Time min	Peak Name	Height mAU	Area mAU*min	Rel.Area %	Amount	Type
1	7.34	n.a.	69.555	12.316	49.77	n.a.	BM
2	8.10	n.a.	62.720	12.428	50.23	n.a.	MB
Total:			132.275	24.743	100.00	0.000	

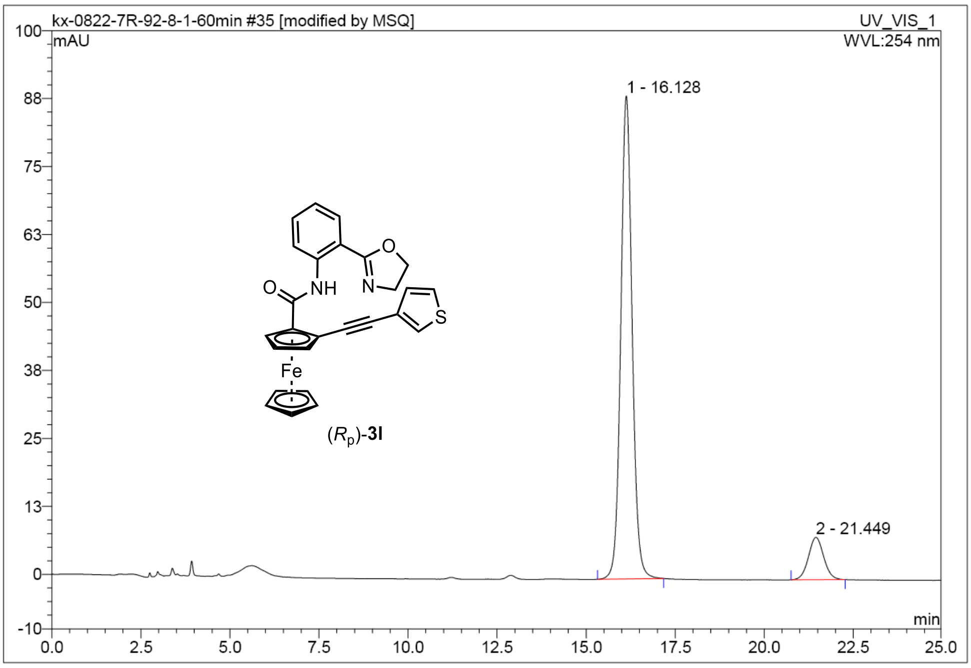

No.	Ret.Time min	Peak Name	Height mAU	Area mAU*min	Rel.Area %	Amount	Type
1	16.13	n.a.	88.820	32.797	89.48	n.a.	BMB
2	21.45	n.a.	7.784	3.857	10.52	n.a.	BMB
Total:			96.605	36.654	100.00	0.000	

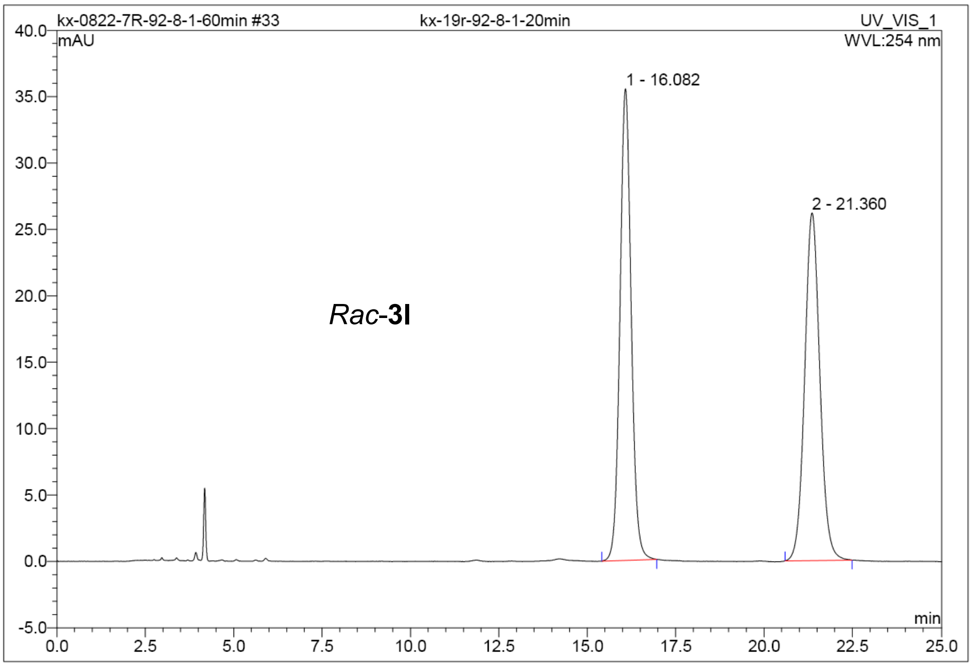

No.	Ret.Time min	Peak Name	Height mAU	Area mAU*min	Rel.Area %	Amount	Type
1	16.08	n.a.	35.521	13.085	50.07	n.a.	BMB
2	21.36	n.a.	26.189	13.047	49.93	n.a.	BMB
Total:			61.710	26.132	100.00	0.000	

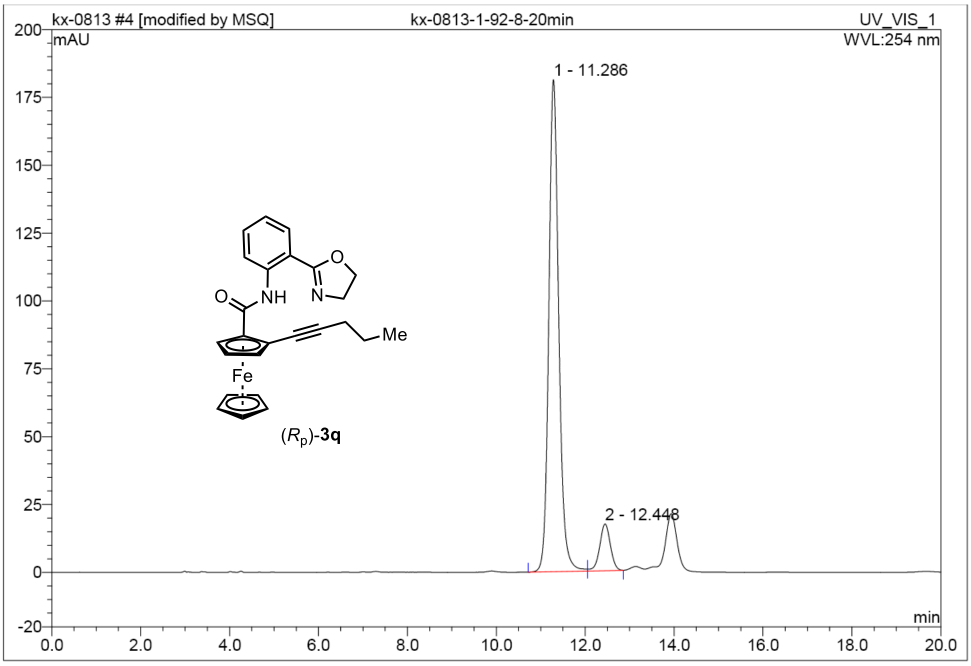

No.	Ret.Time min	Peak Name	Height mAU	Area mAU*min	Rel.Area %	Amount	Type
1	11.29	n.a.	181.273	46.911	90.74	n.a.	BM
2	12.45	n.a.	17.220	4.789	9.26	n.a.	MB
Total:			198.493	51.700	100.00	0.000	

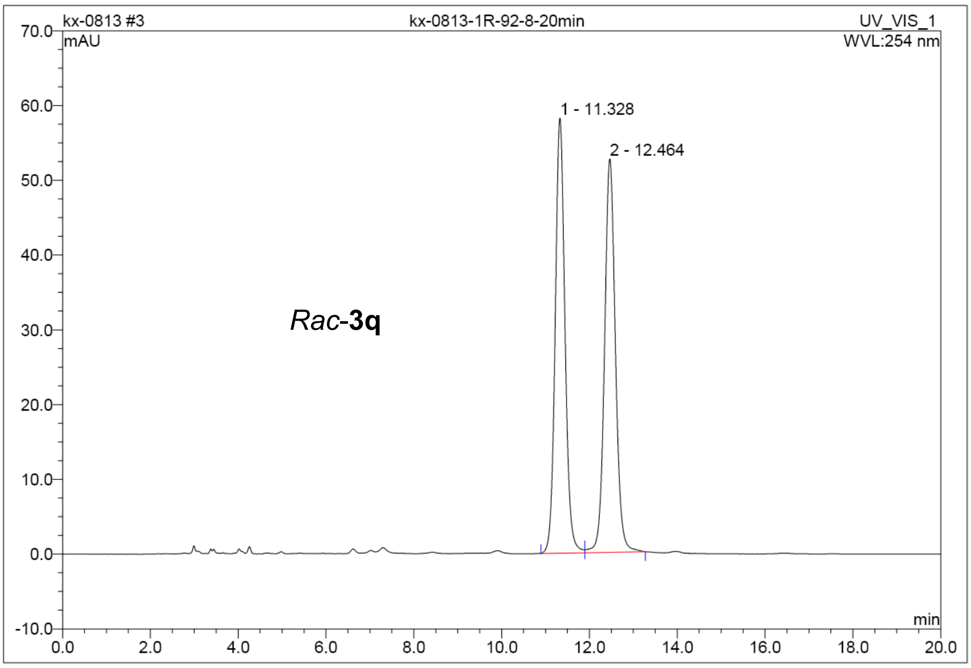

No.	Ret.Time min	Peak Name	Height mAU	Area mAU*min	Rel.Area %	Amount	Type
1	11.33	n.a.	58.217	14.956	49.82	n.a.	BM
2	12.46	n.a.	52.627	15.065	50.18	n.a.	MB
Total:			110.843	30.020	100.00	0.000	

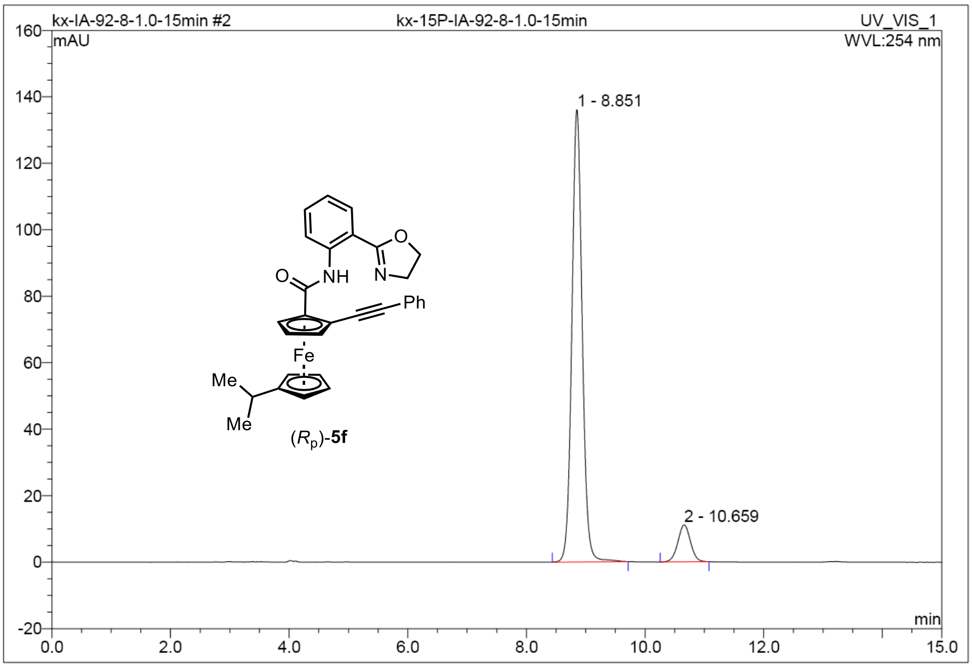

No.	Ret.Time min	Peak Name	Height mAU	Area mAU*min	Rel.Area %	Amount	Type
1	8.85	n.a.	136.090	27.413	90.87	n.a.	BMB
2	10.66	n.a.	11.138	2.753	9.13	n.a.	BMB
**Total:**			147.228	30.166	100.00	0.000	

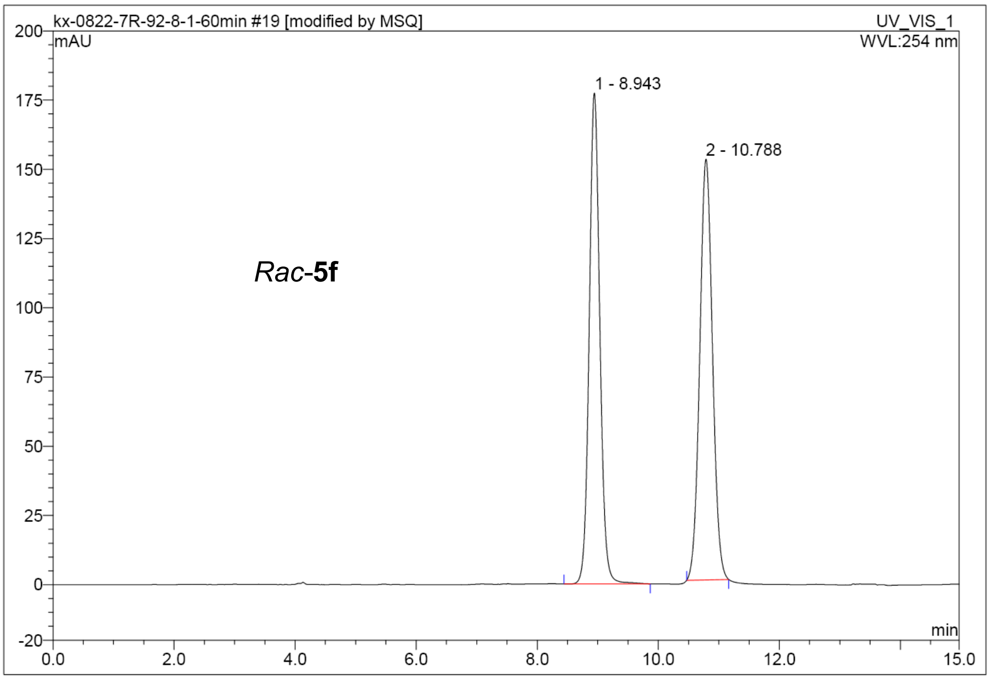

No.	Ret.Time min	Peak Name	Height mAU	Area mAU*min	Rel.Area %	Amount	Type
1	8.94	n.a.	177.250	35.957	49.32	n.a.	BMB*
2	10.79	n.a.	151.914	36.945	50.68	n.a.	BMB*
**Total:**			329.163	72.902	100.00	0.000	

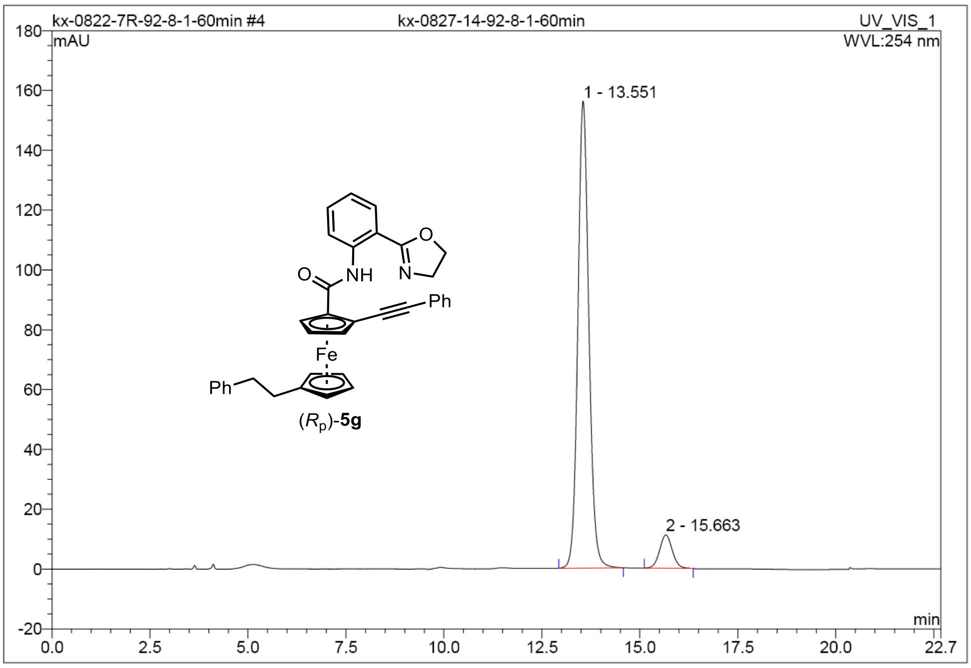

No.	Ret.Time min	Peak Name	Height mAU	Area mAU*min	Rel.Area %	Amount	Type
1	13.55	n.a.	156.124	50.695	92.40	n.a.	BMB
2	15.66	n.a.	11.128	4.171	7.60	n.a.	BMB
**Total:**			167.253	54.866	100.00	0.000	

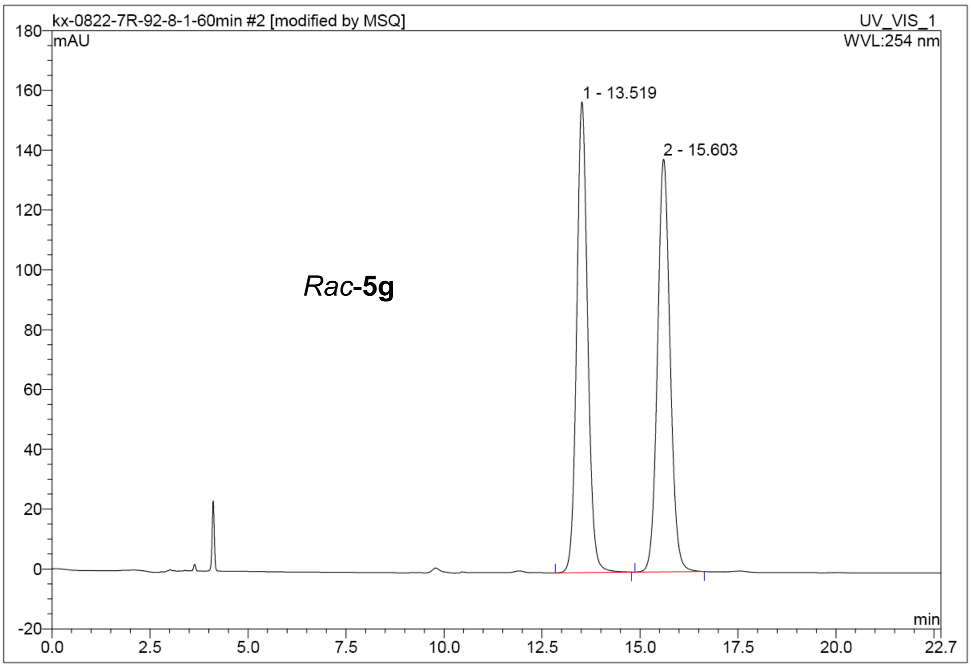

No.	Ret.Time min	Peak Name	Height mAU	Area mAU*min	Rel.Area %	Amount	Type
1	13.52	n.a.	157.382	51.099	49.82	n.a.	BMB
2	15.60	n.a.	138.009	51.477	50.18	n.a.	BMB
**Total:**			295.392	102.576	100.00	0.000	

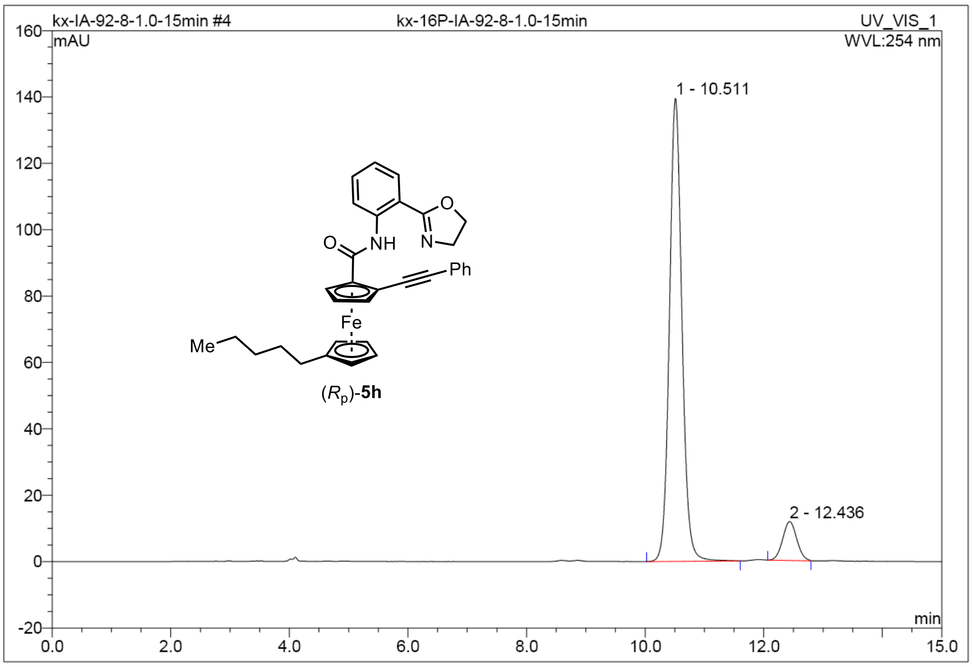

No.	Ret.Time min	Peak Name	Height mAU	Area mAU*min	Rel.Area %	Amount	Type
1	10.51	n.a.	139.512	33.993	91.28	n.a.	BMB
2	12.44	n.a.	11.728	3.246	8.72	n.a.	BMB
**Total:**			151.240	37.239	100.00	0.000	

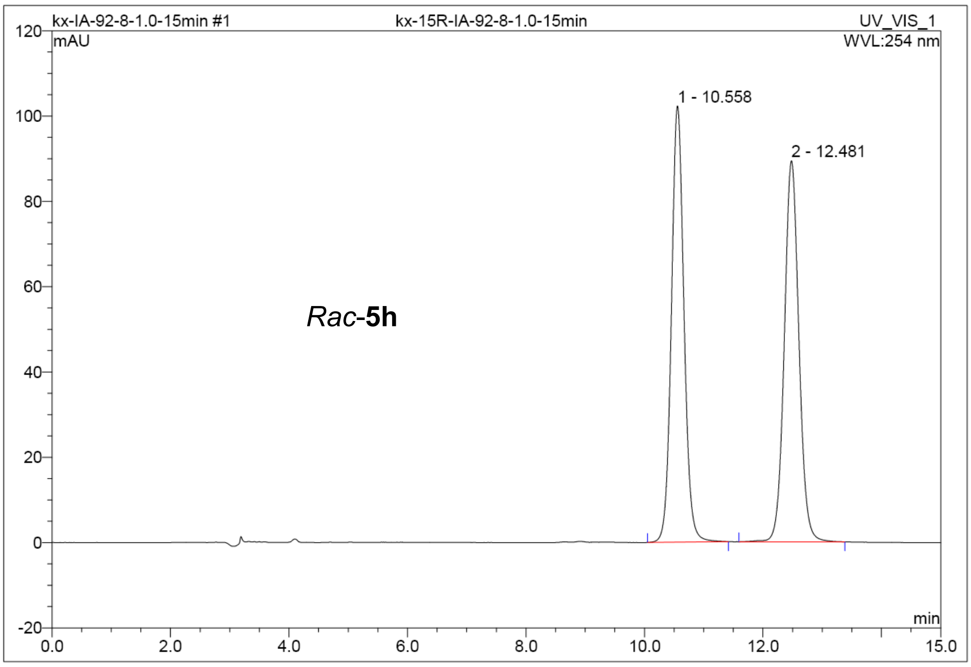

No.	Ret.Time min	Peak Name	Height mAU	Area mAU*min	Rel.Area %	Amount	Type
1	10.56	n.a.	102.271	25.157	49.58	n.a.	BMB
2	12.48	n.a.	89.337	25.579	50.42	n.a.	BMB
**Total:**			191.608	50.735	100.00	0.000	

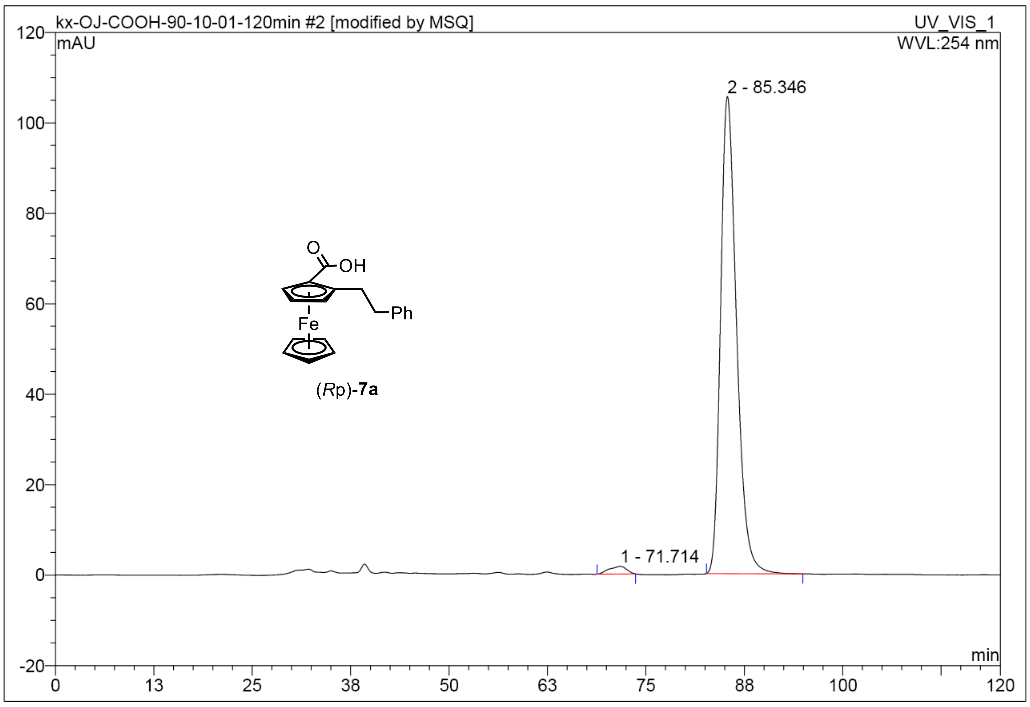

No.	Ret.Time min	Peak Name	Height mAU	Area mAU*min	Rel.Area %	Amount	Type
1	71.71	n.a.	1.704	4.399	1.75	n.a.	BMB*
2	85.35	n.a.	105.497	247.513	98.25	n.a.	BMB*
Total:			107.202	251.912	100.00	0.000	

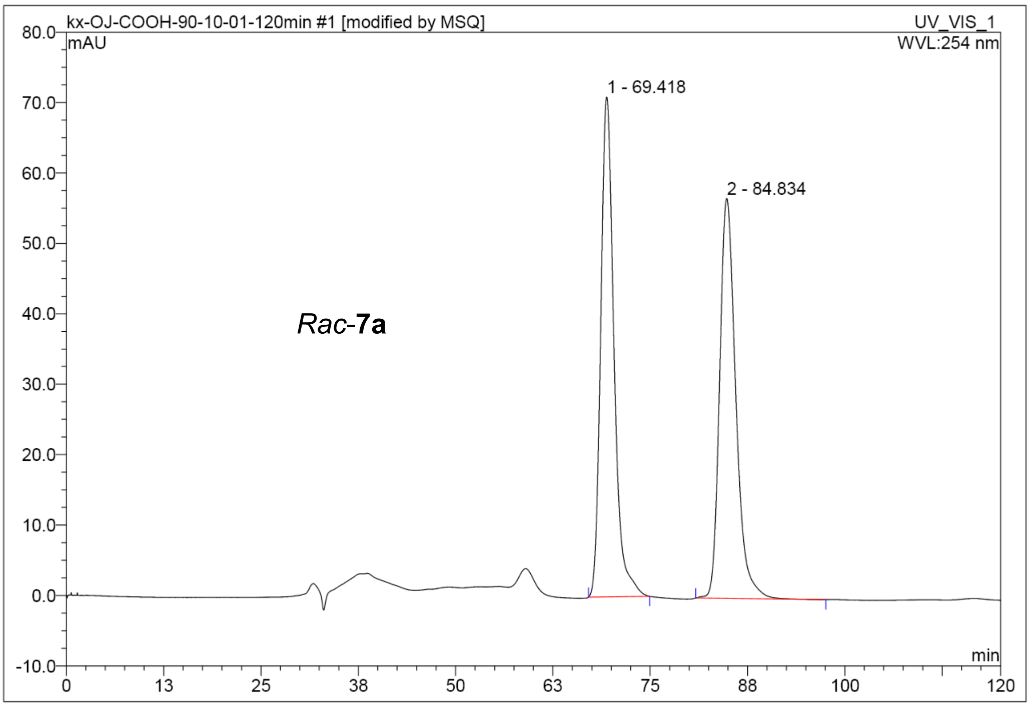

No.	Ret.Time min	Peak Name	Height mAU	Area mAU*min	Rel.Area %	Amount	Type
1	69.42	n.a.	71.002	140.039	50.57	n.a.	BMB*
2	84.83	n.a.	56.828	136.897	49.43	n.a.	BMB*
Total:			127.830	276.936	100.00	0.000	

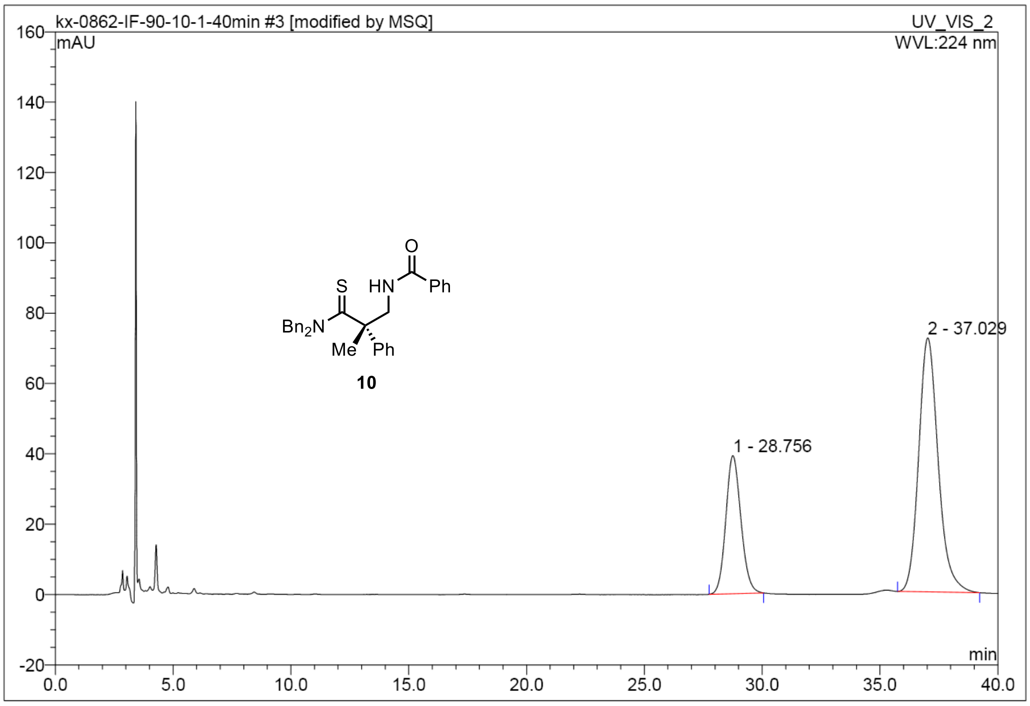

No.	Ret.Time min	Peak Name	Height mAU	Area mAU*min	Rel.Area %	Amount	Type
1	28.76	n.a.	39.255	29.176	29.64	n.a.	BMB
2	37.03	n.a.	72.219	69.266	70.36	n.a.	BMB
Total:			111.474	98.442	100.00	0.000	

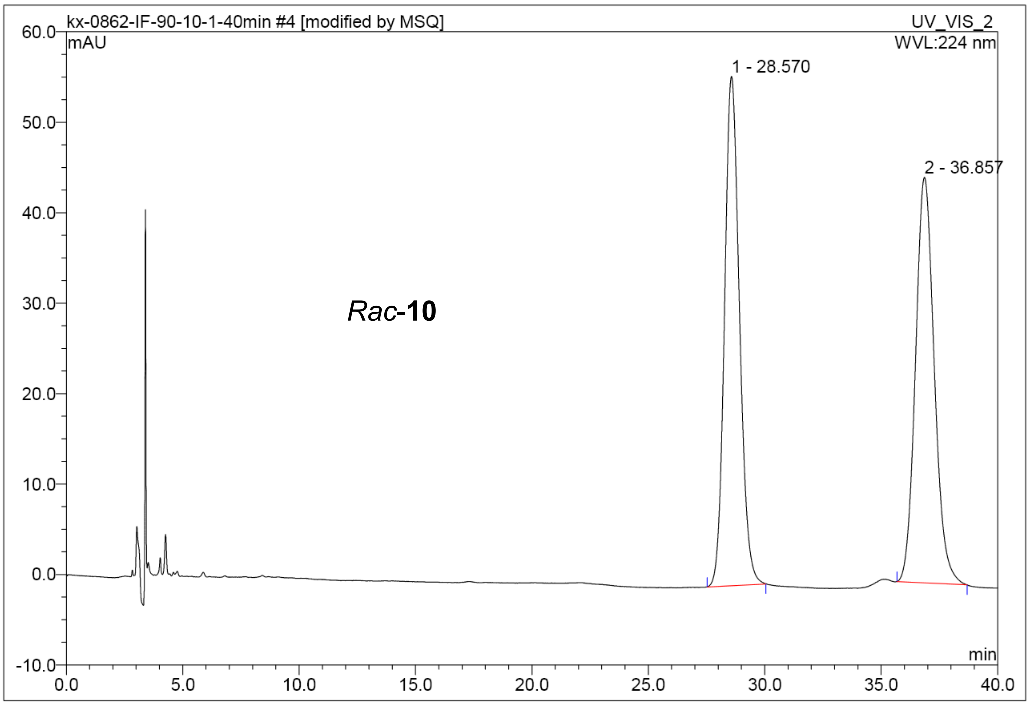

No.	Ret.Time min	Peak Name	Height mAU	Area mAU*min	Rel.Area %	Amount	Type
1	28.57	n.a.	56.319	41.895	49.98	n.a.	BMB
2	36.86	n.a.	44.836	41.922	50.02	n.a.	BMB
Total:			101.156	83.817	100.00	0.000	

6. Supplementary References

1. Wu, T. R., Shen, L. & Chong J. M. Asymmetric Allylboration of Aldehydes and Ketones Using 3,3'-Disubstitutedbinaphthol-Modified Boronates. *Org. Lett.* **6**, 2701- 2704 (2004).
2. Rueping, M., Nachtsheim, B. J., Koenigs, R. M. & Ieawsuwan, W. Synthesis and Structural Aspects of *N*-Triflylphosphoramides and TheirCalcium Salts—Highly Acidic and Effective Brønsted Acids. *Chem. Eur. J.* **16**, 13116-13126 (2010).
3. Gao, G., Gu, F.-L., Jiang, J.-X., Jiang, K., Sheng, C.-Q., Lai, G.-Q. & Xu, L.-W. Neighboring Lithium-Assisted [1,2]-Wittig Rearrangement: Practical Accessto Diarylmethanol-Based 1,4-Diols and Optically Active BINOL Derivativeswith Axial and sp^3-Central Chirality. *Chem. Eur. J.* **17**, 2698-2703 (2011).
4. Théveau, L., Bellini, R., Dydio, P., Szabo, Z., van der Werf, A., Afshin Sander, R., Reek, J. N. H. & Moberg, C. Cofactor-Controlled Chirality of Tropoisomeric Ligand. *Organometallics* **35**, 1956-1963 (2016).
5. More, N. Y. & Jeganmohan, M. Solvent-controlled selective synthesis of biphenols and quinones via oxidative coupling of phenols. *Chem. Commun.* **53**, 9616-9619 (2017).
6. Han, Y.-Q., Ding, Y., Zhou, T., Yan, S.-Y., Song, H. & Shi, B.-F. Pd(II)-Catalyzed Enantioselective Alkynylation of Unbiased Methylene C(sp^3) – H Bonds Using 3,3′ -Fluorinated-BINOL as a Chiral Ligand. *J. Am. Chem. Soc.* **141**, 4558-4563 (2019).
7. Jia, Z.-S., Yue, Q. M., Li, Y. M., Xu, X.-T. M., Zhang, K. & Shi, B.-F. Copper-catalyzed monoselective C–H amination of ferrocenes with alkylamines. *Beilstein J. Org. Chem.* **17**, 2488-2495 (2021).
8. Bildstein, B., Hradsky, A., Kopacka, H., Malleier, R. & Ongania, K.-H. Functionalized pentamethylferrocenes: synthesis, structure, and electrochemistry. *J. Organomet. Chem.* **540**, 127-145 (1997).
9. Garra, P., Brunel, D., Noirbent, G., Graff, B., Morlet-Savary, F., Dietlin, C., Sidorkin, V. F., Dumur, F., Duché, D., Gigmes, D., Fouassier, J.-P. & Lalevée, J. Ferrocene-based (photo)redox polymerization under long wavelengths. *Polym. Chem.* **10**, 1431-1441 (2019).
